# Copper(I)-catalyzed asymmetric alkylation of α-imino-esters

**DOI:** 10.1038/s41467-023-37967-y

**Published:** 2023-04-17

**Authors:** Zong-Ci Liu, Zi-Qing Wang, Xuan Zhang, Liang Yin

**Affiliations:** CAS Key Laboratory of Synthetic Chemistry of Natural Substances, Center for Excellence in Molecular Synthesis, Shanghai Institute of Organic Chemistry, University of Chinese Academy of Sciences, Chinese Academy of Sciences, 345 Lingling Road, 200032 Shanghai, China

**Keywords:** Synthetic chemistry methodology, Asymmetric synthesis

## Abstract

Asymmetric alkylation of enolates is one of the most direct and important reactions to prepare α-chiral carbonyl compounds. Except for the classical methods that rely on the use of chiral auxiliaries, asymmetric catalysis emerged as a powerful tool, especially asymmetric phase-transfer catalysis. However, in the field of transition metal catalysis, only limited success with asymmetric alkylation of enolates was achieved. Hereby, we disclose a copper(I)-catalyzed asymmetric alkylation of α-imino-esters with various alkyl halides, including allyl bromides, propargyl bromide, benzyl bromides, α-bromo carbonyl compounds, and alkyl iodides. Both linear and cyclic α-imino-esters serve as competent pronucleophiles in the alkylation, which affords α-amino acid derivatives bearing either a trisubstituted or a tetrasubstituted stereogenic carbon center in high to excellent enantioselectivity. Control experiments indicate that the α-imino-ester is activated by a chiral copper(I)-phosphine complex through coordination, thus enabling facile deprotonation to provide a stabilized copper(I)-enolate in the presence of a mild base. Finally, the mildly basic nature allows the asymmetric alkylation of chiral dipeptides with excellent both chemo- and enantioselectivities.

## Abstract (web summary)

Asymmetric alkylation of enolates is an important method for the formation of α-stereogenic carbonyls but generally requires the introduction and then removal of chiral auxiliaries. Here, the authors report a copper-catalyzed asymmetric alkylation of α-amino esters.

## Introduction

**Fig. 1:**
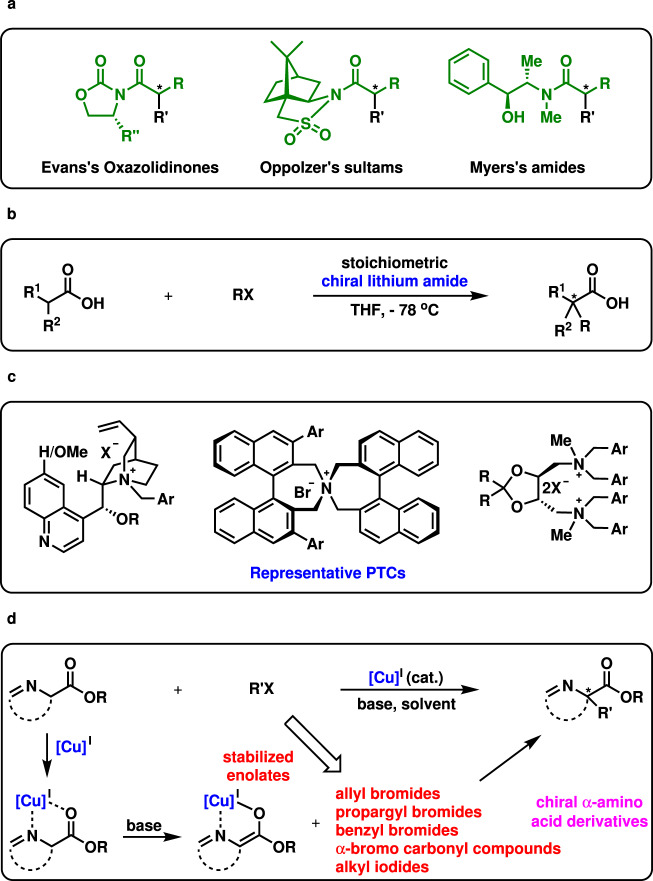
Introduction to asymmetric alkylation of enolates and our working hypothesis. **a** Chiral auxiliaries-induced asymmetric alkylation of enolates with R’X. **b** Asymmetric alkylation of enolates with chiral lithium amides. **c** Catalytic asymmetric alkylation of enolates with phase-transfer catalysts (PTCs). **d** Copper(I)-catalyzed asymmetric alkylation of stabilized enolates: this work.

Asymmetric alkylation of enolates is one of the most fundamental and important methods for the construction of ubiquitous α-stereogenic carbonyl motifs^1–3^ (Due to the limited space, deprotonative alkylation with alkyl halides is mainly discussed in this manuscript.). Tremendous achievements originated from both diastereoselective and enantioselective enolate alkylations. Trustable chiral auxiliaries, such as Evans’s oxazolidinones^4,5^, Oppolzer’s sultams^6^, and Myers’s amides^7–11^, have contributed significantly to the successful control of the diastereoselectivity in the asymmetric alkylations (Fig. 1a). Usually, such alkylations enjoy high to excellent stereoselectivity, predictable stereochemistry in the newly generated chiral carbon centers, and broad substrate scopes. However, the tedious introduction and subsequent removal of the stoichiometric auxiliaries make these methodologies less synthetically efficient. For a study on stereoselectivity and mechanism, see ref. ^5^.

Enantioselective alkylation of prochiral enolates was performed with carbonyl compounds and stoichiometric chiral lithium bases at generally low temperatures^1–3^. The preliminary works on such a topic suffered from low enantioselectivity^12,13^. Encouragingly in 1990, Koga and coworkers carried out the enantioselective alkylation of cyclohexanone and tetralone with benzyl bromide, cinnamyl bromide, allyl bromide, and methyl iodide in moderate to high enantioselectivity^14^. Later, the pronucleophiles were successfully extended to lactams and lactones^15^. Since then, significant efforts have been devoted to this field^1–3,16–19^. Particularly noteworthy is a highly enantioselective alkylation of arylacetic acids with stoichiometric chiral lithium amides as traceless auxiliaries (Fig. 1b, R^1^ = Ar, R^2^ = H)^16,17^. Later, this chemistry was successfully extended to the construction of chiral all-carbon quaternary stereogenic centers^18^ (Fig. 1b, R^2^ = Me or MeO) and the α-alkylation of β,γ-unsaturated carboxylic acids^19^. Uniformly high enantioselectivities were obtained in these reactions.

In addition, asymmetric phase-transfer catalysis is identified as a wonderful tool for alkylations (Fig. 1c)^20–26^. Generally speaking, allyl bromides, propargyl bromides, benzyl bromides, and alkyl iodides served as competent electrophiles with high both yields and enantioselectivity. However, substrates bearing a strong base-sensitive either group or stereogenic center are generally not well tolerated. Therefore, it is highly desirable to develop efficient metal-catalyzed asymmetric alkylation of enolates under mildly basic conditions, which is complementary to phase-transfer catalysis. Transition metal-catalyzed asymmetric allylic alkylation^27–32^ and propargyl alkylation^33–38^ emerged as powerful methods for the alkylations of both stabilized enolates and non-stabilized enolates. However, the electrophiles are generally limited to close derivatives of allyl and propargyl alcohols. Moreover, bimetallic catalysis enabled catalytic asymmetric allylation of enolates^39–43^. Evidently, metal-catalyzed asymmetric alkylation of enolates, which holds a broad scope for alkyl halides, is rare and remains elusive^44–53^.

It is known that stabilized copper(I)-enolate complexes would be generated by introducing an addition coordination group on the carbonyl compounds (Fig. 1d)^54,55^. Such a chelation effect would allow facile deprotonation under mildly basic conditions and be helpful for high enantioselectivity. More importantly, the chelation would prevent the formation of catalytically inactive dimeric copper species caused by the halide bridging effect^56^. Thus α-imino-esters are chosen as the substrates for investigation. Remarkably, the produced α-amino acid derivatives are extremely important in medicinal chemistry, biochemistry, material chemistry, and organic synthesis^20–26^. More appealingly, the mild reaction conditions would allow facile catalytic asymmetric alkylation of chiral peptides.

Here, we show a copper(I)-catalyzed asymmetric alkylation of enolates generated from α-imino-esters, which affords important chiral α-amino acid derivatives in high to excellent enantioselectivity. As for pronucleophiles, both linear and cyclic α-imino-esters work very well. As for electrophiles, allyl bromides, propargyl bromide, benzyl bromides, α-bromo carbonyl compounds, and alkyl iodides serve as competent substrates. Evidently, the present methodology is comparable to the classical asymmetric phase-transfer catalysis in the asymmetric alkylation of α-imino-esters. Control experiments show that α-imino-esters are activated by coordination with a copper(I)-phosphine complex, furnishing stabilized copper(I)-enolates under mildly basic conditions. Moreover, the present alkylation is proposed to proceed through an S_N_2 substitution mechanism. Finally, the methodology is successfully applied in the asymmetric alkylation of chiral dipeptides without both *N-*alkylation and epimerization.

## Results and discussion

### Optimization of reaction conditions

**Fig. 2:**
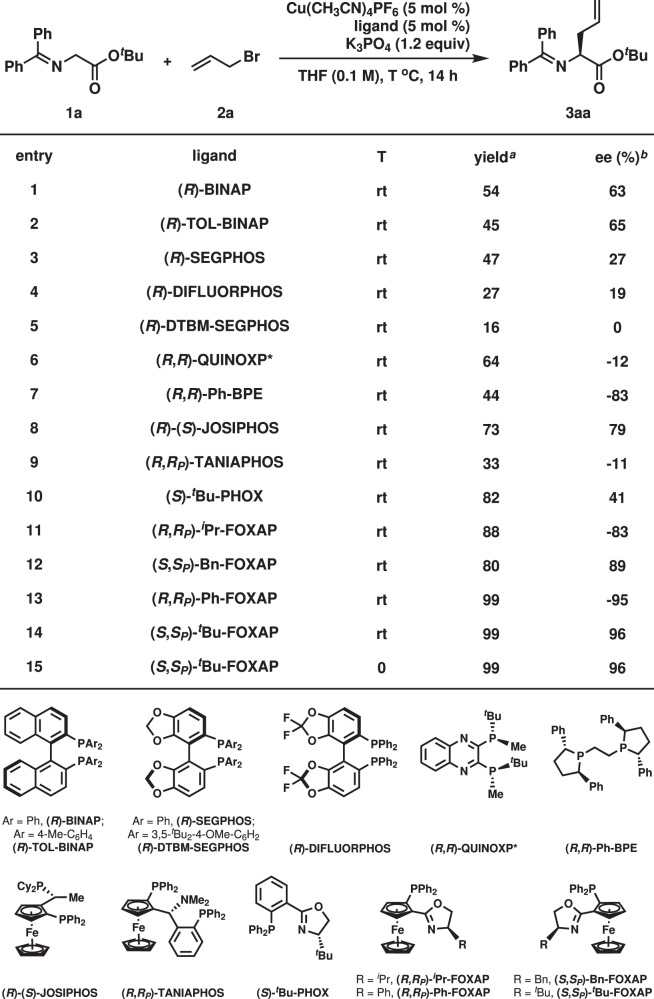
Optimization of reaction conditions. Reaction conditions: **1a** (0.15 mmol), **2a** (0.10 mmol). ^*a*^ Determined by ^1^H NMR analysis of reaction crude mixture using CH_2_Br_2_ as an internal standard. ^*b*^ Determined by chiral stationary-phase HPLC analysis.

The optimization of reaction conditions is described in Fig. 2 by using α-imino-ester **1a** and allyl bromide (**2a**) as the model substrates. In the presence of 5 mol % Cu(CH_3_CN)_4_PF_6_, 5 mol % (*R*)-BINAP, and 1.2 equiv. K_3_PO_4_, the desired alkylation proceeded in 54% yield with 63% ee at room temperature (entry 1). Screening of several commercially available phosphine ligands bearing either a chiral axis, or two stereogenic centers, or a chiral plane identified (*R*,*R*)-Ph-BPE as the superior ligand as product **3aa** was generated in 44% yield with 83% ee (entries 2–9). P,N-Ligands, such as (*S*)-^*t*^Bu-PHOX and (*R*,*R*_*P*_)-^*i*^Pr-FOXAP were also tested (entries 10–11). The reaction with (*R*,*R*_*P*_)-^*i*^Pr- FOXAP afforded **3aa** in 88% yield with 83% ee (entry 11). Further study on a substituent effect on the oxazoline group revealed (*S*,S_P_)-^*t*^Bu-FOXAP as the best ligand as **3aa** was furnished in 99% yield with 96% ee (entry 12–14). The reaction at 0 °C afforded the same results (entry 15).

### Substrate scope investigation

**Fig. 3:**
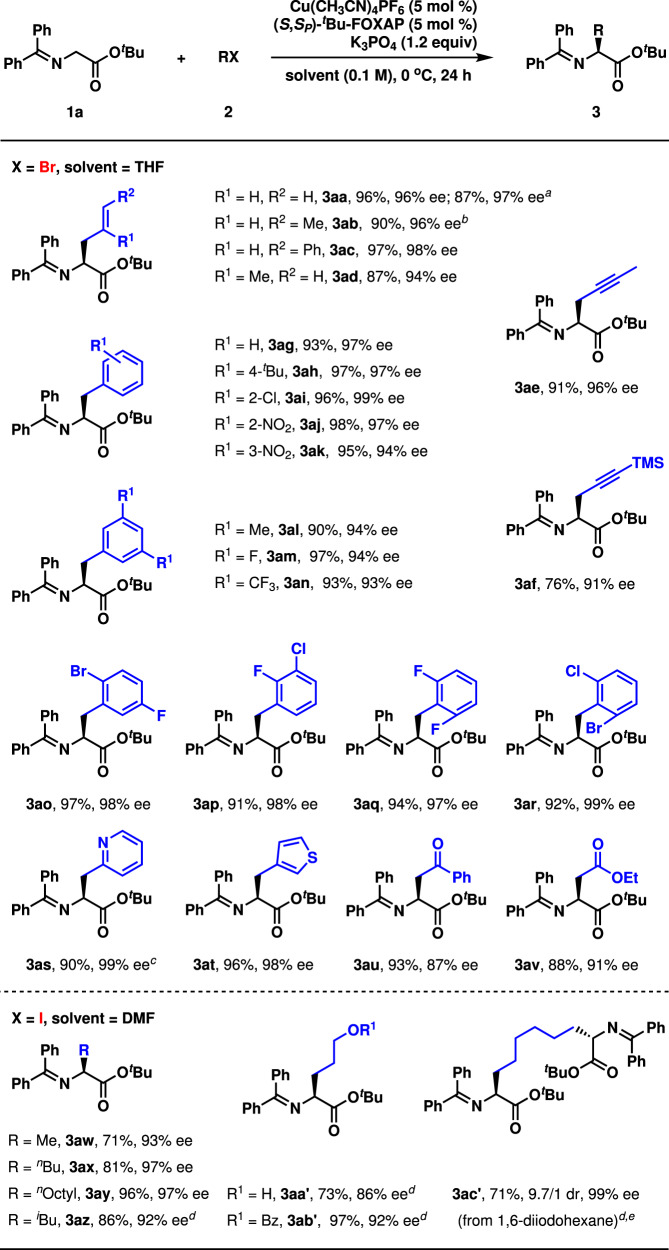
Substrate scope I. Reaction conditions: **1a** (0.3 mmol), **2** (0.2 mmol). Isolated yields were reported. Ee and dr were determined by chiral stationary-phase HPLC analysis. ^*a*^ Gram-scale reaction with 0.5 mol % copper catalyst. rt. 48 h. ^*b*^ (*E*)/(*Z*) = 6/1. **2b** with 5/1 (*E*)/(*Z*) was employed. ^*c*^ HBr salt of **2s** and 2.4 equiv. K_3_PO_4_ were used. ^*d*^ rt. ^*e*^ 3.0 equiv. **1a** and 2.4 equiv. K_3_PO_4_ were used.

With optimized reaction conditions in hand, the substrate scope of alkyl halides with α-imino-ester **1a** was investigated at 0 °C due to the higher enantioselectivity in most cases than at room temperature (Fig. 3). Several allyl bromides with either a terminal olefin or an internal olefin worked as competent electrophiles as the corresponding products were isolated in satisfying results (**3aa**-**3ad**, 87–97%, 94–98% ee). Moreover, propargyl bromide **2e** and **2f** served as wonderful alkylation reagents (**3ae-3af**, 76–91%, 91–96% ee). The reactions with several benzyl bromides bearing a substituent on the phenyl ring proceeded in excellent both yield and enantioselectivity (**3ag**-**3ak**, 93–98%, 94–99% ee), which were not significantly affected by the position of the substituent. Benzyl bromides containing two substituents on the phenyl ring were suitable electrophiles, too (**3al**-**3ar**, 90–97%, 93–99% ee). It is remarkable that two heterobenzyl bromides (**2s** and **2t**) served as wonderful electrophiles (**3as**-**3at**, 90–96%, 98–99% ee). Strikingly, both α-bromoacetophenone (**2u**) and ethyl α-bromoacetate (**2v**) were successfully applied in the alkylation, which provided chiral 1,4-dicarbonyl compounds in satisfactory results (**3au**-**3av**, 88–93%, 87–91% ee). It is well known that the asymmetric synthesis of chiral 1,4-dicarbonyl compounds remains a continuing challenge in organic chemistry^57–62^. Moreover, it is noteworthy that **3au** was produced in very low yield with a byproduct under classical asymmetric phase-transfer catalysis^63^. Alkyl iodides were also appropriate alkylation reagents in DMF (**3aw**-**3ab′**, 71–97%, 86–97% ee). It should be noted that a strong base-sensitive benzoate group is present in **3ab′**, and some reactions were set up at room temperature for higher yields (**3az**, **3aa′**, and **3ab′**). A double alkylation occurred in satisfying results, too (**3ac′**, 71%, 9.7/1 dr, 99% ee). It should be noted that the dialkylated products were not observed in these reactions. The gram-scale reaction of **1a** and **2a** was successfully performed in the presence of 0.5 mol % copper(I) catalyst at room temperature, providing 2.92 gram **3aa** in 87% yield with 97% ee. The absolute configuration of **3aw** was determined to be *S* by comparing its optical rotation value with the reported date (for the details, see Supplementary Information). The stereochemistry of other products was deduced by analogy.

**Fig. 4:**
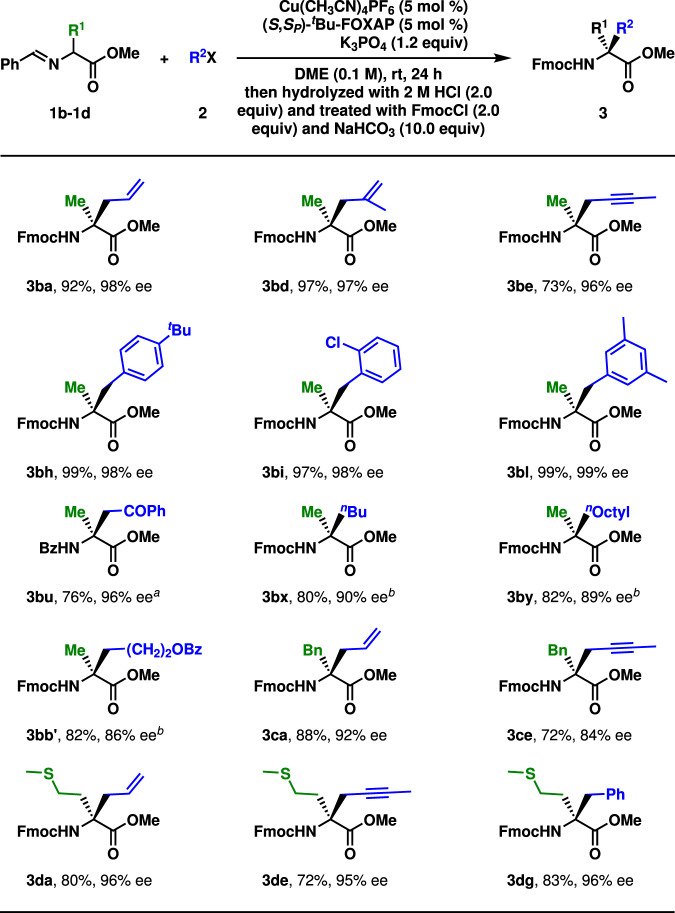
Substrate scope II. Reaction conditions: **1** (0.3 mmol), **2** (0.2 mmol). Isolated yields were reported. Ee was determined by chiral stationary-phase HPLC analysis. ^*a*^ BzCl was used instead of FmocCl. ^*b*^ Cs_2_CO_3_ was employed instead of K_3_PO_4_. 80 °C. 12 h.

**Fig. 5:**
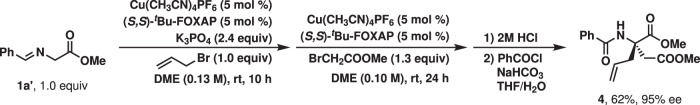
Consecutive asymmetric alkylation in one pot. Reaction conditions: **1a’** (0.2 mmol), allyl bromide (0.2 mmol), and methyl α-bromoacetate (0.26 mmol).

Then the construction of tetrasubstituted carbons through the alkylation was studied, as given in Fig. 4. In these reactions, DME was a superior solvent to others. For the sake of easy manipulation, the labile products from the alkylation were cleaved at acidic conditions and protected by a Fmoc group. Considering the bulky nature of tetrasubstituted stereogenic centers, these reactions were performed at room temperature. By using α-imino-ester **1b** as the pronucleophile, the allylation (**3ba** and **3bd**), the propargylation (**3be**), the benzylation (**3bh**, **3bi**, and **3bl**), and the alkylation with α-bromoacetophenone (**3bu**) occurred smoothly to deliver the products in moderate to high yields with excellent enantioselectivity (73–99%, 96–99% ee). It should be noted that Bz-protection instead of Fmoc-protection was employed in **3bu** for easy separation. Moreover, the alkylation with alkyl iodides proceeded with satisfactory results (**3bx**, **3by**, and **3bb′**, 80–82%, 86–90% ee). However, both 80 °C and Cs_2_CO_3_ were required for higher yields. Several asymmetric alkylations of **1c** and **1d** worked well, too (**3ca**, **3ce**, **3da**, **3de**, and **3dg**, 72–88%, 84–96% ee). A consecutive asymmetric alkylation in one pot was demonstrated to be feasible, as shown in Fig. 5. In the presence of the copper(I) catalyst and K_3_PO_4_, the initial allylation with allyl bromide and the following alkylation with methyl α-bromoacetate afforded α-amino acid derivative **4** in 62% yield with 95% ee after the cleavage of imino group and protection of the generated free amine. The stereochemistry of these products was assigned by their structural analogy to **3aw**.

**Fig. 6:**
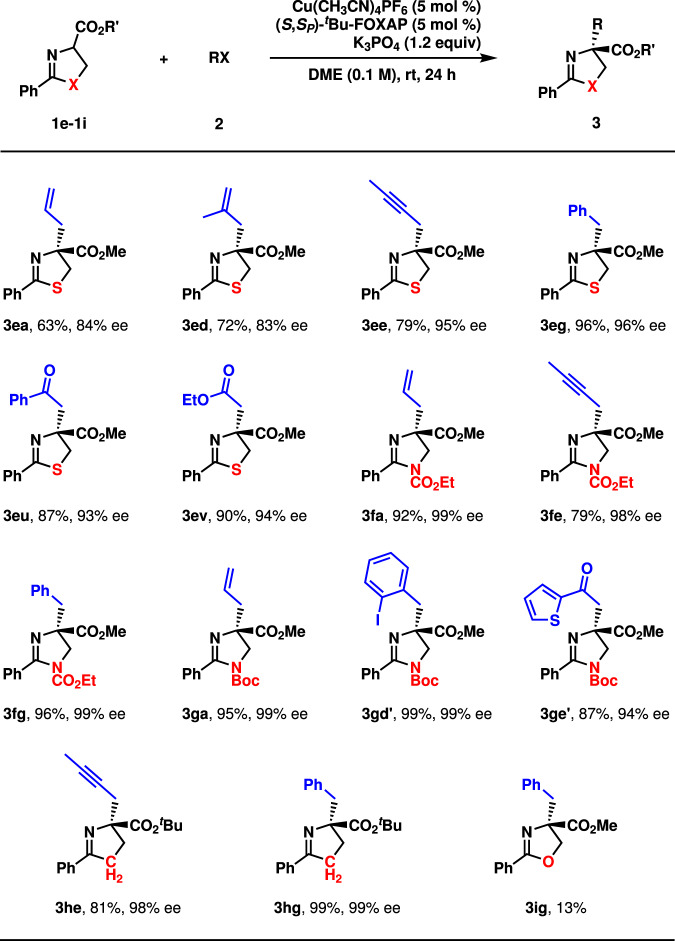
Substrate scope III. Reaction conditions: **1** (0.3 mmol), **2** (0.2 mmol). Isolated yields were reported. Ee was determined by chiral stationary-phase HPLC analysis.

The asymmetric alkylation was further extended from linear α-imino-esters (**1a**-**1d**) to cyclic ones (**1e**-**1i**), as shown in Fig. 6. With **1e** as the precursor of an enolate, the allylation, the propargylation, the benzylation, and the alkylations with α-bromoacetophenone and α-bromoacetate proceeded in high to excellent enantioselectivity (**3ea**, **3ed**, **3ee**, **3eg**, **3eu**, and **3ev**, 63–96%, 83–96% ee). However, the yields were moderate in some cases (**3ea**, **3ed**, and **3ee**). Moreover, the alkylations of cyclic imino-esters (**1f**-**1h**) occurred in moderate to high yields with excellent enantioselectivity (**3fa**, **3fe**, **3fg**, **3ga**, **3gd′**, **3ge′**, **3he**, and **3** **hg**, 79–99%, 94–99% ee). However, the cyclic imino-ester (**1i**) derived from serine was not a competent substrate, as **3ig** was obtained in only 13% yield. The stereochemistry of **3ge′** was determined by X-ray crystallography. The absolute configurations of other products were tentatively assigned.

### Mechanism studies

**Fig. 7:**
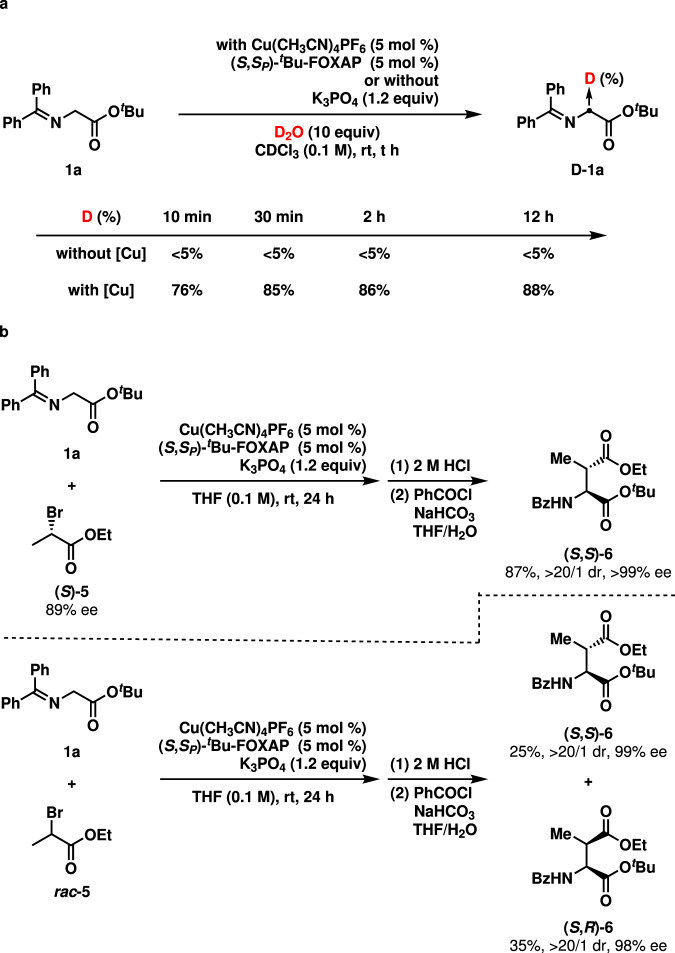
Control experiments. **a** D/H exchange experiments (^1^H NMR yields reported). **b** Asymmetric alkylation with a secondary alkyl halide (isolated yields reported).

Subsequently, some control experiments were performed to probe the reaction mechanism. First, H/D exchange experiments were set up (Fig. 7a). Treating **1a** with 1.2 equiv. K_3_PO_4_ and 10.0 equiv. D_2_O in 1 mL CDCl_3_ afforded **D-1a** in less than 5% yield. However, in the presence of 5 mol % complex of Cu(CH_3_CN)_4_PF_6_ and (*S*,S_P_)-^*t*^Bu-FOXAP, the deuterated ratios of the methylene group reached 76% in 10 min and 85% in 30 min, indicating that the complexation of the Cu(CH_3_CN)_4_PF_6_-(*S*,S_P_)-^*t*^Bu-FOXAP to **1a** significantly activates the methylene group (for details, see Supplementary Information). Second, the Cu(CH_3_CN)_4_PF_6_-(*S*,S_P_)-^*t*^Bu-FOXAP-**1a** complex was clearly observed by both ^1^H NMR and ^31^P spectra (for details, see Supplementary Information). Third, the alkylation of **1a** with a secondary alkyl halide **5** was studied (Fig. 7b). With (*S*)-**5**, the reaction afforded the product (*S*,*S*)-**6** in 10/1 dr, which was isolated in 87% yield with >20/1 dr and >99% ee after hydrolysis and protection. It should be noted that racemization of (*S*)-**5** did not occur under the present alkylation conditions (for details, see Supplementary Information). Furthermore, the alkylation with *rac*-**5** furnished (*S*,*S*)-**6** in 25% yield with 99% ee and (*S*,*R*)-**6** in 35% yield with 98% ee, indicating that the alkylation with (*R*)-**5** might be slightly faster than the reaction with (*S*)-**5**. It is noteworthy that optically active **6** was a useful intermediate in the total synthesis of natural products^64–68^. Evidently, our reaction provides a straightforward synthetic method to two diastereoisomeric chiral 1,4-dicarbonyl compounds from a racemic electrophile^69^. Based on the above experimental facts, a reasonable mechanism, including activation of α-imino-ester by the copper(I)-(*S*,S_P_)-^*t*^Bu-FOXAP complex through further complexation, generation of the copper(I)-enolate complex through deprotonation, S_N_2 substitution with the chiral copper(I)-enolate, and regeneration of the copper(I)-catalyst is proposed in Supplementary Information. Moreover, a rationale for the asymmetric induction is provided in Supplementary Information. For a kinetic resolution of racemic secondary alkyl halides under asymmetric phase-transfer catalysis, see ref. ^69^.

### Synthetic application

**Fig. 8:**
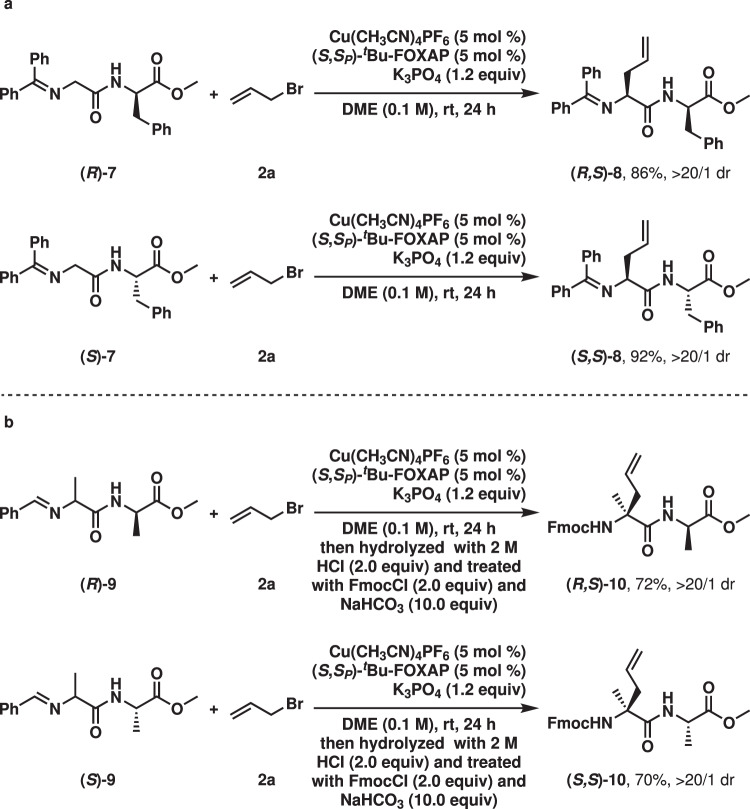
Catalytic asymmetric alkylation of chiral dipeptides. **a** Construction of a trisubstituted stereogenic center through catalytic asymmetric allylation. **b** Construction of a tetrasubstituted stereogenic center through catalytic asymmetric allylation.

Considering the mildly basic nature of the catalytic system, the methodology was extended to the catalytic asymmetric alkylation of chiral dipeptides (Fig. 8a, b). Both (*R*)-**7** and (*S*)-**7** underwent the desired reaction to provide the corresponding products ((*R*,*S*)-**8** and (*S*,*S*)-**8**) in high yields with excellent diastereoselectivity. Moreover, the asymmetric alkylation was successfully applied to the construction of tetrasubstituted stereogenic carbon centers in dipeptides, which gave (*R*,*S*)-**10** and (*S*,*S*)-**10** excellent results after hydrolysis and protection. These experiments indicated that the stereochemistry in the catalytic asymmetric alkylation was fully controlled by the copper(I)-(*S*,S_P_)-^*t*^Bu-FOXAP complex. However, in the classical asymmetric phase-transfer catalysis, the stereochemistry of both the substrate and the organocatalyst contributed to the control of the stereochemistry in the product^70,71^. Remarkably, *N*-alkylation was not observed in these reactions. Moreover, the epimerization of the pre-installed chiral carbon centers was not observed. Evidently, the mild methodology can be potentially used in the modification of more complicated chiral peptides.

## Methods

### Synthesis of compound **3aa**

A dried 25-mL Schlenk tube equipped with a magnetic stirring bar was charged with [Cu(CH_3_CN)_4_]PF_6_ (3.7 mg, 0.01 mmol, 0.05 equiv.), (*S*,S_P_)-^*t*^Bu-FOXAP (5.0 mg, 0.01 mmol, 0.05 equiv.), and K_3_PO_4_ (50.9 mg, 0.24 mmol, 1.2 equiv.) in a glove box under Ar atmosphere. Anhydrous THF (2.0 mL) was added via a syringe. The mixture was stirred at room temperature for 15 min to give an orange catalyst solution. Then α-imino-ester **1a** (88.6 mg, 0.3 mmol, 1.5 equiv.) was added. The reaction mixture was cooled to 0 °C, and allyl bromide **2a** (24.2 mg, 0.2 mmol, 1.0 equiv.) was added. The resulting reaction mixture was stirred at 0 °C for 24 h. After the volatiles were removed under reduced pressure, the crude product was purified by silica gel column chromatography (petroleum ether:EtOAc = 80:1) to give **3aa** as a yellow oil (64.3 mg, 96% yield).

### Synthesis of compound **3ba**

A dried 25-mL Schlenk tube equipped with a magnetic stirring bar was charged with [Cu(CH_3_CN)_4_]PF_6_ (3.7 mg, 0.01 mmol, 0.05 equiv.), (*S*,S_P_)-^*t*^Bu-FOXAP (5.0 mg, 0.01 mmol, 0.05 equiv.), and K_3_PO_4_ (50.9 mg, 0.24 mmol, 1.2 equiv.) in a glove box under Ar atmosphere. Anhydrous DME (2.0 mL) was added via a syringe. The mixture was stirred at room temperature for 15 min to give an orange catalyst solution. Then α-imino-ester **1b** (57.4 mg, 0.3 mmol, 1.5 equiv.) and allyl bromide **2a** (24.2 mg, 0.2 mmol, 1.0 equiv.) were added sequentially. The resulting reaction mixture was stirred at room temperature for 24 h. The reaction mixture was extracted with ethyl ether (3 × 2 mL) and saturated NaCl solution (15 mL) to remove the base. The combined organic layers were evaporated in vacuo. The residue was dissolved in THF (2 mL) and hydrolyzed with 2 M HCl (0.2 mL, 0.4 mmol, 2.0 equiv.) for 15 min (thin-layer chromatography (TLC) monitoring). Then H_2_O (1 mL), NaHCO_3_ (168 mg, 2.0 mmol, 10 equiv.), and FmocCl (103.5 mg, 0.4 mmol, 2.0 equiv.) were added sequentially. The resulting reaction mixture was stirred at room temperature for 30 min and extracted with ethyl ether (3 × 2 mL) and saturated NaCl solution (15 mL). The combined organic layers were evaporated in vacuo, and the crude product was purified by silica gel column chromatography (petroleum ether:EtOAc = 30:1) to give **3ba** as a colorless oil (67.3 mg, 92% yield).

## Supplementary information

Supplementary Information

## Data Availability

All data are available from the authors upon request. Supplementary information and chemical compound information are available along with the online version of the paper. The X-ray crystallographic coordinates for structures reported in this study have been deposited at the Cambridge Crystallographic Data Centre (CCDC) under deposition number 2201046. These data can be obtained free of charge from The Cambridge Crystallographic Data Centre via www.ccdc.cam.ac.uk/data_request/cif.
