# Supplementary material for: Copper(I)-catalyzed asymmetric alkylation of α-imino-esters
Source: Nat Commun. 2023 Apr 17;14:2187. doi: 10.1038/s41467-023-37967-y (PMC10110621; doi:10.1038/s41467-023-37967-y)
Supplement: Supplementary file 1 — Supplementary Information [file 41467_2023_37967_MOESM1_ESM.pdf]

Supplementary Information

for

Copper(I)-Catalyzed Asymmetric Alkylation of α-Imino-Esters

Zong-Ci Liu[1], Zi-Qing Wang[1], Xuan Zhang[1], and Liang Yin[1,*]

[1]CAS Key Laboratory of Synthetic Chemistry of Natural Substances, Center for Excellence in Molecular Synthesis, Shanghai Institute of Organic Chemistry, University of Chinese Academy of Sciences, Chinese Academy of Sciences, 345 Lingling Road, Shanghai 200032, China

**e-mail: liangyin@sioc.ac.cn*

Table of Contents

1 Supplementary Methods 2

1.1 General Information 2

1.2 Preparation of α-Imino-Esters and Alkyl Halides 3

1.3 Copper(I)-Catalyzed Asymmetric Alkylation of α-Imino-Esters 7

2 Supplementary Discussion 66

2.1 Gram-Scale Reaction, Double Asymmetric Alkylation in One Pot, and Catalytic Asymmetric Alkylation of Chiral Dipeptides 66

2.2 Determination of the Absolute Configurations of the Products 72

2.3 Control Experiments and Proposed Mechanism 74

3 Supplementary References 82

4 Supplementary Figures 84

4.1 ^{1}H, ^{13}C{^{1}H}, and ^{19}F NMR Spectra of New Compounds 84

1 Supplementary Methods

1.1 General Information

All reagents were obtained commercially unless otherwise noted. Dry tetrahydrofuran (THF), dry 1,2-Dimethoxyethane (DME), and dry *N*,*N*-Dimethylformamide (DMF) were commercially available and used direct. K_3PO_4 was commercially available and dried *in vacuo* with a heat gun. The commercially available reagents were purchased from Adamas, Aladdin, Accela, Bidepharm and Energy Chemical with ≥95% purities. All of the heat sources were oil baths. Nuclear Magnetic Resonance (NMR) spectra were acquired on a Varian 400, Bruker 400 or Bruker 500 instrument. For ^1H NMR, chemical shifts were reported in δ ppm referenced to an internal $SiMe_4$ standard. For ^{19}F NMR, $CFCl_3$ was used as the reference with chemical shift at 0 ppm. For $^{13}C\{^1H\}$ NMR, chemical shifts were reported in the scale relative to NMR solvent ($CDCl_3$: δ 77.0 ppm) as an internal reference. Multiplicities are reported using the following abbreviations: s = singlet, d = doublet, t = triplet, q = quartet, m = multiplet, br = broad signal. High-resolution mass spectra (ESI) were measured on Agilent Technologies 6230 TOF LC/MS. Infrared (IR) spectra were recorded on a Thermo Scientific Nicolet iS5 FT-IR. Optical rotation was measured using an ϕ 3 mm cell with 1.0 dm path length on a JASCO P-1030 polarimeter. High Performance Liquid Chromatography (HPLC) analysis was conducted on a Shimadzu HPLC system equipped with Daicel chiral-stationary-phase columns. X-ray single crystal diffraction was conducted on Bruker D8 Venture.

1.2 Preparation of α-Imino-Esters and Alkyl Halides

α-Imino-esters **1a**[1], **1b**[2], **1c**[2], **1d**[2], **1e**[3, 4], **1g**[5] , **1h**[6] , and **1i**[7] are known compounds.
Alkyl halides **2a**-**2a'**, **2c'**-**2e'**, and ***rac*-5** were obtained commercially. **2b'**[8] and **(*S*)-5**[9,10] are prepared by known routes.

1-ethyl 4-methyl 2-phenyl-4,5-dihydro-1*H*-imidazole-1,4-dicarboxylate (**1f**)

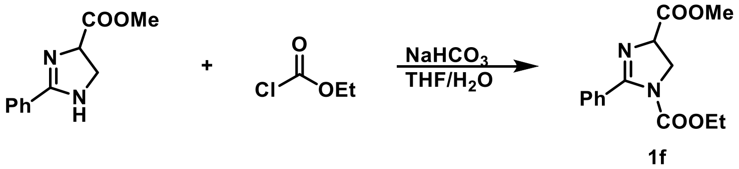

To a 50 mL round-bottom flask were added methyl 2-phenyl-4,5-dihydro-1*H*-imidazole-4-carboxylate (204 mg, 1.0 mmol, 1.0 equiv), $NaHCO_3$ (840 mg, 10 mmol, 10 equiv), THF (10 mL), and H_2O (10 mL). After the reaction mixture was cooled to 0 °C, ethyl carbonochloridate (217 mg, 2.0 mmol, 2.0 equiv) was added. After stirring at 0 °C for 1.5 h (thin-layer chromatography (TLC) monitoring), the reaction mixture was extracted with DCM (3×20 mL) and water (30 mL). The combined organic layers were dried over Na_2SO_4 and filtered. The solvents were removed *in vacuo*. The crude product was then purified by flash chromatography (silica gel, petroleum ether (PE):EtOAc (EA) = 5:1) to provide pure 1-ethyl 4-methyl 2-phenyl-4,5-dihydro-1*H*-imidazole-1,4-dicarboxylate (**1f**, 165.2 mg, 60% yield) as a colorless oil.

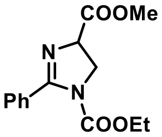

^{1}H NMR (400 MHz, $CDCl_3$) δ 7.58 – 7.52 (m, 2H), 7.47 – 7.41 (m, 1H), 7.40 – 7.34 (m, 2H), 4.84 (dd, *J* = 10.7, 8.0 Hz, 1H), 4.32 (dd, *J* = 11.1, 8.0 Hz, 1H), 4.21 (t, *J* = 10.9 Hz, 1H), 4.10 (q, *J* = 7.1 Hz, 2H), 3.82 (s, 3H), 1.13 (t, *J* = 7.1 Hz, 3H) ppm.
^{13}C{^{1}H} NMR (126 MHz, $CDCl_3$) δ 171.2, 161.4, 151.4, 130.8, 130.3, 128.6, 127.5, 66.4, 62.4, 52.8, 49.5, 14.1 ppm.
HRMS (ESI-TOF) m/z: $[M + H]^+$ Calcd for $C_{14}H_{17}N_2O_4^+$ 277.1183; Found 277.1183.
IR (film): ν_{max} (cm^{-1}) 2982, 2955, 1736, 1624, 1599, 1447 1399, 1377, 1327, 1290, 1204, 1175, 1132, 1034, 1018, 765, 697.

ethyl (*S*)-2-bromopropanoate (**(*S*)-5**)

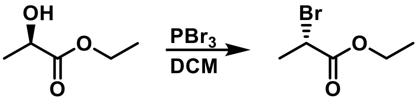

To a 50 mL round-bottom flask were added ethyl (*R*)-2-hydroxypropanoate (1.77 g, 15 mmol, 1.0 equiv) and DCM (20 mL). After the reaction mixture was cooled to 0 °C, PBr_3 (1.35 g, 5.0 mmol, 0.33 equiv) was added. After stirring at 0 °C for 4 h (TLC monitoring), the reaction mixture was neutralized by aqueous $NaHCO_3$ and extracted with DCM (20 mL). The organic layer was dried over Na_2SO_4 and filtered. The solvents were removed *in vacuo*. The crude product was then purified by flash chromatography (PE:EA = 100:1) to provide ethyl (*S*)-2-bromopropanoate (**(*S*)-5**, 274.3 mg, 10% yield) as a colorless oil. It is indicated that the configuration of the product was *S* by comparing the optical rotation of the product with **(*R*)-5** ($[\alpha]_D^{20}$ = +23.9 (*c* = 0.43, $CHCl_3$)) in literature[9].

^{1}H NMR (400 MHz, $CDCl_3$) δ 4.36 (q, *J* = 6.9 Hz, 1H), 4.28 – 4.19 (m, 2H), 1.83 (d, *J* = 6.9 Hz, 3H), 1.30 (t, *J* = 7.1 Hz, 3H) ppm.

The spectroscopic data match the literature.[10]

Optical rotation: $[\alpha]_D^{25}$ = -29.94 (*c* = 1.050, $CHCl_3$, 89% ee).

HPLC: DAICEL CHIRALPAK ID, hexane/*i*-PrOH = 100/0, flow rate: 1.0 mL/min, λ = 220 nm, t_R(major) = 11.7 min, t_R(minor) = 15.0 min, 89% ee.

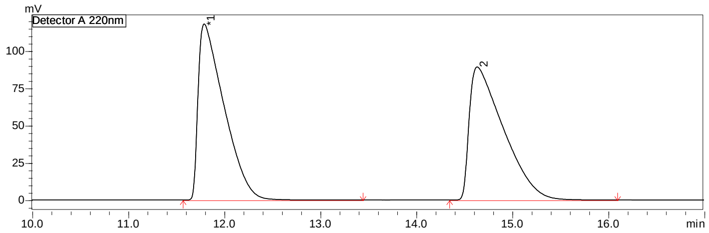

Peak#	Ret. Time	Area%
1	11.794	50.039
2	14.637	49.961

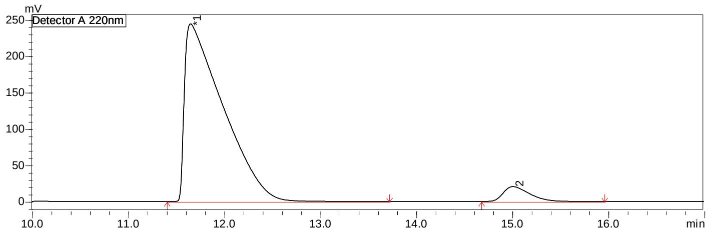

Peak#	Ret. Time	Area%
1	11.652	94.635
2	15.011	5.365

methyl (2-((diphenylmethylene)amino)acetyl)-*D*-phenylalaninate (**(*R*)-7**)

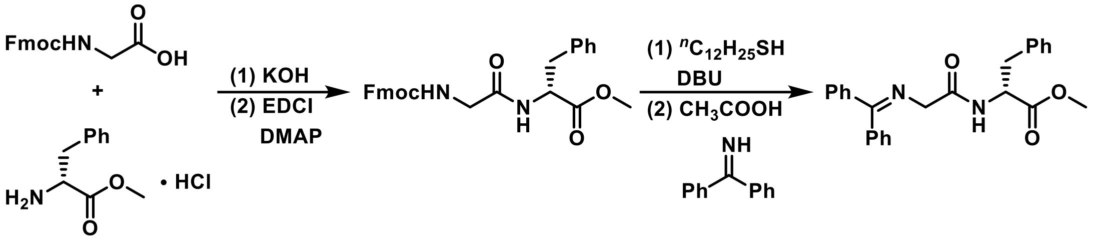

To a 25 mL round-bottom flask were added methyl *D*-phenylalaninate hydrochloride (431.4 mg, 2.0 mmol, 1.0 equiv), H_2O (0.5 mL), DCM (5 mL), and KOH (135 mg, 2.4 mmol, 1.2 equiv) sequentially. The reaction mixture was stirred till all solids were dissolved, dried over anhydrous Na_2SO_4, and filtered. The residue was washed by 5 mL DCM. To a 25 mL round-bottom flask were added the combined organic layers, (((9*H*-fluoren-9-yl)methoxy)carbonyl)glycine (595 mg, 2 mmol, 1.0 equiv), DMAP (48.9 mg, 0.4 mmol, 0.2 equiv), and EDC·HCl (460 mg, 2.4 mmol, 1.2 equiv) sequentially. Then the reaction mixture was stirred at room temperature for 12 hours (TLC monitoring). Then $^{n}C_{12}H_{25}SH$ (607 mg, 3.0 mmol, 1.5 equiv) and DBU (761 mg, 5 mmol, 2.5 equiv) were added sequentially. After the reaction mixture was stirred for 15 minutes (TLC monitoring), diphenylmethanimine (544 mg, 3.0 mmol, 1.5 equiv) and CH_3COOH (480 mg, 8.0 mmol, 4.0 equiv) were added sequentially. Then the reaction mixture was stirred for 12 hours (TLC monitoring). 1 mL NEt_3 was added to neutralize CH_3COOH. After the volatiles were removed under reduced pressure, the crude product was purified by silica gel column chromatography (PE:EA = 10:1~5:1) to give **(*R*)-7** as a white solid (437.5 mg, 55% yield, m.p. 97-101 ℃).

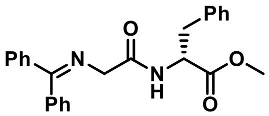

^{1}H NMR (400 MHz, $CDCl_3$) δ 7.92 (d, *J* = 8.1 Hz, 1H), 7.56 – 7.50 (m, 2H), 7.50 – 7.40 (m, 4H), 7.39 – 7.24 (m, 5H), 7.23 – 7.15 (m, 2H), 7.15 – 7.05 (m, 2H), 4.99 (dt, *J* = 8.2, 5.9 Hz, 1H), 4.03 – 3.89 (m, 2H), 3.75 (s, 3H), 3.29 – 3.14 (m, 2H) ppm.

$^{13}C\{^{1}H\}$ NMR (126 MHz, $CDCl_3$) δ 171.8, 170.5, 170.2, 138.6, 135.9, 135.8, 130.6, 129.3, 129.0, 128.8, 128.7, 128.5, 128.1, 127.2, 127.1, 56.4, 52.6, 52.3, 38.0 ppm.

HRMS (ESI-TOF) m/z: $[M + H]^+$ Calcd for $C_{25}H_{25}N_2O_3^+$ 401.1860; Found 401.1860.

IR (film): ν_{max} (cm^{-1}) 3375, 3028, 2952, 2926, 1745, 1682, 1507, 1446, 1362, 1289, 1213, 1178, 1077, 1029, 784, 745, 699.

Optical rotation: $[\alpha]_D^{25}$ = -16.50 (*c* = 0.860, $CHCl_3$).

(*S*)-7 was prepared by the same procedure.

methyl (2-(benzylideneamino)propanoyl)-*L*-alaninate (**(*S*)-9**)

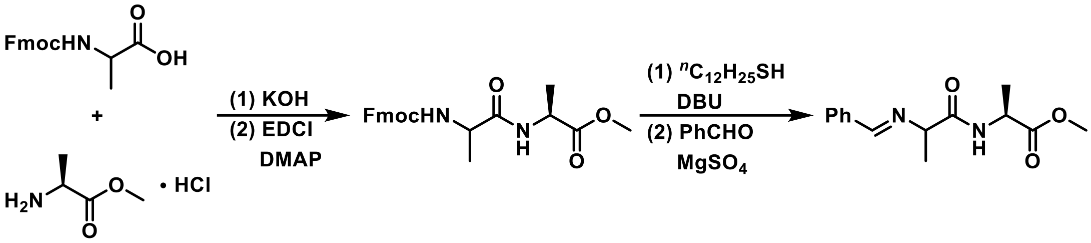

To a 25 mL round-bottom flask were added methyl *L*-alaninate hydrochloride (140 mg, 1.0 mmol, 1.0 equiv), H_2O (0.25 mL), DCM (2.5 mL), and KOH (67.3 mg, 1.2 mmol, 1.2 equiv) sequentially. The reaction mixture was stirred till all solids were dissolved, dried over anhydrous Na_2SO_4, and filtered. The residue was washed by 2.5 mL DCM. To a 25 mL round-bottom flask were added the combined organic layers, (((9*H*-fluoren-9-yl)methoxy)carbonyl)alanine (311 mg, 1.0 mmol, 1.0 equiv), DMAP (24.4 mg, 0.2 mmol, 0.2 equiv), and EDC·HCl (230 mg, 1.2 mmol, 1.2 equiv) sequentially. Then the reaction mixture was stirred at room temperature for 12 hours (TLC monitoring). Then, $^{n}C_{12}H_{25}SH$ (304 mg, 1.5 mmol, 1.5 equiv) and DBU (381 mg, 2.5 mmol, 2.5 equiv) were added sequentially. After the reaction mixture was stirred for 15 minutes (TLC monitoring), PhCHO (159 mg, 1.5 mmol, 1.5 equiv) and $MgSO_4$ (181 mg, 1.5 mmol, 1.5 equiv) were added sequentially. Then the reaction mixture was stirred for 48 hours (TLC monitoring). 0.5 mL NEt_3 was added to prevent the product from hydrolysis. After the volatiles were removed under reduced pressure, the crude product was purified by NEt_3 basified silica gel column chromatography (PE:EA:NEt_3 = 75:25:1) to give **(*S*)-9** as a colorless oil (118.7 mg, 45% yield).

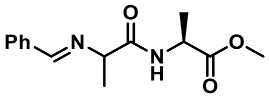

^{1}H NMR (400 MHz, $CDCl_3$) δ 8.27 (d, *J* = 1.6 Hz, 1H), 7.85 – 7.75 (m, 2H), 7.59 – 7.39 (m, 4H), 4.69 – 4.58 (m, 1H), 4.03 – 3.95 (m, 1H), 3.80 – 3.71 (m, 3H), 1.51 – 1.40 (m, 6H) ppm.
$^{13}C\{^{1}H\}$ NMR (126 MHz, $CDCl_3$) δ 173.3, 173.3, 173.2, 173.2, 162.0, 161.7, 135.5, 131.3, 131.3, 128.7, 128.3, 128.3, 67.9, 67.8, 52.4, 52.3, 47.6, 21.2, 21.1, 18.4, 18.3 ppm.
HRMS (ESI-TOF) m/z: $[M + H]^+$ Calcd for $C_{14}H_{19}N_2O_3^+$ 263.1390; Found 263.1390.
IR (film): ν_{max} (cm^{-1}) 3380, 2980, 2931, 2872, 1744, 1678, 1509, 1451, 1375, 1345, 1292, 1218, 1160, 1126, 1063, 757, 694.
Optical rotation: $[\alpha]_D^{25}$ = +49.61 (*c* = 1.790, $CHCl_3$).

(*R*)-9 was prepared by the same procedure.

1.3 Copper(I)-Catalyzed Asymmetric Alkylation of α-Imino-Esters

General Procedure

Procedure A:

A dried 25 mL Schlenk tube equipped with a magnetic stirring bar was charged with $[Cu(CH_3CN)_4]PF_6$ (3.7 mg, 0.01 mmol, 0.05 equiv), (*S*,*S*$_P$)-^{t}Bu-FOXAP (5.0 mg, 0.01 mmol, 0.05 equiv), and K_3PO_4 (50.9 mg, 0.24 mmol, 1.2 equiv) in a glove box under Ar atmosphere. Anhydrous THF (2.0 mL) was added via a syringe. The mixture was stirred at room temperature for 15 minutes to give an orange catalyst solution. Then α-imino-ester **1** (0.3 mmol, 1.5 equiv) was added. The reaction mixture was cooled to 0 °C and alkyl bromide **2** (0.2 mmol, 1.0 equiv) was added. The resulting reaction mixture was stirred at 0 °C for 24 hours. After the volatiles were removed under reduced pressure, the crude product was purified by silica gel column chromatography to give the desired product.

Procedure B:

Following procedure A, however using alkyl iodide instead of alkyl bromide, and using DMF as solvent instead of THF.

Procedure C:

Following procedure B, however performing the reaction at room temperature.

Procedure D:

Following procedure C, however using 1,6-diiodohexane (**2c'**, 67.7 mg, 0.2 mmol, 1.0 equiv) as alkyl iodide, and increasing the amounts of **1a** (177.2 mg, 0.6 mmol, 3.0 equiv) and K_3PO_4 (101.9 mg, 0.48 mmol, 2.4 equiv).

Procedure E:

Following procedure A, however using DME as the solvent instead of THF and performing the reaction at room temperature. A different work-up procedure was involved. After being stirred at room temperature for 24 hours, the reaction mixture was extracted with ethyl ether (3x2 mL) and saturated NaCl solution (15 mL) to remove the base. The combined organic layers were evaporated *in vacuo*. The residue was dissolved in THF (2 mL) and hydrolyzed with 2 M HCl (0.2 mL, 0.4 mmol, 2.0 equiv) for 15 min (TLC monitoring). Then H_2O (1 mL), $NaHCO_3$ (168 mg, 2.0 mmol, 10 equiv), and FmocCl (103.5 mg, 0.4 mmol, 2.0 equiv) were added sequentially. The resulting reaction mixture was stirred at room temperature for 30 minutes and extracted with ethyl ether (3x2 mL) and saturated NaCl solution (15 mL). The combined organic layers were evaporated *in vacuo* and the crude product was purified by silica gel column chromatography to give the desired product.

Procedure F:

Following procedure E, however using PhCOCl (56.2 mg, 0.4 mmol, 2.0 equiv) instead of FmocCl.

Procedure G:

Following procedure E, however using alkyl iodide instead of alkyl bromide, using Cs_2CO_3 (78.2 mg, 0.24 mmol, 1.2 equiv) instead of K_3PO_4, and performing the reaction at 80 °C for 12 hours.

Procedure H:

Following procedure A, however using DME as the solvent instead of THF and performing the reaction at room temperature.

tert-butyl (*S*)-2-((diphenylmethylene)amino)pent-4-enoate (**3aa**)

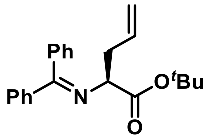

Procedure A. Purification by flash column chromatography (PE:EA = 80:1) afforded the product as a yellow oil (64.3mg, 96% yield).

^{1}H NMR (400 MHz, $CDCl_3$) δ 7.69 – 7.61 (m, 2H), 7.48 – 7.41 (m, 3H), 7.41 – 7.36 (m, 1H), 7.35 – 7.29 (m, 2H), 7.21 – 7.13 (m, 2H), 5.72 (ddt, *J* = 17.2, 10.1, 7.1 Hz, 1H), 5.18 – 4.94 (m, 2H), 4.01 (dd, *J* = 7.6, 5.3 Hz, 1H), 2.75 – 2.55 (m, 2H), 1.44 (s, 9H) ppm.

The spectroscopic data match the literature.[11]

HPLC: DAICEL CHIRALPAK IE, hexane/*i*-PrOH = 99/1, flow rate: 1.0 mL/min, λ = 254 nm, t_R(minor) = 8.3 min, t_R(major) = 9.3 min, 96% ee.

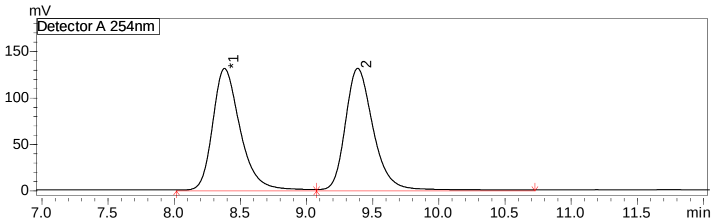

Peak#	Ret. Time	Area%
1	8.387	49.774
2	9.393	50.226

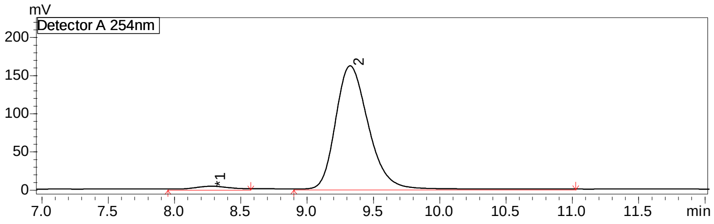

Peak#	Ret. Time	Area%
1	8.285	2.147
2	9.331	97.853

tert-butyl (*S*)-2-((diphenylmethylene)amino)hex-4-enoate (**3ab**)

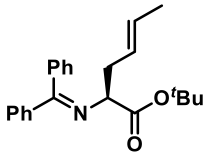

Procedure A, while **2b** had a *Z*/*E* = 1:5 ratio. Purification by flash column chromatography (PE:EA = 80:1) afforded the product as a yellow oil (62.6 mg, 90% yield, 6/1 (*E*)/(*Z*) ((*E*)/(*Z*) was determined by HPLC analysis of **3ab** after column chromatography)).

^{1}H NMR (500 MHz, $CDCl_3$) δ 7.66 – 7.62 (m, 2H), 7.46 – 7.41 (m, 3H), 7.40 – 7.35 (m, 1H), 7.35 – 7.29 (m, 2H), 7.20 – 7.12 (m, 2H), 5.55 – 5.43 (m, 1H), 5.37 – 5.26 (m, 1H), 4.00 – 3.92 (m, 1H), 2.66 – 2.45 (m, 2H), 1.64 – 1.59 (m, 3H), 1.47 – 1.39 (m, 9H) ppm.

The spectroscopic data match the literature.[12]

HPLC: Connecting DAICEL CHIRALPAK IG-3 and OX-3 in sequence, hexane/*i*-PrOH = 49/1, flow rate: 0.5 mL/min, λ = 254 nm, t_R(major) = 21.0 min, t_R(minor) = 22.9 min, 96% ee, 6/1 (*E*)/(*Z*).

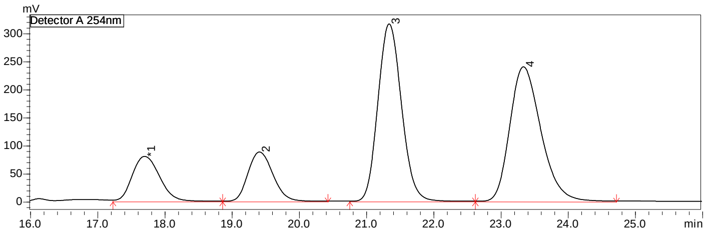

Peak#	Ret. Time	Area%
1	17.709	11.308
2	19.417	11.302
3	21.347	38.681
4	23.345	38.709

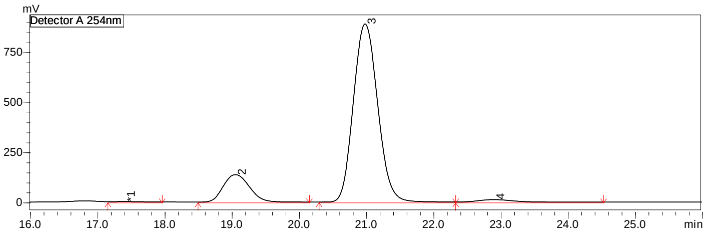

Peak#	Ret. Time	Area%
1	17.410	0.288
2	19.059	13.838
3	20.989	84.303
4	22.905	1.571

tert-butyl (*S*,*E*)-2-((diphenylmethylene)amino)-5-phenylpent-4-enoate (**3ac**)

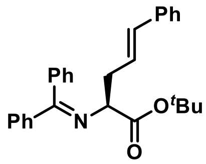

Procedure A. Purification by flash column chromatography (PE:EA = 80:1) afforded the product as a yellow oil (79.9mg, 97% yield).

^{1}H NMR (500 MHz, $CDCl_3$) δ 7.70 – 7.64 (m, 2H), 7.46 – 7.38 (m, 4H), 7.37 – 7.33 (m, 2H), 7.33 – 7.29 (m, 4H), 7.25 – 7.19 (m, 1H), 7.18 – 7.13 (m, 2H), 6.43 (d, *J* = 15.8 Hz, 1H), 6.11 (dt, *J* = 15.8, 7.4 Hz, 1H), 4.11 (dd, *J* = 7.9, 5.1 Hz, 1H), 2.88 – 2.74 (m, 2H), 1.47 (s, 9H) ppm.

The spectroscopic data match the literature.[13]

HPLC: DAICEL CHIRALPAK IG3, hexane/*i*-PrOH = 74/1, flow rate: 0.75 mL/min, λ = 254 nm, t_R(minor) = 9.6 min, t_R(major) = 10.4 min, 98% ee.

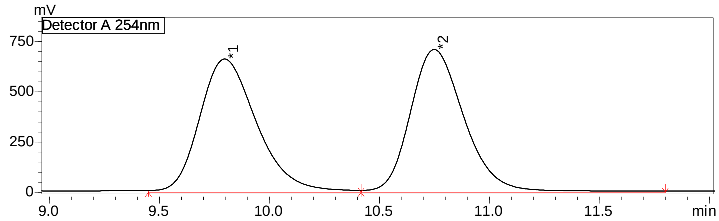

Peak#	Ret. Time	Area%
1	9.801	49.864
2	10.754	50.136

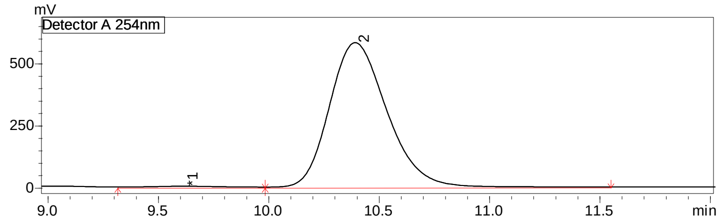

Peak#	Ret. Time	Area%
1	9.620	0.851
2	10.396	99.149

tert-butyl (*S*)-2-((diphenylmethylene)amino)-4-methylpent-4-enoate (**3ad**)

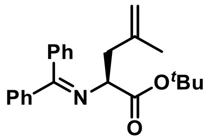

Procedure A. Purification by flash column chromatography (PE:EA = 80:1) afforded the product as a white solid (60.6mg, 87% yield, m.p. 60-63).

^{1}H NMR (400 MHz, $CDCl_3$) δ 7.67 – 7.60 (m, 2H), 7.46 – 7.41 (m, 3H), 7.40 – 7.35 (m, 1H), 7.35 – 7.28 (m, 2H), 7.21 – 7.14 (m, 2H), 4.80 – 4.67 (m, 2H), 4.08 (dd, *J* = 8.3, 5.2 Hz, 1H), 2.69 – 2.51 (m, 2H), 1.52 (s, 3H), 1.45 (s, 9H) ppm.

The spectroscopic data match the literature.[11]

HPLC: DAICEL CHIRALPAK IE, hexane/*i*-PrOH = 99/1, flow rate: 1.0 mL/min, λ = 254 nm, t_R(minor) = 8.9 min, t_R(major) = 10.3 min, 94% ee.

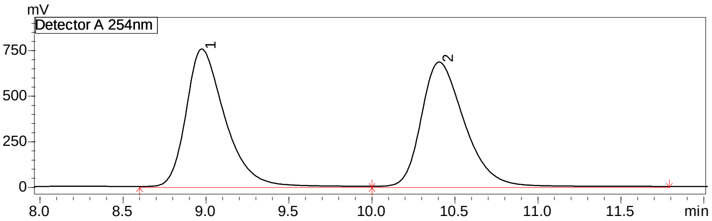

Peak#	Ret. Time	Area%
1	8.980	49.878
2	10.411	50.122

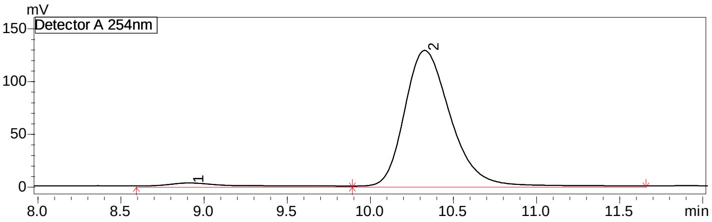

Peak#	Ret. Time	Area%
1	8.919	2.767
2	10.333	97.233

tert-butyl (*S*)-2-((diphenylmethylene)amino)hex-4-ynoate (**3ae**)

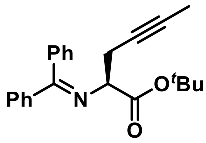

Procedure A. Purification by flash column chromatography (PE:EA = 60:1) afforded the product as a yellow oil (63.0mg, 91% yield).

^{1}H NMR (400 MHz, $CDCl_3$) δ 7.69 – 7.61 (m, 2H), 7.48 – 7.42 (m, 3H), 7.42 – 7.36 (m, 1H), 7.36 – 7.30 (m, 2H), 7.26 – 7.20 (m, 2H), 4.12 (dd, *J* = 8.1, 5.2 Hz, 1H), 2.84 – 2.71 (m, 1H), 2.71 – 2.59 (m, 1H), 1.73 (t, *J* = 2.5 Hz, 3H), 1.45 (s, 9H) ppm.

The spectroscopic data match the literature.[14]

HPLC: DAICEL CHIRALPAK IF-3, hexane/*i*-PrOH = 495/5, flow rate: 0.5 mL/min, λ = 254 nm, t_R(minor) = 15.9 min, t_R(major) = 21.1 min, 96% ee.

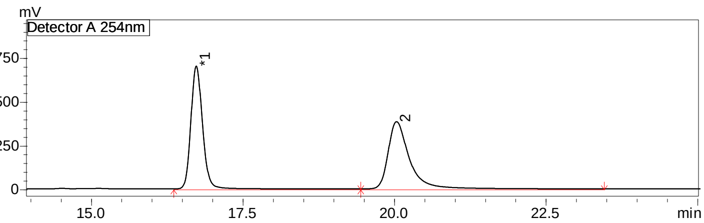

Peak#	Ret. Time	Area%
1	16.745	50.085
2	20.049	49.915

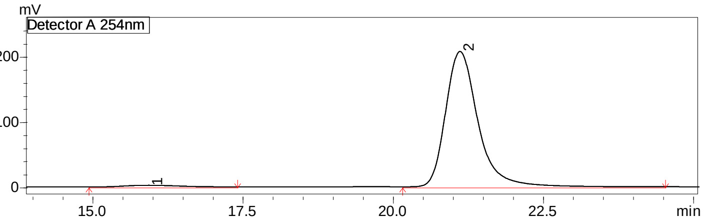

Peak#	Ret. Time	Area%
1	15.944	1.913
2	21.128	98.087

tert-butyl (*S*)-2-((diphenylmethylene)amino)-5-(trimethylsilyl)pent-4-ynoate(**3af**)

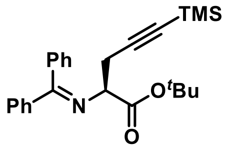

Procedure A. Purification by flash column chromatography (PE:EA = 80:1) afforded the product as a white solid (62.0 mg, 76% yield, m.p. 97-98℃).

^{1}H NMR (400 MHz, $CDCl_3$) δ 7.68 – 7.58 (m, 2H), 7.49 – 7.24 (m, 8H), 4.18 (dd, J = 8.1, 5.4 Hz, 1H), 2.92 – 2.76 (m, 2H), 1.44 (s, 9H), 0.10 (s, 9H) ppm.

The spectroscopic data match the literature.[15]

HPLC: DAICEL CHIRALPAK IG-3, hexane/*i*-PrOH = 497/3, flow rate: 0.5 mL/min, λ = 254 nm, t_R(minor) = 10.0 min, t_R(major) = 11.6 min, 91% ee.

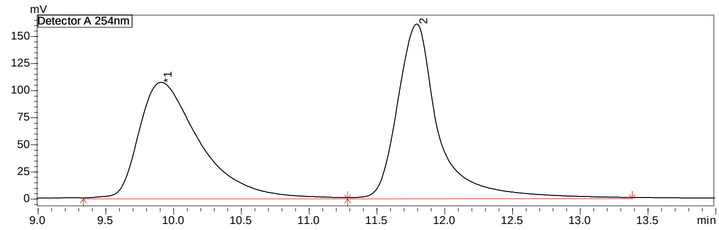

Peak#	Ret. Time	Area%
1	9.914	49.692
2	11.799	50.308

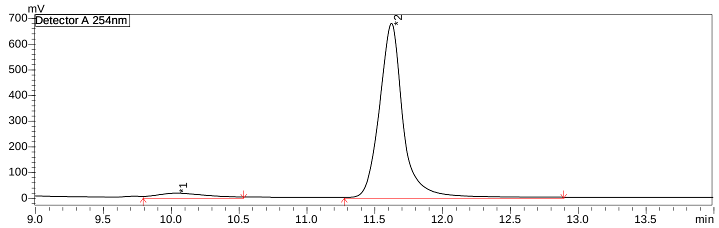

Peak#	Ret. Time	Area%
1	10.044	4.724
2	11.628	95.276

tert-butyl (*S*)-2-((diphenylmethylene)amino)-3-phenylpropanoate (**3ag**)

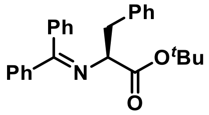

Procedure A. Purification by flash column chromatography (PE:EA = 60:1) afforded the product as a yellow oil (71.8 mg, 93% yield).

^{1}H NMR (400 MHz, $CDCl_3$) δ 7.64 – 7.53 (m, 2H), 7.42 – 7.26 (m, 6H), 7.22 – 7.12 (m, 3H), 7.10 – 7.02 (m, 2H), 6.60 (d, *J* = 6.1 Hz, 2H), 4.10 (dd, *J* = 9.2, 4.3 Hz, 1H), 3.23 (dd, *J* = 13.3, 4.3 Hz, 1H), 3.16 (dd, *J* = 13.3, 9.2 Hz, 1H), 1.44 (s, 9H) ppm.

The spectroscopic data match the literature.[11]

HPLC: DAICEL CHIRALPAK IE, hexane/*i*-PrOH = 98/2, flow rate: 1.0 mL/min, λ = 254 nm, t_R(minor) = 7.9 min, t_R(major) = 8.8 min, 97% ee.

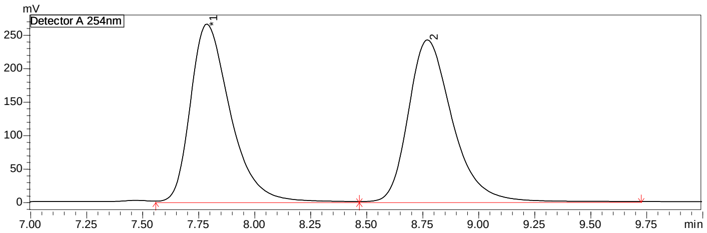

Peak#	Ret. Time	Area%
1	7.789	49.814
2	8.774	50.186

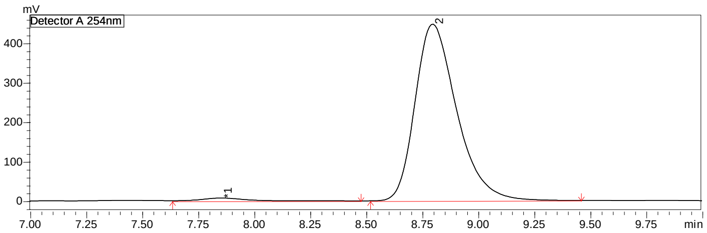

Peak#	Ret. Time	Area%
1	7.854	1.552
2	8.797	98.448

tert-butyl (*S*)-3-(4-(*tert*-butyl)phenyl)-2-((diphenylmethylene)amino)propanoate (**3ah**)

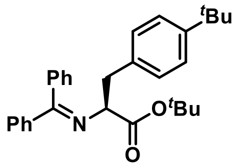

Procedure A. Purification by flash column chromatography (PE:EA = 70:1) afforded the product as a yellow oil (85.3 mg, 97% yield).

^{1}H NMR (400 MHz, $CDCl_3$) δ 7.62 – 7.55 (m, 2H), 7.40 – 7.34 (m, 1H), 7.34 – 7.28 (m, 3H), 7.25 – 7.16 (m, 4H), 6.97 (d, *J* = 8.2 Hz, 2H), 6.53 (d, *J* = 5.8 Hz, 2H), 4.07 (dd, *J* = 9.2, 4.1 Hz, 1H), 3.19 (dd, *J* = 13.4, 4.0 Hz, 1H), 3.11 (dd, *J* = 13.3, 9.3 Hz, 1H), 1.43 (s, 9H), 1.29 (s, 9H) ppm.

The spectroscopic data match the literature.[16]

HPLC: DAICEL CHIRALPAK IE, hexane/*i*-PrOH = 99/1, flow rate: 1.0 mL/min, λ = 254 nm, t_R(minor) = 9.0 min, t_R(major) = 10.3 min, 97% ee.

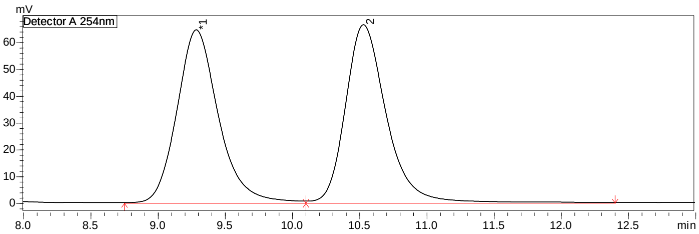

Peak#	Ret. Time	Area%
1	9.290	49.756
2	10.533	50.244

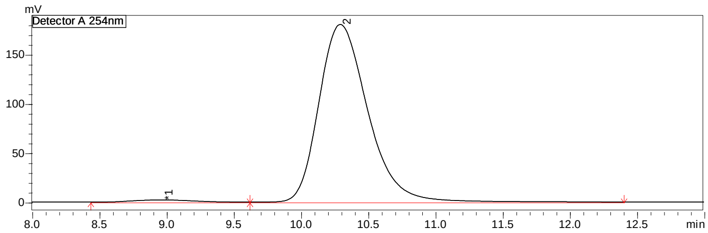

Peak#	Ret. Time	Area%
1	8.968	1.376
2	10.294	98.624

tert-butyl (*S*)-3-(2-chlorophenyl)-2-((diphenylmethylene)amino)propanoate (**3ai**)

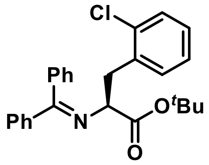

Procedure A. Purification by flash column chromatography (PE:EA = 60:1) afforded the product as a colorless oil (80.5 mg, 96% yield).

^{1}H NMR (400 MHz, $CDCl_3$) δ 7.63 – 7.54 (m, 2H), 7.40 – 7.27 (m, 6H), 7.25 – 7.18 (m, 2H), 7.15 – 7.04 (m, 2H), 6.61 (d, *J* = 6.2 Hz, 2H), 4.30 (dd, *J* = 9.5, 4.2 Hz, 1H), 3.46 (dd, *J* = 13.4, 4.1 Hz, 1H), 3.20 (dd, *J* = 13.3, 9.5 Hz, 1H), 1.44 (s, 9H) ppm.

The spectroscopic data match the literature.[16]

HPLC: DAICEL CHIRALPAK IE, hexane/*i*-PrOH = 99/1, flow rate: 1.0 mL/min, λ = 254 nm, t_R(minor) = 10.3 min, t_R(major) = 12.2 min, 99% ee.

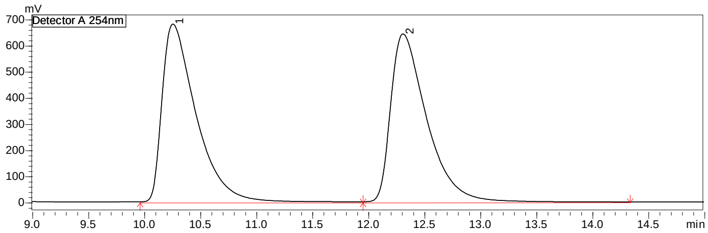

Peak#	Ret. Time	Area%
1	10.259	49.954
2	12.313	50.046

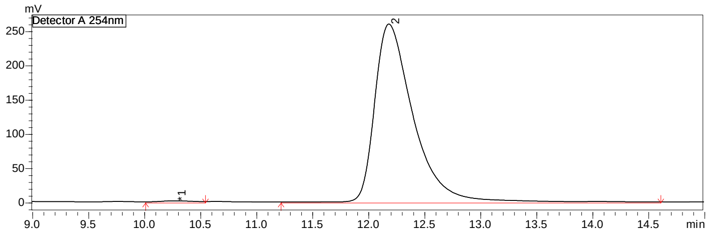

Peak#	Ret. Time	Area%
1	10.279	0.644
2	12.186	99.356

tert-butyl (*S*)-2-((diphenylmethylene)amino)-3-(2-nitrophenyl)propanoate (**3aj**)

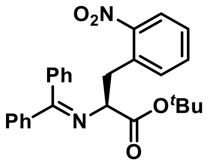

Procedure A. Purification by flash column chromatography (PE:acetone = 100:1) afforded the product as a green oil (84.1 mg, 98% yield).

^{1}H NMR (400 MHz, $CDCl_3$) δ 7.86 (dd, J = 8.1, 1.3 Hz, 1H), 7.61 – 7.53 (m, 2H), 7.46 – 7.23 (m, 9H), 6.60 (d, J = 7.0 Hz, 2H), 4.32 (dd, J = 9.3, 4.0 Hz, 1H), 3.69 (dd, J = 13.3, 4.0 Hz, 1H), 3.40 (dd, J = 13.3, 9.3 Hz, 1H), 1.43 (s, 9H) ppm.

The spectroscopic data match the literature.[17]

HPLC: DAICEL CHIRALPAK IE, hexane/*i*-PrOH = 95/5, flow rate: 1.0 mL/min, λ = 254 nm, t_R(minor) = 10.8 min, t_R(major) = 13.8 min, 97% ee.

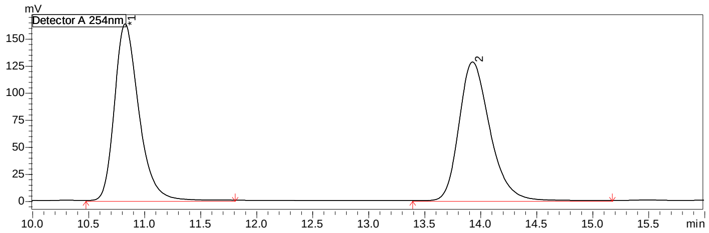

Peak#	Ret. Time	Area%
1	10.831	50.188
2	13.934	49.812

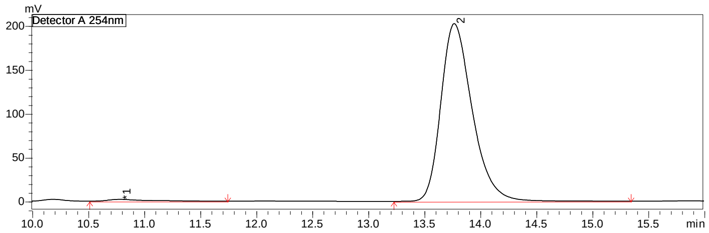

Peak#	Ret. Time	Area%
1	10.786	1.372
2	13.770	98.628

tert-butyl (*S*)-2-((diphenylmethylene)amino)-3-(3-nitrophenyl)propanoate (**3ak**)

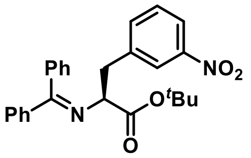

Procedure A. Purification by flash column chromatography (PE:acetone = 200:1) afforded the product as a yellow oil (82.0 mg, 95% yield).

^{1}H NMR (400 MHz, $CDCl_3$) δ 8.03 (dd, *J* = 8.2, 2.2 Hz, 1H), 7.95 (s, 1H), 7.57 (d, *J* = 7.4 Hz, 2H), 7.46 (d, *J* = 7.5 Hz, 1H), 7.42 – 7.26 (m, 7H), 6.72 (d, *J* = 7.2 Hz, 2H), 4.20 (dd, *J* = 7.6, 5.6 Hz, 1H), 3.38 – 3.23 (m, 2H), 1.45 (s, 9H) ppm.

^{13}C{^{1}H} NMR (101 MHz, $CDCl_3$) δ 171.0, 170.0, 148.0, 140.4, 139.0, 136.1, 135.9, 130.3, 128.8, 128.6, 128.6, 128.2, 127.9, 127.3, 124.5, 121.3, 81.6, 66.9, 39.0, 27.9 ppm.

The spectroscopic data match the literature.[18]

HRMS (ESI-TOF) m/z: $[M + H]^+$ Calcd for $C_{26}H_{27}N_2O_4^+$ 431.1965; Found 431.1965.

IR (film): ν_{max} (cm^{-1}) 3061, 2976, 2928, 2869, 1732, 1622, 1576, 1529, 1479, 1446, 1393, 1351, 1316, 1285, 1259, 1151, 1086, 1029, 845, 804, 781, 736, 696.

Optical rotation: $[\alpha]_D^{25}$ = -215.45 (*c* = 0.880, $CHCl_3$, 94% ee).

HPLC: DAICEL CHIRALPAK IE, hexane/*i*-PrOH = 95/5, flow rate: 1.0 mL/min, λ = 254 nm, t_R(minor) = 10.9 min, t_R(major) = 12.0 min, 94% ee.

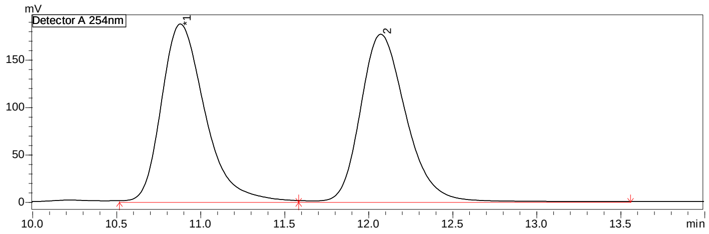

Peak#	Ret. Time	Area%
1	10.883	50.483
2	12.076	49.517

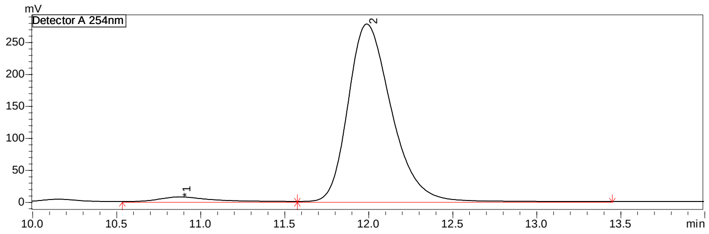

Peak#	Ret. Time	Area%
1	10.881	2.880
2	11.993	97.120

tert-butyl (*S*)-3-(3,5-dimethylphenyl)-2-((diphenylmethylene)amino)propanoate (**3al**)

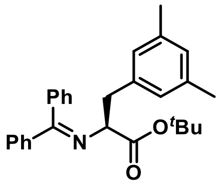

Procedure A. Purification by flash column chromatography (PE:EA = 60:1) afforded the product as a yellow oil (74.2 mg, 90% yield).

^{1}H NMR (400 MHz, $CDCl_3$) δ 7.57 – 7.52 (m, 2H), 7.38 – 7.27 (m, 6H), 6.78 (s, 1H), 6.69 – 6.56 (m, 4H), 4.09 (dd, *J* = 9.4, 4.3 Hz, 1H), 3.15 (dd, *J* = 13.3, 4.3 Hz, 1H), 3.07 (dd, *J* = 13.2, 9.4 Hz, 1H), 2.18 (s, 6H), 1.45 (s, 9H) ppm.

The spectroscopic data match the literature.[19]

HPLC: DAICEL CHIRALPAK IE, hexane/*i*-PrOH = 99/1, flow rate: 1.0 mL/min, λ = 254 nm, t_R(minor) = 11.6 min, t_R(major) = 14.1 min, 94% ee.

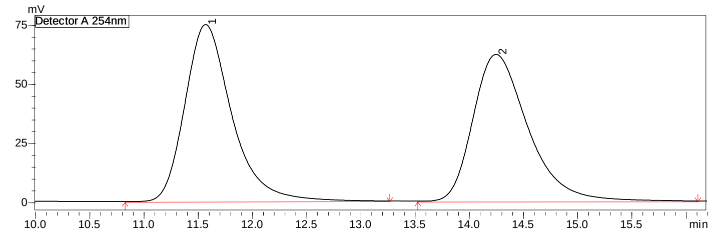

Peak#	Ret. Time	Area%
1	11.574	50.661
2	14.254	49.339

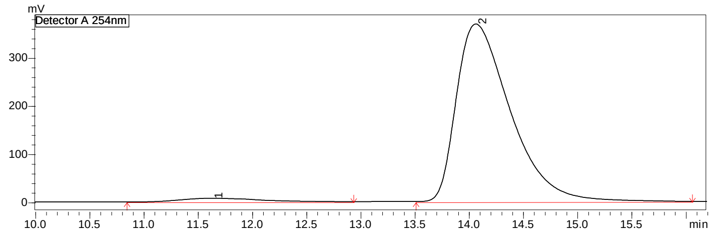

Peak#	Ret. Time	Area%
1	11.634	3.022
2	14.067	96.978

tert-butyl (*S*)-3-(3,5-difluorophenyl)-2-((diphenylmethylene)amino)propanoate (**3am**)

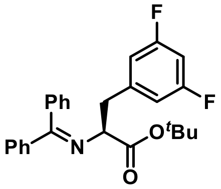

Procedure A. Purification by flash column chromatography (PE:EA = 70:1) afforded the product as a colorless oil (82.0 mg, 97% yield).

^{1}H NMR (400 MHz, $CDCl_3$) δ 7.57 (d, *J* = 7.9 Hz, 2H), 7.44 – 7.29 (m, 6H), 6.78 (d, *J* = 6.5 Hz, 2H), 6.67 – 6.56 (m, 3H), 4.12 (dd, *J* = 8.9, 4.4 Hz, 1H), 3.20 (dd, *J* = 13.5, 4.4 Hz, 1H), 3.13 (dd, *J* = 13.4, 9.0 Hz, 1H), 1.44 (s, 9H) ppm.

The spectroscopic data match the literature.[20]

HPLC: DAICEL CHIRALPAK IE, hexane/*i*-PrOH = 49/1, flow rate: 0.5 mL/min, λ = 254 nm, t_R(minor) = 11.5 min, t_R(major) = 12.4 min, 94% ee.

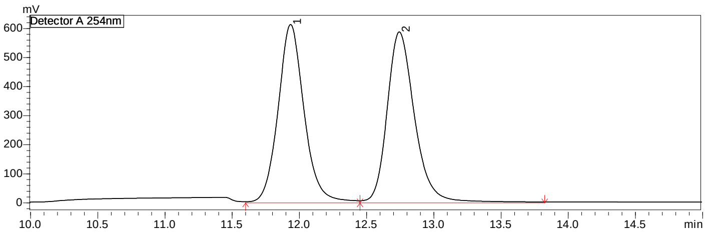

Peak#	Ret. Time	Area%
1	11.940	49.817
2	12.749	50.183

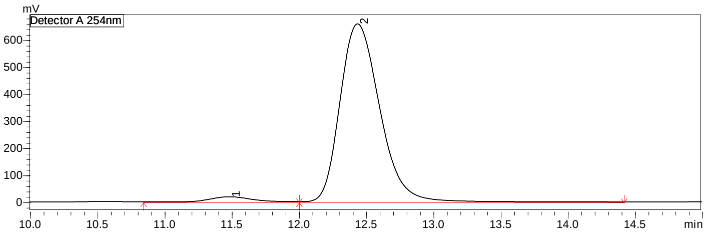

Peak#	Ret. Time	Area%
1	11.484	2.932
2	12.438	97.068

tert-butyl (*S*)-3-(3,5-bis(trifluoromethyl)phenyl)-2-((diphenylmethylene)amino)propanoate (**3an**)

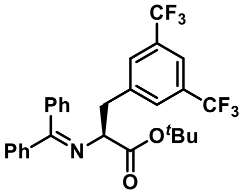

Procedure A. Purification by flash column chromatography (PE:EA = 100:1) afforded the product as a colorless oil (97.0 mg, 93% yield).

^{1}H NMR (400 MHz, $CDCl_3$) δ 7.69 (s, 1H), 7.57 (s, 2H), 7.55 – 7.51 (m, 2H), 7.41 – 7.35 (m, 2H), 7.35 – 7.27 (m, 4H), 6.68 (d, *J* = 7.0 Hz, 2H), 4.15 (dd, *J* = 8.3, 5.0 Hz, 1H), 3.39 – 3.25 (m, 2H), 1.45 (s, 9H) ppm.

The spectroscopic data match the literature.[21]

HPLC: DAICEL CHIRALPAK IC-3, hexane/*i*-PrOH = 495/5, flow rate: 0.5 mL/min, λ = 254 nm, t_R(minor) = 8.0 min, t_R(major) = 8.4 min, 93% ee.

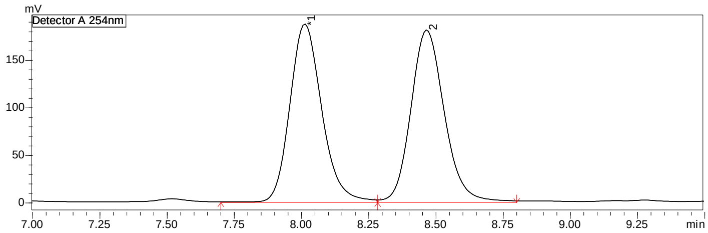

Peak#	Ret. Time	Area%
1	8.015	49.678
2	8.468	50.322

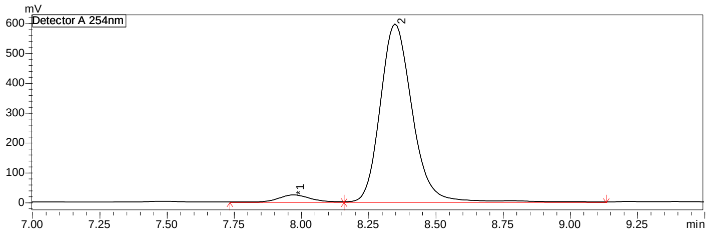

Peak#	Ret. Time	Area%
1	7.973	3.643
2	8.350	96.357

tert-butyl (*S*)-3-(2-bromo-5-fluorophenyl)-2-((diphenylmethylene)amino)propanoate (**3ao**)

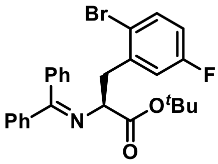

Procedure A. Purification by flash column chromatography (PE:EA = 60:1) afforded the product as a yellow oil (93.8 mg, 97% yield).

^{1}H NMR (400 MHz, $CDCl_3$) δ 7.59 (d, *J* = 8.1 Hz, 2H), 7.43 – 7.34 (m, 3H), 7.34 – 7.28 (m, 4H), 6.95 (dd, *J* = 9.3, 2.9 Hz, 1H), 6.82 – 6.64 (m, 3H), 4.33 (dd, *J* = 9.3, 4.3 Hz, 1H), 3.41 (dd, *J* = 13.5, 4.2 Hz, 1H), 3.22 (dd, *J* = 13.3, 9.5 Hz, 1H), 1.45 (s, 9H) ppm.

The spectroscopic data match the literature.[12]

HPLC: DAICEL CHIRALPAK IE, hexane/*i*-PrOH = 99/1, flow rate: 1.0 mL/min, λ = 254 nm, t_R(minor) = 9.2 min, t_R(major) = 10.9 min, 98% ee.

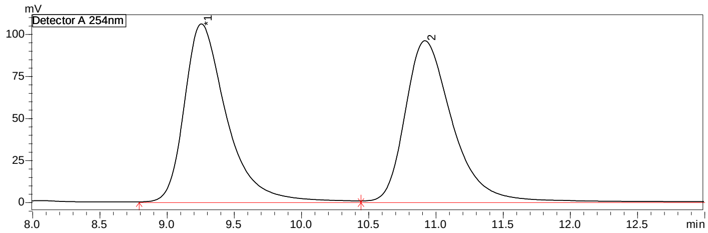

Peak#	Ret. Time	Area%
1	9.260	49.642
2	10.924	50.358

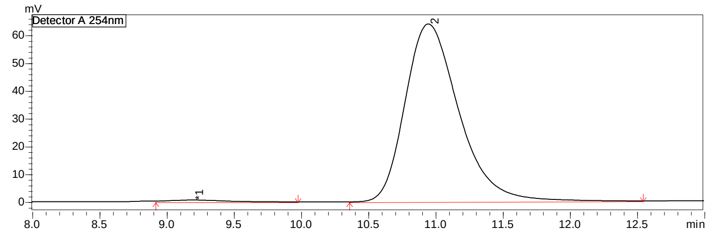

Peak#	Ret. Time	Area%
1	9.195	0.972
2	10.949	99.028

tert-butyl (*S*)-3-(3-chloro-2-fluorophenyl)-2-((diphenylmethylene)amino)propanoate (**3ap**)

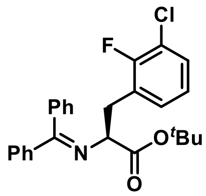

Procedure A. Purification by flash column chromatography (PE:EA = 60:1) afforded the product as a colorless oil (79.8mg, 91% yield).

^{1}H NMR (400 MHz, $CDCl_3$) δ 7.60 – 7.53 (m, 2H), 7.41 – 7.25 (m, 6H), 7.23 – 7.17 (m, 1H), 7.07 (t, *J* = 6.7 Hz, 1H), 6.90 (t, *J* = 7.8 Hz, 1H), 6.67 (d, *J* = 6.1 Hz, 2H), 4.21 (dd, *J* = 9.4, 4.2 Hz, 1H), 3.35 (dd, *J* = 13.5, 3.6 Hz, 1H), 3.16 (dd, *J* = 13.0, 9.9 Hz, 1H), 1.45 (s, 9H) ppm.

$^{13}C\{^1H\}$ NMR (101 MHz, $CDCl_3$) δ 170.9, 170.2, 156.6 (d, *J* = 248.3 Hz) 139.2, 135.9, 130.6 (d, *J* = 4.2 Hz) 130.2, 128.7, 128.6, 128.5, 128.2, 127.9, 127.4, 127.1 (d, *J* = 15.4 Hz) 124.0 (d, *J* = 4.6 Hz) 120.7 (d, *J* = 18.3 Hz) 81.4, 65.5, 32.9, 28.0 ppm.

^{19}F NMR (376 MHz, $CDCl_3$) δ -119.27 ppm.

HRMS (ESI-TOF) m/z: $[M + H]^+$ Calcd for $C_{26}H_{26}ClFNO_2^+$ 438.1631; Found 438.1631.

IR (film): ν_{max} (cm^{-1}) 2975, 2928, 2855, 1735, 1624, 1577, 1459, 1392, 1368, 1262, 1229, 1152, 1073, 1028, 896, 845, 817, 778, 696, 641.

Optical rotation: $[\alpha]_D^{25}$ = -248.31 (*c* = 0.910, $CHCl_3$, 98% ee).

HPLC: DAICEL CHIRALPAK IE, hexane/*i*-PrOH = 99/1, flow rate: 1.0 mL/min, λ = 254 nm, t_R(minor) = 9.2 min, t_R(major) = 10.6 min, 98% ee.

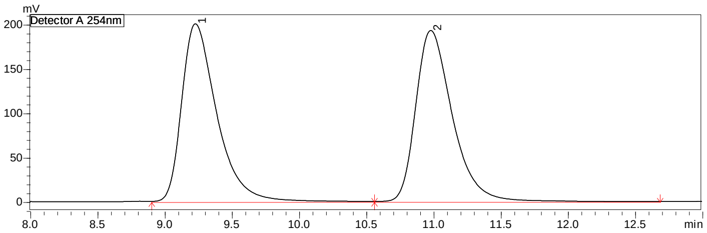

Peak#	Ret. Time	Area%
1	9.231	49.908
2	10.982	50.092

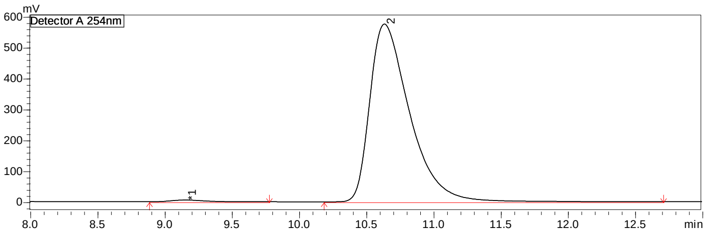

Peak#	Ret. Time	Area%
1	9.156	0.855
2	10.637	99.145

tert-butyl (*S*)-3-(2,6-difluorophenyl)-2-((diphenylmethylene)amino)propanoate (**3aq**)

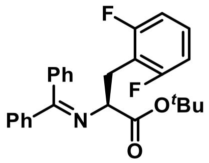

Procedure A. Purification by flash column chromatography (PE:EA = 80:1) afforded the product as a yellow oil (79.0 mg, 94% yield).

^{1}H NMR (400 MHz, $CDCl_3$) δ 7.57 (d, *J* = 7.3 Hz, 2H), 7.40 – 7.25 (m, 6H), 7.15 – 7.04 (m, 1H), 6.82 – 6.65 (m, 4H), 4.25 (dd, *J* = 9.2, 4.6 Hz, 1H), 3.33 (dd, *J* = 13.6, 9.3 Hz, 1H), 3.25 (dd, *J* = 13.5, 4.4 Hz, 1H), 1.45 (s, 9H) ppm.

^{13}C{^{1}H} NMR (101 MHz, $CDCl_3$) δ 170.8, 170.3, 161.8 (dd, *J* = 247.9, 8.7 Hz) 139.4, 136.0, 130.1, 128.8, 128.4, 128.1, 127.8, 127.8 (t, *J* = 10.1 Hz) 127.6, 114.1 (t, *J* = 20.0 Hz), 110.8 (dd, *J* = 18.8, 6.2 Hz), 81.3, 65.1, 27.9, 26.1 ppm.

^{19}F NMR (376 MHz, $CDCl_3$) δ -113.52 ppm.

HRMS (ESI-TOF) m/z: $[M + H]^+$ Calcd for $C_{26}H_{26}F_2NO_2^+$ 422.1926; Found 422.1926.

IR (film): ν_{max} (cm^{-1}) 2976, 2928, 2856, 1733, 1625, 1593, 1470, 1446, 1369, 1266, 1152, 1067, 1030, 845, 782, 702.

Optical rotation: $[\alpha]_D^{25}$ = -206.71 (*c* = 0.990, $CHCl_3$, 97% ee).

HPLC: DAICEL CHIRALPAK IE, hexane/*i*-PrOH = 98/2, flow rate: 1.0 mL/min, λ = 254 nm, t_R(minor) = 7.9 min, t_R(major) = 10.3 min, 97% ee.

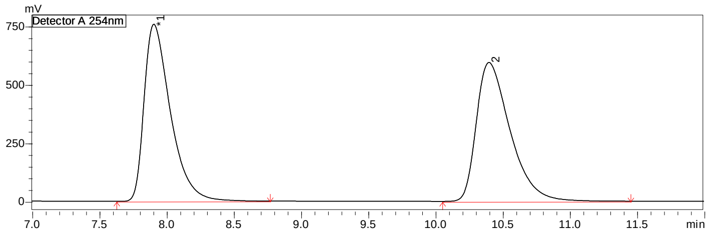

Peak#	Ret. Time	Area%
1	7.907	49.865
2	10.400	50.135

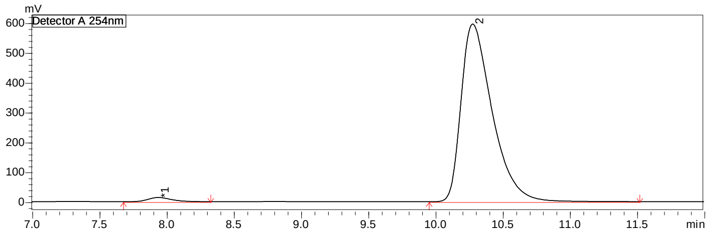

Peak#	Ret. Time	Area%
1	7.940	1.722
2	10.279	98.278

tert-butyl (*S*)-3-(2-bromo-6-chlorophenyl)-2-((diphenylmethylene)amino)propanoate (**3ar**)

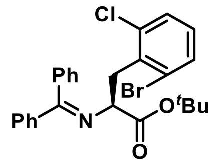

Procedure A. Purification by flash column chromatography (PE:EA = 80:1) afforded the product as a yellow oil (91.8 mg, 92% yield).

^{1}H NMR (400 MHz, $CDCl_3$) δ 7.70 – 7.60 (m, 2H), 7.38 – 7.31 (m, 3H), 7.31 – 7.23 (m, 4H), 7.20 (d, *J* = 8.0 Hz, 1H), 6.94 (t, *J* = 8.0 Hz, 1H), 6.62 (d, *J* = 4.0 Hz, 2H), 4.53 (dd, *J* = 10.2, 3.6 Hz, 1H), 3.75 (dd, *J* = 13.5, 10.3 Hz, 1H), 3.47 (dd, *J* = 13.6, 3.6 Hz, 1H), 1.46 (s, 9H) ppm.

$^{13}C\{^{1}H\}$ NMR (101 MHz, $CDCl_3$) δ 170.4, 139.2, 136.2, 136.0, 135.8, 131.4, 130.0, 129.0, 128.7, 128.3, 128.2, 128.0, 127.8, 127.6, 126.7, 81.2, 64.3, 36.8, 28.0 ppm.

HRMS (ESI-TOF) m/z: $[M + H]^+$ Calcd for $C_{26}H_{26}BrClNO_2^+$ 498.0830; Found 498.0830.

IR (film): v_{max} (cm^{-1}) 3057, 2975, 2927, 2855, 1735, 1662, 1623, 1577, 1556, 1431, 1392, 1368, 1315, 1288, 1151, 1066, 1027, 847, 775, 743, 696.

Optical rotation: $[\alpha]_D^{25}$ = -261.22 (*c* = 0.970, $CHCl_3$, 99% ee).

HPLC: DAICEL CHIRALPAK IE, hexane/*i*-PrOH = 99/1, flow rate: 1.0 mL/min, λ = 254 nm, t_R(minor) = 13.2 min, t_R(major) = 19.1 min, 99% ee.

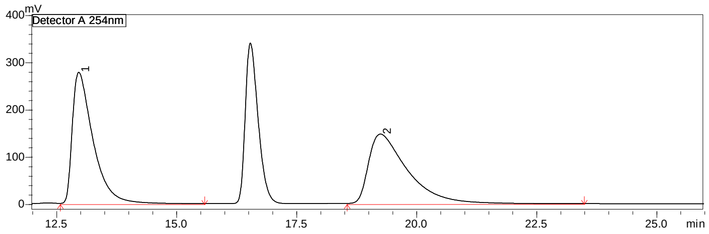

Peak#	Ret. Time	Area%
1	12.976	50.236
2	19.259	49.764

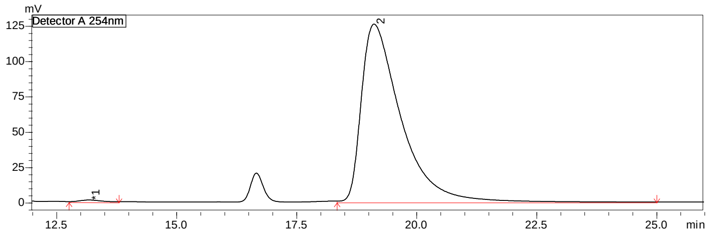

Peak#	Ret. Time	Area%
1	13.192	0.607
2	19.127	99.393

tert-butyl (*S*)-2-((diphenylmethylene)amino)-3-(pyridin-2-yl)propanoate (**3as**)

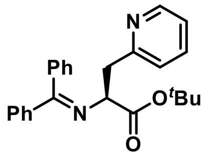

Procedure A, however using 2-(bromomethyl)pyridine hydrobromide instead of instead of alkyl bromide, and increasing the amount of K_3PO_4 (101.9 mg, 0.48 mmol, 2.4 equiv). Purification by flash column chromatography (PE:EA = 15:1) afforded the product as a colorless oil (69.4 mg, 90% yield).

^{1}H NMR (500 MHz, $CDCl_3$) δ 8.41 (d, *J* = 4.2 Hz, 1H), 7.64 – 7.45 (m, 3H), 7.40 – 7.24 (m, 6H), 7.16 (d, *J* = 7.7 Hz, 1H), 7.05 (dd, *J* = 7.5, 4.9 Hz, 1H), 6.69 (d, *J* = 5.7 Hz, 2H), 4.45 (dd, *J* = 9.4, 3.8 Hz, 1H), 3.53 – 3.28 (m, 2H), 1.44 (s, 9H) ppm.

The spectroscopic data match the literature.[22]

HPLC: DAICEL CHIRALPAK IA, hexane/*i*-PrOH = 98/2, flow rate: 1.0 mL/min, λ = 254 nm, t_R(minor) = 9.9 min, t_R(major) = 11.1 min, 99% ee.

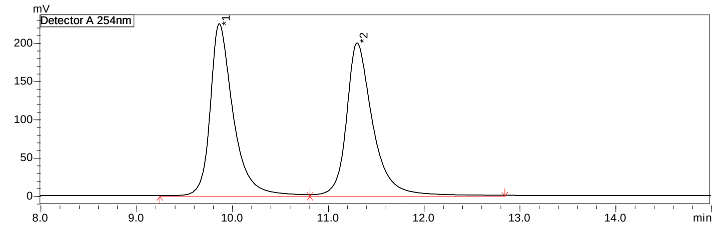

Peak#	Ret. Time	Area%
1	9.869	49.682
2	11.308	50.318

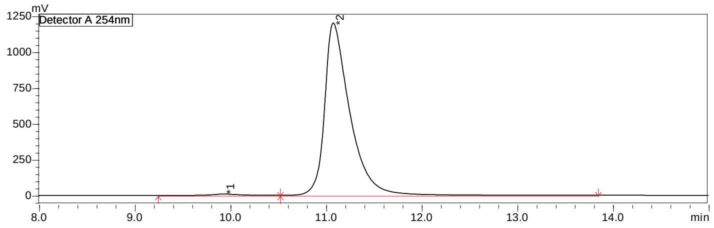

Peak#	Ret. Time	Area%
1	9.936	0.748
2	11.079	99.252

tert-butyl (*S*)-2-((diphenylmethylene)amino)-3-(thiophen-3-yl)propanoate (**3at**)

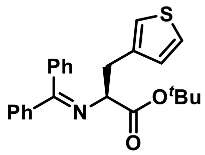

Procedure A. Purification by flash column chromatography (PE:EA = 60:1) afforded the product as a colorless oil (74.8mg, 96% yield).

^{1}H NMR (400 MHz, $CDCl_3$) δ 7.67 – 7.54 (m, 2H), 7.44 – 7.24 (m, 6H), 7.13 (dd, *J* = 4.8, 3.0 Hz, 1H), 6.90 (d, *J* = 2.9 Hz, 1H), 6.86 – 6.65 (m, 3H), 4.10 (dd, *J* = 8.4, 4.9 Hz, 1H), 3.33 – 3.13 (m, 2H), 1.43 (s, 9H) ppm.

$^{13}C\{^1H\}$ NMR (126 MHz, $CDCl_3$) δ 170.6, 170.3, 139.4, 138.5, 136.3, 130.1, 129.2, 128.7, 128.2, 128.1, 127.9, 127.5, 124.7, 122.2, 81.0, 67.2, 33.8, 27.9 ppm.

HRMS (ESI-TOF) m/z: $[M + H]^+$ Calcd for $C_{24}H_{26}NO_2S^+$ 392.1679; Found 392.1679.

IR (film): ν_{max} 3057, 2975, 2928, 1732, 1623, 1597, 1576, 1446, 1392, 1368, 1285, 1259, 1149, 1076, 1028, 847, 779, 696.

Optical rotation: $[\alpha]_D^{25}$ = -166.30 (*c* = 0.905, $CHCl_3$, 98% ee).

HPLC: DAICEL CHIRALPAK IC, hexane/*i*-PrOH = 99/1, flow rate: 1.0 mL/min, λ = 254 nm, t_R(major) = 9.1 min, t_R(minor) = 11.1 min, 98% ee.

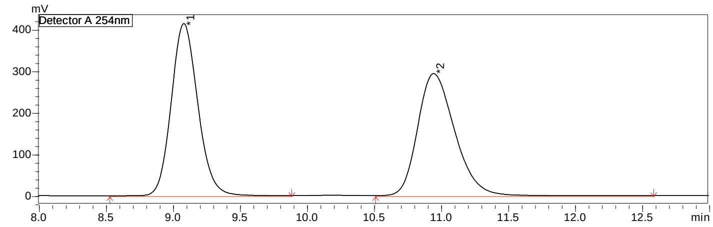

Peak#	Ret. Time	Area%
1	9.083	50.258
2	10.947	49.742

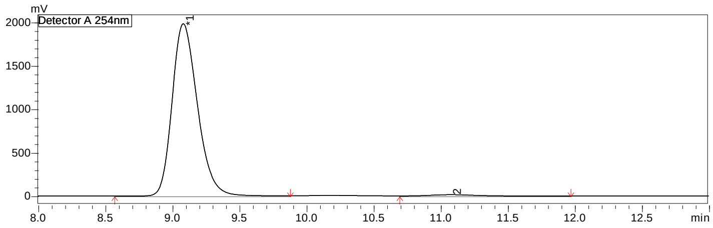

Peak#	Ret. Time	Area%
1	9.083	98.845
2	11.075	1.155

tert-butyl (*S*)-2-((diphenylmethylene)amino)-4-oxo-4-phenylbutanoate (**3au**)

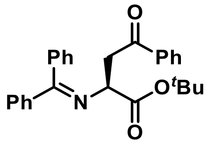

Procedure A. Purification by flash column chromatography (PE:acetone = 100:1) afforded the product as a yellow oil (77.1 mg, 93% yield).

^{1}H NMR (400 MHz, $CDCl_3$) δ 8.00 – 7.92 (m, 2H), 7.62 – 7.56 (m, 2H), 7.56 – 7.49 (m, 1H), 7.49 – 7.39 (m, 5H), 7.39 – 7.33 (m, 1H), 7.33 – 7.25 (m, 4H), 4.67 (dd, *J* = 7.1, 5.9 Hz, 1H), 3.72 (dd, *J* = 17.1, 5.9 Hz, 1H), 3.49 (dd, *J* = 17.1, 7.1 Hz, 1H), 1.44 (s, 9H) ppm.

^{13}C{^{1}H} NMR (101 MHz, $CDCl_3$) δ 197.4, 171.2, 170.4, 139.6, 136.9, 136.3, 133.0, 130.2, 128.7, 128.6, 128.4, 128.3, 128.1, 127.9, 81.3, 62.6, 42.1, 27.9 ppm.

HRMS (ESI-TOF) m/z: $[M + H]^+$ Calcd for $C_{27}H_{28}NO_3^+$ 414.2064; Found 414.2064.

IR (film): ν_{max} (cm^{-1}) 3058, 2926, 2854, 1735, 1685, 1624, 1597, 1578, 1448, 1393, 1368, 1277, 1150, 1074, 1029, 1002, 846, 781, 755, 696.

Optical rotation: $[\alpha]_D^{25}$ = -55.81 (*c* = 0.910, $CHCl_3$, 87% ee).

HPLC: DAICEL CHIRALPAK IE, hexane/*i*-PrOH = 95/5, flow rate: 1.0 mL/min, λ = 254 nm, t_R(minor) = 11.4 min, t_R(major) = 13.7 min, 87% ee.

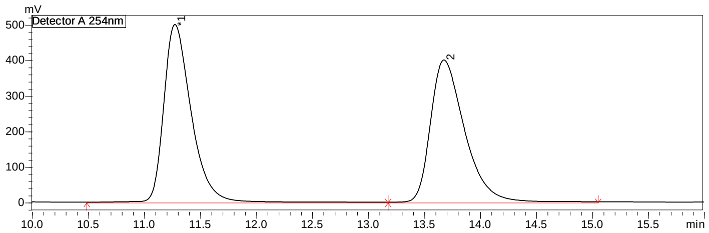

Peak#	Ret. Time	Area%
1	11.277	49.957
2	13.679	50.043

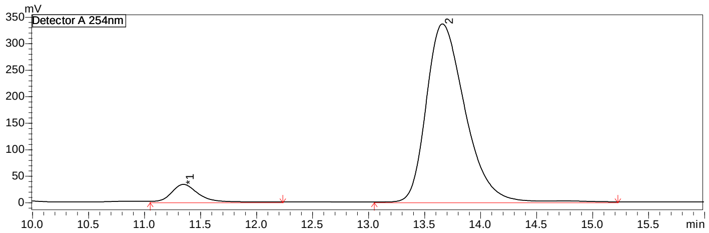

Peak#	Ret. Time	Area%
1	11.353	6.282
2	13.666	93.718

1-(*tert*-butyl) 4-ethyl (*S*)-2-((diphenylmethylene)amino)succinate (**3av**)

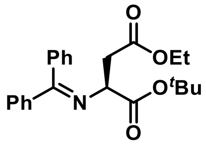

Procedure A. Purification by flash column chromatography (PE:acetone = 150:1) afforded the product as a yellow oil (67.4 mg, 88% yield).

^{1}H NMR (400 MHz, $CDCl_3$) δ 7.65 – 7.58 (m, 2H), 7.51 – 7.42 (m, 3H), 7.40 – 7.34 (m, 1H), 7.34 – 7.28 (m, 2H), 7.28 – 7.23 (m, 2H), 4.43 (dd, *J* = 7.5, 5.7 Hz, 1H), 4.17 – 4.02 (m, 2H), 3.01 (dd, *J* = 16.0, 5.6 Hz, 1H), 2.86 (dd, *J* = 16.0, 7.7 Hz, 1H), 1.44 (s, 9H), 1.21 (t, *J* = 7.1 Hz, 3H) ppm.

^{13}C{^{1}H} NMR (101 MHz, $CDCl_3$) δ 171.3, 171.0, 169.8, 139.5, 136.2, 130.2, 128.8, 128.6, 128.2, 127.9, 127.9, 81.4, 62.5, 60.4, 38.3, 27.9, 14.1 ppm.

HRMS (ESI-TOF) m/z: $[M + H]^+$ Calcd for $C_{23}H_{28}NO_4^+$ 382.2013; Found 382.2013.

IR (film): ν_{max} (cm^{-1}) 2978, 2930, 1735, 1624, 1577, 1446, 1369, 1277, 1151, 1096, 1029, 847, 782, 698.

Optical rotation: $[\alpha]_D^{25}$ = -94.19 (*c* = 0.920, $CHCl_3$, 91% ee).

HPLC: DAICEL CHIRALPAK IC-3, hexane/*i*-PrOH = 45/5, flow rate: 0.5 mL/min, λ = 254 nm, t_R(minor) = 19.7 min, t_R(major) = 25.2 min, 91% ee.

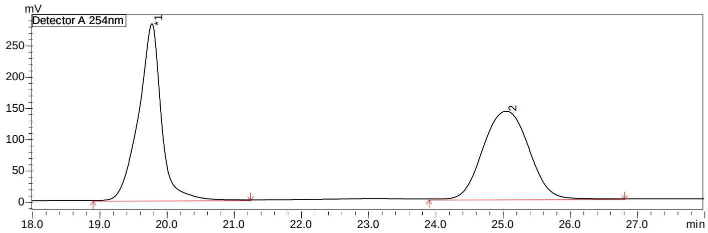

Peak#	Ret. Time	Area%
1	19.784	49.876
2	25.056	50.124

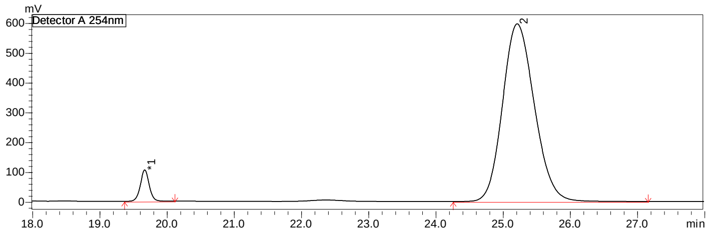

Peak#	Ret. Time	Area%
1	19.679	4.695
2	25.220	95.305

tert-butyl (*S*)-2-((diphenylmethylene)amino)propanoate (**3aw**)

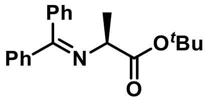

Procedure B. Purification by flash column chromatography (PE:EA = 70:1) afforded the product as a colorless oil (43.7 mg, 71% yield).

^{1}H NMR (400 MHz, $CDCl_3$) δ 7.67 – 7.60 (m, 2H), 7.49 – 7.42 (m, 3H), 7.41 – 7.35 (m, 1H), 7.35 – 7.29 (m, 2H), 7.22 – 7.16 (m, 2H), 4.03 (q, J = 6.7 Hz, 1H), 1.44 (s, 9H), 1.40 (d, J = 6.7 Hz, 3H) ppm. The spectroscopic data match the literature.[11]

Optical rotation: $[\alpha]_D^{25}$ = -56.91 (c = 0.900, $CHCl_3$, 93% ee).

The comparison of the optical rotation of the product **3aw** with **(*S*)-3aw** ($[\alpha]_D^{23}$ = -60.7 (c = 1.69, $CHCl_3$, 97% ee)) in literature[15] indicated that the configuration of the product was *S*.

HPLC: DAICEL CHIRALPAK IE, hexane/*i*-PrOH = 99/1, flow rate: 1.0 mL/min, λ = 254 nm, t_R(minor) = 9.2 min, t_R(major) = 10.0 min, 93% ee.

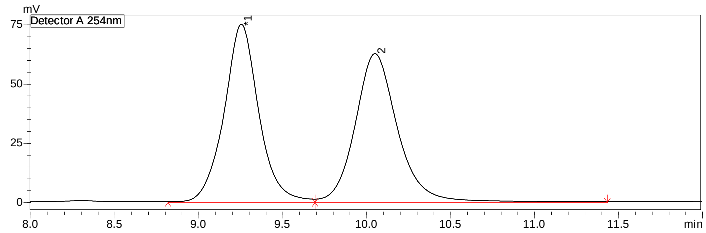

Peak#	Ret. Time	Area%
1	9.258	49.691
2	10.053	50.309

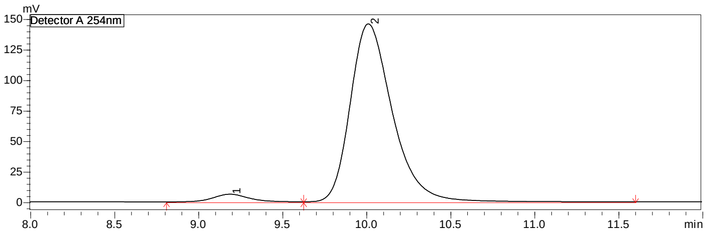

Peak#	Ret. Time	Area%
1	9.190	3.518
2	10.014	96.482

tert-butyl (*S*)-2-((diphenylmethylene)amino)hexanoate (**3ax**)

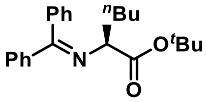

Procedure B. Purification by flash column chromatography (PE:EA = 100:1) afforded the product as a colorless oil (57.0 mg, 81% yield).

^{1}H NMR (400 MHz, $CDCl_3$) δ 7.65 (d, J = 7.3 Hz, 2H), 7.48 – 7.40 (m, 3H), 7.41 – 7.35 (m, 1H), 7.32 (t, J = 7.3 Hz, 2H), 7.21 – 7.13 (m, 2H), 3.90 (t, J = 6.5 Hz, 1H), 1.95 – 1.80 (m, 2H), 1.44 (s, 9H), 1.32 – 1.11 (m, 4H), 0.86 (t, J = 6.9 Hz, 3H) ppm.

The spectroscopic data match the literature.[23]

HPLC: DAICEL CHIRALPAK IE, hexane/*i*-PrOH = 99/1, flow rate: 1.0 mL/min, λ = 254 nm, t_R(minor) = 8.3 min, t_R(major) = 9.4 min, 97% ee.

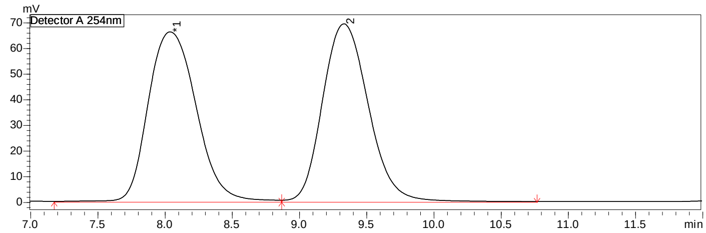

Peak#	Ret. Time	Area%
1	8.043	49.991
2	9.336	50.009

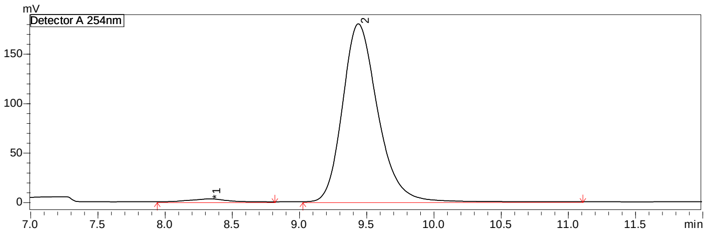

Peak#	Ret. Time	Area%
1	8.338	1.581
2	9.443	98.419

tert-butyl (*S*)-2-((diphenylmethylene)amino)decanoate (**3ay**)

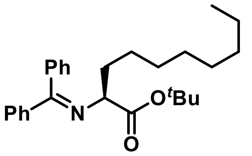

Procedure B. Purification by flash column chromatography (PE:EA = 100:1) afforded the product as a yellow oil (78.3 mg, 96% yield).

^{1}H NMR (400 MHz, $CDCl_3$) δ 7.68 – 7.62 (m, 2H), 7.46 – 7.40 (m, 3H), 7.40 – 7.35 (m, 1H), 7.35 – 7.29 (m, 2H), 7.20 – 7.14 (m, 2H), 3.90 (t, *J* = 6.5 Hz, 1H), 1.87 (q, *J* = 6.6, 6.0 Hz, 2H), 1.44 (s, 9H), 1.33 – 1.14 (m, 12H), 0.87 (t, *J* = 6.8 Hz, 3H) ppm.

The spectroscopic data match the literature.[23]

HPLC: DAICEL CHIRALPAK IE, hexane/*i*-PrOH = 49/1, flow rate: 0.5 mL/min, λ = 254 nm, t_R(minor) = 11.5 min, t_R(major) = 12.4 min, 97% ee.

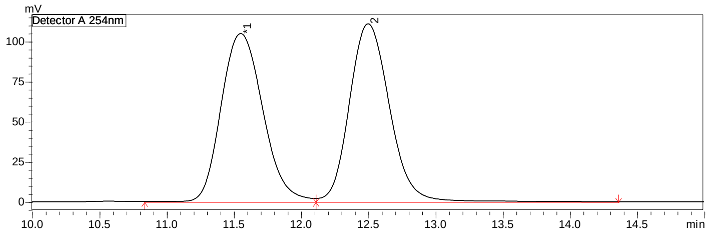

Peak#	Ret. Time	Area%
1	11.553	49.550
2	12.500	50.450

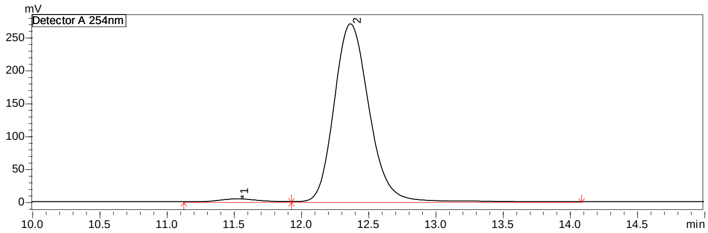

Peak#	Ret. Time	Area%
1	11.530	1.622
2	12.369	98.378

tert-butyl (*S*)-2-((diphenylmethylene)amino)-4-methylpentanoate (**3az**)

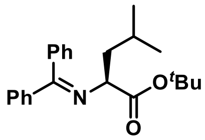

Procedure C. Purification by flash column chromatography (PE:EA = 80:1) afforded the product as a white solid (60.5 mg, 86% yield, m.p. 83-86 ℃).

^{1}H NMR (400 MHz, $CDCl_3$) δ 7.67 – 7.61 (m, 2H), 7.46 – 7.41 (m, 3H), 7.40 – 7.35 (m, 1H), 7.35 – 7.29 (m, 2H), 7.21 – 7.16 (m, 2H), 3.95 (dd, *J* = 8.8, 4.9 Hz, 1H), 1.84 (ddd, *J* = 14.0, 8.9, 5.3 Hz, 1H), 1.73 (ddd, *J* = 13.4, 8.5, 5.0 Hz, 1H), 1.66 – 1.52 (m, 1H), 1.45 (s, 9H), 0.85 (d, *J* = 6.6 Hz, 3H), 0.68 (d, *J* = 6.6 Hz, 3H) ppm.

The spectroscopic data match the literature.[24]

HPLC: DAICEL CHIRALPAK IE, hexane/*i*-PrOH = 495/5, flow rate: 0.5 mL/min, λ = 254 nm, t_R(minor) = 17.9 min, t_R(major) = 19.5 min, 92% ee.

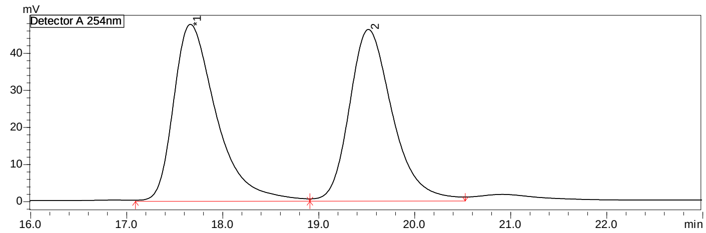

Peak#	Ret. Time	Area%
1	17.671	50.525
2	19.525	49.475

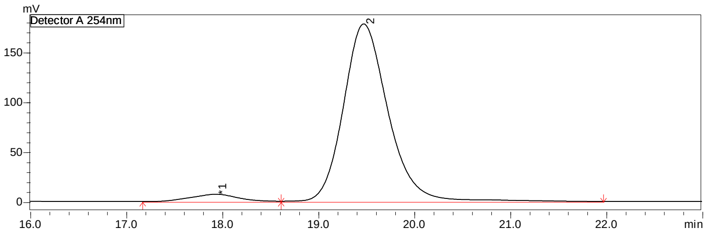

Peak#	Ret. Time	Area%
1	17.935	4.129
2	19.476	95.871

tert-butyl (*S*)-2-((diphenylmethylene)amino)-5-hydroxypentanoate (**3aa'**)

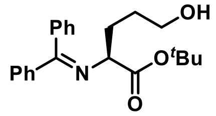

Procedure C. Purification by flash column chromatography (PE:EA = 6:1) afforded the product as a yellow oil (51.8 mg, 73% yield).

^{1}H NMR (400 MHz, $CDCl_3$) δ 7.65 – 7.59 (m, 2H), 7.48 – 7.42 (m, 3H), 7.41 – 7.36 (m, 1H), 7.36 – 7.30 (m, 2H), 7.20 – 7.14 (m, 2H), 4.02 (dd, *J* = 7.0, 4.3 Hz, 1H), 3.71 – 3.55 (m, 2H), 3.24 (s, 1H), 2.15 – 2.04 (m, 1H), 1.93 – 1.81 (m, 1H), 1.74 – 1.57 (m, 2H), 1.44 (s, 9H) ppm.

^{13}C{^{1}H} NMR (101 MHz, $CDCl_3$) δ 171.0, 170.7, 139.2, 136.3, 130.4, 128.7, 128.6, 128.4, 128.0, 127.6, 81.1, 65.1, 62.3, 30.7, 29.1, 27.9 ppm.

HRMS (ESI-TOF) m/z: $[M + H]^+$ Calcd for $C_{22}H_{28}NO_3^+$ 354.2064; Found 354.2063.

IR (film): ν_{max} (cm^{-1}) 3427, 2930, 2869, 1732, 1622, 1598, 1446, 1368, 1286, 1258, 1151, 1058, 1029, 846, 781, 697.

Optical rotation: $[\alpha]_D^{25}$ = -64.96 (*c* = 0.910, $CHCl_3$, 86% ee).

HPLC: DAICEL CHIRALPAK IE, hexane/*i*-PrOH = 80/20, flow rate: 1.0 mL/min, λ = 254 nm, t_R(minor) = 6.9 min, t_R(major) = 8.8 min, 86% ee.

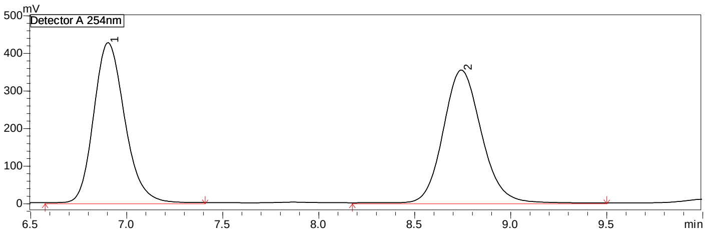

Peak#	Ret. Time	Area%
1	6.907	49.547
2	8.746	50.453

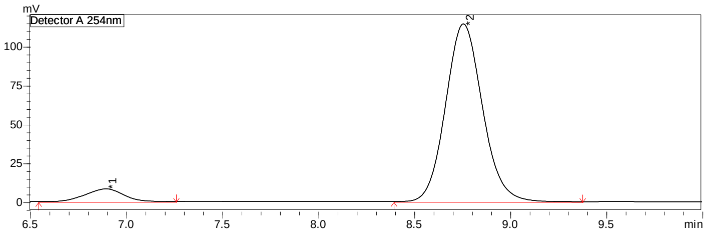

Peak#	Ret. Time	Area%
1	6.896	6.941
2	8.757	93.059

(*S*)-5-(*tert*-butoxy)-4-((diphenylmethylene)amino)-5-oxopentyl benzoate (**3ab'**)

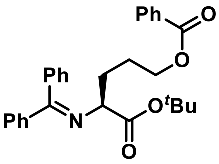

Procedure C. Purification by flash column chromatography (PE:acetone = 80:1) afforded the product as a yellow oil (89.0 mg, 97% yield).

^{1}H NMR (400 MHz, $CDCl_3$) δ 8.03 (d, *J* = 7.8 Hz, 2H), 7.66 (d, *J* = 7.5 Hz, 2H), 7.54 (t, *J* = 7.3 Hz, 1H), 7.47 – 7.36 (m, 6H), 7.32 (t, *J* = 7.4 Hz, 2H), 7.21 – 7.14 (m, 2H), 4.32 – 4.22 (m, 2H), 4.00 (t, *J* = 6.4 Hz, 1H), 2.06 (q, *J* = 7.3, 6.8 Hz, 2H), 1.89 – 1.67 (m, 2H), 1.45 (s, 9H) ppm.

^{13}C{^{1}H} NMR (101 MHz, $CDCl_3$) δ 171.1, 170.3, 166.5, 139.5, 136.5, 132.8, 130.3, 130.2, 129.5, 128.7, 128.5, 128.4, 128.2, 128.0, 127.7, 81.0, 65.5, 64.7, 30.2, 28.0, 25.3 ppm.

HRMS (ESI-TOF) m/z: [M + H]$^+$ Calcd for $C_{29}H_{32}NO_4^+$ 458.2326; Found 458.2326.

IR (film): ν_{max} (cm^{-1}) 3060, 2975, 2930, 1720, 1623, 1600, 1448, 1368, 1315, 1274, 1151, 1112, 1070, 1027, 962, 847, 781, 712.

Optical rotation: $[\alpha]_D^{25}$ = -55.96 (*c* = 0.820, $CHCl_3$, 92% ee).

HPLC: DAICEL CHIRALPAK IC, hexane/*i*-PrOH = 95/5, flow rate: 1.0 mL/min, λ = 254 nm, t_R(minor) = 10.5 min, t_R(major) = 12.5 min, 92% ee.

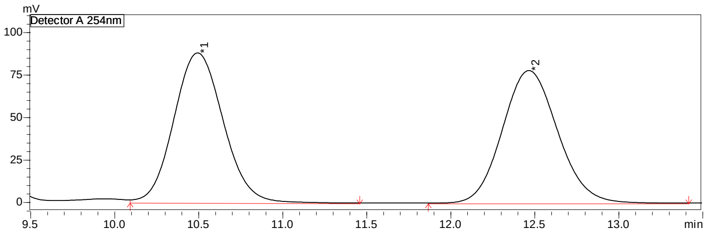

Peak#	Ret. Time	Area%
1	10.498	50.201
2	12.470	49.799

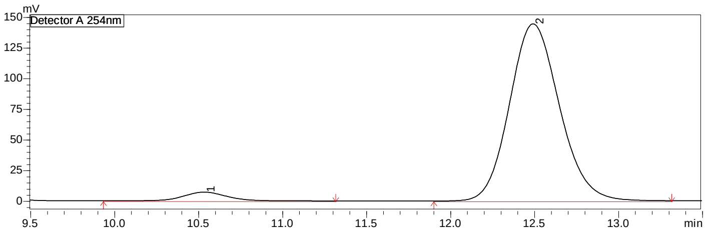

Peak#	Ret. Time	Area%
1	10.537	4.059
2	12.495	95.941

di-*tert*-butyl (2*S*,9*S*)-2,9-bis((diphenylmethylene)amino)decanedioate (**3ac'**)

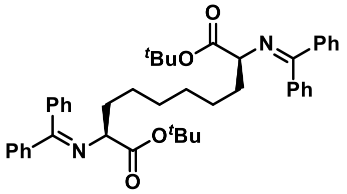

Procedure D. Purification by flash column chromatography (PE:EA = 30:1) afforded the product as a yellow oil (95.1 mg, 71% yield, 9.7:1 dr (Dr was determined by HPLC analysis of **3az** after column chromatography)).

^{1}H NMR (400 MHz, $CDCl_3$) δ 7.67 – 7.59 (m, 4H), 7.45 – 7.34 (m, 8H), 7.34 – 7.27 (m, 4H), 7.21 – 7.08 (m, 4H), 3.88 (t, *J* = 6.5 Hz, 2H), 1.85 (q, *J* = 7.0 Hz, 4H), 1.43 (s, 18H), 1.31 – 1.07 (m, 8H) ppm.

^{13}C{^{1}H} NMR (101 MHz, $CDCl_3$) δ 171.6, 169.7, 139.7, 136.7, 130.0, 128.7, 128.4, 128.3, 127.9, 127.8, 80.7, 66.0, 33.6, 29.3, 28.0, 26.0 ppm.

HRMS (ESI-TOF) m/z: [M + H]$^+$ Calcd for $C_{44}H_{53}N_2O_4^+$ 673.4000; Found 673.4000.

IR (film): ν_{max} (cm^{-1}) 2976, 2928, 2856, 1735, 1624, 1577, 1490, 1446, 1392, 1367, 1286, 1257, 1148, 1074, 1029, 848, 780, 696.

Optical rotation: $[\alpha]_D^{25}$ = -71.14 (*c* = 0.810, $CHCl_3$, 99% ee, 9.7:1 dr).

HPLC: tadem of DAICEL CHIRALPAK OX-3 and ID in sequence, hexane/*i*-PrOH = 485/15, flow rate: 0.5 mL/min, λ = 254 nm, t_R(minor) = 36.2 min, t_R(major) = 45.7 min, 99% ee.

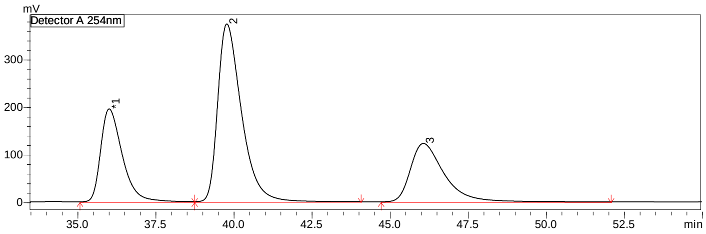

Peak#	Ret. Time	Area%
1	36.029	24.466
2	39.795	51.609
3	46.088	23.925

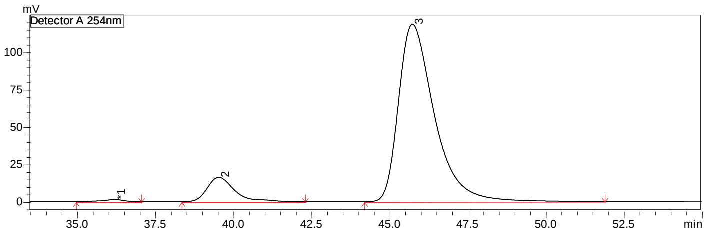

Peak#	Ret. Time	Area%
1	36.204	0.627
2	39.542	9.367
3	45.744	90.006

methyl (*S*)-2-((((9*H*-fluoren-9-yl)methoxy)carbonyl)amino)-2-methylpent-4-enoate (**3ba**)

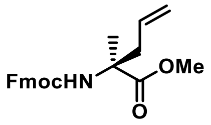

Procedure E. Purification by flash column chromatography (PE:EA = 30:1) afforded the product as a colorless oil (67.3 mg, 92% yield).

^{1}H NMR (400 MHz, $CDCl_3$) δ 7.75 (d, *J* = 7.5 Hz, 2H), 7.59 (d, *J* = 7.3 Hz, 2H), 7.39 (t, *J* = 7.4 Hz, 2H), 7.30 (t, *J* = 7.3 Hz, 2H), 5.84 – 5.35 (m, 2H), 5.24 – 4.93 (m, 2H), 4.54 – 4.26 (m, 2H), 4.21 (t, *J* = 6.8 Hz, 1H), 3.74 (s, 3H), 3.01 – 2.72 (m, 1H), 2.70 – 2.38 (m, 1H), 1.59 (s, 3H) ppm.

$^{13}C\{^{1}H\}$ NMR (101 MHz, $CDCl_3$) δ 174.2, 154.6, 143.8, 141.2, 132.1, 127.6, 127.0, 125.0, 119.9, 119.4, 66.4, 59.5, 52.6, 47.2, 41.1, 23.1 ppm.

HRMS (ESI-TOF) m/z: $[M + H]^+$ Calcd for $C_{22}H_{24}NO_4^+$ 366.1700; Found 366.1700.

IR (film): ν_{max} (cm^{-1}) 3416, 3355, 3066, 2950, 1724, 1503, 1450, 1376, 1322, 1232, 1152, 1090, 995, 923, 759, 740.

Optical rotation: $[\alpha]_D^{25}$ = +5.56 (*c* = 0.970, $CHCl_3$, 98% ee).

HPLC: DAICEL CHIRALPAK IC, hexane/*i*-PrOH = 70/30, flow rate: 1.0 mL/min, λ = 254 nm, t_R(minor) = 7.6 min, t_R(major) = 14.2 min, 98% ee.

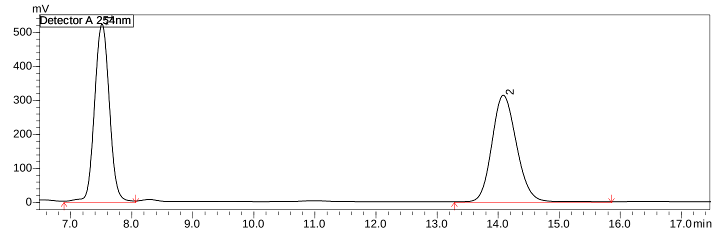

Peak#	Ret. Time	Area%
1	7.525	50.265
2	14.097	49.735

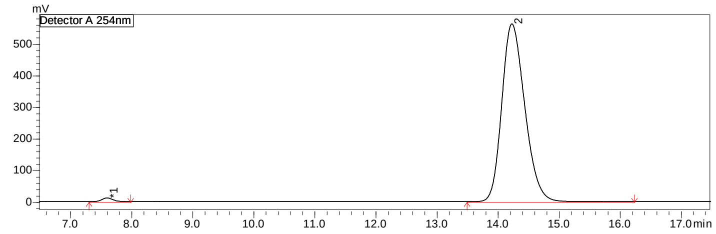

Peak#	Ret. Time	Area%
1	7.609	0.964
2	14.238	99.036

methyl (*S*)-2-((((9*H*-fluoren-9-yl)methoxy)carbonyl)amino)-2,4-dimethylpent-4-enoate (**3bd**)

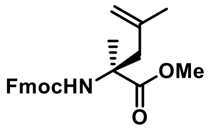

Procedure E. Purification by flash column chromatography (PE:acetone = 50:1) afforded the product as a colorless oil (73.6 mg, 97% yield).

^{1}H NMR (400 MHz, $CDCl_3$) δ 7.75 (d, *J* = 7.5 Hz, 2H), 7.59 (dd, *J* = 7.5, 3.0 Hz, 2H), 7.39 (t, *J* = 7.4 Hz, 2H), 7.30 (t, *J* = 7.4 Hz, 2H), 5.79 (s, 1H), 4.86 (s, 1H), 4.71 (s, 1H), 4.49 – 4.26 (m, 2H), 4.22 (t, *J* = 6.7 Hz, 1H), 3.76 (s, 3H), 2.96 (d, *J* = 13.5 Hz, 1H), 2.56 (d, *J* = 13.4 Hz, 1H), 1.65 (s, 6H) ppm.

^{13}C{^{1}H} NMR (101 MHz, $CDCl_3$) δ 174.6, 154.4, 143.9, 143.9, 141.3, 140.8, 127.6, 127.6, 127.0, 125.0, 119.9, 115.6, 66.4, 59.6, 52.6, 47.2, 44.3, 24.1, 23.1 ppm.

HRMS (ESI-TOF) m/z: $[M + H]^+$ Calcd for $C_{23}H_{26}NO_4^+$ 380.1856; Found 380.1856.

IR (film): ν_{max} (cm^{-1}) 3419, 2950, 1724, 1502, 1451, 1376, 1315, 1222, 1111, 1076, 900, 759, 740.

Optical rotation: $[\alpha]_D^{25}$ = +10.76 (*c* = 0.970, $CHCl_3$, 97% ee).

HPLC: DAICEL CHIRALPAK IBN-3, hexane/*i*-PrOH = 30/20, flow rate: 0.5 mL/min, λ = 254 nm, t_R(minor) = 15.6 min, t_R(major) = 18.4 min, 97% ee.

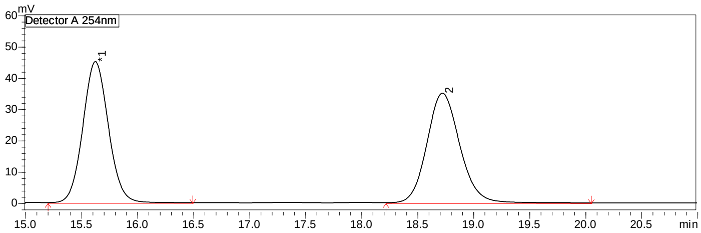

Peak#	Ret. Time	Area%
1	15.628	50.072
2	18.727	49.928

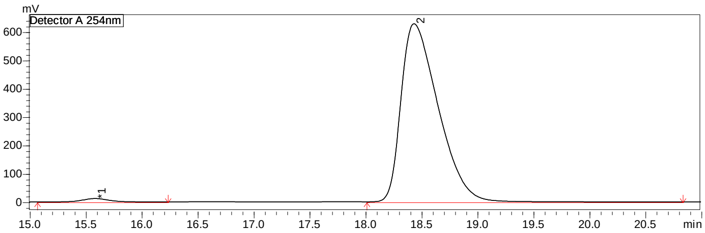

Peak#	Ret. Time	Area%
1	15.587	1.265
2	18.436	98.735

methyl (*S*)-2-((((9*H*-fluoren-9-yl)methoxy)carbonyl)amino)-2-methylhex-4-ynoate (**3be**)

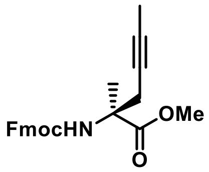

Procedure E. Purification by flash column chromatography (PE:EA = 30:1) afforded the product as a colorless oil (54.9 mg, 73% yield).

^{1}H NMR (400 MHz, $CDCl_3$) δ 7.76 (d, *J* = 7.5 Hz, 2H), 7.61 (d, *J* = 7.3 Hz, 2H), 7.39 (t, *J* = 7.4 Hz, 2H), 7.31 (t, *J* = 7.4 Hz, 2H), 5.68 (s, 1H), 4.35 (d, *J* = 7.2 Hz, 2H), 4.25 (t, *J* = 6.8 Hz, 1H), 3.77 (s, 3H), 3.01 – 2.67 (m, 2H), 1.78 (s, 3H), 1.60 (s, 3H) ppm.

^{13}C{^{1}H} NMR (101 MHz, $CDCl_3$) δ 173.5, 154.7, 143.9, 143.9, 141.3, 127.6, 127.0, 125.1, 119.9, 78.9, 73.5, 66.6, 59.1, 52.8, 47.2, 27.6, 23.0, 3.5 ppm.

HRMS (ESI-TOF) m/z: $[M + H]^+$ Calcd for $C_{23}H_{24}NO_4^+$ 378.1700; Found 378.1699.

IR (film): ν_{max} (cm^{-1}) 3358, 2951, 2922, 1725, 1503, 1450, 1376, 1323, 1275, 1231, 1116, 1077, 759, 740.

Optical rotation: $[\alpha]_D^{25}$ = -11.24 (*c* = 1.276, $CHCl_3$, 96% ee).

HPLC: DAICEL CHIRALPAK IC, hexane/*i*-PrOH = 70/30, flow rate: 1.0 mL/min, λ = 254 nm, t_R(minor) = 8.6 min, t_R(major) = 19.4 min, 96% ee.

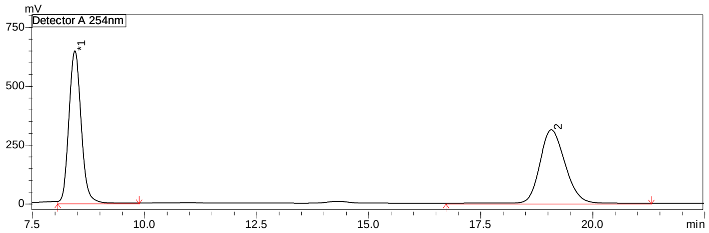

Peak#	Ret. Time	Area%
1	8.457	50.424
2	19.091	49.576

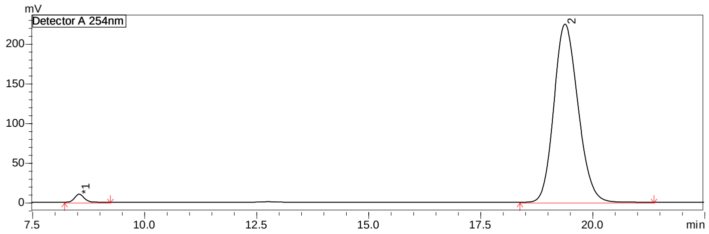

Peak#	Ret. Time	Area%
1	8.554	1.753
2	19.397	98.247

methyl (*S*)-2-((((9*H*-fluoren-9-yl)methoxy)carbonyl)amino)-3-(4-(*tert*-butyl)phenyl)-2-methylpropanoate (**3bh**)

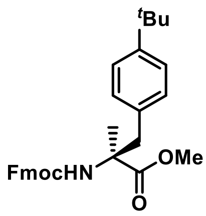

Procedure E. Purification by flash column chromatography (PE:acetone = 50:1) afforded the product as a colorless oil (93.3 mg, 99% yield).

^{1}H NMR (400 MHz, $CDCl_3$) δ 7.77 (d, *J* = 7.4 Hz, 2H), 7.64 – 7.54 (m, 2H), 7.40 (t, *J* = 7.1 Hz, 2H), 7.31 (q, *J* = 6.7 Hz, 2H), 7.22 (d, *J* = 8.3 Hz, 2H), 6.92 (d, *J* = 7.7 Hz, 2H), 5.47 (s, 1H), 4.50 (t, *J* = 8.8 Hz, 1H), 4.37 (t, *J* = 8.8 Hz, 1H), 4.27 (t, *J* = 6.4 Hz, 1H), 3.77 (s, 3H), 3.38 (d, *J* = 13.2 Hz, 1H), 3.18 (d, *J* = 13.1 Hz, 1H), 1.63 (s, 3H), 1.28 (s, 9H) ppm.

$^{13}C\{^1H\}$ NMR (101 MHz, $CDCl_3$) δ 174.1, 154.6, 149.6, 143.9, 143.8, 141.3, 141.2, 132.9, 129.5, 127.6, 127.6, 127.0, 125.1, 125.1, 125.0, 119.9, 119.9, 66.3, 60.6, 52.5, 47.2, 41.0, 34.3, 31.3, 31.3, 31.3, 23.5 ppm.

HRMS (ESI-TOF) m/z: $[M + H]^+$ Calcd for $C_{30}H_{34}NO_4^+$ 472.2482; Found 472.2483.

IR (film): ν_{max} (cm^{-1}) 3418, 2961, 2869, 1724, 1504, 1451, 1365, 1329, 1267, 1223, 1109, 1059, 759, 740.

Optical rotation: $[\alpha]_D^{25}$ = +14.39 (*c* = 0.910, $CHCl_3$, 98% ee).

HPLC: DAICEL CHIRALPAK IBN-3, hexane/*i*-PrOH = 30/20, flow rate: 0.5 mL/min, λ = 254 nm, t_R(minor) = 13.4 min, t_R(major) = 23.7 min, 98% ee.

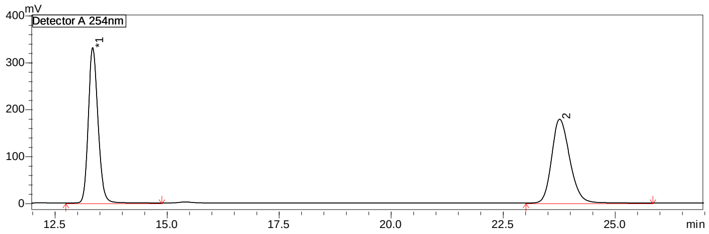

Peak#	Ret. Time	Area%
1	13.357	49.919
2	23.779	50.081

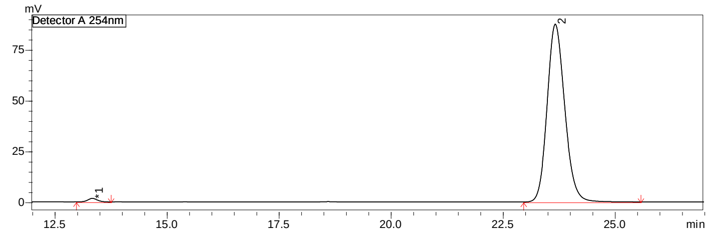

Peak#	Ret. Time	Area%
1	13.351	1.031
2	23.680	98.969

methyl (*S*)-2-((((9*H*-fluoren-9-yl)methoxy)carbonyl)amino)-3-(2-chlorophenyl)-2-methylpropanoate (**3bi**)

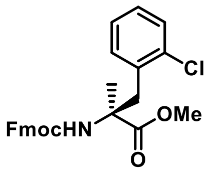

Procedure E. Purification by flash column chromatography (PE:acetone = 50:1) afforded the product as a colorless oil (87.0 mg, 97% yield).

^{1}H NMR (400 MHz, $CDCl_3$) δ 7.75 (d, *J* = 7.4 Hz, 2H), 7.59 (t, *J* = 6.3 Hz, 2H), 7.47 – 7.24 (m, 5H), 7.19 – 6.92 (m, 3H), 5.48 (s, 1H), 4.58 – 4.28 (m, 2H), 4.22 (t, *J* = 6.3 Hz, 1H), 3.73 (s, 3H), 3.59 – 3.26 (m, 2H), 1.58 (s, 3H) ppm.

^{13}C{^{1}H} NMR (101 MHz, $CDCl_3$) δ 173.8, 154.7, 143.8, 143.7, 141.3, 134.8, 133.9, 132.2, 129.6, 128.3, 127.6, 127.0, 126.6, 125.0, 119.9, 66.4, 60.3, 52.7, 47.2, 38.3, 23.1 ppm.

HRMS (ESI-TOF) m/z: $[M + H]^+$ Calcd for $C_{26}H_{25}ClNO_4^+$ 450.1467; Found 450.1467.

IR (film): ν_{max} (cm^{-1}) 3416, 3355, 3065, 2951, 1724, 1502, 1450, 1377, 1326, 1267, 1224, 1110, 1061, 758, 740.

Optical rotation: $[\alpha]_D^{25}$ = +1.72 (*c* = 1.100, $CHCl_3$, 98% ee).

HPLC: DAICEL CHIRALPAK IBN-3, hexane/*i*-PrOH = 30/20, flow rate: 0.5 mL/min, λ = 254 nm, t_R(minor) = 17.2 min, t_R(major) = 28.7 min, 98% ee.

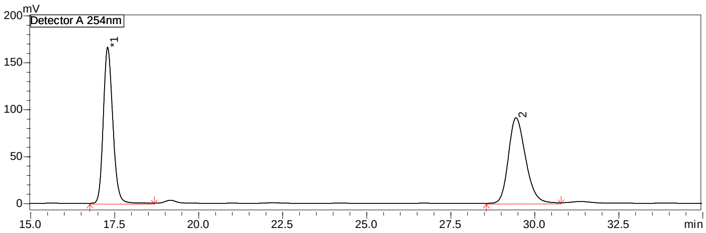

Peak#	Ret. Time	Area%
1	17.312	50.052
2	29.464	49.948

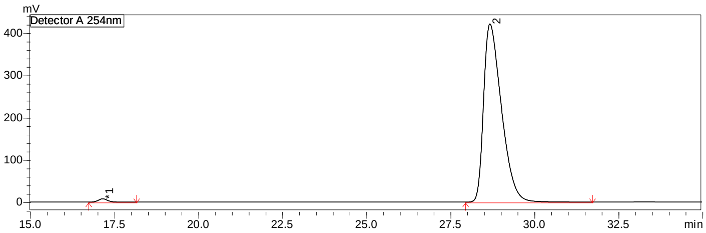

Peak#	Ret. Time	Area%
1	17.167	0.926
2	28.690	99.074

methyl (*S*)-2-((((9*H*-fluoren-9-yl)methoxy)carbonyl)amino)-3-(3,5-dimethylphenyl)-2-methylpropanoate (**3bl**)

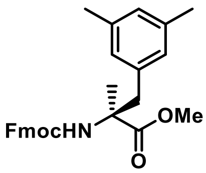

Procedure E. Purification by flash column chromatography (PE:acetone = 50:1) afforded the product as a colorless oil (87.7 mg, 99% yield).

^{1}H NMR (400 MHz, $CDCl_3$) δ 7.75 (d, *J* = 7.5 Hz, 2H), 7.57 (dd, *J* = 15.1, 7.4 Hz, 2H), 7.39 (t, *J* = 7.3 Hz, 2H), 7.34 – 7.24 (m, 2H), 6.85 (s, 1H), 6.67 (s, 2H), 5.55 (s, 1H), 4.59 – 4.16 (m, 3H), 3.77 (s, 3H), 3.36 (d, *J* = 12.6 Hz, 1H), 3.13 (d, *J* = 12.8 Hz, 1H), 2.20 (s, 6H), 1.65 (s, 3H) ppm.

^{13}C{^{1}H} NMR (101 MHz, $CDCl_3$) δ 174.1, 154.7, 143.9, 143.8, 141.2, 141.2, 137.6, 135.8, 128.6, 127.6, 127.0, 125.0, 119.9, 66.6, 60.7, 52.4, 47.2, 41.8, 23.5, 21.2 ppm.

HRMS (ESI-TOF) m/z: $[M + H]^+$ Calcd for $C_{28}H_{30}NO_4^+$ 444.2169; Found 444.2169.

IR (film): ν_{max} (cm^{-1}) 3419, 2949, 1724, 1605, 1503, 1450, 1375, 1308, 1268, 1222, 1108, 1060, 852, 759, 740.

Optical rotation: $[\alpha]_D^{25}$ = +20.17 (*c* = 0.945, $CHCl_3$, 99% ee).

HPLC: DAICEL CHIRALPAK IBN-3, hexane/*i*-PrOH = 30/20, flow rate: 0.5 mL/min, λ = 254 nm, t_R(minor) = 15.5 min, t_R(major) = 25.8 min, 99% ee.

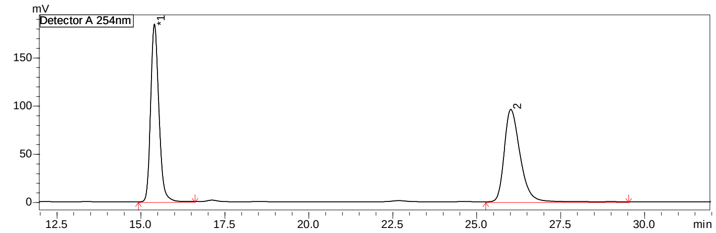

Peak#	Ret. Time	Area%
1	15.424	50.227
2	26.038	49.773

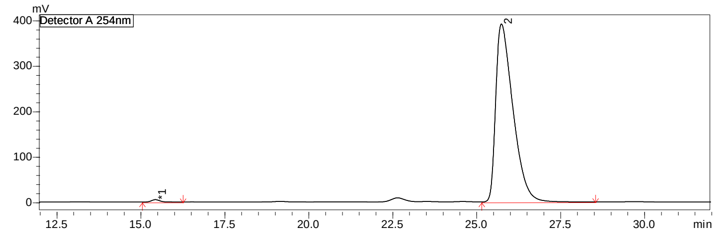

Peak#	Ret. Time	Area%
1	15.454	0.663
2	25.758	99.337

methyl (*S*)-2-benzamido-2-methyl-4-oxo-4-phenylbutanoate (**3bu**)

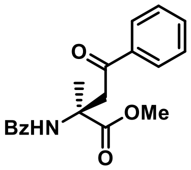

Procedure F. Purification by flash column chromatography (PE:EA = 10:1) afforded the product as a colorless oil (49.2 mg, 76% yield).

^{1}H NMR (400 MHz, $CDCl_3$) δ 7.97 – 7.90 (m, 2H), 7.78 – 7.69 (m, 2H), 7.63 (s, 1H), 7.54 (tt, *J* = 6.9, 1.2 Hz, 1H), 7.49 – 7.33 (m, 5H), 4.68 (d, *J* = 18.0 Hz, 1H), 3.81 (s, 3H), 3.56 (d, *J* = 18.0 Hz, 1H), 1.86 (s, 3H) ppm.

$^{13}C\{^{1}H\}$ NMR (126 MHz, $CDCl_3$) δ 197.5, 174.9, 166.5, 136.2, 134.5, 133.5, 131.5, 128.6, 128.4, 128.1, 126.9, 57.8, 53.0, 44.0, 23.6 ppm.

HRMS (ESI-TOF) m/z: $[M + H]^+$ Calcd for $C_{19}H_{20}NO_4^+$ 326.1387; Found 326.1387.

IR (film): ν_{max} (cm^{-1}) 3415, 3061, 2954, 2926, 1741, 1685, 1598, 1580, 1522, 1486, 1449, 1400, 1355, 1320, 1217, 1114, 1011, 880, 802, 756, 714, 690.

Optical rotation: $[\alpha]_D^{25}$ = -30.50 (*c* = 1.020, $CHCl_3$, 96% ee).

HPLC: DAICEL CHIRALPAK IE, hexane/*i*-PrOH = 80/20, flow rate: 1.0 mL/min, λ = 254 nm, t_R(major) = 14.2 min, t_R(minor) = 16.3 min, 96% ee.

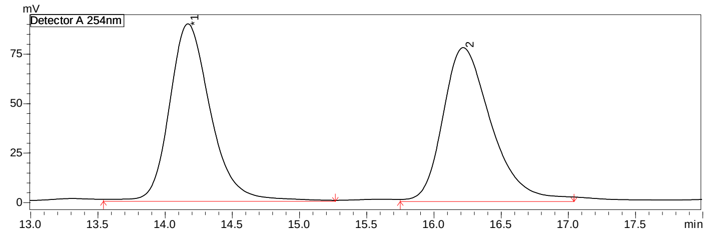

Peak#	Ret. Time	Area%
1	14.175	49.502
2	16.223	50.498

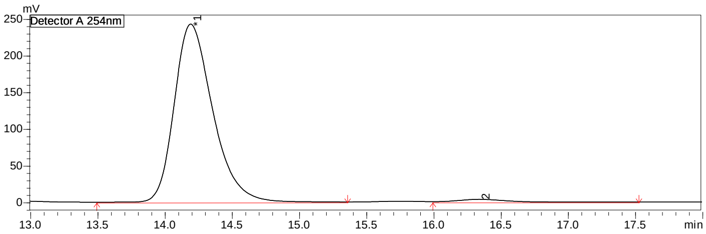

Peak#	Ret. Time	Area%
1	14.196	98.021
2	16.343	1.979

methyl (*S*)-2-((((9*H*-fluoren-9-yl)methoxy)carbonyl)amino)-2-methylheptanoate (**3bx**)

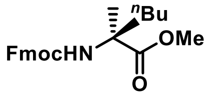

Procedure G. Purification by flash column chromatography (PE:acetone = 50:1) afforded the product as a colorless oil (60.9 mg, 80% yield).

^{1}H NMR (400 MHz, $CDCl_3$) δ 7.76 (d, *J* = 7.5 Hz, 2H), 7.60 (d, *J* = 7.4 Hz, 2H), 7.39 (t, *J* = 7.4 Hz, 2H), 7.31 (td, *J* = 7.4, 1.1 Hz, 2H), 5.63 (s, 1H), 4.37 (s, 2H), 4.22 (t, *J* = 6.8 Hz, 1H), 3.75 (s, 3H), 2.38 – 1.94 (m, 1H), 1.92 – 1.69 (m, 1H), 1.58 (s, 3H), 1.38 – 1.14 (m, 3H), 1.03 (s, 1H), 0.87 (t, *J* = 6.1 Hz, 3H) ppm.

$^{13}C\{^1H\}$ NMR (126 MHz, $CDCl_3$) δ 174.9, 154.4, 143.9, 143.9, 141.3, 127.6, 127.0, 125.0, 119.9, 66.3, 60.0, 52.7, 47.2, 36.6, 26.2, 23.3, 22.5, 13.9 ppm.

HRMS (ESI-TOF) m/z: $[M + H]^+$ Calcd for $C_{23}H_{28}NO_4^+$ 382.2013; Found 382.2013.

IR (film): ν_{max} (cm^{-1}) 3419, 3358, 2955, 1724, 1503, 1450, 1377, 1322, 1280, 1259, 1134, 1107, 1051, 759, 740.

Optical rotation: $[\alpha]_D^{25}$ = +5.82 (*c* = 0.910, $CHCl_3$, 90% ee).

HPLC: DAICEL CHIRALPAK IC, hexane/*i*-PrOH = 80/20, flow rate: 1.0 mL/min, λ = 254 nm, t_R(minor) = 9.4 min, t_R(major) = 14.9 min, 90% ee.

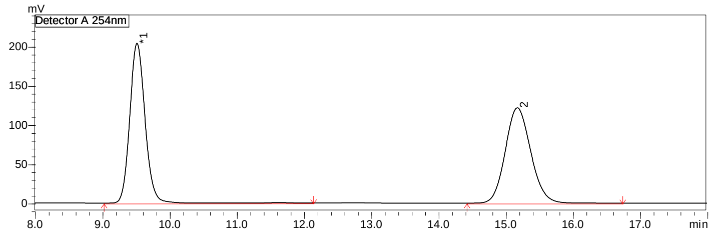

Peak#	Ret. Time	Area%
1	9.519	50.222
2	15.178	49.778

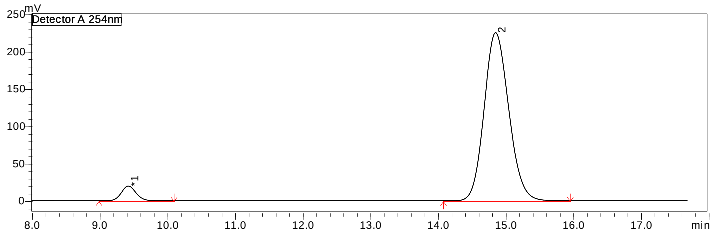

Peak#	Ret. Time	Area%
1	9.429	4.959
2	14.857	95.041

methyl (*S*)-2-((((9*H*-fluoren-9-yl)methoxy)carbonyl)amino)-2-methyldecanoate (**3by**)

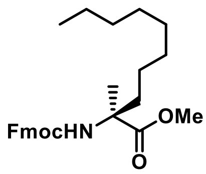

Procedure G. Purification by flash column chromatography (PE:acetone = 50:1) afforded the product as a colorless oil (71.8 mg, 82% yield).

^{1}H NMR (400 MHz, $CDCl_3$) δ 7.76 (d, *J* = 7.5 Hz, 2H), 7.60 (d, *J* = 7.3 Hz, 2H), 7.40 (t, *J* = 7.4 Hz, 2H), 7.31 (td, *J* = 7.4, 1.1 Hz, 2H), 5.64 (s, 1H), 4.36 (s, 2H), 4.22 (t, *J* = 6.8 Hz, 1H), 3.76 (s, 3H), 2.26 – 2.01 (m, 1H), 1.75 (s, 1H), 1.58 (s, 3H), 1.32 – 1.18 (m, 11H), 1.05 (s, 1H), 0.86 (t, *J* = 6.8 Hz, 3H) ppm.

^{13}C{^{1}H} NMR (126 MHz, $CDCl_3$) δ 174.9, 154.4, 143.9, 143.9, 141.3, 127.6, 127.0, 125.0, 119.9, 66.3, 60.0, 52.6, 47.2, 36.9, 31.8, 29.4, 29.3, 29.1, 24.0, 23.3, 22.6, 14.0 ppm.

HRMS (ESI-TOF) m/z: [M + H]$^+$ Calcd for $C_{27}H_{36}NO_4^+$ 438.2639; Found 438.2639.

IR (film): v_{max} (cm^{-1}) 3420, 3359, 2926, 2855, 1725, 1503, 1451, 1377, 1323, 1247, 1133, 1077, 758, 740.

Optical rotation: $[\alpha]_D^{25}$ = +8.30 (*c* = 0.945, $CHCl_3$, 89% ee).

HPLC: DAICEL CHIRALPAK IC, hexane/*i*-PrOH = 80/20, flow rate: 1.0 mL/min, λ = 254 nm, t_R(minor) = 8.1 min, t_R(major) = 12.0 min, 89% ee.

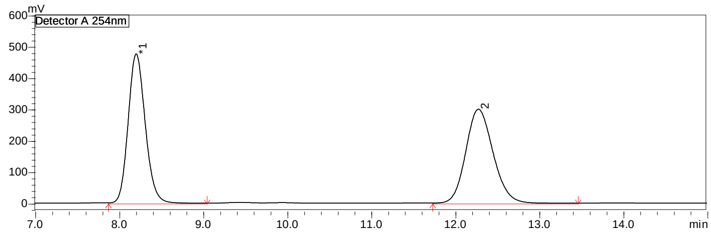

Peak#	Ret. Time	Area%
1	8.205	49.825
2	12.279	50.175

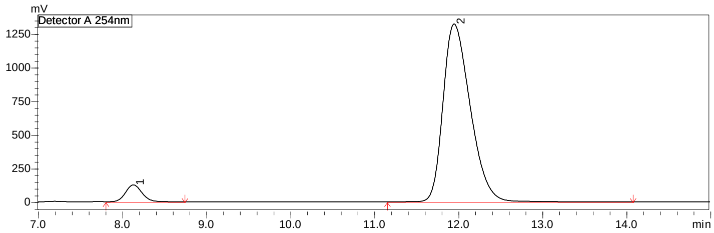

Peak#	Ret. Time	Area%
1	8.136	5.394
2	11.954	94.606

(*S*)-4-((((9*H*-fluoren-9-yl)methoxy)carbonyl)amino)-5-methoxy-4-methyl-5-oxopentyl benzoate (**3bb'**)

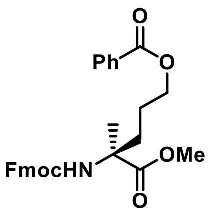

Procedure G. Purification by flash column chromatography (PE:acetone = 100:1) afforded the product as a colorless oil (79.6 mg, 82% yield).

^{1}H NMR (400 MHz, $CDCl_3$) δ 8.08 – 7.98 (m, 2H), 7.75 (d, *J* = 7.5 Hz, 2H), 7.63 – 7.50 (m, 3H), 7.46 – 7.35 (m, 4H), 7.31 (td, *J* = 7.5, 1.2 Hz, 2H), 5.70 (s, 1H), 4.48 – 4.16 (m, 5H), 3.76 (s, 3H), 2.48 – 2.28 (m, 1H), 2.07 – 1.92 (m, 1H), 1.82 – 1.34 (m, 5H) ppm.

^{13}C{^{1}H} NMR (101 MHz, $CDCl_3$) δ 174.5, 166.4, 154.3, 143.8, 141.2, 132.8, 130.1, 129.4, 128.2, 127.6, 126.9, 124.9, 119.9, 66.2, 64.3, 59.7, 52.8, 47.1, 32.7, 23.6, 23.5 ppm.

HRMS (ESI-TOF) m/z: [M + H]$^+$ Calcd for $C_{29}H_{30}NO_6^+$ 488.2068; Found 488.2068.

IR (film): ν_{max} (cm^{-1}) 3361, 2953, 1720, 1506, 1451, 1316, 1274, 1109, 1027, 759, 741, 713.

Optical rotation: $[\alpha]_D^{25}$ = +6.66 (*c* = 0.835, $CHCl_3$, 86% ee).

HPLC: DAICEL CHIRALPAK IC-3, hexane/*i*-PrOH = 15/25, flow rate: 0.4 mL/min, λ = 254 nm, t_R(minor) = 22.5 min, t_R(major) = 35.9 min, 86% ee.

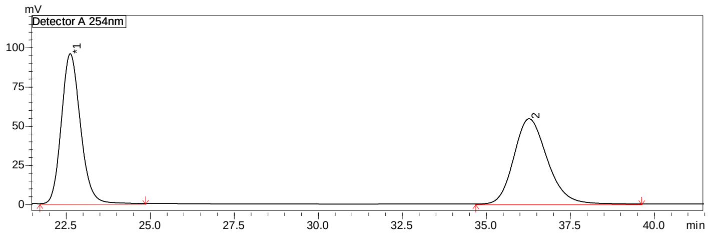

Peak#	Ret. Time	Area%
1	22.640	50.052
2	36.294	49.948

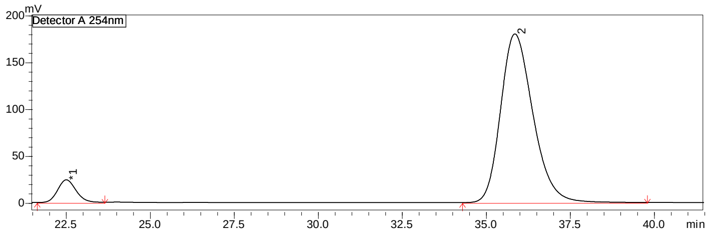

Peak#	Ret. Time	Area%
1	22.523	7.108
2	35.871	92.892

methyl (*R*)-2-((((9*H*-fluoren-9-yl)methoxy)carbonyl)amino)-2-benzylpent-4-enoate (**3ca**)

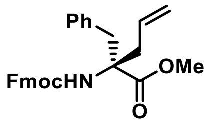

Procedure E. Purification by flash column chromatography (PE:acetone = 100:1) afforded the product as a colorless oil (77.7 mg, 88% yield).

^{1}H NMR (400 MHz, $CDCl_3$) δ 7.78 (d, *J* = 7.5 Hz, 2H), 7.59 (dd, *J* = 12.6, 7.5 Hz, 2H), 7.41 (t, *J* = 7.3 Hz, 2H), 7.36 – 7.28 (m, 2H), 7.24 – 7.15 (m, 3H), 7.05 – 6.94 (m, 2H), 5.79 – 5.53 (m, 2H), 5.21 – 5.03 (m, 2H), 4.48 (dd, *J* = 10.5, 7.1 Hz, 1H), 4.38 (dd, *J* = 10.4, 7.2 Hz, 1H), 4.26 (t, *J* = 6.8 Hz, 1H), 3.78 (s, 3H), 3.63 (d, *J* = 13.6 Hz, 1H), 3.28 (dd, *J* = 13.7, 7.1 Hz, 1H), 3.13 (d, *J* = 13.6 Hz, 1H), 2.63 (dd, *J* = 13.8, 7.4 Hz, 1H) ppm.

^{13}C{^{1}H} NMR (101 MHz, $CDCl_3$) δ 172.7, 154.2, 143.8, 143.8, 141.3, 135.9, 132.0, 129.6, 128.3, 127.6, 127.0, 127.0, 126.9, 125.1, 125.1, 119.9, 119.2, 66.3, 65.2, 52.6, 47.2, 40.7, 39.8 ppm.

HRMS (ESI-TOF) m/z: $[M + H]^+$ Calcd for $C_{28}H_{28}NO_4^+$ 442.2013; Found 442.2013.

IR (film): ν_{max} (cm^{-1}) 3419, 3064, 3030, 2951, 1721, 1498, 1449, 1345, 1311, 1231, 1078, 1026, 925, 741, 703, 564, 540.

Optical rotation: $[\alpha]_D^{25}$ = -19.71 (*c* = 1.100, $CHCl_3$, 92% ee).

HPLC: DAICEL CHIRALPAK IC, hexane/*i*-PrOH = 80/20, flow rate: 1.0 mL/min, λ = 254 nm, t_R(major) = 7.2 min, t_R(minor) = 8.1 min, 92% ee.

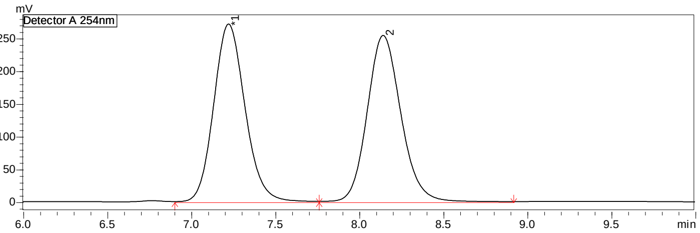

Peak#	Ret. Time	Area%
1	7.224	49.842
2	8.144	50.158

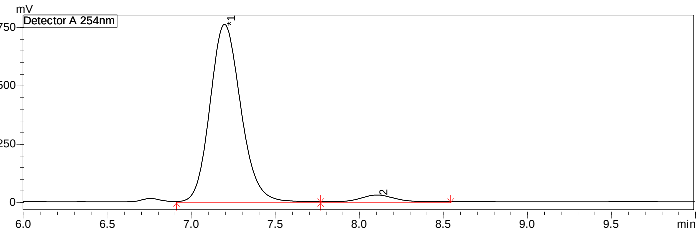

Peak#	Ret. Time	Area%
1	7.199	95.853
2	8.107	4.147

methyl (*S*)-2-((((9*H*-fluoren-9-yl)methoxy)carbonyl)amino)-2-benzylhex-4-ynoate (**3ce**)

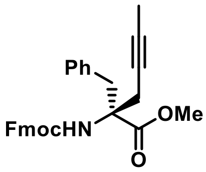

Procedure E. Purification by flash column chromatography (PE:acetone = 50:1) afforded the product as a yellow oil (65.0 mg, 72% yield).

^{1}H NMR (400 MHz, $CDCl_3$) δ 7.78 (d, *J* = 7.4 Hz, 2H), 7.67 – 7.55 (m, 2H), 7.41 (t, *J* = 7.3 Hz, 2H), 7.36 – 7.28 (m, 2H), 7.22 (s, 3H), 7.04 (s, 2H), 5.72 (s, 1H), 4.52 – 4.34 (m, 2H), 4.29 (t, *J* = 7.0 Hz, 1H), 3.79 (s, 3H), 3.53 (d, *J* = 13.4 Hz, 1H), 3.25 (d, *J* = 16.5 Hz, 1H), 3.12 (d, *J* = 13.4 Hz, 1H), 2.78 (d, *J* = 16.6 Hz, 1H), 1.77 (s, 3H) ppm.

$^{13}C\{^{1}H\}$ NMR (101 MHz, $CDCl_3$) δ 172.0, 154.4, 143.8, 141.2, 135.5, 129.6, 128.3, 127.6, 127.0, 127.0, 125.1, 125.1, 119.9, 78.9, 73.4, 66.5, 64.3, 52.8, 47.1, 40.2, 26.2, 3.6 ppm.

HRMS (ESI-TOF) m/z: $[M + H]^+$ Calcd for $C_{29}H_{28}NO_4^+$ 454.2013; Found 454.2012.

IR (film): ν_{max} (cm^{-1}) 3420, 3030, 2951, 2921, 1720, 1499, 1449, 1346, 1312, 1228, 1107, 1079, 1042, 759, 740, 703.

Optical rotation: $[\alpha]_D^{25}$ = -19.13 (*c* = 0.960, $CHCl_3$, 84% ee).

HPLC: DAICEL CHIRALPAK IC, hexane/*i*-PrOH = 80/20, flow rate: 1.0 mL/min, λ = 254 nm, t_R(major) = 12.6 min, t_R(minor) = 17.9 min, 84% ee.

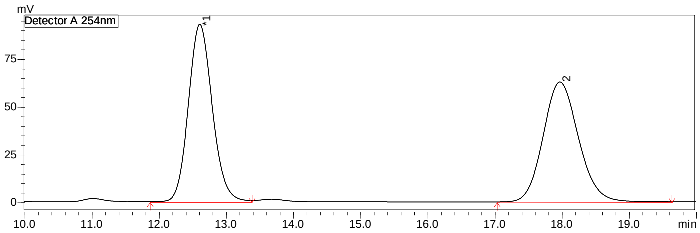

Peak#	Ret. Time	Area%
1	12.614	50.123
2	17.977	49.877

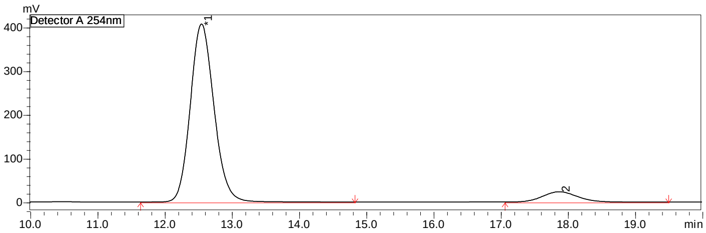

Peak#	Ret. Time	Area%
1	12.552	92.120
2	17.875	7.880

methyl (*R*)-2-((((9*H*-fluoren-9-yl)methoxy)carbonyl)amino)-2-(2-(methylthio)ethyl)pent-4-enoate (**3da**)

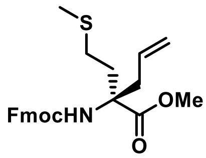

Procedure E. Purification by flash column chromatography (PE:acetone = 50:1) afforded the product as a colorless oil (67.9 mg, 80% yield).

^{1}H NMR (400 MHz, $CDCl_3$) δ 7.77 (d, *J* = 7.5 Hz, 2H), 7.60 (d, *J* = 7.4 Hz, 2H), 7.40 (t, *J* = 7.4 Hz, 2H), 7.32 (t, *J* = 7.4 Hz, 2H), 5.86 (s, 1H), 5.65 – 5.43 (m, 1H), 5.17 – 4.93 (m, 2H), 4.52 – 4.28 (m, 2H), 4.21 (t, *J* = 6.6 Hz, 1H), 3.78 (s, 3H), 3.09 (dd, *J* = 13.6, 7.2 Hz, 1H), 2.79 – 2.58 (m, 1H), 2.57 – 2.32 (m, 2H), 2.30 – 2.17 (m, 1H), 2.17 – 2.07 (m, 1H), 2.04 (s, 3H) ppm.

^{13}C{^{1}H} NMR (101 MHz, $CDCl_3$) δ 173.3, 153.9, 143.8, 143.7, 141.3, 131.7, 127.6, 127.0, 125.0, 119.9, 119.3, 66.2, 63.4, 52.8, 47.2, 39.9, 34.5, 28.7, 15.5 ppm.

HRMS (ESI-TOF) m/z: $[M + H]^+$ Calcd for $C_{24}H_{28}NO_4S^+$ 426.1734; Found 426.1734.

IR (film): ν_{max} (cm^{-1}) 3416, 2951, 2917, 1722, 1499, 1449, 1331, 1272, 1228, 1090, 1043, 924, 759, 741.

Optical rotation: $[\alpha]_D^{25}$ = -15.00 (*c* = 0.940, $CHCl_3$, 96% ee).

HPLC: DAICEL CHIRALPAK IC, hexane/*i*-PrOH = 80/20, flow rate: 1.0 mL/min, λ = 254 nm, t_R(minor) = 8.8 min, t_R(major) = 10.5 min, 96% ee.

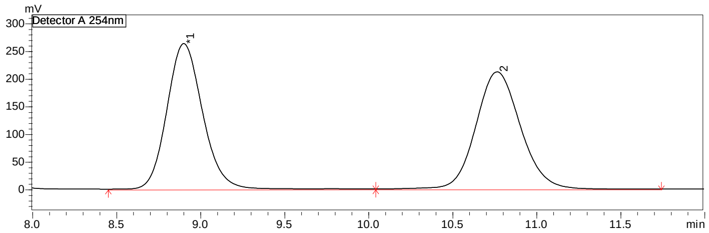

Peak#	Ret. Time	Area%
1	8.903	49.804
2	10.769	50.196

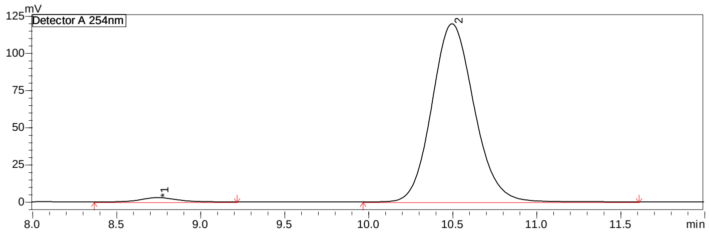

Peak#	Ret. Time	Area%
1	8.750	1.884
2	10.500	98.116

methyl (*R*)-2-((((9*H*-fluoren-9-yl)methoxy)carbonyl)amino)-2-(2-(methylthio)ethyl)hex-4-ynoate (**3de**)

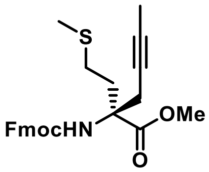

Procedure E. Purification by flash column chromatography (PE:acetone = 50:1) afforded the product as a yellow oil (63.0 mg, 72% yield).

^{1}H NMR (400 MHz, $CDCl_3$) δ 7.77 (d, *J* = 7.5 Hz, 2H), 7.62 (dd, *J* = 7.0, 3.0 Hz, 2H), 7.41 (t, *J* = 7.4 Hz, 2H), 7.32 (tt, *J* = 7.5, 1.2 Hz, 2H), 5.96 (s, 1H), 4.53 – 4.29 (m, 2H), 4.25 (t, *J* = 7.0 Hz, 1H), 3.82 (s, 3H), 3.16 (d, *J* = 16.8 Hz, 1H), 2.76 – 2.52 (m, 2H), 2.50 – 2.37 (m, 1H), 2.34 – 2.20 (m, 1H), 2.18 – 1.93 (m, 4H), 1.75 (s, 3H) ppm.

^{13}C{^{1}H} NMR (101 MHz, $CDCl_3$) δ 172.5, 154.2, 143.9, 143.7, 141.3, 127.6, 127.0, 125.0, 119.9, 78.8, 73.1, 66.4, 62.8, 53.0, 47.2, 34.4, 28.7, 26.5, 15.5, 3.5 ppm.

HRMS (ESI-TOF) m/z: $[M + H]^+$ Calcd for $C_{25}H_{28}NO_4S^+$ 438.1734; Found 438.1734.

IR (film): ν_{max} (cm^{-1}) 3417, 2952, 2918, 1723, 1500, 1448, 1333, 1273, 1224, 1106, 1050, 759, 741.

Optical rotation: $[\alpha]_D^{25}$ = -11.47 (*c* = 0.855, $CHCl_3$, 95% ee).

HPLC: DAICEL CHIRALPAK ID, hexane/*i*-PrOH = 90/10, flow rate: 1.0 mL/min, λ = 254 nm, t_R(major) = 15.0 min, t_R(minor) = 26.8 min, 95% ee.

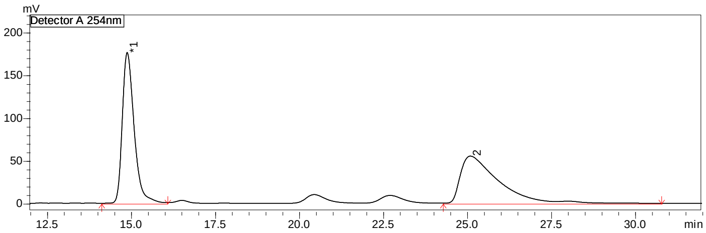

Peak#	Ret. Time	Area%
1	14.894	49.978
2	25.108	50.022

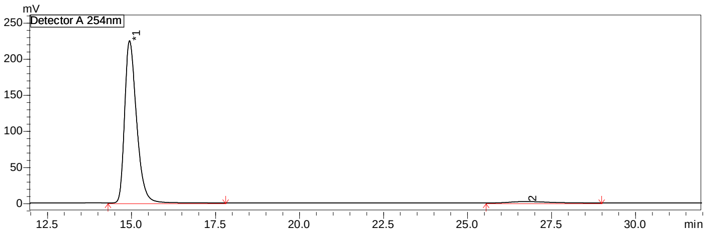

Peak#	Ret. Time	Area%
1	14.964	97.422
2	26.756	2.578

methyl (*R*)-2-((((9*H*-fluoren-9-yl)methoxy)carbonyl)amino)-2-benzyl-4-(methylthio)butanoate (**3dg**)

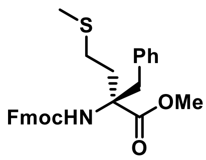

Procedure E. Purification by flash column chromatography (PE:acetone = 50:1) afforded the product as a colorless oil (79.4 mg, 83% yield).

^{1}H NMR (400 MHz, $CDCl_3$) δ 7.78 (d, *J* = 7.5 Hz, 2H), 7.59 (dd, *J* = 12.9, 7.5 Hz, 2H), 7.46 – 7.37 (m, 2H), 7.36 – 7.28 (m, 2H), 7.24 – 7.12 (m, 3H), 7.04 – 6.85 (m, 2H), 5.72 (s, 1H), 4.55 (dd, *J* = 10.6, 6.7 Hz, 1H), 4.38 (dd, *J* = 10.5, 7.1 Hz, 1H), 4.25 (t, *J* = 6.6 Hz, 1H), 3.77 (s, 3H), 3.62 (d, *J* = 13.5 Hz, 1H), 3.05 (d, *J* = 13.5 Hz, 1H), 2.94 – 2.81 (m, 1H), 2.50 – 2.39 (m, 1H), 2.29 – 2.16 (m, 2H), 2.06 (s, 3H) ppm.

^{13}C{^{1}H} NMR (101 MHz, $CDCl_3$) δ 172.8, 154.1, 143.9, 143.7, 141.3, 135.6, 129.5, 128.3, 127.7, 127.0, 127.0, 127.0, 125.1, 125.0, 120.0, 66.3, 64.8, 52.7, 47.3, 41.3, 34.9, 28.8, 15.5 ppm.

HRMS (ESI-TOF) m/z: $[M + H]^+$ Calcd for $C_{28}H_{30}NO_4S^+$ 476.1890; Found 476.1890.

IR (film): ν_{max} (cm^{-1}) 3416, 3030, 2950, 1720, 1497, 1449, 1318, 1271, 1216, 1079, 1044, 741, 703, 540.

Optical rotation: $[\alpha]_D^{25}$ = +11.10 (*c* = 0.880, $CHCl_3$, 96% ee).

HPLC: DAICEL CHIRALPAK IC, hexane/*i*-PrOH = 90/10, flow rate: 1.0 mL/min, λ = 254 nm, t_R(minor) = 12.9 min, t_R(major) = 21.8 min, 96% ee.

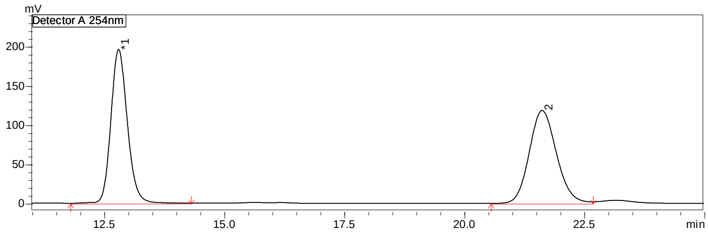

Peak#	Ret. Time	Area%
1	12.805	50.042
2	21.627	49.958

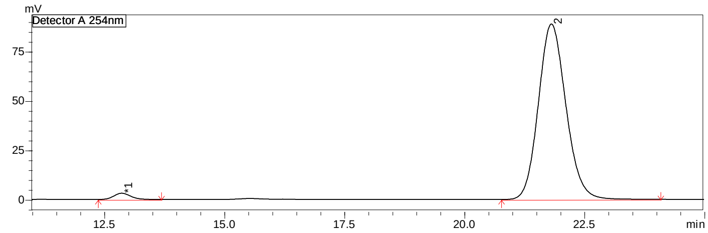

Peak#	Ret. Time	Area%
1	12.871	2.071
2	21.821	97.929

methyl (*S*)-4-allyl-2-phenyl-4,5-dihydrothiazole-4-carboxylate (**3ea**)

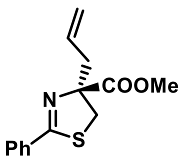

Procedure H. Purification by flash column chromatography (PE:EA = 100:1) afforded the product as a colorless oil (32.8mg, 63% yield).

^{1}H NMR (400 MHz, $CDCl_3$) δ 7.86 (d, *J* = 7.7 Hz, 2H), 7.48 (t, *J* = 7.3 Hz, 1H), 7.40 (t, *J* = 7.6 Hz, 2H), 5.89 – 5.71 (m, 1H), 5.27 – 5.11 (m, 2H), 3.88 (d, *J* = 11.5 Hz, 1H), 3.81 (s, 3H), 3.40 (d, *J* = 11.5 Hz, 1H), 2.83 – 2.71 (m, 2H) ppm.

The spectroscopic data match the literature.[3]

HPLC: DAICEL CHIRALPAK IG-3, hexane/*i*-PrOH = 98/2, flow rate: 1.0 mL/min, λ = 254 nm, t_R(major) = 7.9 min, t_R(minor) = 8.6 min, 84% ee.

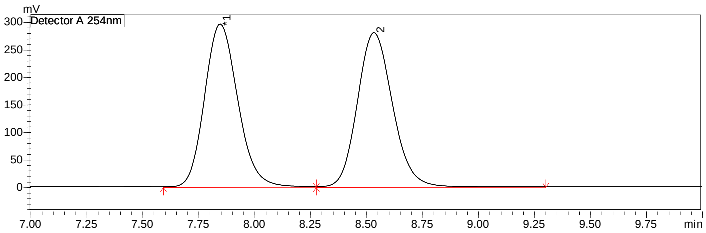

Peak#	Ret. Time	Area%
1	7.848	49.975
2	8.535	50.025

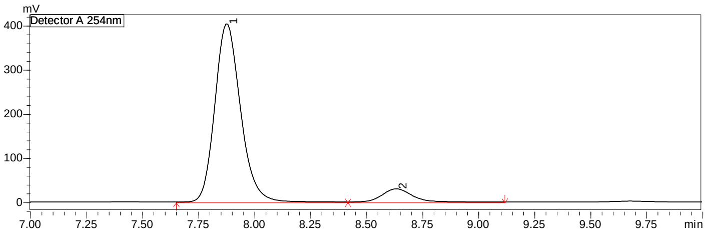

Peak#	Ret. Time	Area%
1	7.878	92.241
2	8.635	7.759

methyl (*S*)-4-(2-methylallyl)-2-phenyl-4,5-dihydrothiazole-4-carboxylate (**3ed**)

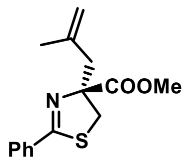

Procedure H. Purification by flash column chromatography (PE:EA = 20:1) afforded the product as a yellow oil (39.4 mg, 72% yield).

^{1}H NMR (400 MHz, $CDCl_3$) δ 7.90 – 7.81 (m, 2H), 7.50 – 7.44 (m, 1H), 7.44 – 7.37 (m, 2H), 4.95 – 4.90 (m, 1H), 4.84 – 4.79 (m, 1H), 3.91 (d, *J* = 11.5 Hz, 1H), 3.80 (s, 3H), 3.49 (d, *J* = 11.5 Hz, 1H), 2.85 – 2.72 (m, 2H), 1.79 (s, 3H) ppm.

The spectroscopic data match the literature.[3]

HPLC: DAICEL CHIRALPAK IC, hexane/*i*-PrOH = 98/2, flow rate: 1.0 mL/min, λ = 254 nm, t_R(minor) = 7.0 min, t_R(major) = 9.2 min, 83% ee.

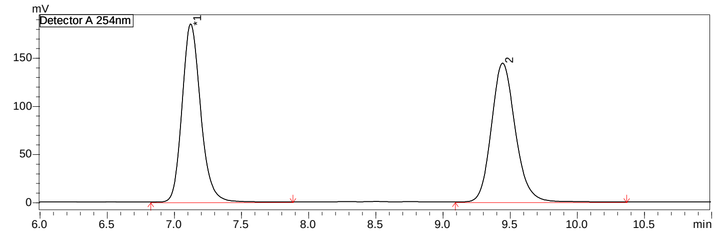

Peak#	Ret. Time	Area%
1	7.127	49.849
2	9.450	50.151

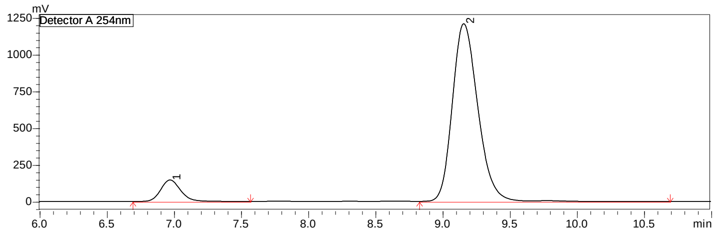

Peak#	Ret. Time	Area%
1	6.973	8.551
2	9.160	91.449

methyl (*S*)-4-(but-2-yn-1-yl)-2-phenyl-4,5-dihydrothiazole-4-carboxylate (**3ee**)

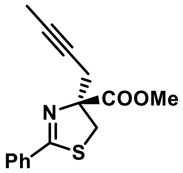

Procedure H. Purification by flash column chromatography (PE:EA = 20:1) afforded the product as a colorless oil (42.5 mg, 79% yield).

^{1}H NMR (400 MHz, $CDCl_3$) δ 7.84 (d, *J* = 7.5 Hz, 2H), 7.47 (t, *J* = 7.3 Hz, 1H), 7.40 (t, *J* = 7.5 Hz, 2H), 4.02 (d, *J* = 11.6 Hz, 1H), 3.84 (s, 3H), 3.62 (d, *J* = 11.6 Hz, 1H), 2.96 (dq, *J* = 16.4, 2.5 Hz, 1H), 2.73 (dq, *J* = 16.4, 2.5 Hz, 1H), 1.77 (t, *J* = 2.3 Hz, 3H) ppm.

$^{13}C\{^{1}H\}$ NMR (101 MHz, $CDCl_3$) δ 171.7, 169.9, 132.7, 131.6, 128.5, 128.4, 87.8, 79.0, 73.3, 53.1, 38.4, 27.6, 3.6 ppm.

HRMS (ESI-TOF) m/z: $[M + H]^+$ Calcd for $C_{15}H_{16}NO_2S^+$ 274.0896; Found 274.0897.

IR (film): ν_{max} (cm^{-1}) 2952, 2920, 1739, 1594, 1577, 1491, 1447, 1315, 1229, 1179, 1102, 1065, 946, 816, 767, 691, 622.

Optical rotation: $[\alpha]_D^{25}$ = -97.82 (*c* = 0.910, $CHCl_3$, 95% ee).

HPLC: DAICEL CHIRALPAK IG-3, hexane/*i*-PrOH = 98/2, flow rate: 1.0 mL/min, λ = 254 nm, t_R(major) = 14.9 min, t_R(minor) = 20.6 min, 95% ee.

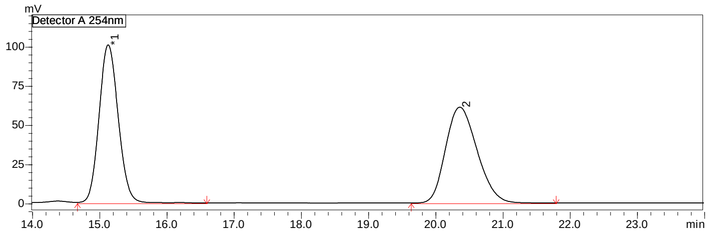

Peak#	Ret. Time	Area%
1	15.136	50.154
2	20.368	49.846

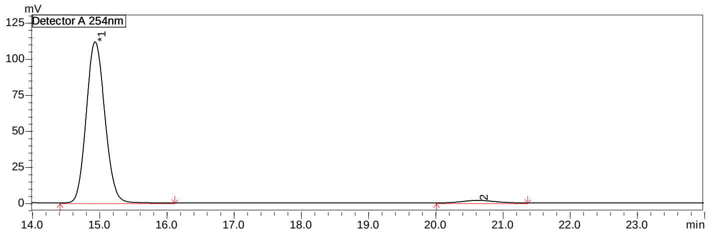

Peak#	Ret. Time	Area%
1	14.942	97.534
2	20.630	2.466

methyl (*S*)-4-benzyl-2-phenyl-4,5-dihydrothiazole-4-carboxylate (**3eg**)

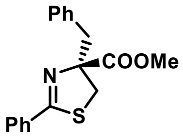

Procedure H. Purification by flash column chromatography (PE:EA = 20:1) afforded the product as a colorless oil (60.1 mg, 96% yield).

^{1}H NMR (400 MHz, $CDCl_3$) δ 7.85 (d, *J* = 7.5 Hz, 2H), 7.48 (t, *J* = 7.2 Hz, 1H), 7.40 (t, *J* = 7.5 Hz, 2H), 7.32 – 7.16 (m, 5H), 3.82 (d, *J* = 11.6 Hz, 1H), 3.78 (s, 3H), 3.44 (d, *J* = 11.6 Hz, 1H), 3.37 – 3.26 (m, 2H) ppm.

The spectroscopic data match the literature.[3]

HPLC: DAICEL CHIRALPAK IG-3, hexane/*i*-PrOH = 98/2, flow rate: 1.0 mL/min, λ = 254 nm, t_R(major) = 12.4 min, t_R(minor) = 14.3 min, 96% ee.

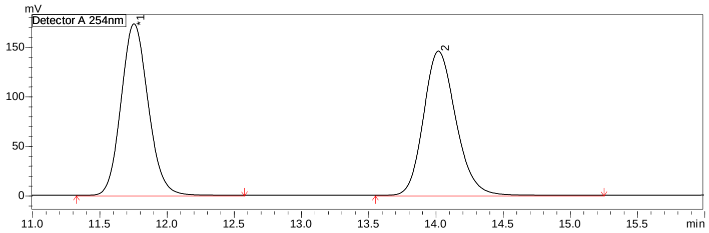

Peak#	Ret. Time	Area%
1	11.759	49.986
2	14.024	50.014

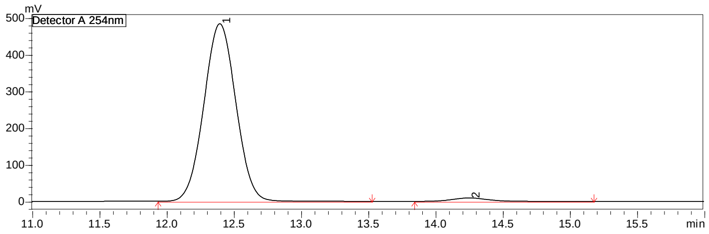

Peak#	Ret. Time	Area%
1	12.397	98.009
2	14.252	1.991

methyl (*S*)-4-(2-oxo-2-phenylethyl)-2-phenyl-4,5-dihydrothiazole-4-carboxylate (**3eu**)

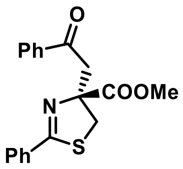

Procedure H. Purification by flash column chromatography (PE:EA = 40:1) afforded the product as a colorless oil (59.3 mg, 87% yield).

^{1}H NMR (400 MHz, $CDCl_3$) δ 7.97 (d, *J* = 7.9 Hz, 2H), 7.84 (d, *J* = 7.7 Hz, 2H), 7.58 (t, *J* = 7.4 Hz, 1H), 7.51 – 7.43 (m, 3H), 7.39 (t, *J* = 7.5 Hz, 2H), 4.38 (d, *J* = 11.9 Hz, 1H), 4.10 (d, *J* = 17.9 Hz, 1H), 3.79 (s, 3H), 3.55 – 3.40 (m, 2H) ppm.

^{13}C{^{1}H} NMR (101 MHz, $CDCl_3$) δ 197.0, 171.2, 170.3, 136.1, 133.5, 132.5, 131.6, 128.6, 128.5, 128.4, 128.0, 85.7, 52.9, 44.9, 39.2 ppm.

HRMS (ESI-TOF) m/z: [M + H]$^+$ Calcd for $C_{19}H_{18}NO_3S^+$ 340.1002; Found 340.1002.

IR (film): v_{max} (cm^{-1}) 3060, 2951, 1742, 1683, 1596, 1577, 1491, 1448, 1401, 1350, 1301, 1224, 1061, 1000, 948, 881, 813, 757, 689, 628, 607, 579.

Optical rotation: $[\alpha]_D^{25}$ = -209.64 (*c* = 0.880, $CHCl_3$, 93% ee).

HPLC: DAICEL CHIRALPAK ID, hexane/*i*-PrOH = 90/10, flow rate: 1.0 mL/min, λ = 254 nm, t_R(minor) = 17.5 min, t_R(major) = 18.8 min, 93% ee.

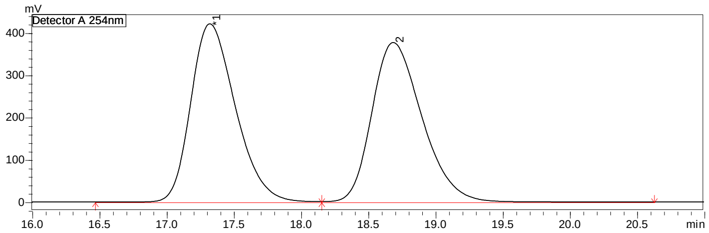

Peak#	Ret. Time	Area%
1	17.323	49.890
2	18.688	50.110

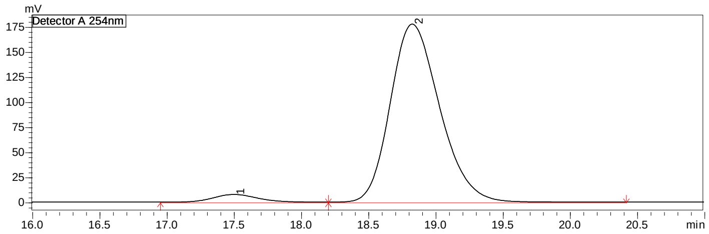

Peak#	Ret. Time	Area%
1	17.503	3.697
2	18.829	96.303

methyl (*S*)-4-(2-ethoxy-2-oxoethyl)-2-phenyl-4,5-dihydrothiazole-4-carboxylate (**3ev**)

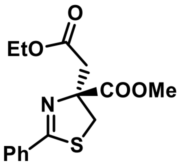

Procedure H. Purification by flash column chromatography (PE:EA = 40:1) afforded the product as a colorless oil (55.6 mg, 90% yield).

^{1}H NMR (400 MHz, $CDCl_3$) δ 7.89 – 7.80 (m, 2H), 7.51 – 7.45 (m, 1H), 7.44 – 7.36 (m, 2H), 4.16 (q, *J* = 7.1 Hz, 2H), 4.11 (d, *J* = 11.8 Hz, 1H), 3.82 (s, 3H), 3.54 (d, *J* = 11.8 Hz, 1H), 3.22 (d, *J* = 16.2 Hz, 1H), 2.89 (d, *J* = 16.2 Hz, 1H), 1.26 (t, *J* = 7.1 Hz, 3H) ppm.

^{13}C{^{1}H} NMR (101 MHz, $CDCl_3$) δ 171.4, 170.4, 170.0, 132.5, 131.7, 128.6, 128.4, 85.5, 60.9, 53.0, 40.6, 39.3, 14.1 ppm.

HRMS (ESI-TOF) m/z: $[M + H]^+$ Calcd for $C_{15}H_{18}NO_4S^+$ 308.0951; Found 308.0952.

IR (film): ν_{max} (cm^{-1}) 3062, 2958, 1733, 1594, 1575, 1491, 1447, 1372, 1260, 1067, 1023, 947, 795, 767, 690, 609.

Optical rotation: $[\alpha]_D^{25}$ = -109.70 (*c* = 1.040, $CHCl_3$, 94% ee).

HPLC: DAICEL CHIRALPAK IC, hexane/*i*-PrOH = 90/10, flow rate: 1.0 mL/min, λ = 254 nm, t_R(minor) = 11.5 min, t_R(major) = 15.7 min, 94% ee.

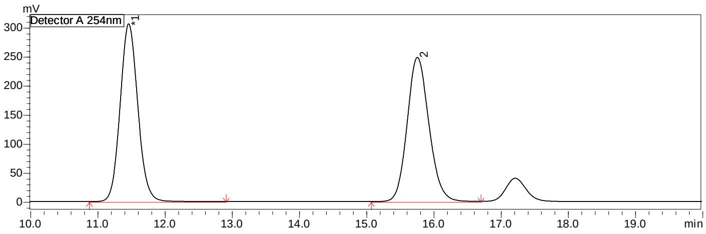

Peak#	Ret. Time	Area%
1	11.469	49.962
2	15.765	50.038

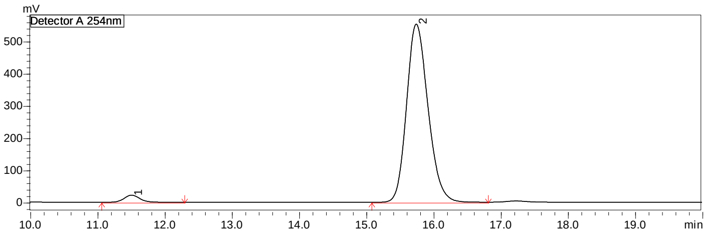

Peak#	Ret. Time	Area%
1	11.513	2.809
2	15.748	97.191

1-ethyl 4-methyl (*R*)-4-allyl-2-phenyl-4,5-dihydro-1*H*-imidazole-1,4-dicarboxylate (**3fa**)

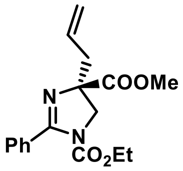

Procedure H. Purification by flash column chromatography (PE:EA = 5:1) afforded the product as a yellow oil (58.4 mg, 92% yield).

^{1}H NMR (400 MHz, $CDCl_3$) δ 7.55 (d, *J* = 7.1 Hz, 2H), 7.47 – 7.31 (m, 3H), 5.72 (ddt, *J* = 17.3, 10.1, 7.3 Hz, 1H), 5.29 – 5.10 (m, 2H), 4.39 (d, *J* = 11.3 Hz, 1H), 4.09 (q, *J* = 7.0 Hz, 2H), 3.95 (d, *J* = 11.3 Hz, 1H), 3.81 (s, 3H), 2.77 – 2.59 (m, 2H), 1.13 (t, *J* = 7.1 Hz, 3H) ppm.

^{13}C{^{1}H} NMR (101 MHz, $CDCl_3$) δ 172.6, 160.1, 151.3, 131.0, 130.7, 130.2, 128.5, 127.4, 120.1, 74.8, 62.2, 53.0, 52.8, 42.2, 14.0 ppm.

HRMS (ESI-TOF) m/z: $[M + H]^+$ Calcd for $C_{17}H_{21}N_2O_4^+$ 317.1496; Found 317.1496.

IR (film): ν_{max} (cm^{-1}) 2981, 2955, 1736, 1623, 1599, 1447, 1399, 1377, 1321, 1298, 1262, 1216, 1176, 1140, 1089, 1019, 766, 697.

Optical rotation: $[\alpha]_D^{25}$ = -67.88 (*c* = 0.955, $CHCl_3$, 99% ee).

HPLC: DAICEL CHIRALPAK IG-3, hexane/*i*-PrOH = 40/10, flow rate: 0.5 mL/min, λ = 254 nm, t_R(minor) = 14.4 min, t_R(major) = 15.8 min, 99% ee.

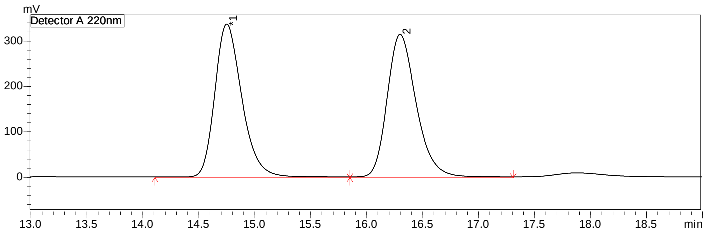

Peak#	Ret. Time	Area%
1	14.756	49.980
2	16.304	50.020

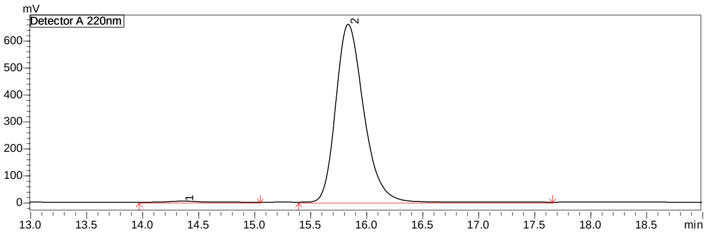

Peak#	Ret. Time	Area%
1	14.364	0.577
2	15.841	99.423

1-ethyl 4-methyl (*R*)-4-(but-2-yn-1-yl)-2-phenyl-4,5-dihydro-1*H*-imidazole-1,4-dicarboxylate (**3fe**)

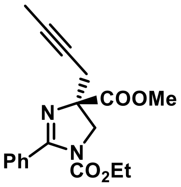

Procedure H. Purification by flash column chromatography (PE:EA = 5:1) afforded the product as a yellow oil (51.8 mg, 79% yield).

^{1}H NMR (400 MHz, $CDCl_3$) δ 7.60 – 7.51 (m, 2H), 7.47 – 7.41 (m, 1H), 7.40 – 7.32 (m, 2H), 4.48 (d, *J* = 11.3 Hz, 1H), 4.18 – 4.05 (m, 3H), 3.81 (s, 3H), 2.86 (dq, *J* = 16.5, 2.5 Hz, 1H), 2.70 (dq, *J* = 16.5, 2.5 Hz, 1H), 1.74 (t, *J* = 2.4 Hz, 3H), 1.14 (t, *J* = 7.1 Hz, 3H) ppm.

^{13}C{^{1}H} NMR (101 MHz, $CDCl_3$) δ 171.7, 160.8, 151.4, 130.8, 130.2, 128.5, 127.5, 78.5, 74.5, 72.6, 62.2, 53.5, 53.0, 28.6, 14.0, 3.4 ppm.

HRMS (ESI-TOF) m/z: [M + H]$^+$ Calcd for $C_{18}H_{21}N_2O_4^+$ 329.1496; Found 329.1496.

IR (film): v_{max} (cm^{-1}) 2955, 2920, 1735, 1623, 1598, 1447, 1399, 1376, 1297, 1263, 1214, 1176, 1138, 1092, 1019, 971, 766, 697.

Optical rotation: $[\alpha]_D^{25}$ = -95.43 (*c* = 0.960, $CHCl_3$, 98% ee).

HPLC: DAICEL CHIRALPAK IC, hexane/*i*-PrOH = 80/20, flow rate: 1.0 mL/min, λ = 254 nm, t_R(minor) = 12.4 min, t_R(major) = 20.7 min, 98% ee.

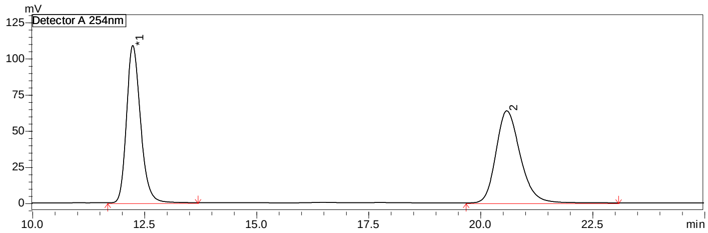

Peak#	Ret. Time	Area%
1	12.251	49.791
2	20.599	50.209

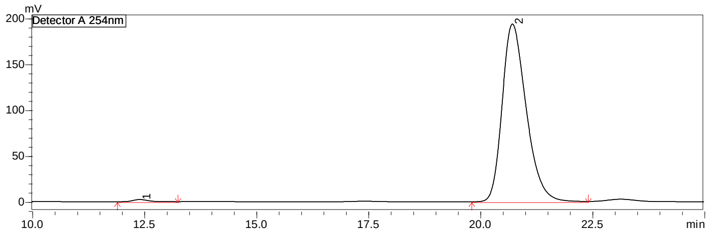

Peak#	Ret. Time	Area%
1	12.409	0.794
2	20.723	99.206

1-ethyl 4-methyl (*R*)-4-benzyl-2-phenyl-4,5-dihydro-1*H*-imidazole-1,4-dicarboxylate (**3fg**)

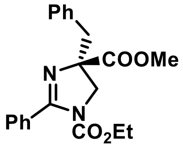

Procedure H. Purification by flash column chromatography (PE:EA = 5:1) afforded the product as a yellow oil (70.6 mg, 96% yield).

^{1}H NMR (400 MHz, $CDCl_3$) δ 7.52 – 7.45 (m, 2H), 7.44 – 7.38 (m, 1H), 7.37 – 7.31 (m, 2H), 7.30 – 7.18 (m, 5H), 4.30 (d, *J* = 11.4 Hz, 1H), 4.08 (d, *J* = 11.4 Hz, 1H), 3.95 – 3.77 (m, 5H), 3.30 (d, *J* = 13.6 Hz, 1H), 3.15 (d, *J* = 13.5 Hz, 1H), 0.98 (t, *J* = 7.1 Hz, 3H) ppm.

^{13}C{^{1}H} NMR (101 MHz, $CDCl_3$) δ 172.9, 160.2, 150.8, 134.3, 130.7, 130.2, 130.1, 128.4, 128.0, 127.3, 127.0, 75.5, 61.9, 52.8, 52.5, 43.2, 13.8 ppm.

HRMS (ESI-TOF) m/z: $[M + H]^+$ Calcd for $C_{21}H_{23}N_2O_4^+$ 367.1652; Found 367.1652.

IR (film): ν_{max} (cm^{-1}) 2954, 1735, 1620, 1598, 1495, 1447, 1399, 1376, 1322, 1294, 1260, 1214, 1141, 1092, 1019, 969, 765, 698.

Optical rotation: $[\alpha]_D^{25}$ = -80.10 (*c* = 1.030, $CHCl_3$, 99% ee).

HPLC: DAICEL CHIRALPAK IG-3, hexane/*i*-PrOH = 40/10, flow rate: 0.5 mL/min, λ = 254 nm, t_R(minor) = 18.4 min, t_R(major) = 22.0 min, 99% ee.

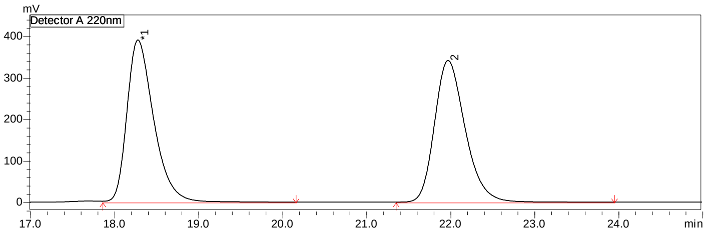

Peak#	Ret. Time	Area%
1	18.285	50.060
2	21.977	49.940

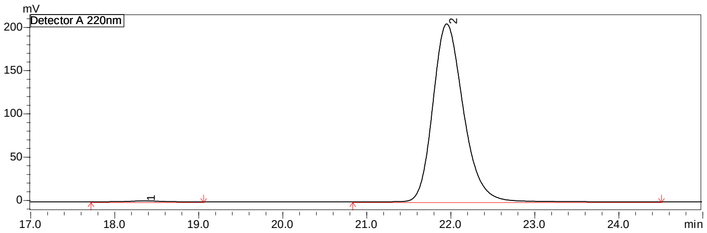

Peak#	Ret. Time	Area%
1	18.362	0.508
2	21.960	99.492

1-(*tert*-butyl) 4-methyl (*R*)-4-allyl-2-phenyl-4,5-dihydro-1*H*-imidazole-1,4-dicarboxylate (**3ga**)

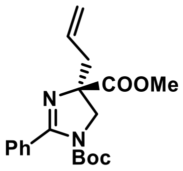

Procedure H. Purification by flash column chromatography (PE:EA = 5:1) afforded the product as a colorless oil (65.3 mg, 95% yield).

^{1}H NMR (400 MHz, $CDCl_3$) δ 7.52 (d, *J* = 7.1 Hz, 2H), 7.45 – 7.31 (m, 3H), 5.85 – 5.55 (m, 1H), 5.31 – 5.06 (m, 2H), 4.33 (d, *J* = 11.3 Hz, 1H), 3.93 (d, *J* = 11.3 Hz, 1H), 3.79 (s, 3H), 2.67 (d, *J* = 7.0 Hz, 2H), 1.25 (s, 9H) ppm.

^{13}C{^{1}H} NMR (126 MHz, $CDCl_3$) δ 172.7, 160.4, 150.2, 131.5, 131.1, 129.9, 128.3, 127.5, 119.9, 82.1, 74.5, 53.2, 52.7, 42.1, 27.6 ppm.

HRMS (ESI-TOF) m/z: [M + H]$^+$ Calcd for $C_{19}H_{25}N_2O_4^+$ 345.1809; Found 345.1809.

IR (film): ν_{max} (cm^{-1}) 2979, 1735, 1624, 1598, 1476, 1448, 1367, 1259, 1217, 1141, 1087, 1029, 1001, 968, 922, 856, 841, 769, 697.

Optical rotation: $[\alpha]_D^{25}$ = -58.22 (*c* = 1.130, $CHCl_3$, 99% ee).

HPLC: DAICEL CHIRALPAK IG-3, hexane/*i*-PrOH = 40/10, flow rate: 0.5 mL/min, λ = 254 nm, t_R(minor) = 11.1 min, t_R(major) = 13.1 min, 99% ee.

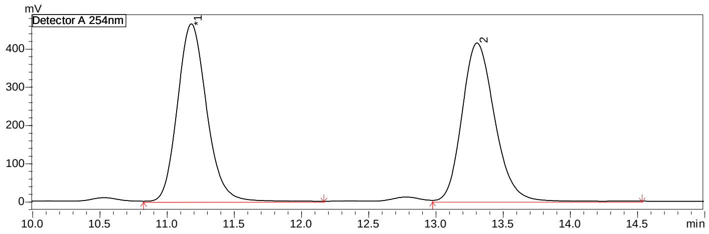

Peak#	Ret. Time	Area%
1	11.186	50.069
2	13.311	49.931

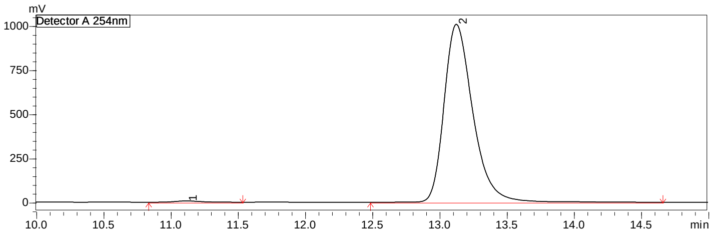

Peak#	Ret. Time	Area%
1	11.119	0.722
2	13.127	99.278

1-(*tert*-butyl) 4-methyl (*R*)-4-(2-iodobenzyl)-2-phenyl-4,5-dihydro-1*H*-imidazole-1,4-dicarboxylate (**3gd'**)

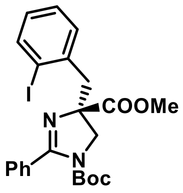

Procedure H. Purification by flash column chromatography (PE:EA = 5:1) afforded the product as a colorless oil (102.6 mg, 99% yield).

^{1}H NMR (400 MHz, $CDCl_3$) δ 7.84 (dd, *J* = 7.9, 1.2 Hz, 1H), 7.54 – 7.47 (m, 2H), 7.44 – 7.30 (m, 4H), 7.24 (td, *J* = 7.5, 1.3 Hz, 1H), 6.89 (td, *J* = 7.8, 1.5 Hz, 1H), 4.29 (d, *J* = 11.7 Hz, 1H), 4.15 (d, *J* = 11.7 Hz, 1H), 3.84 (s, 3H), 3.56 – 3.42 (m, 2H), 1.17 (s, 9H) ppm.

^{13}C{^{1}H} NMR (126 MHz, $CDCl_3$) δ 172.8, 160.7, 149.6, 139.7, 138.1, 131.2, 131.1, 130.0, 128.8, 128.3, 127.8, 127.4, 102.6, 81.8, 75.2, 53.0, 52.6, 46.0, 27.6 ppm.

HRMS (ESI-TOF) m/z: $[M + H]^+$ Calcd for $C_{23}H_{26}IN_2O_4^+$ 521.0932; Found 521.0932.

IR (film): v_{max} (cm^{-1}) 2976, 2929, 1733, 1621, 1598, 1464, 1436, 1367, 1258, 1142, 1083, 1011, 971, 842, 766, 696.

Optical rotation: $[\alpha]_D^{25}$ = -116.07 (*c* = 1.055, $CHCl_3$, 99% ee).

HPLC: DAICEL CHIRALPAK ID, hexane/*i*-PrOH = 90/10, flow rate: 1.0 mL/min, λ = 254 nm, t_R(minor) = 10.5 min, t_R(major) = 13.3 min, 99% ee.

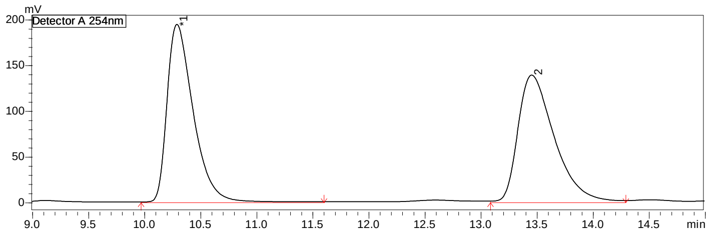

Peak#	Ret. Time	Area%
1	10.294	50.113
2	13.458	49.887

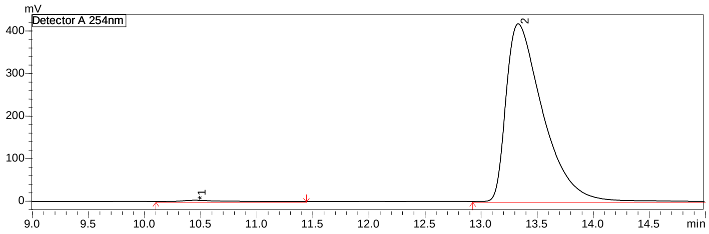

Peak#	Ret. Time	Area%
1	10.456	0.597
2	13.337	99.403

1-(*tert*-butyl) 4-methyl (*R*)-4-(2-oxo-2-(thiophen-2-yl)ethyl)-2-phenyl-4,5-dihydro-1*H*-imidazole-1,4-dicarboxylate (**3ge'**)

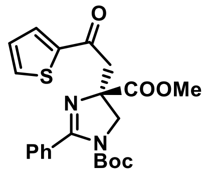

Procedure H. Purification by flash column chromatography (PE:acetone = 20:1) afforded the product as a white solid (74.5 mg, 87% yield, m.p. 129-131 ℃).

^{1}H NMR (400 MHz, $CDCl_3$) δ 7.76 (dd, *J* = 3.8, 0.9 Hz, 1H), 7.67 (dd, *J* = 4.9, 0.9 Hz, 1H), 7.54 – 7.48 (m, 2H), 7.45 – 7.33 (m, 3H), 7.15 (dd, *J* = 4.9, 3.9 Hz, 1H), 4.86 (d, *J* = 11.8 Hz, 1H), 4.14 (d, *J* = 17.6 Hz, 1H), 3.91 (d, *J* = 11.8 Hz, 1H), 3.76 (s, 3H), 3.33 (d, *J* = 17.6 Hz, 1H), 1.31 (s, 9H) ppm.

^{13}C{^{1}H} NMR (126 MHz, $CDCl_3$) δ 189.8, 171.2, 161.7, 150.3, 143.2, 134.2, 132.6, 131.2, 130.1, 128.3, 128.2, 127.6, 82.3, 72.5, 55.3, 53.0, 47.4, 27.7 ppm.

HRMS (ESI-TOF) m/z: $[M + H]^+$ Calcd for $C_{22}H_{25}N_2O_5S^+$ 429.1479; Found 429.1478.

IR (film): ν_{max} (cm^{-1}) 2955, 2928, 1740, 1660, 1617, 1597, 1447, 1416, 1368, 1319, 1234, 1141, 1086, 1059, 966, 858, 806, 734, 698.

Optical rotation: $[\alpha]_D^{25}$ = -175.58 (*c* = 0.900, $CHCl_3$, 94% ee).

HPLC: DAICEL CHIRALPAK IA, hexane/*i*-PrOH = 90/10, flow rate: 1.0 mL/min, λ = 254 nm, t_R(major) = 14.3 min, t_R(minor) = 19.3 min, 94% ee.

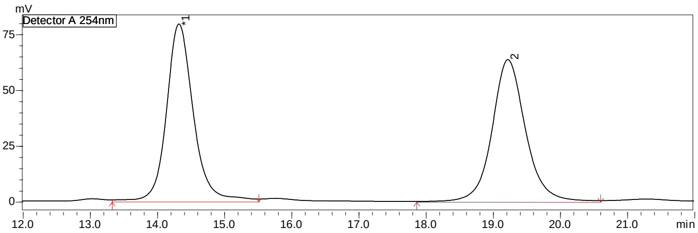

Peak#	Ret. Time	Area%
1	14.330	50.215
2	19.228	49.785

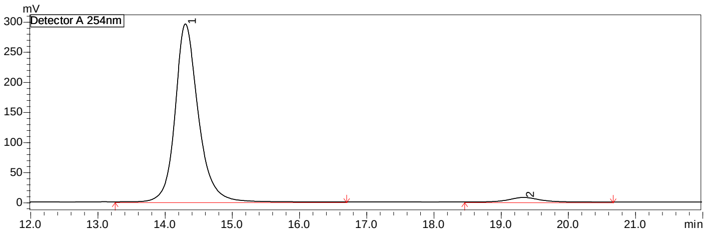

Peak#	Ret. Time	Area%
1	14.313	96.785
2	19.345	3.215

tert-butyl (*S*)-2-(but-2-yn-1-yl)-5-phenyl-3,4-dihydro-2*H*-pyrrole-2-carboxylate (**3he**)

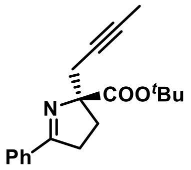

Procedure H. Purification by flash column chromatography (PE:EA = 20:1) afforded the product as a white solid (48.4mg, 81% yield, m.p. 44-47 ℃).

^{1}H NMR (500 MHz, $CDCl_3$) δ 7.92 – 7.83 (m, 2H), 7.48 – 7.36 (m, 3H), 3.17 – 3.02 (m, 2H), 2.91 (dq, *J* = 16.4, 2.4 Hz, 1H), 2.69 (dq, *J* = 15.9, 2.3 Hz, 1H), 2.46 (ddd, *J* = 13.3, 8.9, 6.2 Hz, 1H), 2.16 (ddd, *J* = 13.2, 9.3, 7.2 Hz, 1H), 1.71 (t, *J* = 2.5 Hz, 3H), 1.47 (s, 9H) ppm.

^{13}C{^{1}H} NMR (126 MHz, $CDCl_3$) δ 175.0, 172.1, 134.1, 130.7, 128.3, 128.0, 83.2, 81.2, 74.9, 36.2, 30.8, 28.5, 27.9, 3.5 ppm.

HRMS (ESI-TOF) m/z: $[M + H]^+$ Calcd for $C_{19}H_{24}NO_2^+$ 298.1802; Found 298.1803.

IR (film): ν_{max} 2976, 2920, 1728, 1614, 1576, 1392, 1368, 1343, 1256. 1158, 1069, 848, 762, 693.

Optical rotation: $[\alpha]_D^{25}$ = -101.23 (*c* = 1.020, $CHCl_3$, 98% ee).

HPLC: DAICEL CHIRALPAK ID, hexane/*i*-PrOH = 98/2, flow rate: 1.0 mL/min, λ = 254 nm, t_R(major) = 9.2 min, t_R(minor) = 10.5 min, 98% ee.

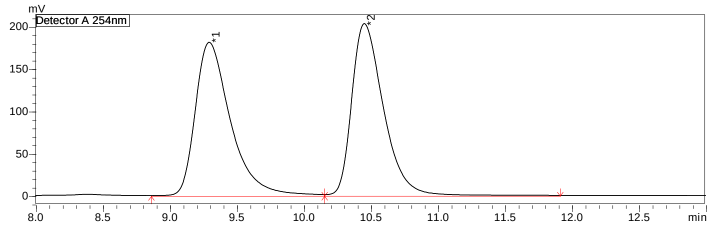

Peak#	Ret. Time	Area%
1	9.295	49.804
2	10.452	50.196

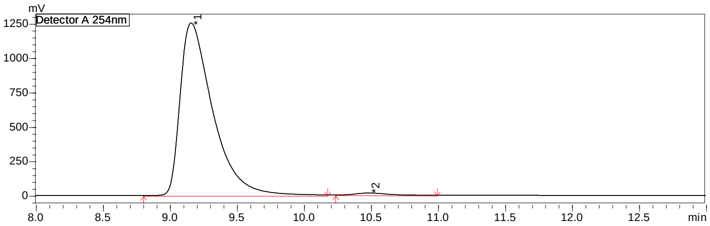

Peak#	Ret. Time	Area%
1	9.160	98.965
2	10.487	1.035

tert-butyl (*S*)-2-benzyl-5-phenyl-3,4-dihydro-2*H*-pyrrole-2-carboxylate (**3hg**)

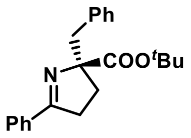

Procedure H. Purification by flash column chromatography (PE:EA = 20:1) afforded the product as a white solid (66.2 mg, 99% yield, m.p.101-103 ℃).

^{1}H NMR (500 MHz, $CDCl_3$) δ 7.80 (d, *J* = 6.9 Hz, 2H), 7.44 – 7.33 (m, 3H), 7.24 (d, *J* = 6.6 Hz, 2H), 7.21 – 7.11 (m, 3H), 3.35 (d, *J* = 13.6 Hz, 1H), 3.23 (d, *J* = 13.6 Hz, 1H), 2.91 – 2.76 (m, 1H), 2.40 – 2.22 (m, 2H), 2.04 (ddd, *J* = 12.5, 10.6, 5.5 Hz, 1H), 1.46 (s, 9H) ppm.

The spectroscopic data match the literature.[6]

HPLC: DAICEL CHIRALPAK ID, hexane/*i*-PrOH = 98/2, flow rate: 1.0 mL/min, λ = 254 nm, t_R(major) = 7.8 min, t_R(minor) = 9.7 min, 99% ee.

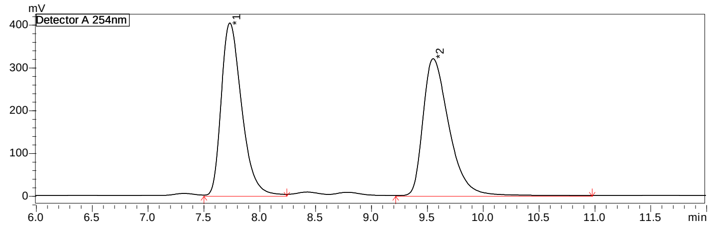

Peak#	Ret. Time	Area%
1	7.738	49.939
2	9.559	50.061

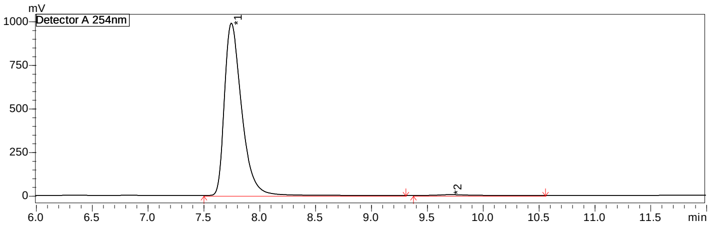

Peak#	Ret. Time	Area%
1	7.752	99.440
2	9.718	0.560

3ig[7] is a known compound.

2 Supplementary Discussion

2.1 Gram-Scale Reaction, Double Asymmetric Alkylation in One Pot, and Catalytic Asymmetric Alkylation of Chiral Dipeptides

2.1.1 Gram-Scale Reaction

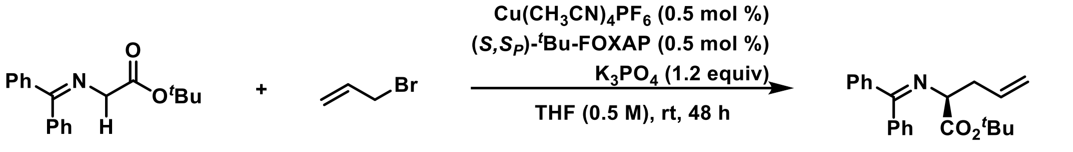

1a, 3.54 g, 12 mmol   **2a**, 1.21 g, 10 mmol   **3aa**, 2.92 g, 87%, 97% ee

A dried 100 mL Schlenk flask equipped with a magnetic stirring bar was charged with $[Cu(CH_3CN)_4]PF_6$ (18.6 mg, 0.05 mmol, 0.005 equiv), (S,S_P)-^{t}Bu-FOXAP (24.8 mg, 0.05 mmol, 0.005 equiv), *tert*-butyl 2-((diphenylmethylene)amino)acetate (**1a**, 3.54 g, 12 mmol, 1.2 equiv), and K_3PO_4 (2.55 g, 12 mmol, 1.2 equiv) in a glove box under Ar atmosphere. Anhydrous THF (20 mL) was added via a syringe. The mixture was stirred for 15 minutes to give an orange catalyst solution. Then allyl bromide (**2a**, 1.21 g, 10 mmol, 1.0 equiv) was added. The resulting reaction mixture was stirred at room temperature for 48 h. After the volatiles were removed under reduced pressure, the crude product was purified by silica gel column chromatography (PE:EA = 100:1) to give the desired product as a pale yellow oil (**3aa**, 2.92 g, 87% yield, 97% ee).

2.1.2 Double Asymmetric Alkylation in One Pot

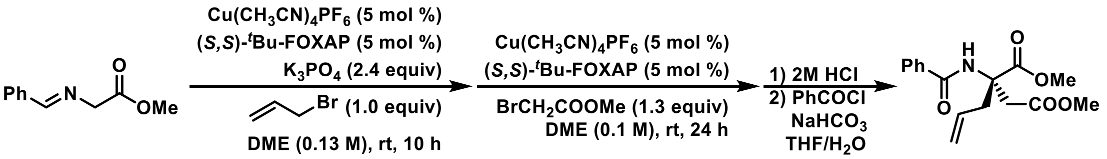

A dried 25 mL Schlenk tube equipped with a magnetic stirring bar was charged with $[Cu(CH_3CN)_4]PF_6$ (3.7 mg, 0.01 mmol, 0.05 equiv), (S,S_P)-^{t}Bu-FOXAP (5.0 mg, 0.01 mmol, 0.05 equiv), and K_3PO_4 (101.9 mg, 0.48 mmol, 2.4 equiv) in a glove box under Ar atmosphere. Anhydrous DME (1.5 mL) was added via a syringe. The mixture was stirred at room temperature for 15 minutes to give an orange catalyst solution. Then α-imino-ester **1a'** (35.4 mg, 0.2 mmol, 1.0 equiv) and allyl bromide **2a** (0.2 mmol, 1.0 equiv) were added. After the resulting reaction mixture was stirred at room temperature for 10 hours (TLC monitoring), a solution of $[Cu(CH_3CN)_4]PF_6$ (3.7 mg, 0.01 mmol, 0.05 equiv) and (S,S_P)-^{t}Bu-FOXAP (5.0 mg, 0.01 mmol, 0.05 equiv) in 0.5 mL DME which had been stirred for 15 minutes was added. Then $BrCH_2COOMe$ (39.8 mg, 0.26 mmol, 1.3 equiv) was added. After the reaction mixture was stirred at room temperature for 24 hours, the reaction mixture was extracted with ethyl ether (3x2 mL) and saturated NaCl solution (15 mL) to remove the base. The combined organic layers were evaporated *in vacuo*. The residue was dissolved in THF (2 mL) and hydrolyzed with 2 M HCl (0.15 mL, 0.3 mmol, 1.5 equiv) for 15 min (TLC monitoring). Then H_2O (1

mL), $NaHCO_3$ (168 mg, 2.0 mmol, 10 equiv), and PhCOCl (42.2 mg, 0.3 mmol, 1.5 equiv) were added sequentially. The resulting reaction mixture was stirred at room temperature for 30 minutes and extracted with ethyl ether (3x2 mL) and water (10 mL). The combined organic layers were then evaporated *in vacuo* and the crude product was purified by silica gel column chromatography to give the desired product as a colorless oil (**4**, 37.9 mg, 62%).

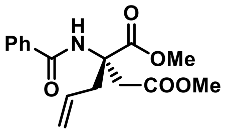

^{1}H NMR (500 MHz, $CDCl_3$) δ 7.81 – 7.74 (m, 2H), 7.54 – 7.35 (m, 4H), 5.62 (ddt, *J* = 17.4, 10.1, 7.4 Hz, 1H), 5.14 – 5.02 (m, 2H), 3.90 – 3.79 (m, 4H), 3.62 (s, 3H), 3.42 (dd, *J* = 13.8, 7.3 Hz, 1H), 3.03 (d, *J* = 16.7 Hz, 1H), 2.56 (dd, *J* = 13.8, 7.5 Hz, 1H) ppm.

The spectroscopic data match the literature.[25]

HPLC: DAICEL CHIRALPAK IBN-3, hexane/*i*-PrOH =40/10, flow rate: 0.5 mL/min, λ = 254 nm, t_R(minor) = 11.5 min, t_R(major) = 13.0 min, 95% ee.

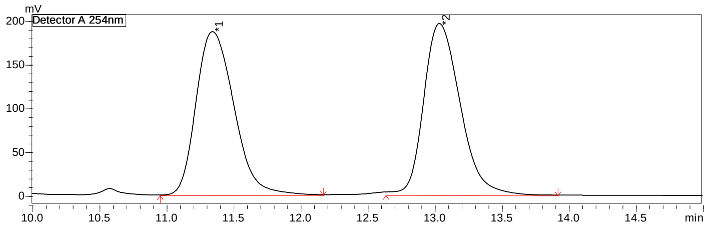

Peak#	Ret. Time	Area%
1	11.345	49.691
2	13.037	50.309

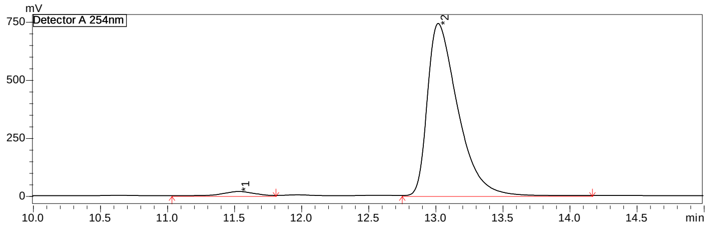

Peak#	Ret. Time	Area%
1	11.537	2.445
2	13.023	97.555

2.1.3 Catalytic Asymmetric Alkylation of Chiral Dipeptides

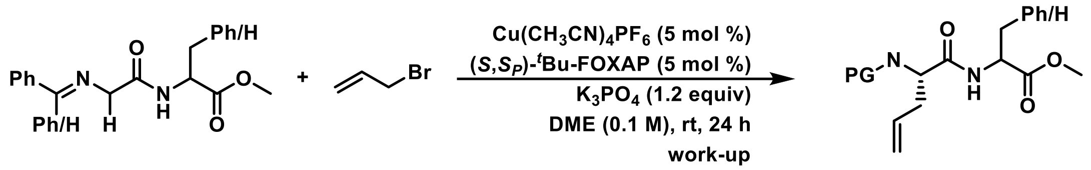

methyl ((*S*)-2-((diphenylmethylene)amino)pent-4-enoyl)-*D*-phenylalaninate (***(R,S)*-8**)

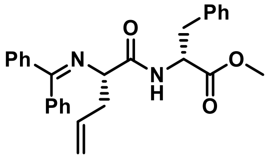

Procedure H. Purification by flash column chromatography (PE:EA = 5:1) afforded the desired product as a colorless oil (76.1 mg, 86% yield, >20:1 dr (Dr was determined by both ^{1}H NMR and HPLC analysis of ***(R,S)*-8** after column chromatography)).

^{1}H NMR (400 MHz, $CDCl_3$) δ 7.62 – 7.55 (m, 2H), 7.48 – 7.32 (m, 6H), 7.26 (d, *J* = 8.6 Hz, 1H), 7.14 – 7.07 (m, 1H), 7.07 – 6.98 (m, 4H), 6.96 – 6.87 (m, 2H), 5.64 (ddt, *J* = 17.4, 10.3, 7.2 Hz, 1H), 5.04 – 4.95 (m, 2H), 4.91 (ddd, *J* = 8.5, 7.2, 5.3 Hz, 1H), 3.97 (dd, *J* = 7.1, 5.0 Hz, 1H), 3.76 (s, 3H), 3.16 (dd, *J* = 13.9, 5.3 Hz, 1H), 3.05 (dd, *J* = 13.9, 7.2 Hz, 1H), 2.60 – 2.42 (m, 2H) ppm.

$^{13}C\{^1H\}$ NMR (101 MHz, $CDCl_3$) δ 172.4, 171.7, 169.5, 138.9, 135.7, 135.5, 134.0, 130.5, 129.0, 128.6, 128.5, 128.4, 128.0, 127.6, 126.9, 117.5, 65.5, 52.4, 52.2, 39.5, 37.7 ppm.

HRMS (ESI-TOF) m/z: $[M + H]^+$ Calcd for $C_{28}H_{29}N_2O_3^+$ 441.2173; Found 441.2173.

IR (film): ν_{max} (cm^{-1}) 3383, 3027, 2952, 2926, 1747, 1682, 1623, 1505, 1446, 1360, 1316, 1286, 1212, 1177, 1077, 1030, 1001, 915, 783, 699.

Optical rotation: $[\alpha]_D^{25}$ = +10.24 (*c* = 0.945, $CHCl_3$, >99% ee, >20:1 dr).

HPLC: DAICEL CHIRALPAK OX-3, hexane/*i*-PrOH = 40/10, flow rate: 0.5 mL/min, λ = 254 nm, t_R(minor) = 16.9 min, t_R(major) = 37.5 min, >99% ee, >20:1 dr.

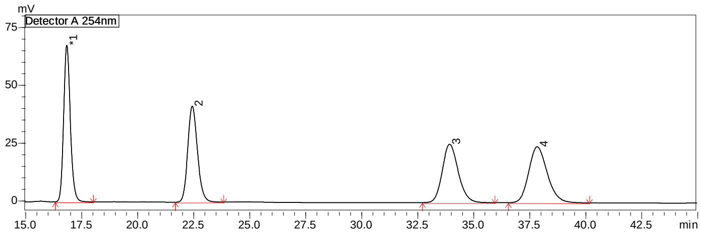

Peak#	Ret. Time	Area%
1	16.867	26.458
2	22.470	23.652
3	33.957	23.599
4	37.865	26.291

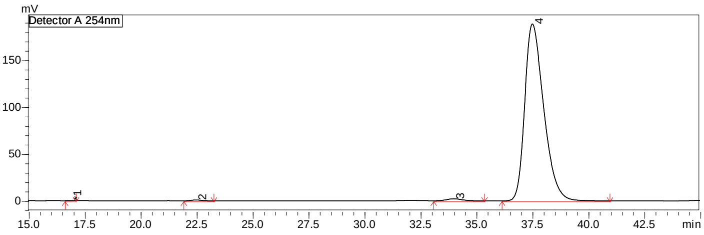

Peak#	Ret. Time	Area%
1	16.895	0.013
2	22.478	0.215
3	34.003	0.954
4	37.513	98.818

methyl ((*S*)-2-((diphenylmethylene)amino)pent-4-enoyl)-*L*-phenylalaninate (**(*S*,*S*)-8**)

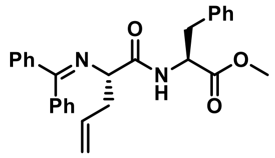

Procedure H. Purification by flash column chromatography (PE:EA = 5:1) afforded the desired product as a colorless oil (81.3 mg, 92% yield, >20:1 dr (Dr was determined by both ^{1}H NMR and HPLC analysis of **(*S*,*S*)-8** after column chromatography)).

^{1}H NMR (400 MHz, $CDCl_3$) δ 7.58 – 7.52 (m, 2H), 7.46 – 7.38 (m, 4H), 7.37 – 7.30 (m, 3H), 7.30 – 7.23 (m, 3H), 7.23 – 7.17 (m, 2H), 7.12 – 7.04 (m, 2H), 5.59 (ddt, *J* = 17.3, 10.1, 7.2 Hz, 1H), 5.03 – 4.94 (m, 2H), 4.91 (dt, *J* = 8.2, 6.0 Hz, 1H), 4.02 (t, *J* = 5.8 Hz, 1H), 3.69 (s, 3H), 3.16 (d, *J* = 6.0 Hz, 2H), 2.53 – 2.38 (m, 2H) ppm.

^{13}C{^{1}H} NMR (101 MHz, $CDCl_3$) δ 172.2, 171.7, 169.8, 139.0, 135.8, 135.6, 133.9, 130.5, 129.2, 128.7, 128.5, 128.0, 127.6, 127.0, 117.6, 65.3, 52.5, 52.1, 39.5, 37.9 ppm.

HRMS (ESI-TOF) m/z: $[M + H]^+$ Calcd for $C_{28}H_{29}N_2O_3^+$ 441.2173; Found 441.2172.

IR (film): ν_{max} (cm^{-1}) 3379, 3028, 2953, 1746, 1682, 1624, 1505, 1445, 1286, 1261, 1203, 1178, 1078, 1029, 915, 802, 784, 700.

Optical rotation: $[\alpha]_D^{25}$ = +45.64 (*c* = 0.935, $CHCl_3$, >99% ee, >20:1 dr).

HPLC: DAICEL CHIRALPAK OX-3, hexane/*i*-PrOH = 40/10, flow rate: 0.5 mL/min, λ = 254 nm, t_R(major) = 22.3 min, t_R(minor) = 34.1 min, >99% ee, >20:1 dr.

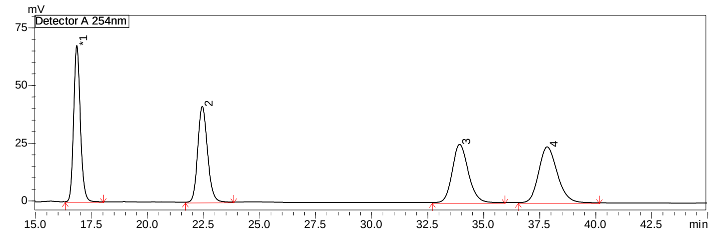

Peak#	Ret. Time	Area%
1	16.867	26.458
2	22.470	23.652
3	33.957	23.599
4	37.865	26.291

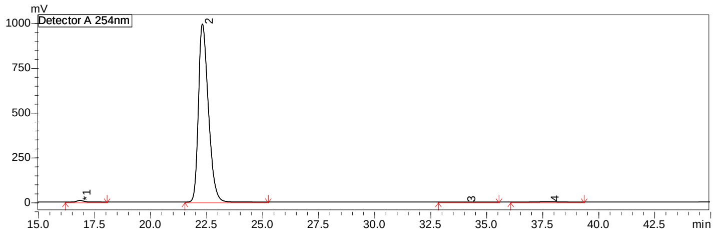

Peak#	Ret. Time	Area%
1	16.879	0.605
2	22.345	98.948
3	34.058	0.030
4	37.777	0.416

methyl ((*S*)-2-((((9*H*-fluoren-9-yl)methoxy)carbonyl)amino)-2-methylpent-4-enoyl)-*D*-alaninate ((***R*,*S***)-**10**)

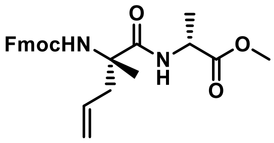

Procedure E. Purification by flash column chromatography (PE:EA = 5:1) afforded the desired product as a yellow oil (63.2 mg, 72% yield, >20:1 dr (Dr was determined by HPLC analysis of (***R*,*S***)-**10** after column chromatography)).

^{1}H NMR (400 MHz, $CDCl_3$) δ 7.76 (d, *J* = 7.5 Hz, 2H), 7.63 – 7.55 (m, 2H), 7.40 (t, *J* = 7.4 Hz, 2H), 7.31 (t, *J* = 7.4 Hz, 2H), 6.72 (s, 1H), 5.71 (ddt, *J* = 17.3, 10.0, 7.3 Hz, 1H), 5.52 (s, 1H), 5.22 – 5.05 (m, 2H), 4.62 – 4.49 (m, 1H), 4.47 – 4.31 (m, 2H), 4.21 (t, *J* = 6.8 Hz, 1H), 3.74 (s, 3H), 2.64 (d, *J* = 43.2 Hz, 2H), 1.54 (s, 3H), 1.38 (d, *J* = 7.1 Hz, 3H) ppm.

^{13}C{^{1}H} NMR (101 MHz, $CDCl_3$) δ 173.2, 172.8, 154.8, 143.7, 143.7, 141.2, 132.0, 127.6, 127.0, 124.9, 124.9, 119.9, 66.6, 59.0, 52.4, 48.2, 47.1, 41.9, 22.9, 18.2 ppm.

HRMS (ESI-TOF) m/z: $[M + H]^+$ Calcd for $C_{25}H_{29}N_2O_5^+$ 437.2071; Found 437.2071.

IR (film): ν_{max} (cm^{-1}) 3343, 2953, 1735, 1664, 1492, 1450, 1376, 1330, 1244, 1166, 1090, 1057, 759, 741.

Optical rotation: $[\alpha]_D^{25}$ = -22.71 (*c* = 0.900, $CHCl_3$, >99% ee, >20:1 dr).

HPLC: DAICEL CHIRALPAK ID, hexane/*i*-PrOH = 80/20, flow rate: 1.0 mL/min, λ = 254 nm, t_R(major) = 12.3 min, t_R(minor) = 13.9 min, >99% ee, >20:1 dr.

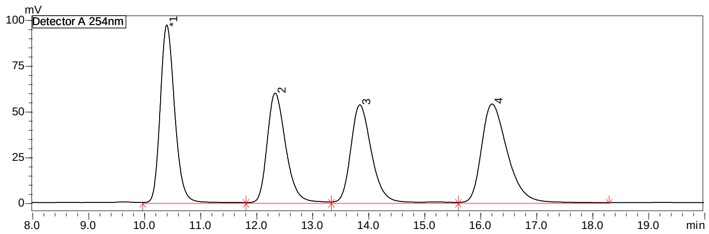

Peak#	Ret. Time	Area%
1	10.408	27.942
2	12.343	22.213
3	13.855	22.157
4	16.215	27.689

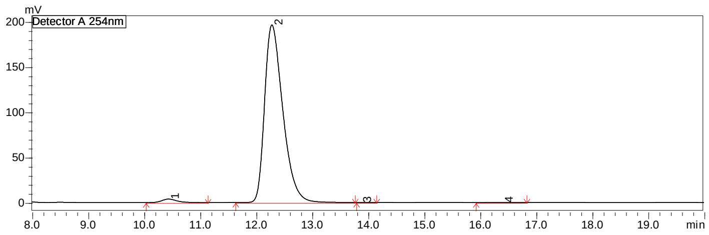

Peak#	Ret. Time	Area%
1	10.440	1.552
2	12.284	98.410
3	13.869	0.006
4	16.396	0.031

methyl ((*S*)-2-((((9*H*-fluoren-9-yl)methoxy)carbonyl)amino)-2-methylpent-4-enoyl)-*L*-alaninate ((***S*,*S***)-**10**)

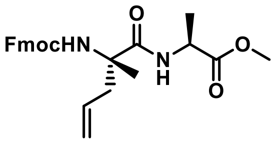

Procedure E. Purification by flash column chromatography (PE:EA = 5:1) afforded the desired product as a yellow oil (61.0 mg, 70% yield, >20:1 dr (Dr was determined by HPLC analysis of (***S*,*S***)-**10** after column chromatography)).

^{1}H NMR (400 MHz, $CDCl_3$) δ 7.77 (d, *J* = 7.5 Hz, 2H), 7.59 (dd, *J* = 7.2, 2.9 Hz, 2H), 7.40 (t, *J* = 7.4 Hz, 2H), 7.32 (t, *J* = 7.4 Hz, 2H), 6.73 (s, 1H), 5.69 (dq, *J* = 16.8, 7.4 Hz, 1H), 5.44 (s, 1H), 5.22 – 5.02 (m, 2H), 4.64 – 4.49 (m, 1H), 4.41 (d, *J* = 5.9 Hz, 2H), 4.21 (t, *J* = 6.7 Hz, 1H), 3.73 (s, 3H), 2.66 (s, 2H), 1.52 (s, 3H), 1.40 (d, *J* = 7.1 Hz, 3H) ppm.

$^{13}C\{^1H\}$ NMR (101 MHz, $CDCl_3$) δ 173.2, 172.9, 154.9, 143.7, 143.7, 141.2, 132.0, 127.6, 127.0, 124.9, 119.9, 66.6, 59.0, 52.4, 48.2, 47.1, 41.2, 23.2, 18.2 ppm.

HRMS (ESI-TOF) m/z: [M + H]$^+$ Calcd for $C_{25}H_{29}N_2O_5^+$ 437.2071; Found 437.2071.

IR (film): ν_{max} (cm^{-1}) 3342, 2954, 1732, 1667, 1496, 1450, 1377, 1318, 1244, 1166, 1090, 1053, 800, 759, 741.

Optical rotation: $[\alpha]_D^{25}$ = -5.44 (*c* = 0.720, $CHCl_3$, 99% ee, >20:1 dr).

HPLC: DAICEL CHIRALPAK ID, hexane/*i*-PrOH = 80/20, flow rate: 1.0 mL/min, λ = 254 nm, t_R(minor) = 10.0 min, t_R(major) = 16.0 min, 99% ee, >20:1 dr.

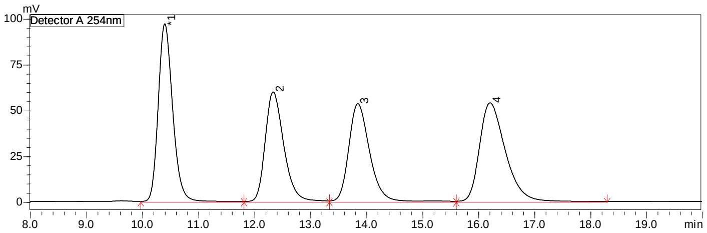

Peak#	Ret. Time	Area%
1	10.408	27.942
2	12.343	22.213
3	13.855	22.157
4	16.215	27.689

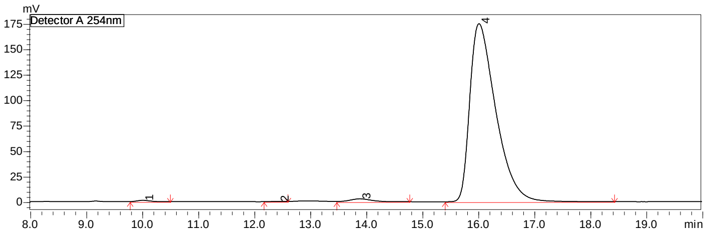

Peak#	Ret. Time	Area%
1	10.017	0.340
2	12.434	0.029
3	13.892	1.286
4	16.018	98.345

2.2 Determination of the Absolute Configurations of the Products

2.2.1 Determination of the Absolute Configuration of **3aw**

The comparison of the optical rotation of the product **3aw** ($[\alpha]_D^{25}$ = -56.91 (*c* = 0.900, $CHCl_3$, 93% ee)) with **(*S*)-3aw** ($[\alpha]_D^{23}$ = -60.7 (c = 1.69, $CHCl_3$, 97% ee)) in literature[15] indicated that the configuration of the product was *S*. The absolute configurations of **3aa-3av**, **3ax-3dg**, **(*S*,*S*)-6**, **(*S*,*R*)-6**, **(*R*,*S*)-8**, **(*S*,*S*)-8**, **(*R*,*S*)-10**, and **(*S*,*S*)-10** were deduced by analogy.

2.2.2 Determination of the Absolute Configuration of **3ge'**

A solution of **3ge'** in petroleum ether and diethyl ether in a glass bottle was left at room temperature to grow single crystals. Then the absolute configuration of **3ge'** was determined by X-ray crystallography. The absolute configurations of **3ea-3hg** were deduced by analogy. CCDC 2201046 contains the supplementary crystallographic data of **3ge'**.

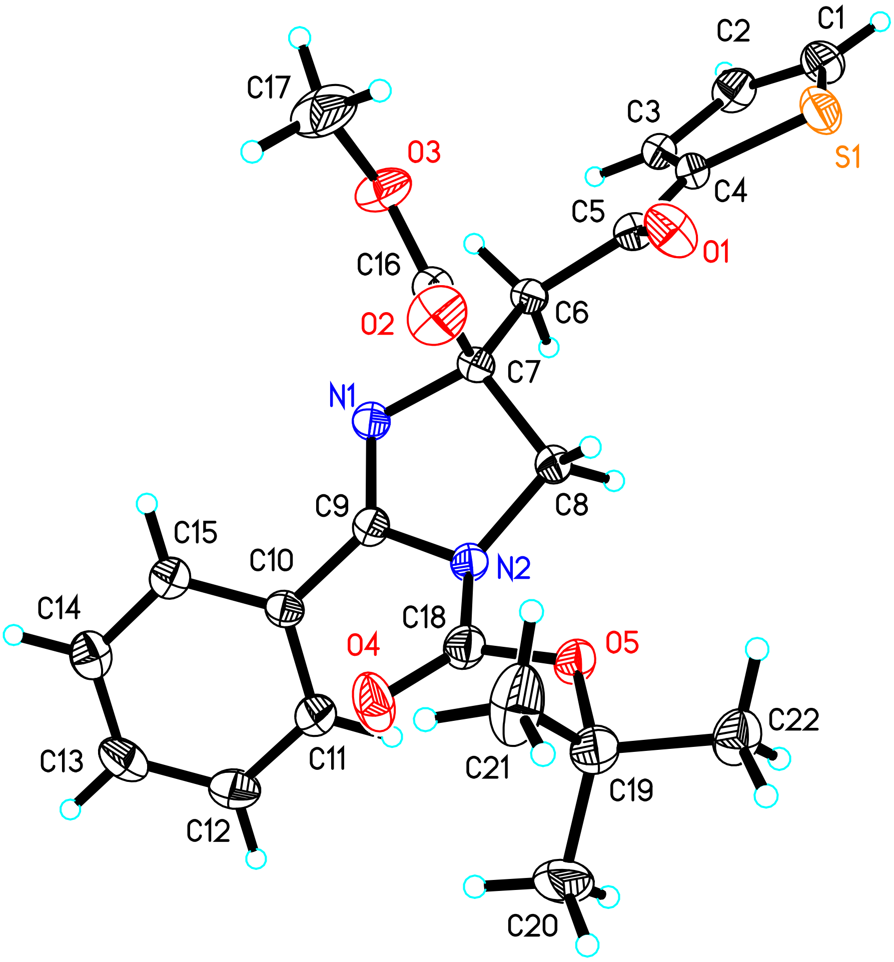

Supplementary Fig. 1. X-ray structure of **3ge'** (CCDC 2201046, 30% ellipsoid contour probability)

Supplementary Table 1. Crystal data and structure refinement for CCDC 2201046

Identification code	mo_d8v21664_0m	
Empirical formula	C22 H24 N2 O5 S	
Formula weight	428.49	
Temperature	213(2) K	
Wavelength	0.71073 Å	
Crystal system	Orthorhombic	
Space group	P 21 21 21	
Unit cell dimensions	a = 9.7501(7) Å	a= 90°.
	b = 11.2757(9) Å	b= 90°.
	c = 20.1132(15) Å	g = 90°.
Volume	2211.2(3) $Å^3$	
Z	4	
Density (calculated)	1.287 Mg/m^3	
Absorption coefficient	0.181 mm^{-1}	
F(000)	904	
Crystal size	0.160 x 0.120 x 0.080 mm^3	
Theta range for data collection	2.714 to 25.999°.	
Index ranges	-12<=h<=12, -13<=k<=12, -24<=l<=20	
Reflections collected	11230	
Independent reflections	4333 [R(int) = 0.0516]	
Completeness to theta = 25.242°	99.7 %	
Absorption correction	Semi-empirical from equivalents	
Max. and min. transmission	0.7456 and 0.6702	
Refinement method	Full-matrix least-squares on F^2	
Data / restraints / parameters	4333 / 0 / 275	
Goodness-of-fit on F^2	1.081	
Final R indices [I>2sigma(I)]	R1 = 0.0488, wR2 = 0.0879	
R indices (all data)	R1 = 0.0801, wR2 = 0.1031	
Absolute structure parameter	0.00(7)	
Extinction coefficient	n/a	
Largest diff. peak and hole	0.196 and -0.186 e.$Å^{-3}$	

2.3 Control Experiments and Proposed Mechanism

2.3.1 NMR Analyses of Some Mixtures

To get insight into the mechanism, both ^{1}H (Supplementary Fig. 2) and ^{31}P (Supplementary Fig. 3) NMR analyses for the $CDCl_3$ (1.0 mL, 0.03 M) solution of (*S*,*S*$_P$)-^{t}Bu-FOXAP (14.9 mg, 0.03 mmol, 1 equiv), $[Cu(CH_3CN)_4]PF_6$ (11.2 mg, 0.03 mmol, 1 equiv), and substrate **1a** (8.9 mg, 0.03 mmol, 1 equiv) were used. In a $CDCl_3$ solution of (*S*,*S*$_P$)-^{t}Bu-FOXAP, $[Cu(CH_3CN)_4]PF_6$, and **1a**, both O^{t}Bu and CH_2 have different ^{1}H NMR signals from the ones of **1a**, which indicates the formation of the Cu(I)-(*S*,*S*$_P$)-^{t}Bu-FOXAP-**1a** complex. Moreover, the differences in three ^{31}P NMR peaks of (*S*,*S*$_P$)-^{t}Bu-FOXAP, (*S*,*S*$_P$)-^{t}Bu-FOXAP with $[Cu(CH_3CN)_4]PF_6$, and the mixture of (*S*,*S*$_P$)-^{t}Bu-FOXAP, $[Cu(CH_3CN)_4]PF_6$, and **1a** in Supplementary Fig. 3 also support the conclusion.

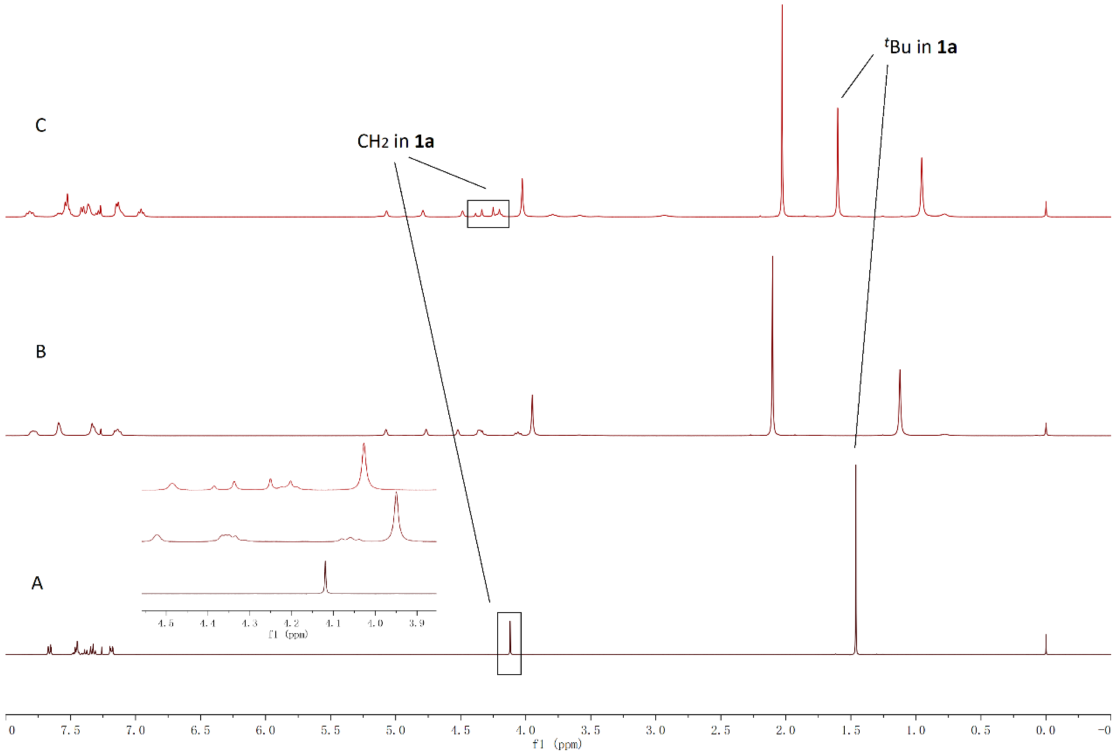

Supplementary Fig. 2. ^{1}H NMR Analyses. A: substrate **1a** (8.9 mg, 0.03 mmol, 1 equiv) in $CDCl_3$ (1.0 mL, 0.03 M, r.t.). B: (*S*,*S*$_P$)-^{t}Bu-FOXAP (14.9 mg, 0.03 mmol, 1 equiv) + $[Cu(CH_3CN)_4]PF_6$ (11.2 mg, 0.03 mmol, 1 equiv) in $CDCl_3$ (1.0 mL, 0.03 M, r.t.). C: (*S*,*S*$_P$)-^{t}Bu-FOXAP (14.9 mg, 0.03 mmol, 1 equiv) + $[Cu(CH_3CN)_4]PF_6$ (11.2 mg, 0.03 mmol, 1 equiv) + substrate **1a** (8.9 mg, 0.03 mmol, 1 equiv) in $CDCl_3$ (1.0 mL, 0.03 M, r.t.).

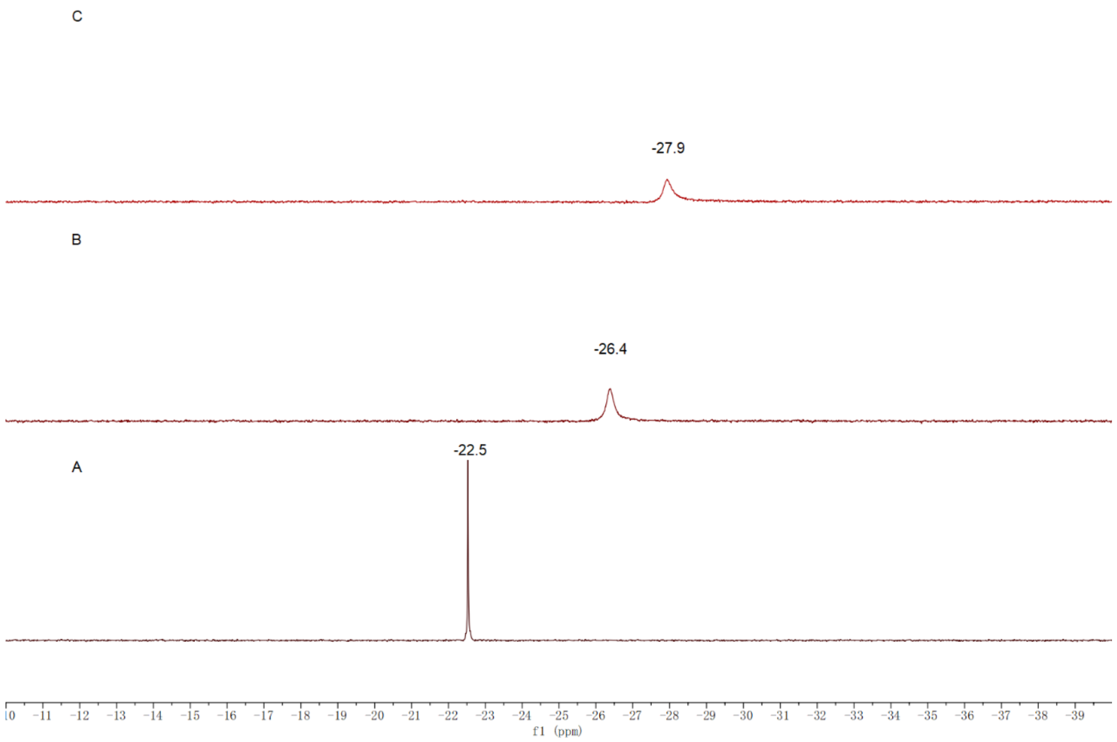

Supplementary Fig. 3. ^{31}P NMR Analyses. A: (S,S_P)-^{t}Bu-FOXAP (14.9 mg, 0.03 mmol) in $CDCl_3$ (1.0 mL, 0.03 M, r.t.). B: (S,S_P)-^{t}Bu-FOXAP (14.9 mg, 0.03 mmol, 1 equiv) + $[Cu(CH_3CN)_4]PF_6$ (11.2 mg, 0.03 mmol, 1 equiv) in $CDCl_3$ (1.0 mL, 0.03 M, r.t.). C: (S,S_P)-^{t}Bu-FOXAP (14.9 mg, 0.03 mmol, 1 equiv) + $[Cu(CH_3CN)_4]PF_6$ (11.2 mg, 0.03 mmol, 1 equiv) + substrate **1a** (8.9 mg, 0.03 mmol, 1 equiv) in $CDCl_3$ (1.0 mL, 0.03 M, r.t.).

2.3.2 D/H Exchange Experiments

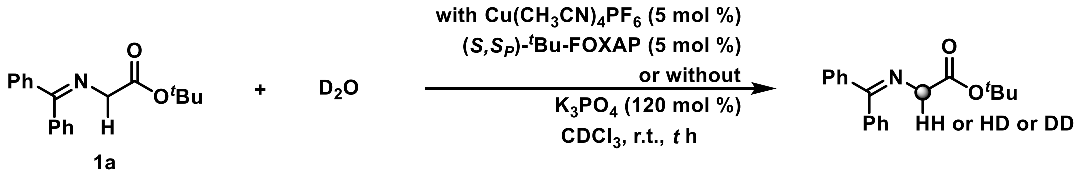

D/%	10 min	30 min	2 h	12 h
without [Cu]	< 5%	< 5%	< 5%	< 5%
with [Cu]	76%	85%	86%	88%

A dried NMR tube was charged with $[Cu(CH_3CN)_4]PF_6$ (1.8 mg, 0.005 mmol, 0.05 equiv), (S,S_P)-^{t}Bu-FOXAP (2.5 mg, 0.005 mmol, 0.05 equiv), and K_3PO_4 (25.5 mg, 0.12 mmol, 1.2 equiv) in a glove box under Ar atmosphere. $CDCl_3$ (1.0 mL) was added to give a mixture. Then α-imino-eater **1a** (29.5 mg, 0.1 mmol, 1.0 equiv) and D_2O (18.0 μL, 1 mmol, 10 equiv) were added. ^1H NMR analysis at the mentioned time using ^{t}Bu group as an integration standard determined the D/H ratio.

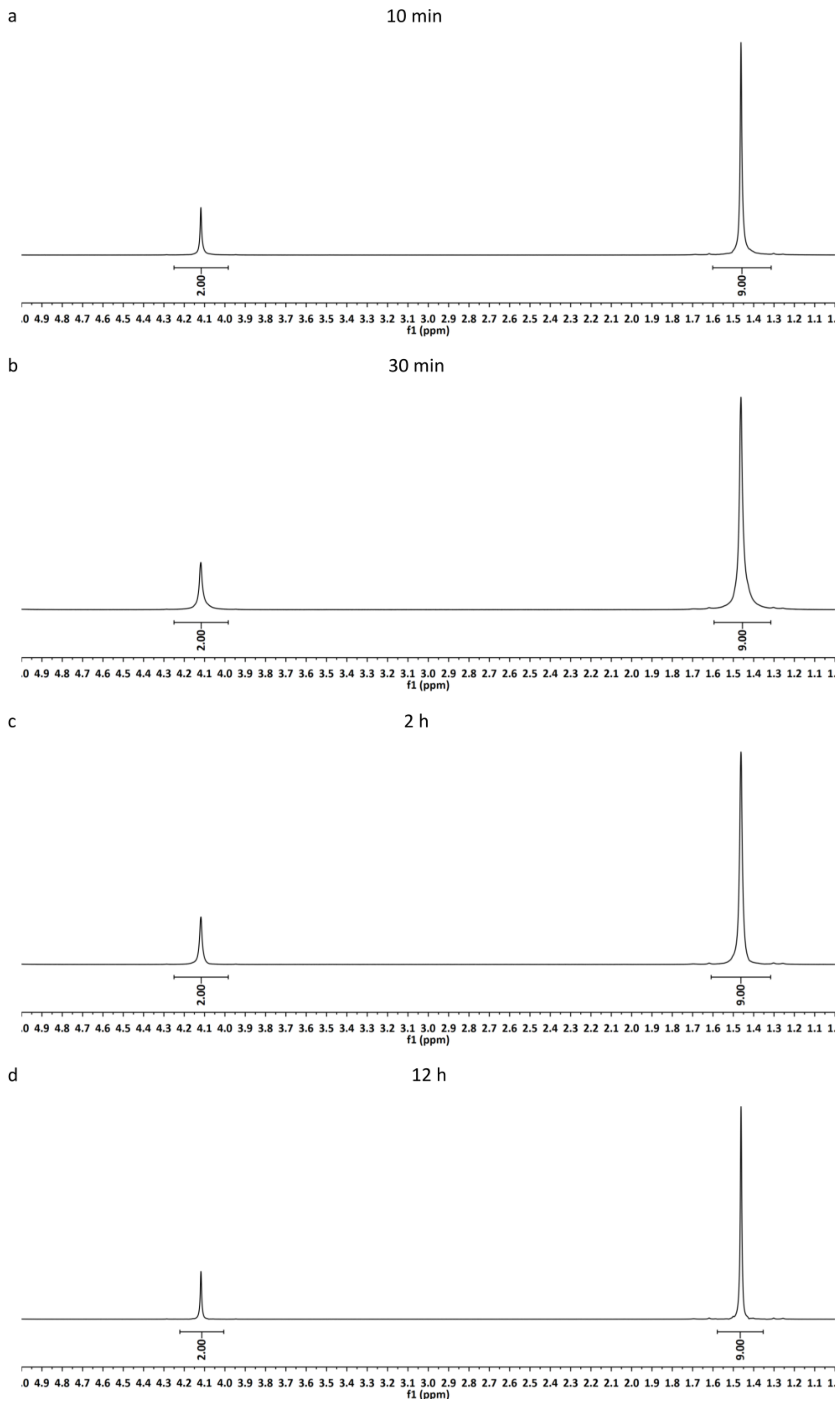

Supplementary Fig. 4. ^{1}H NMR integrations for D/H exchange experiments without $[Cu(CH_3CN)_4]PF_6$ and (S,S_P)-^{t}Bu-FOXAP at different reaction times. **a** At 10 min. **b** At 30 min. **c** At 2 h. **d** At 12 h.

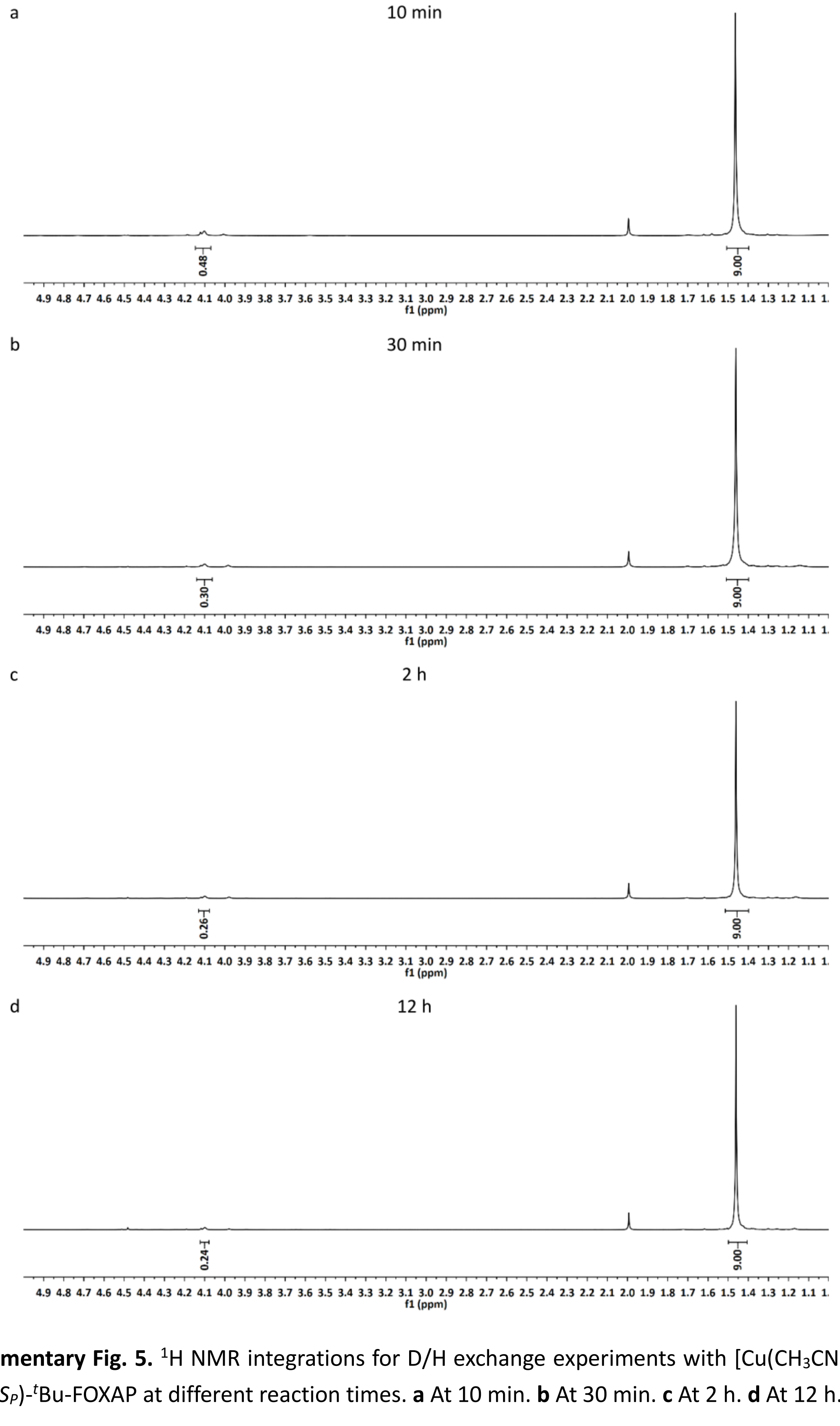

Supplementary Fig. 5. ^{1}H NMR integrations for D/H exchange experiments with $[Cu(CH_3CN)_4]PF_6$ and (S,S_P)-^{t}Bu-FOXAP at different reaction times. **a** At 10 min. **b** At 30 min. **c** At 2 h. **d** At 12 h.

2.3.3 Unsuccessful Alkylation with Methyl 2-((Diphenylmethylene)amino)propanoate

In the alkylation described in Table 2, we did not observe any dialkylated products. Actually, the substrate with the imino group derived from benzophenone did not undergo the alkylation to afford the tetrasubstituted carbon center as shown below. Therefore, it is realized that the substrates with imino group derived from benzaldehyde and benzophenone have big difference on the reactivity. Evidently, the substrates with the imino group derived from benzophenone only allowed facile preparation of trisubstituted carbon center in the present catalytic system.

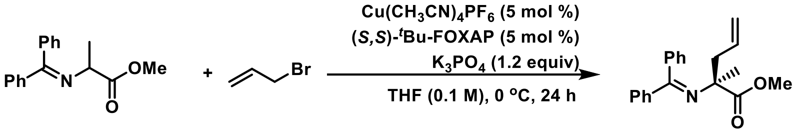

0.15 mmol   0.1 mmol   Not detected

Following procedure A, however a 0.1 mmol scale reaction was carried out.

2.3.4 Asymmetric Alkylation with a Secondary Alkyl Halide

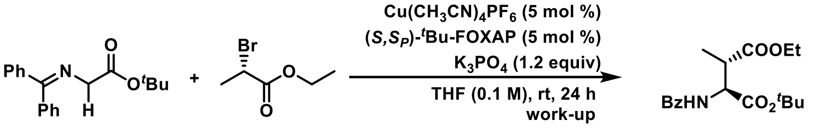

89% ee

(*S*,*S*)-6
87%, >20/1 dr, >99% ee

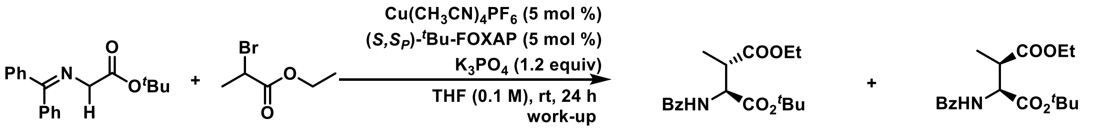

racemic

(*S*,*S*)-6
25%, >20/1 dr, 99% ee

(*S*,*R*)-6
35%, >20/1 dr, 98% ee

Following procedure F, however using THF as the solvent instead of DME.

When using **(*S*)-5** (36.2 mg, 0.2 mmol, 1.0 equiv, 89% ee) as the alkyl bromide, purification by flash column chromatography (PE:EA = 15:1) afforded the product **(*S*,*S*)-6** as a colorless oil (50.0 mg, 87% yield, >99% ee, >20:1 dr (Dr was determined by both ^{1}H NMR and HPLC analysis of **(*S*,*S*)-6** after column chromatography)).

When using ***rac*-5** (36.2 mg, 0.2 mmol, 1.0 equiv) as the alkyl bromide, purification by flash column chromatography (PE:EA = 15:1) afforded the product **(*S*,*S*)-6** as a colorless oil (16.8 mg, 25% yield, 99% ee, >20:1 dr (Dr was determined by both ^{1}H NMR and HPLC analysis of **(*S*,*S*)-6** after column chromatography)) and the product **(*S*,*R*)-6** as a colorless oil (23.5 mg, 35% yield, 98% ee, >20:1 dr (Dr was determined by both ^{1}H NMR and HPLC analysis of **(*S*,*R*)-6** after column chromatography))

When using **(*S*)-5** (18.1 mg, 0.1 mmol, 1.0 equiv, 87% ee, 0.1 mmol scale reaction) as the alkyl bromide without **1a**, the ee of **(*S*)-5** remained unchanged, indicating that no racemization of **(*S*)-5** occurred in the reaction condition.

1-(*tert*-butyl) 4-ethyl (2*S*,3*S*)-2-benzamido-3-methylsuccinate (**(*S*,*S*)-6**)

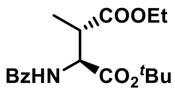

^{1}H NMR (400 MHz, $CDCl_3$) δ 7.82 – 7.75 (m, 2H), 7.54 – 7.48 (m, 1H), 7.46 – 7.40 (m, 2H), 7.00 (d, *J* = 8.1 Hz, 1H), 4.95 (dd, *J* = 8.2, 4.1 Hz, 1H), 4.22 – 4.12 (m, 2H), 3.07 (qd, *J* = 7.3, 4.1 Hz, 1H), 1.49 (s, 9H), 1.33 (d, *J* = 7.3 Hz, 3H), 1.28 (t, *J* = 7.1 Hz, 3H) ppm.

$^{13}C\{^1H\}$ NMR (126 MHz, $CDCl_3$) δ 173.1, 169.5, 166.8, 133.9, 131.6, 128.5, 127.0, 82.8, 61.0, 54.7, 42.4, 27.9, 14.1, 13.2 ppm.

HRMS (ESI-TOF) m/z: $[M + H]^+$ Calcd for $C_{18}H_{26}NO_5^+$ 336.1805; Found 336.1805.

IR (film): v_{max} (cm^{-1}) 3350, 2980, 2936, 1738, 1652, 1526, 1488, 1394, 1369, 1258, 1155, 1110, 1074, 1027, 844, 713, 694.

Optical rotation: $[\alpha]_D^{25}$ = +45.37 (*c* = 1.020, $CHCl_3$, >99% ee, >20:1 dr).

HPLC: DAICEL CHIRALPAK ID, hexane/*i*-PrOH = 90/10, flow rate: 1.0 mL/min, λ = 254 nm, t_R(major) = 16.7 min, t_R(minor) = 20.4 min, >99% ee, >20:1 dr.

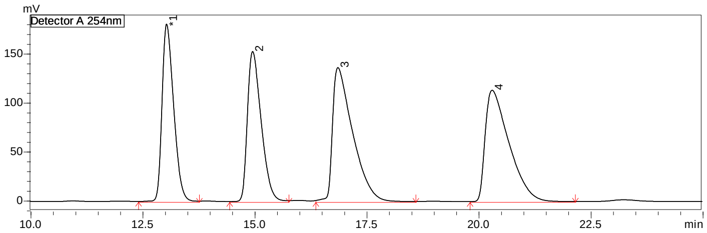

Peak#	Ret. Time	Area%
1	13.045	21.796
2	14.964	21.826
3	16.869	28.465
4	20.312	27.914

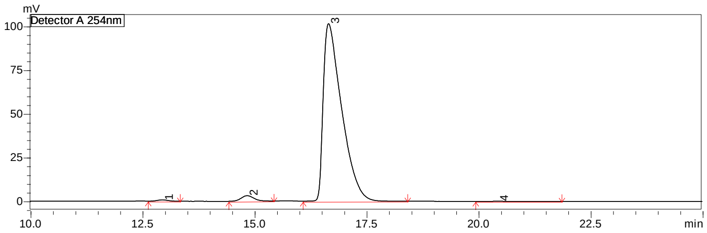

Peak#	Ret. Time	Area%
1	12.950	0.414
2	14.842	2.060
3	16.659	97.478
4	20.428	0.048

1-(*tert*-butyl) 4-ethyl (2*S*,3*R*)-2-benzamido-3-methylsuccinate (**(*S*,*R*)-6**)

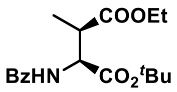

^{1}H NMR (400 MHz, $CDCl_3$) δ 7.89 – 7.80 (m, 2H), 7.56 – 7.49 (m, 1H), 7.49 – 7.42 (m, 2H), 7.14 (d, *J* = 8.7 Hz, 1H), 5.00 (dd, *J* = 8.7, 3.7 Hz, 1H), 4.18 (q, *J* = 7.1 Hz, 2H), 3.31 (qd, *J* = 7.3, 3.7 Hz, 1H), 1.47 (s, 9H), 1.29 (t, *J* = 7.2 Hz, 3H), 1.26 (d, *J* = 7.3 Hz, 3H) ppm.
^{13}C{^{1}H} NMR (126 MHz, $CDCl_3$) δ 174.2, 169.6, 167.4, 134.0, 131.7, 128.6, 127.0, 82.6, 61.0, 54.5, 41.3, 27.9, 14.2, 13.5 ppm.
HRMS (ESI-TOF) m/z: $[M + H]^+$ Calcd for $C_{18}H_{26}NO_5^+$ 336.1805; Found 336.1806.
IR (film): v_{max} (cm^{-1}) 3432, 2962, 1735, 1654, 1602, 1580, 1508, 1483, 1458, 1395, 1368, 1260, 1198, 1153, 1096, 1022, 800, 712.
Optical rotation: $[\alpha]_D^{25}$ = +30.19 (*c* = 0.945, $CHCl_3$, 98% ee, >20:1 dr).
HPLC: DAICEL CHIRALPAK ID, hexane/*i*-PrOH = 90/10, flow rate: 1.0 mL/min, λ = 254 nm, t_R(minor) = 13.1 min, t_R(major) = 14.8 min, 98% ee, >20:1 dr.

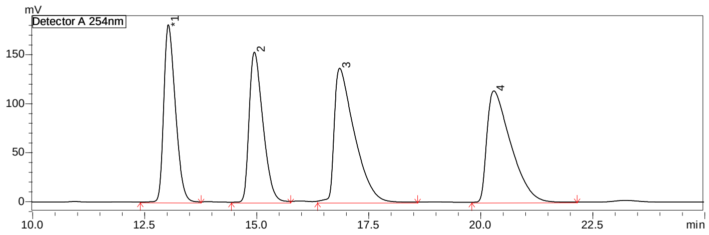

Peak#	Ret. Time	Area%
1	13.045	21.796
2	14.964	21.826
3	16.869	28.465
4	20.312	27.914

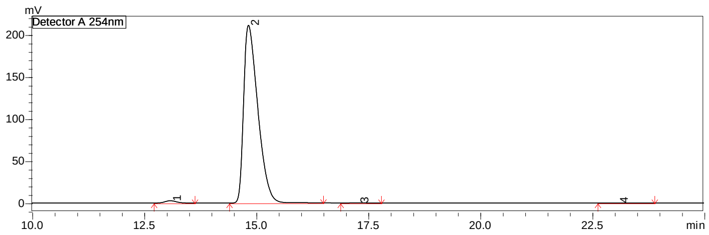

Peak#	Ret. Time	Area%
1	13.089	0.986
2	14.834	98.944
3	17.275	0.060
4	23.068	0.010

2.3.5 Proposed Mechanism

Based on the above control experiments, a mechanism was proposed below.

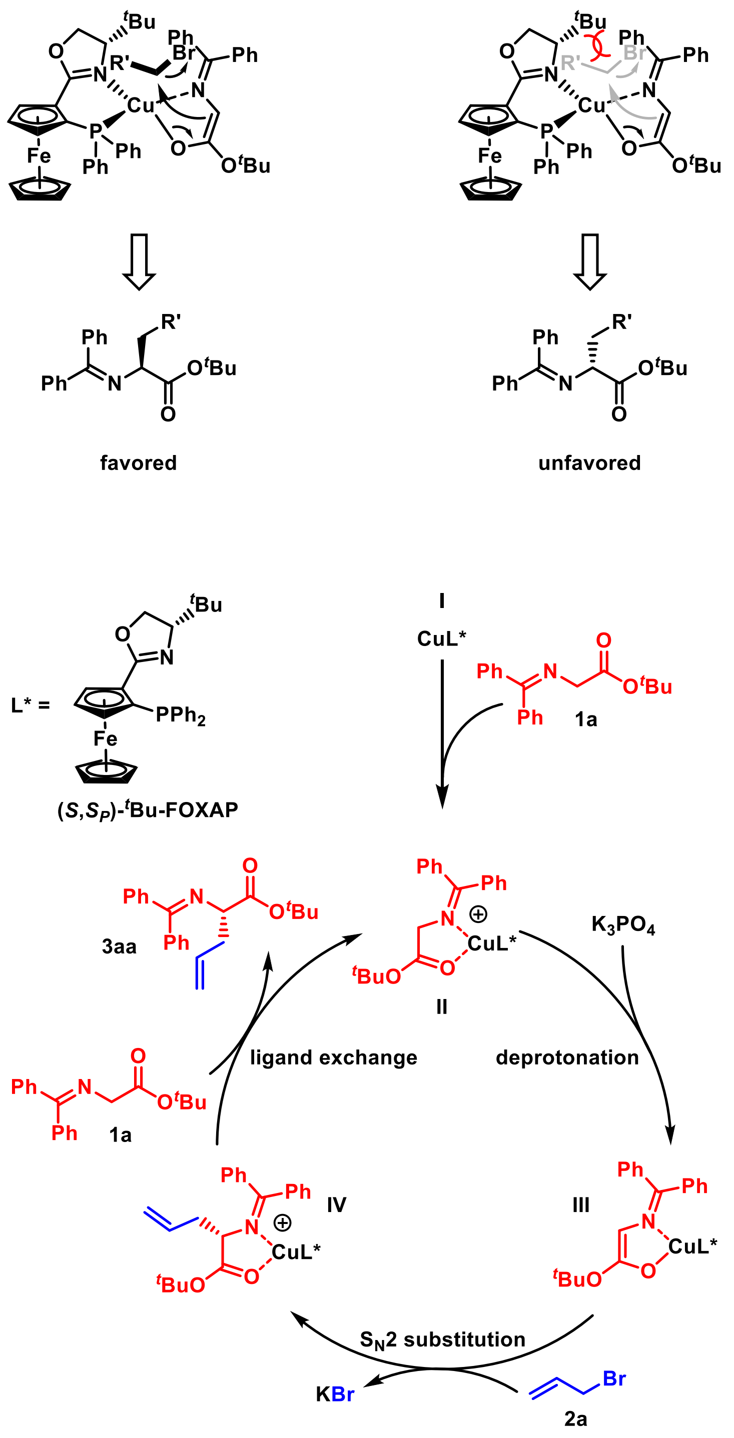

Supplementary Fig. 6. Proposed Mechanism

3 Supplementary References

1. O'Donnell, M. J. & Polt, R. L. A Mild and Efficient Route to Schiff Base Derivatives of Amino Acids. *J. Org. Chem.* **47**, 2663-2666 (1982).
2. McCune, C. D., Beio, M. L., Sturdivant, J. M., de la Salud-Bea, R., Darnell, B. M. & Berkowitz, D. B. Synthesis and Deployment of an Elusive Fluorovinyl Cation Equivalent: Access to Quaternary *α*-(1'-Fluoro)vinyl Amino Acids as Potential PLP Enzyme Inactivators. *J. Am. Chem. Soc.* **139**, 14077-14089 (2017).
3. Kim, T.-S., Lee, Y.-J., Jeong, B.-S., Park, H.-g. & Jew, S.-s. Enantioselective Synthesis of (*R*)- and (*S*)-*α*-Alkylcysteines via Phase-Transfer Catalytic Alkylation. *J. Org. Chem.* **71**, 8276-8278 (2006).
4. Dawsey, A. C., Li, V., Hamilton, K. C., Wang, J. & Williams, T. J. Copper-Catalyzed Oxidation of Azolines to Azoles. *Dalton Trans.* **41**, 7994-8002 (2012).
5. Park, Y., Kang, S., Lee, Y. J., Kim, T.-S., Jeong, B.-S., Park, H.-g. & Jew, S.-s. Highly Enantioselective Synthesis of (*S*)-*α*-Alkyl-*α*,*β*-diaminopropionic Acids via Asymmetric Phase-Transfer Catalytic Alkylation of 2-Phenyl-2-imidazoline-4-carboxylic Acid *tert*-Butyl Esters. *Org. Lett.* **11**, 3738-3741 (2009).
6. Lee, M., Lee, Y.-J., Park, E., Park, Y., Ha, M. W., Hong, S., Lee, Y.-J., Kim, T.-S., Kim, M.-h. & Park, H.-g. Highly Enantioselective Synthesis of 5-Phenyl-2-Alkylprolines Using Phase-Transfer Catalytic Alkylation. *Org. Biomol. Chem.* **11**, 2039-2046 (2013).
7. Lee, J., Ha, M. W., Kim, T.-S., Kim, M.-J., Ku, J.-M., Jew, S.-s., Park, H.-g. & Jeong, B.-S. Solid-Phase Synthesis of *α*-Alkylserines via Phase-Transfer Catalytic Alkylation of Polymer-Supported 2-Phenyl-2-oxazoline-4-carboxylate. *Tetrahedron* **65**, 8839-8843 (2009).
8. Miura, K., Tomita, M., Yamada, Y. & Hosomi, A. Indium-Catalyzed Radical Reductions of Organic Halides with Hydrosilanes. *J. Org. Chem.* **72**, 787-792 (2007).
9. Wimmer, Z., Šaman, D. & Francke, W. Novel Juvenoids of the 2-(4-Hydroxybenzyl)cyclohexan-1-one Series. *Helv. Chim. Acta* **77**, 502-508 (1994).
10. Lepore, S. D., Mondal, D., Li, S. Y. & Bhunia, A. K. Stereoretentive Halogenations and Azidations with Titanium(IV) Enabled by Chelating Leaving Groups. *Angew. Chem. Int. Ed.* **47**, 7511-7514 (2008).
11. Ooi, T., Kameda, M. & Maruoka, K. Design of *N*-Spiro C_2-Symmetric Chiral Quaternary Ammonium Bromides as Novel Chiral Phase-Transfer Catalysts: Synthesis and Application to Practical Asymmetric Synthesis of *α*-Amino Acids. *J. Am. Chem. Soc.* **125**, 5139-5151 (2003).
12. Nun, P., Pérez, V., Calmès, M., Martinez, J. & Lamaty, F. Preparation of Chiral Amino Esters by Asymmetric Phase-Transfer Catalyzed Alkylations of Schiff Bases in a Ball Mill. *Chem. - Eur. J.* **18**, 3773-3779 (2012).
13. Huo, X., Fu, J., He, X., Chen, J., Xie, F. & Zhang, W. Pd/Cu Dual Catalysis: Highly Enantioselective Access to *α*-Substituted *α*-Amino Acids and *α*-Amino Amides. *Chem. Commun.* **54**, 599-602 (2018).
14. Huynh, F., Tailby, M., Finniear, A., Stephens, K., Allemann, R. K. & Wirth, T. Accelerating Biphasic Biocatalysis through New Process Windows. *Angew. Chem. Int. Ed.* **59**, 16490-16495 (2020).
15. Andrus, M. B., Ye, Z. & Zhang, J. Highly Selective Glycine Phase-Transfer Catalysis Using

Fluoroanthracenylmethyl Cinchonidine Catalysts. *Tetrahedron Lett.* **46**, 3839-3842 (2005).

16. Majdecki, M., Niedbala, P. & Jurczak, J. Amide-Based Cinchona Alkaloids as Phase-Transfer Catalysts: Synthesis and Potential Application. *Org. Lett.* **21**, 8085-8090 (2019).
17. Hulin, B. & Lopaze, M. G. A Practical Synthesis of (*R*)- and (*S*)-3-Amino-3,4-dihydro-1*H*-quinolin-2-one. *Tetrahedron: Asymmetry* **15**, 1957-1958 (2004).
18. Feng, D., Xu, J., Wan, J., Xie, B. & Ma, X. Facile One-Pot Fabrication of a Silica Gel-Supported Chiral Phase-Transfer Catalyst—*N*-(2-Cyanobenzyl)-O(9)-allyl-cinchonidinium Salt. *Catal. Sci. Technol.* **5**, 2141-2148 (2015).
19. Schettini, R., Nardone, B., De Riccardis, F., Sala, G. D. & Izzo, I. Cyclopeptoids as Phase-Transfer Catalysts for the Enantioselective Synthesis of α-Amino Acids. *Eur. J. Org. Chem.* **2014**, 7793-7797 (2014).
20. Jin, L., Zhao, S. & Chen, X. Synthesis of Both Enantiomers of Chiral Phenylalanine Derivatives Catalyzed by Cinchona Alkaloid Quaternary Ammonium Salts as Asymmetric Phase Transfer Catalysts. *Molecules* **23**, 1421 (2018).
21. Xu, C., Qi, Y., Yang, X., Li, X., Li, Z. & Bai, L. Development of C_2-Symmetric Chiral Spirocyclic Phase-Transfer Catalysts: Synthesis and Application to Asymmetric Alkylation of Glycinate Schiff Base. *Org. Lett.* **23**, 2890-2894 (2021).
22. Manaprasertsak, A., Tharamak, S., Schedl, C., Roller, A. & Widhalm, M. Improved Access to Chiral Tetranaphthoazepinium-Based Organocatalysts Using Aqueous Ammonia as Nitrogen Source. *Molecules* **24**, 3844 (2019).
23. O'Donnell, M. J., Drew, M. D., Cooper, J. T., Delgado, F. & Zhou, C. The Enantioselective Synthesis of α-Amino Acid Derivatives via Organoboranes. *J. Am. Chem. Soc.* **124**, 9348-9349 (2002).
24. Siebum, A. H. G., Woo, W. S. & Lugtenburg, J. Preparation and Characterization of [5-^{13}C]-(2*S*,4*R*)-Leucine and [4-^{13}C]-(2*S*,3*S*)-Valine − Establishing Synthetic Schemes to Prepare Any Site-Directed Isotopomer of L-Leucine, L-Isoleucine and L-Valine. *Eur. J. Org. Chem.* **2003**, 4664-4678 (2003).
25. Juaristi, E., Balderas, M., López-Ruiz, H., Jiménez-Pérez, V. c. M., Kaiser-Carril, M. a. L. & Ramírez-Quirós, Y. Enantioselective Synthesis of β-Amino Acids. Part 10: Preparation of Novel α,α- and β,β-Disubstituted β-Amino Acids from (*S*)-Asparagine. *Tetrahedron: Asymmetry* **10**, 3493-3505 (1999).

4 Supplementary Figures

4.1 ^{1}H, ^{13}C{^{1}H}, and ^{19}F NMR Spectra of New Compounds

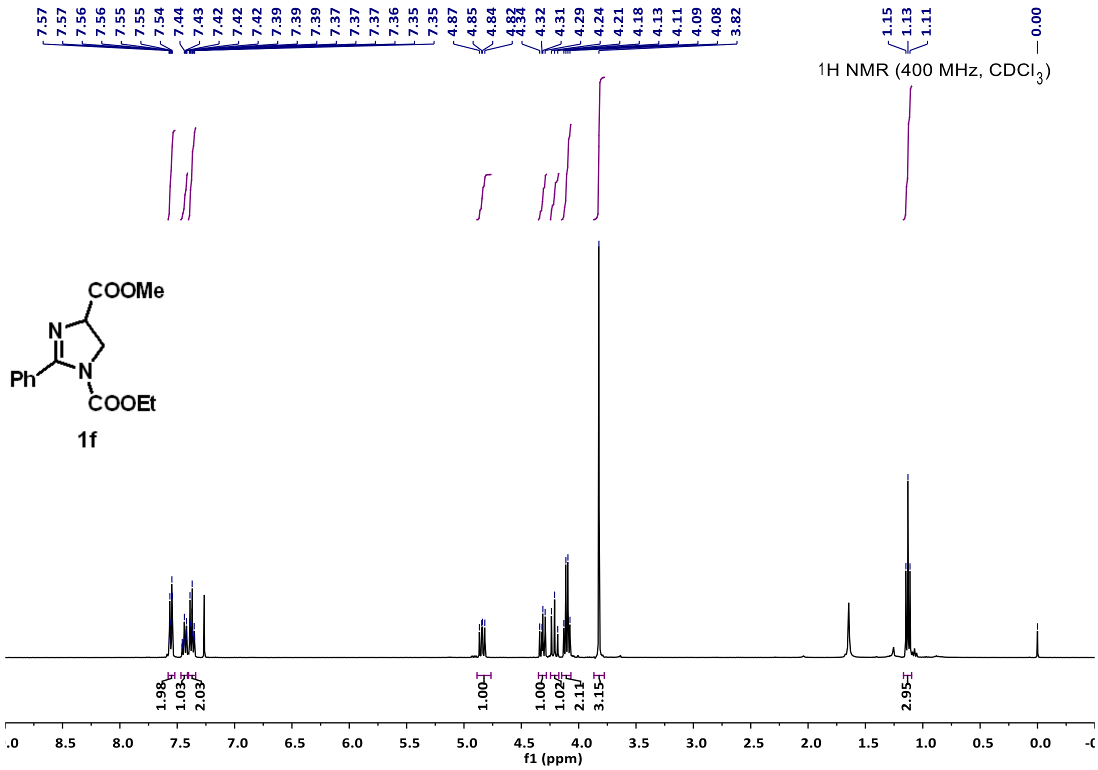

Supplementary Fig. 7. ^{1}H NMR spectrum of compound **1f**

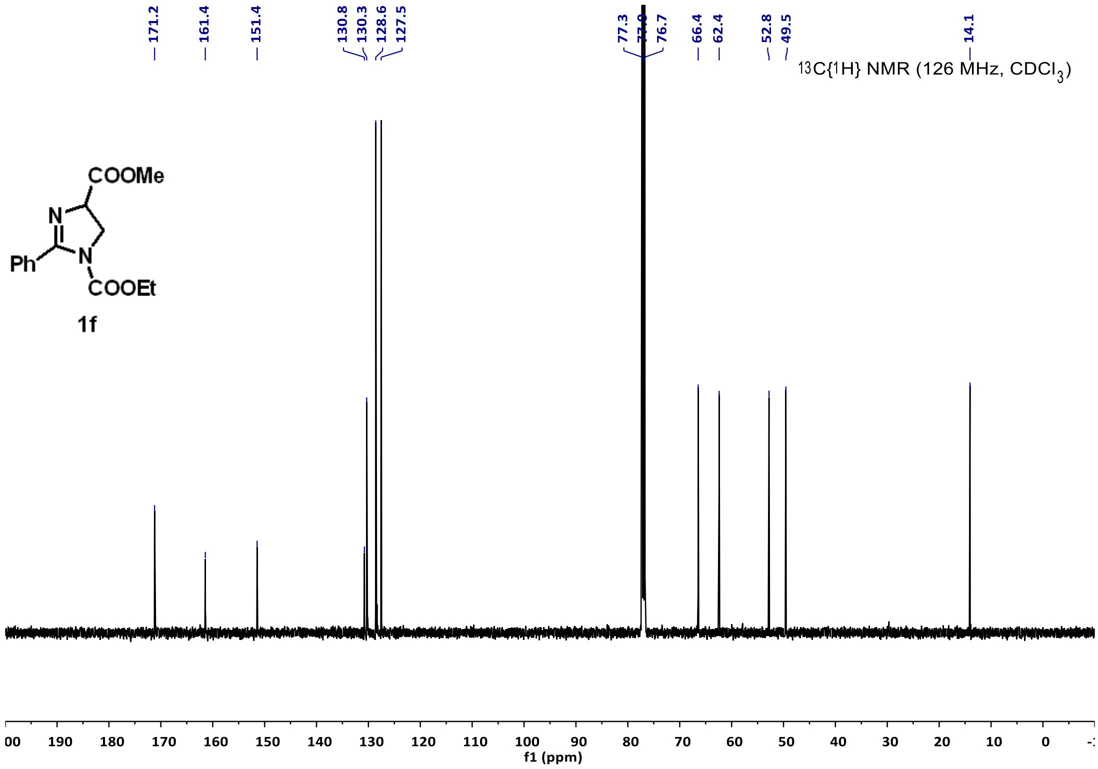

Supplementary Fig. 8. ^{13}C NMR spectrum of compound **1f**

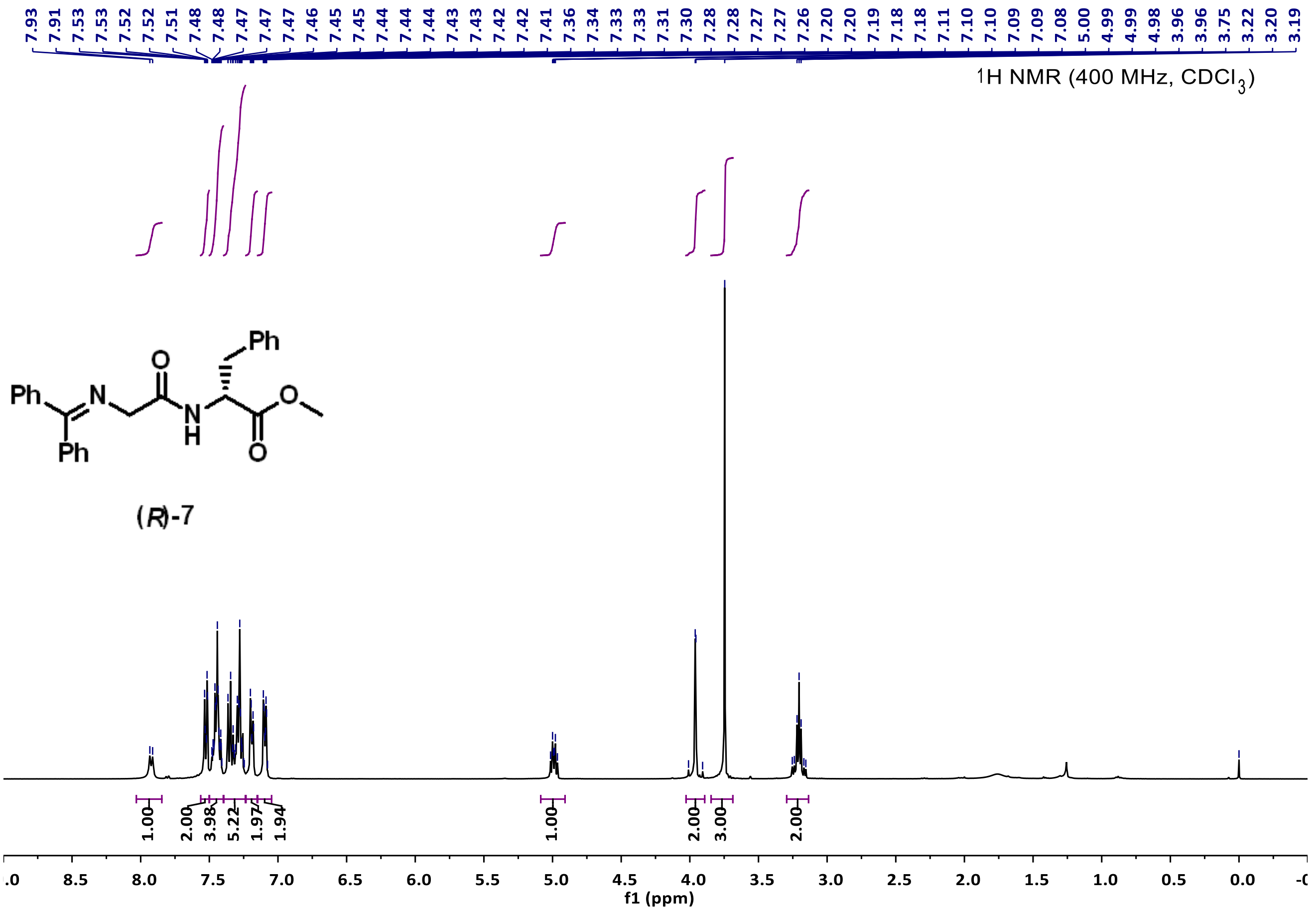

Supplementary Fig. 9. ^{1}H NMR spectrum of compound **(*R*)-7**

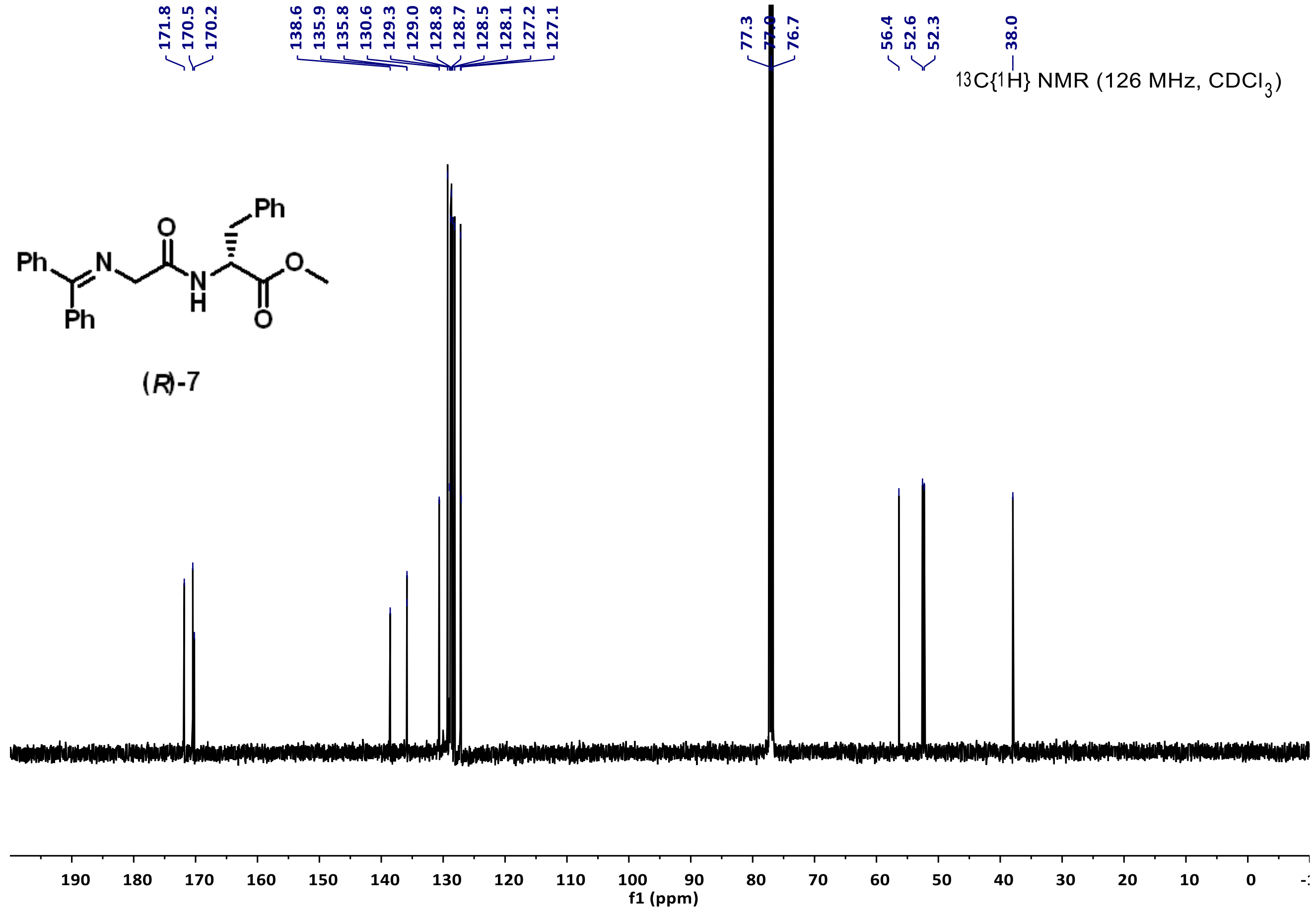

Supplementary Fig. 10. ^{13}C NMR spectrum of compound **(*R*)-7**

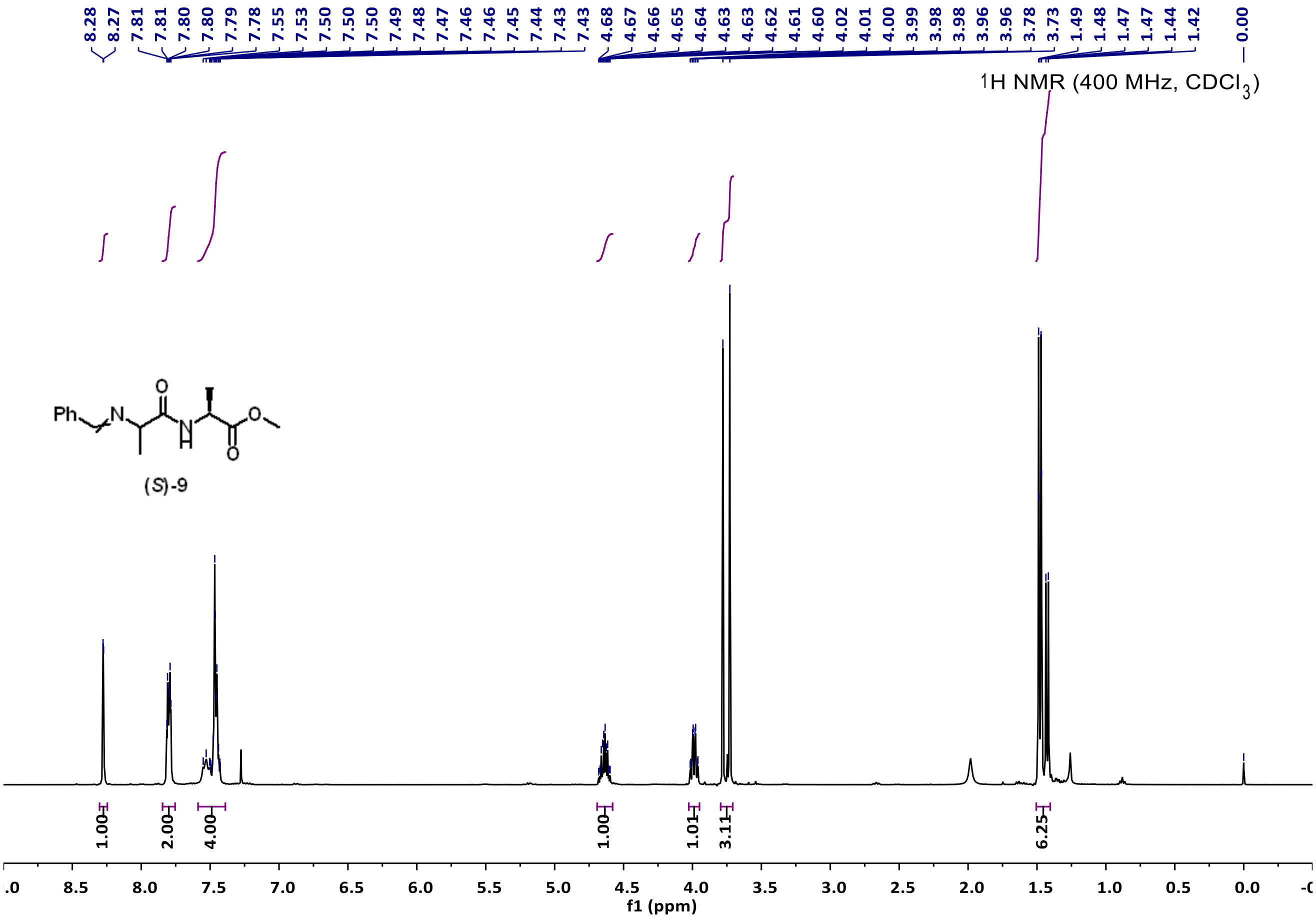

Supplementary Fig. 11. ^{1}H NMR spectrum of compound **(*S*)-9**

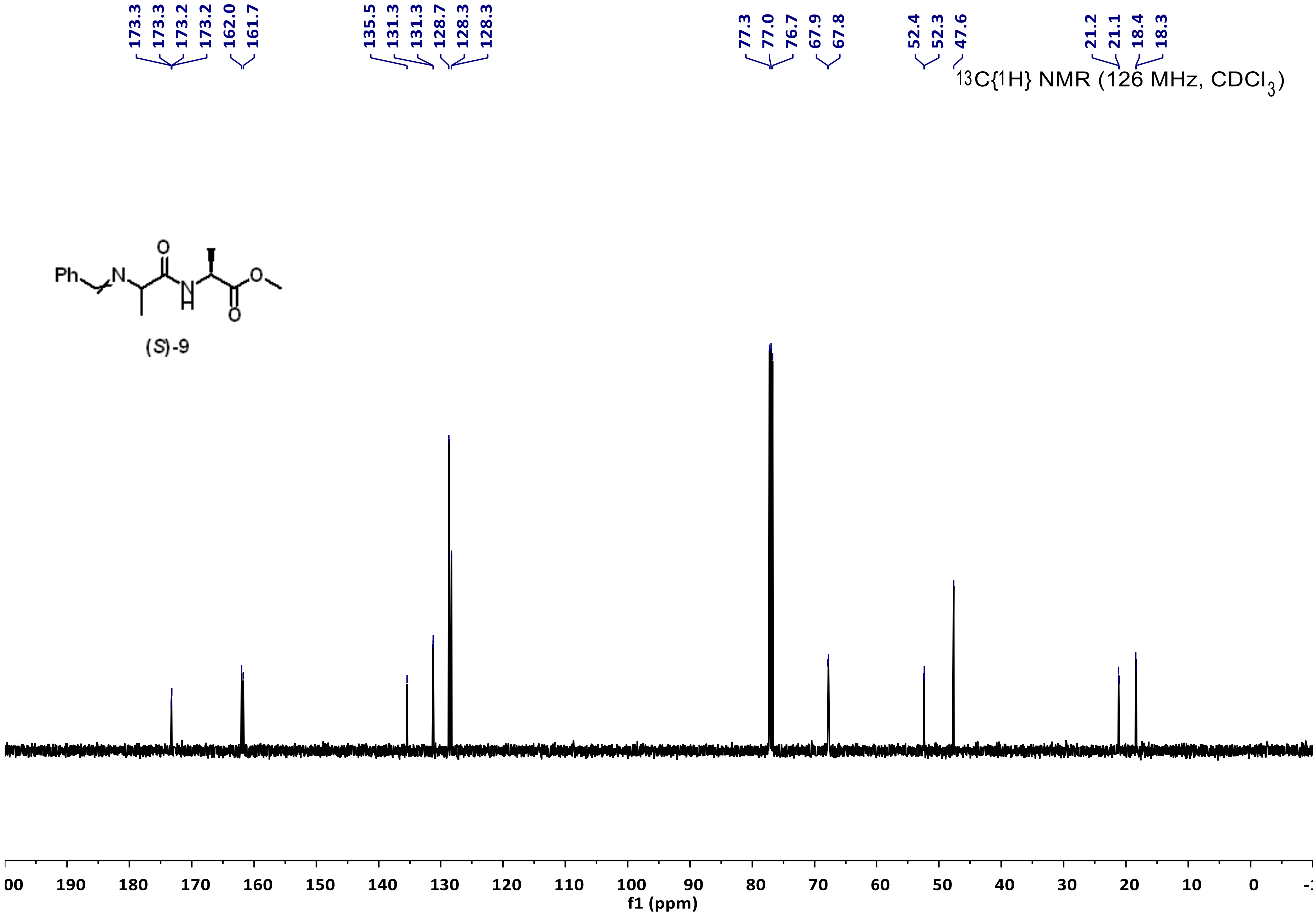

Supplementary Fig. 12. ^{13}C NMR spectrum of compound **(*S*)-9**

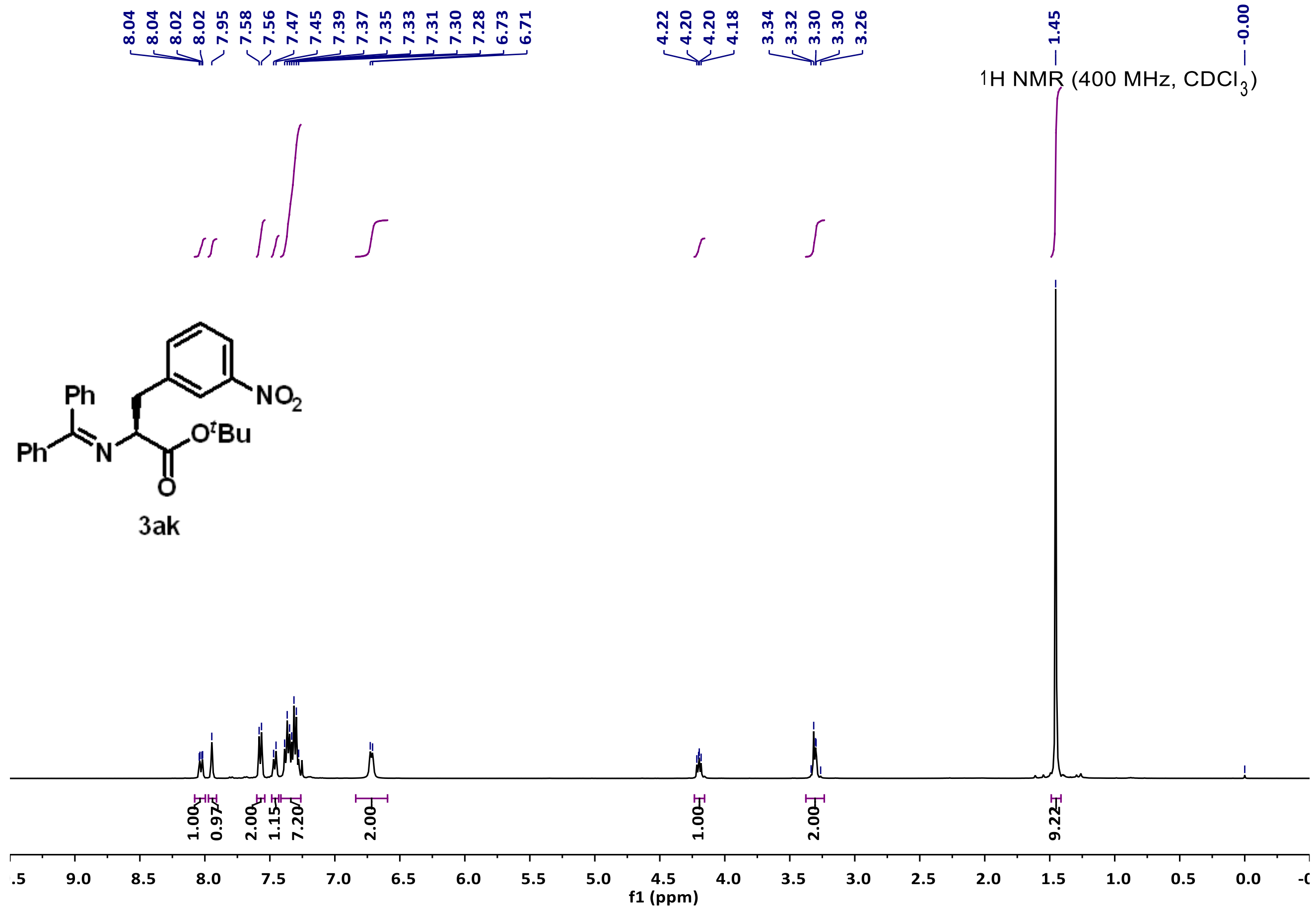

Supplementary Fig. 13. ^{1}H NMR spectrum of compound **3ak**

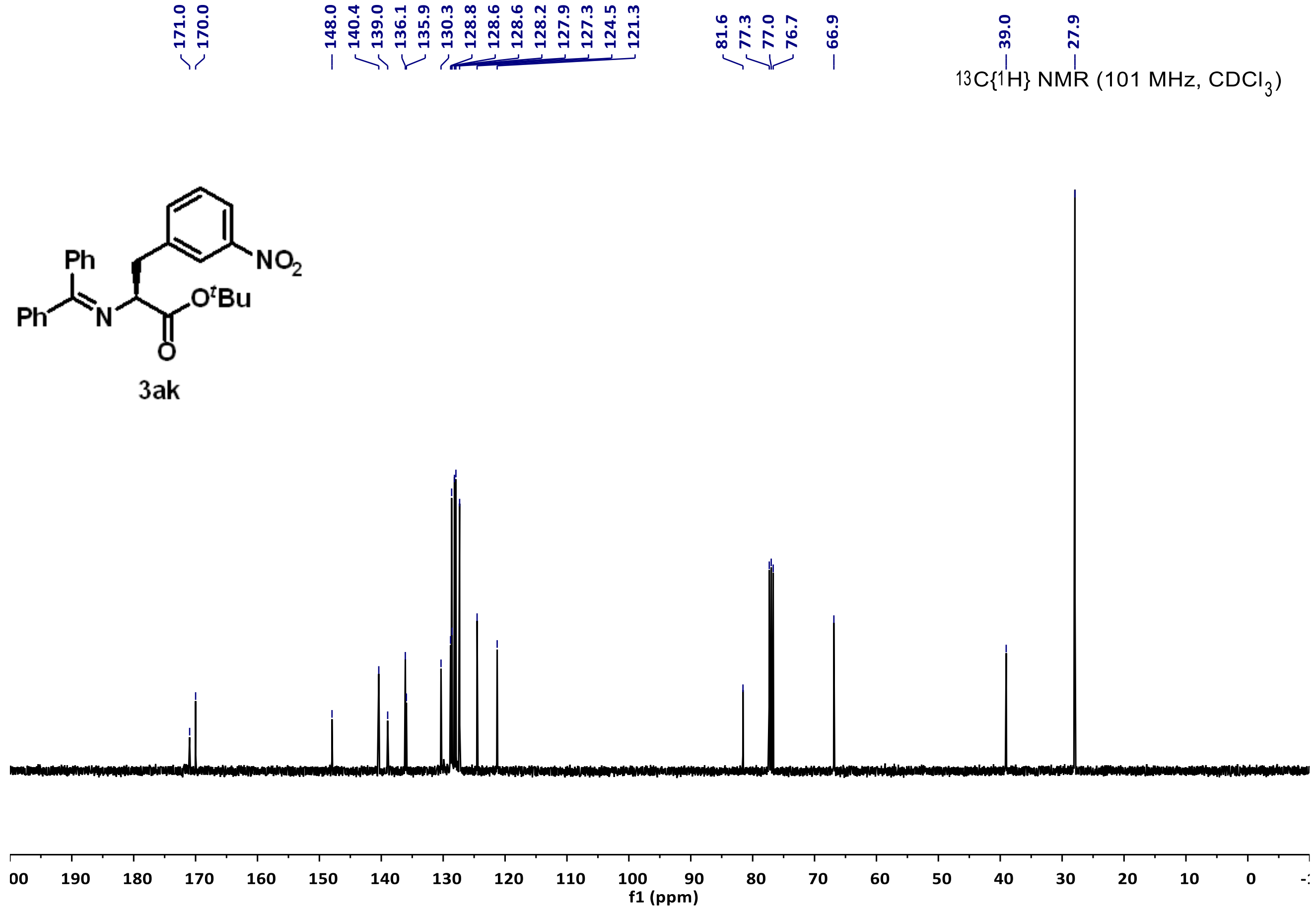

Supplementary Fig. 14. ^{13}C NMR spectrum of compound **3ak**

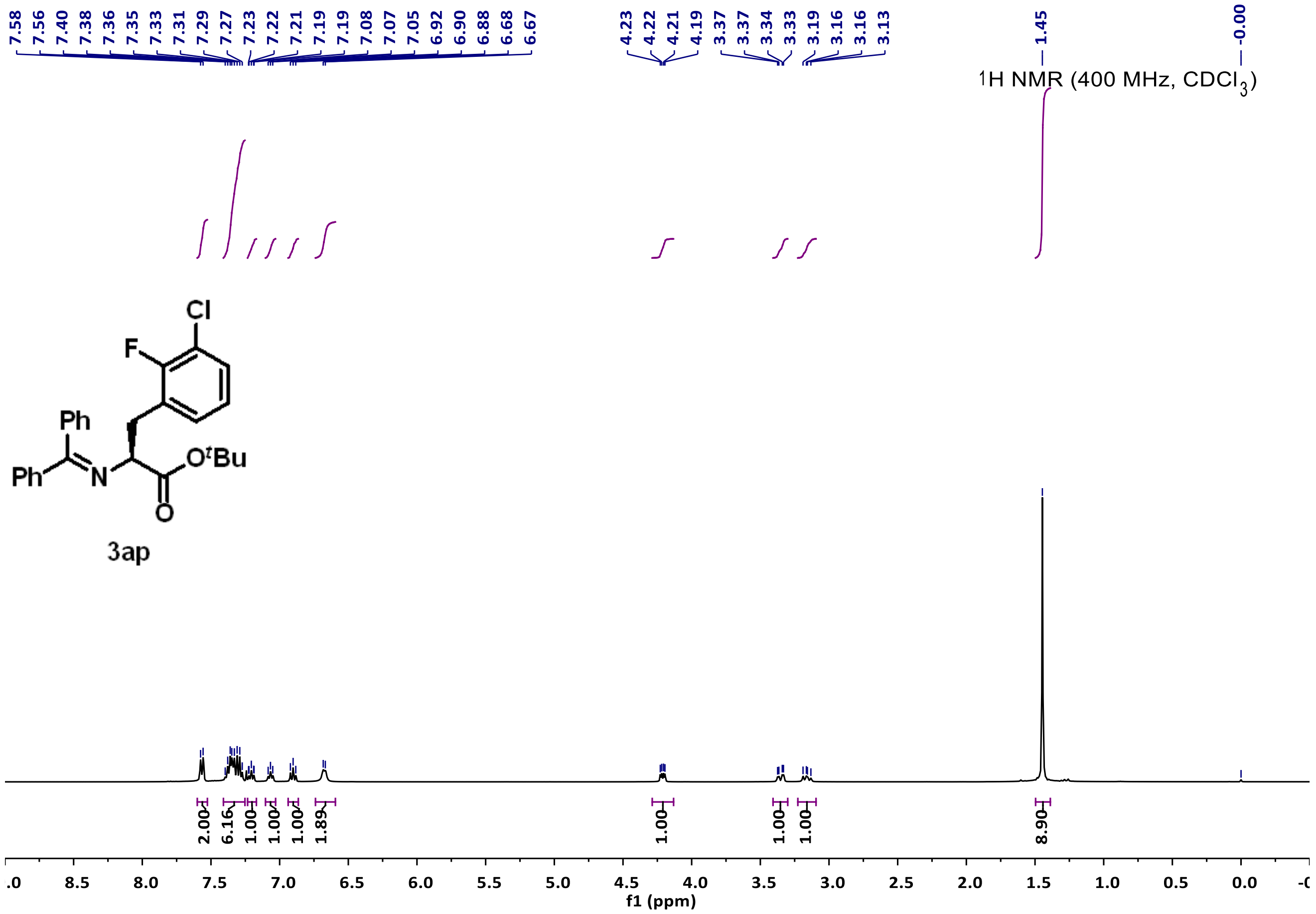

Supplementary Fig. 15. ^{1}H NMR spectrum of compound **3ap**

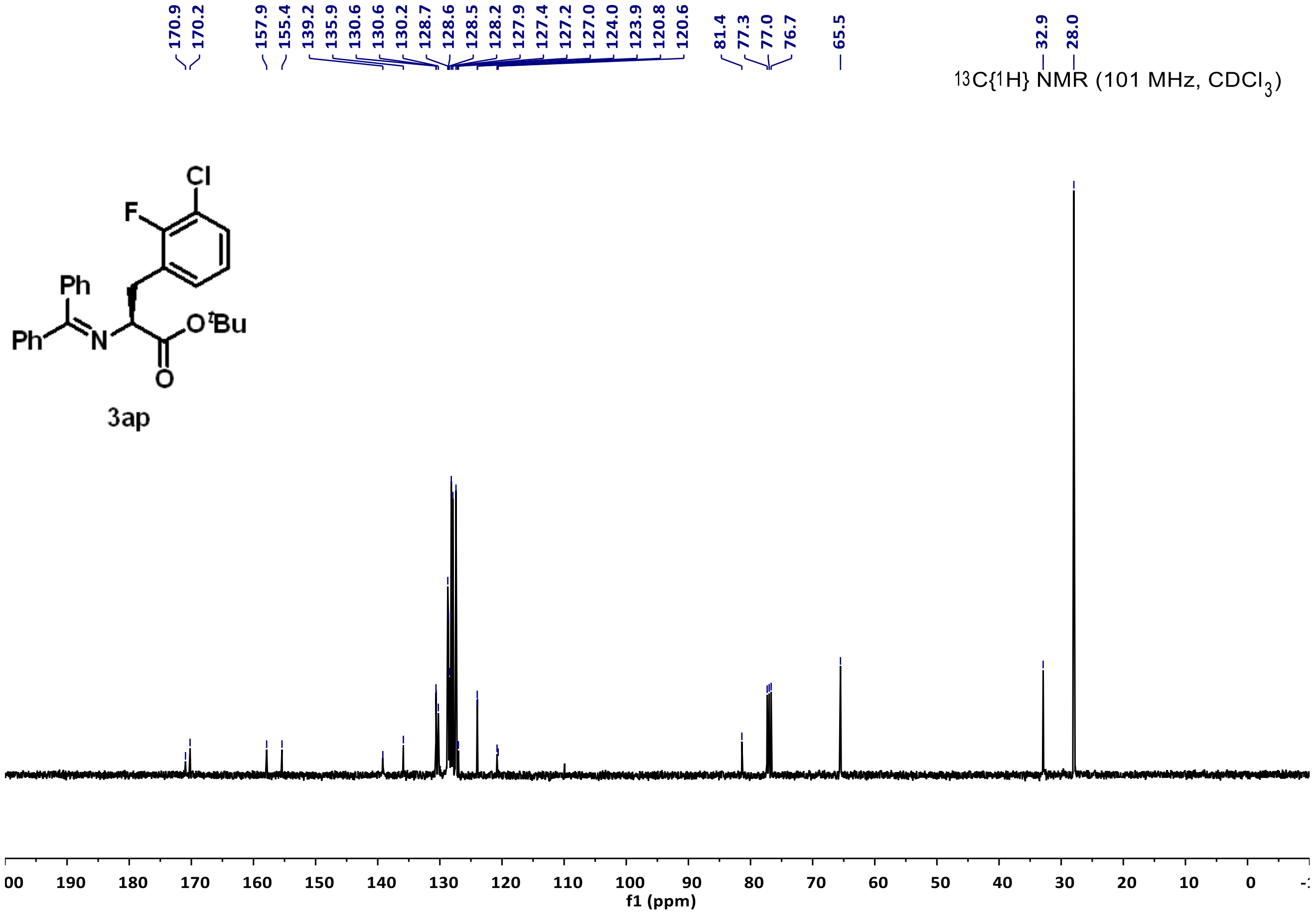

Supplementary Fig. 16. ^{13}C NMR spectrum of compound **3ap**

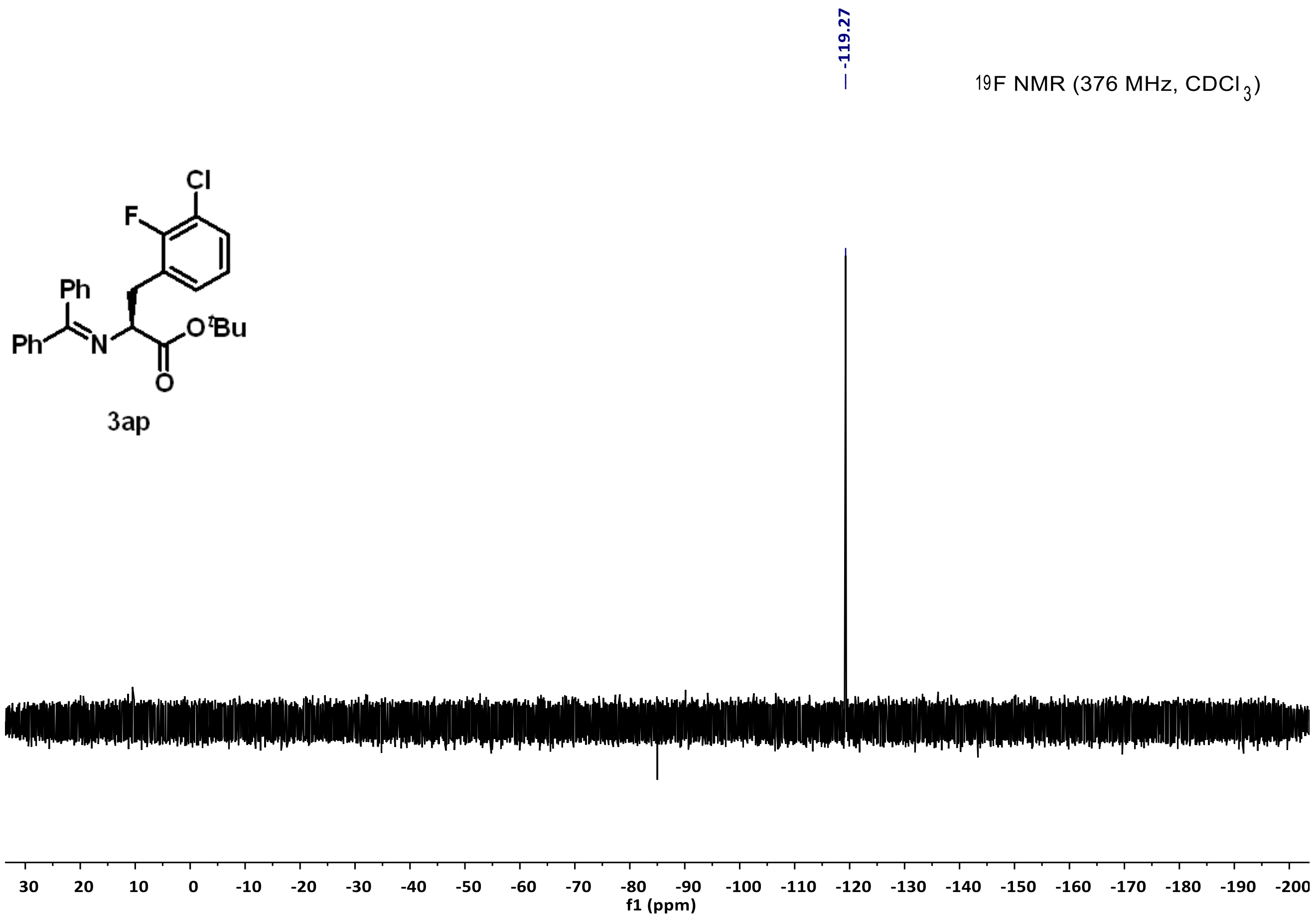

Supplementary Fig. 17. ^{19}F NMR spectrum of compound **3ap**

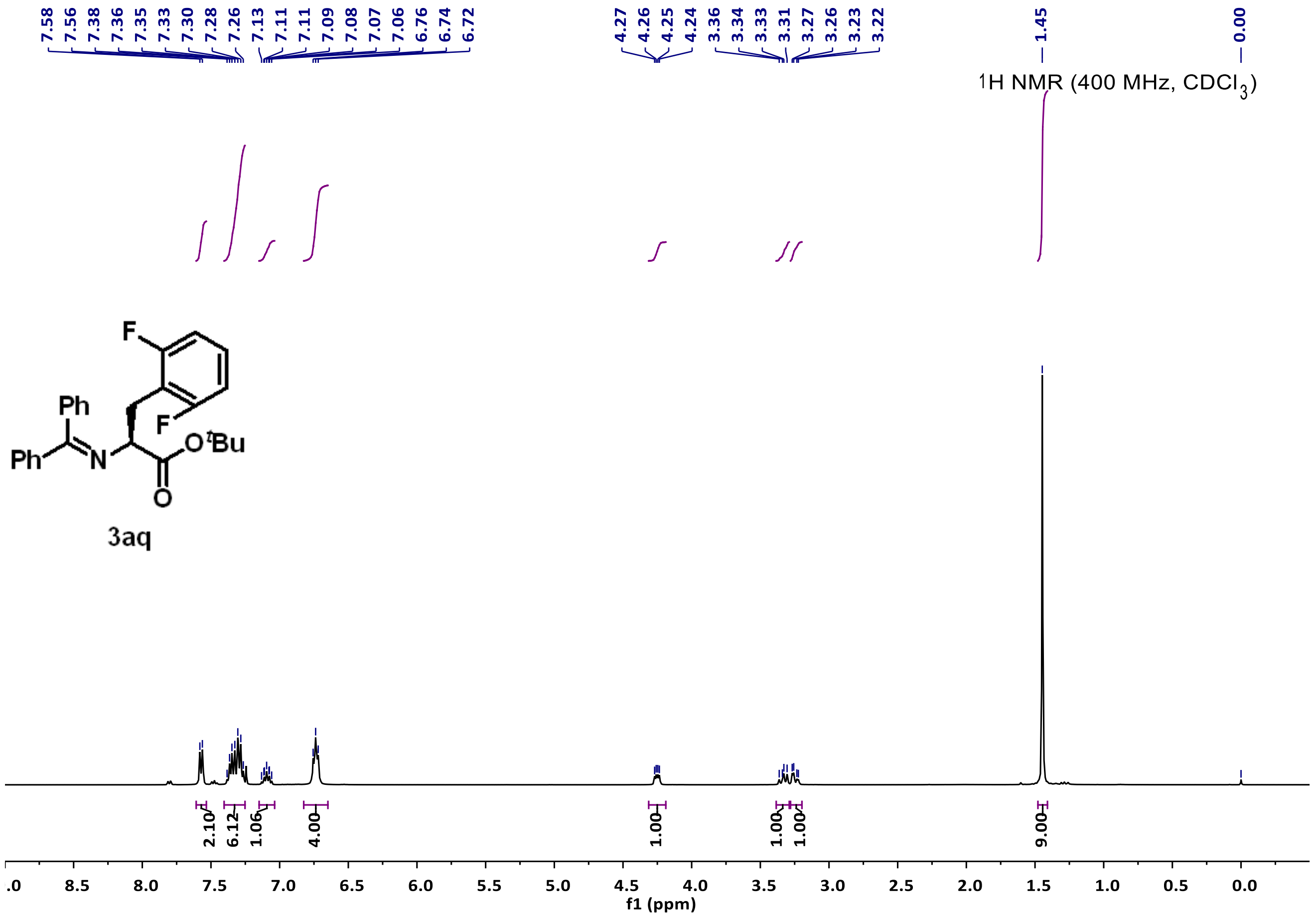

Supplementary Fig. 18. ^{1}H NMR spectrum of compound **3aq**

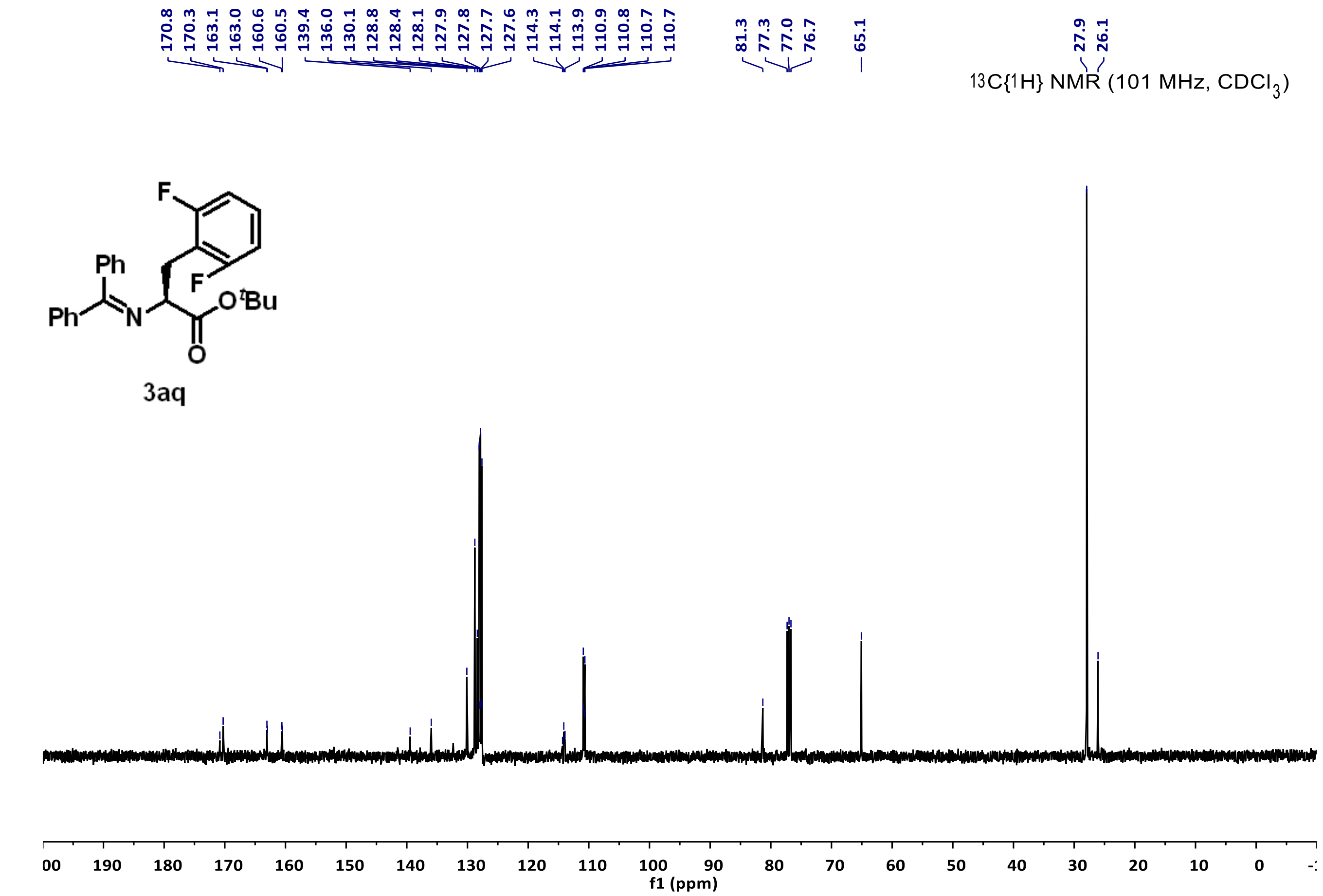

Supplementary Fig. 19. ^{13}C NMR spectrum of compound **3aq**

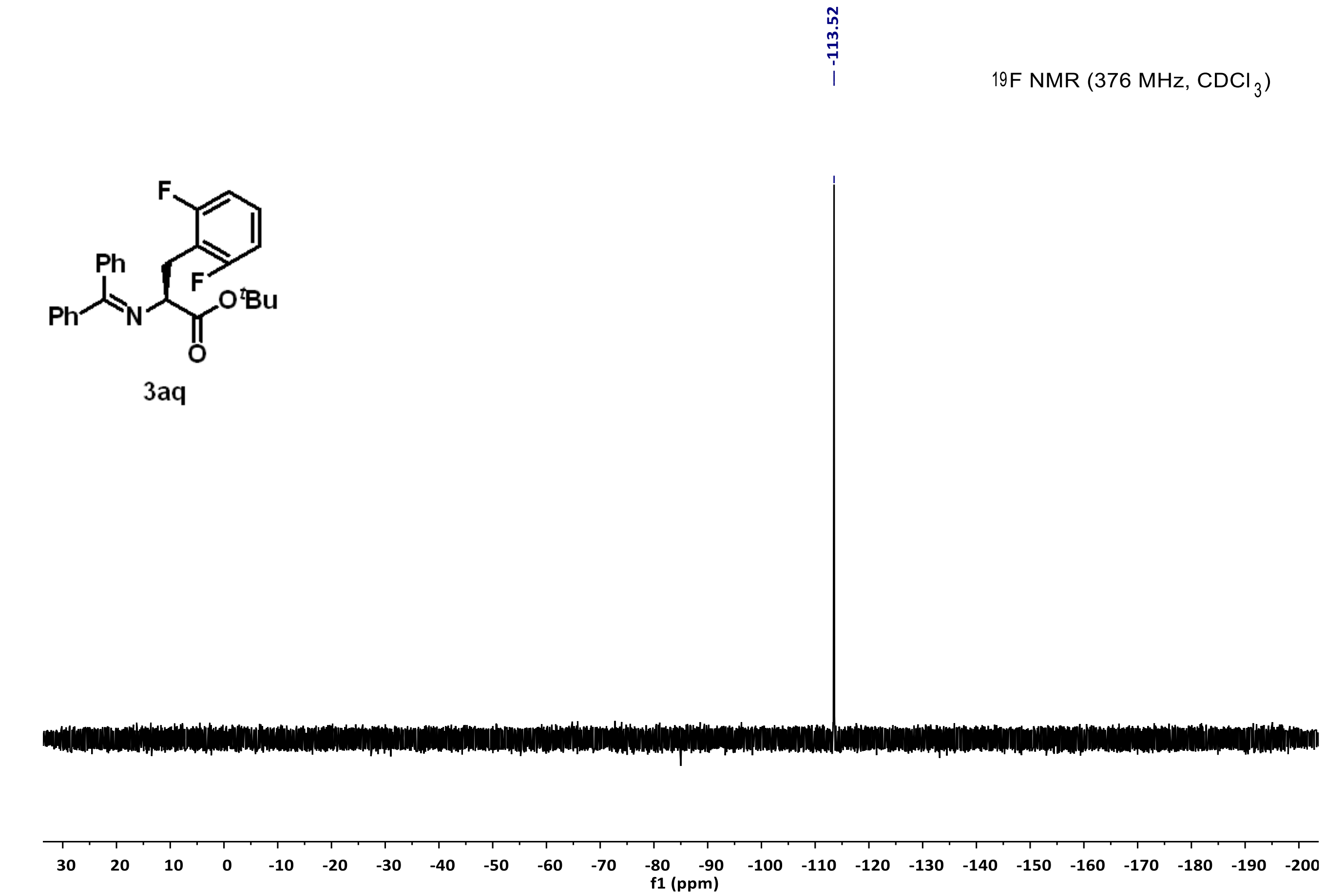

Supplementary Fig. 20. ^{19}F NMR spectrum of compound **3aq**

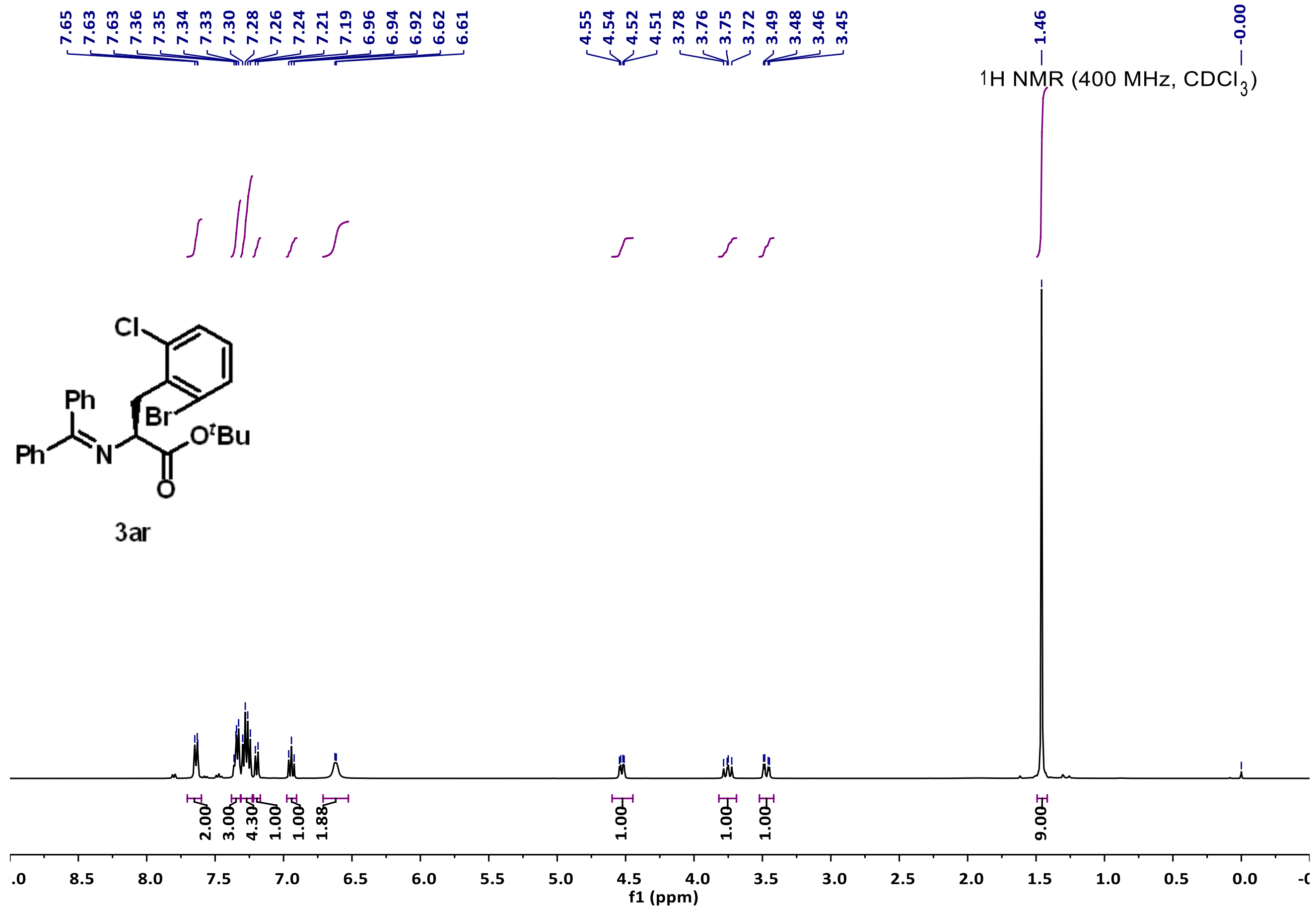

Supplementary Fig. 21. ^{1}H NMR spectrum of compound **3ar**

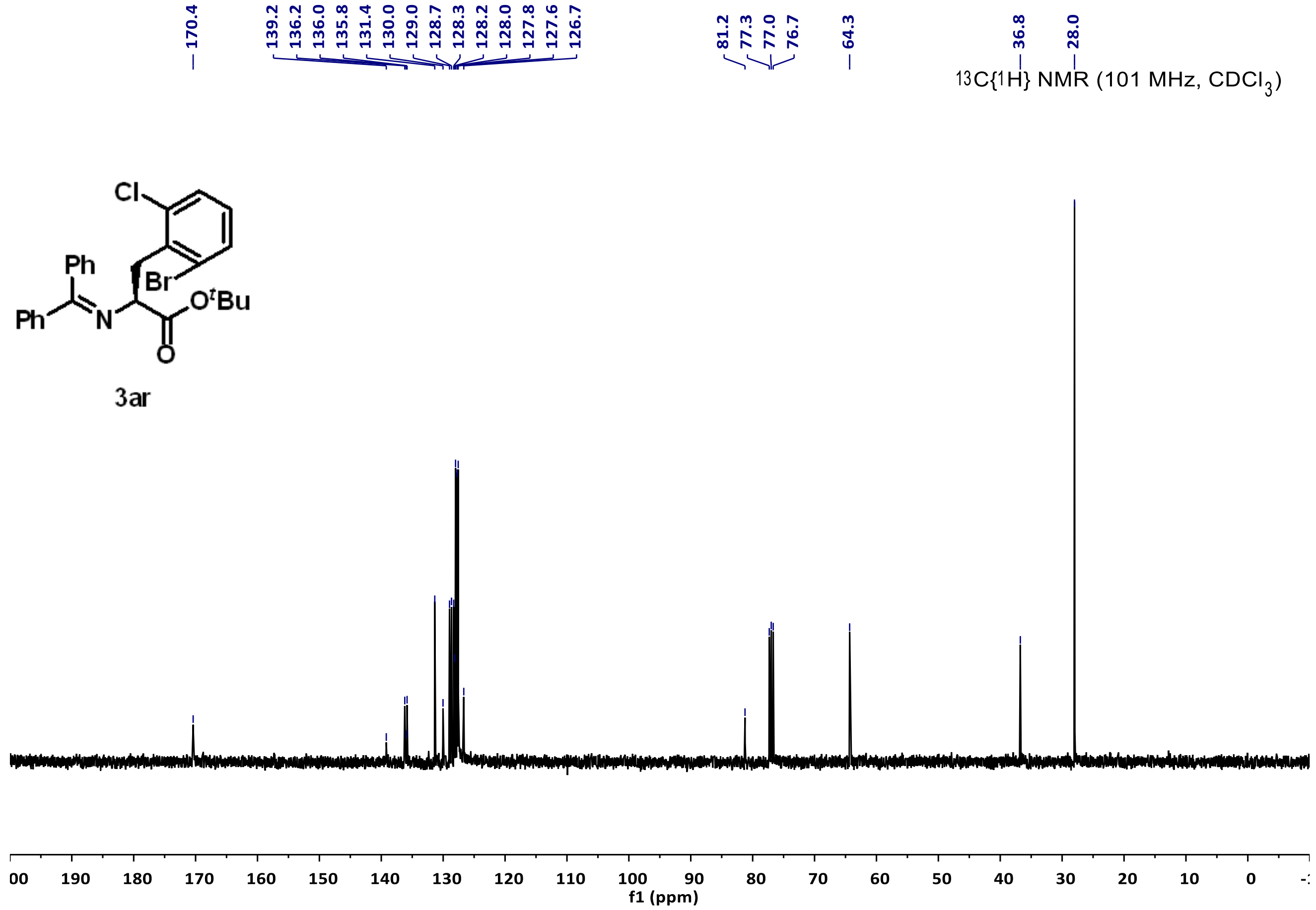

Supplementary Fig. 22. ^{13}C NMR spectrum of compound **3ar**

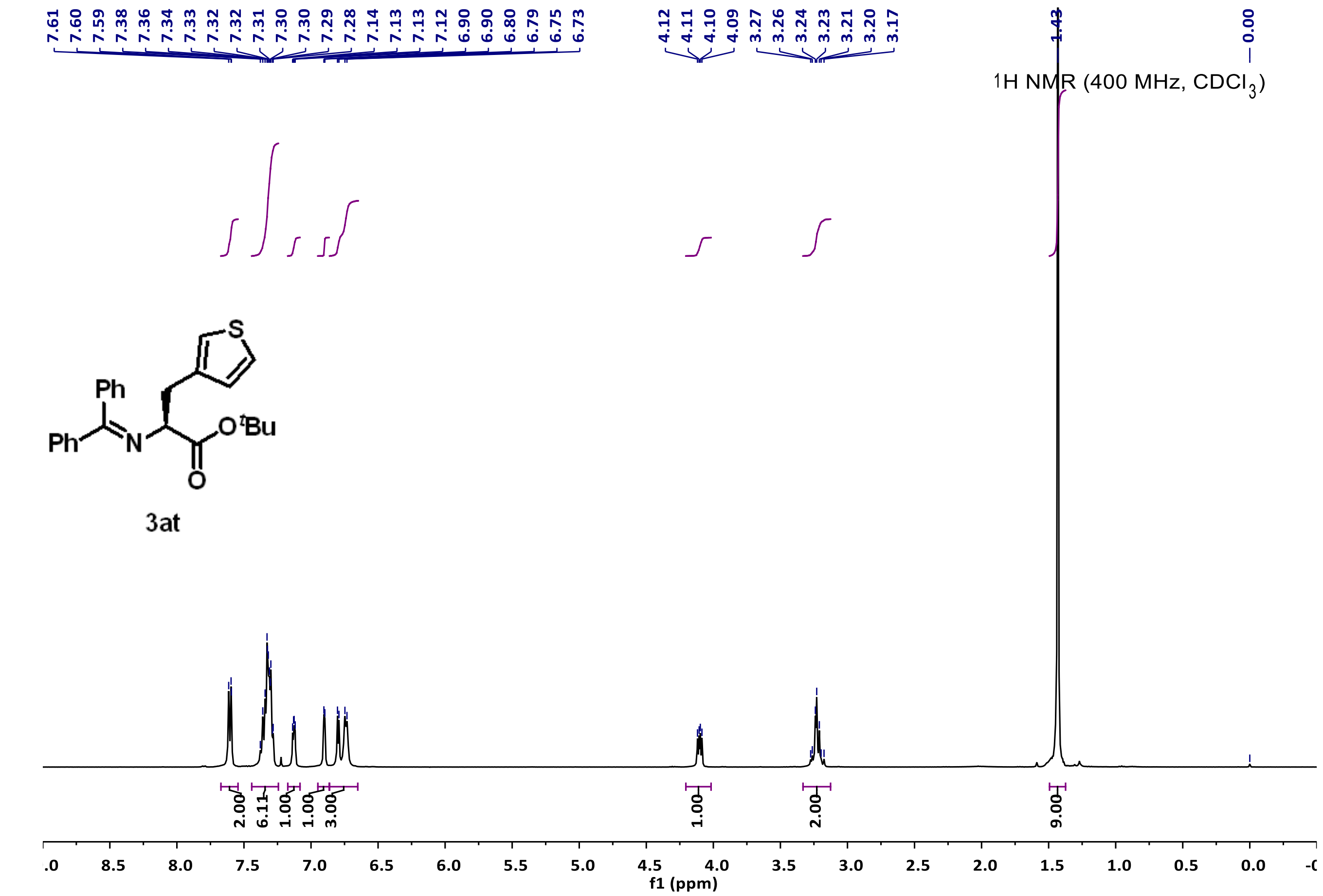

Supplementary Fig. 23. ^{1}H NMR spectrum of compound **3at**

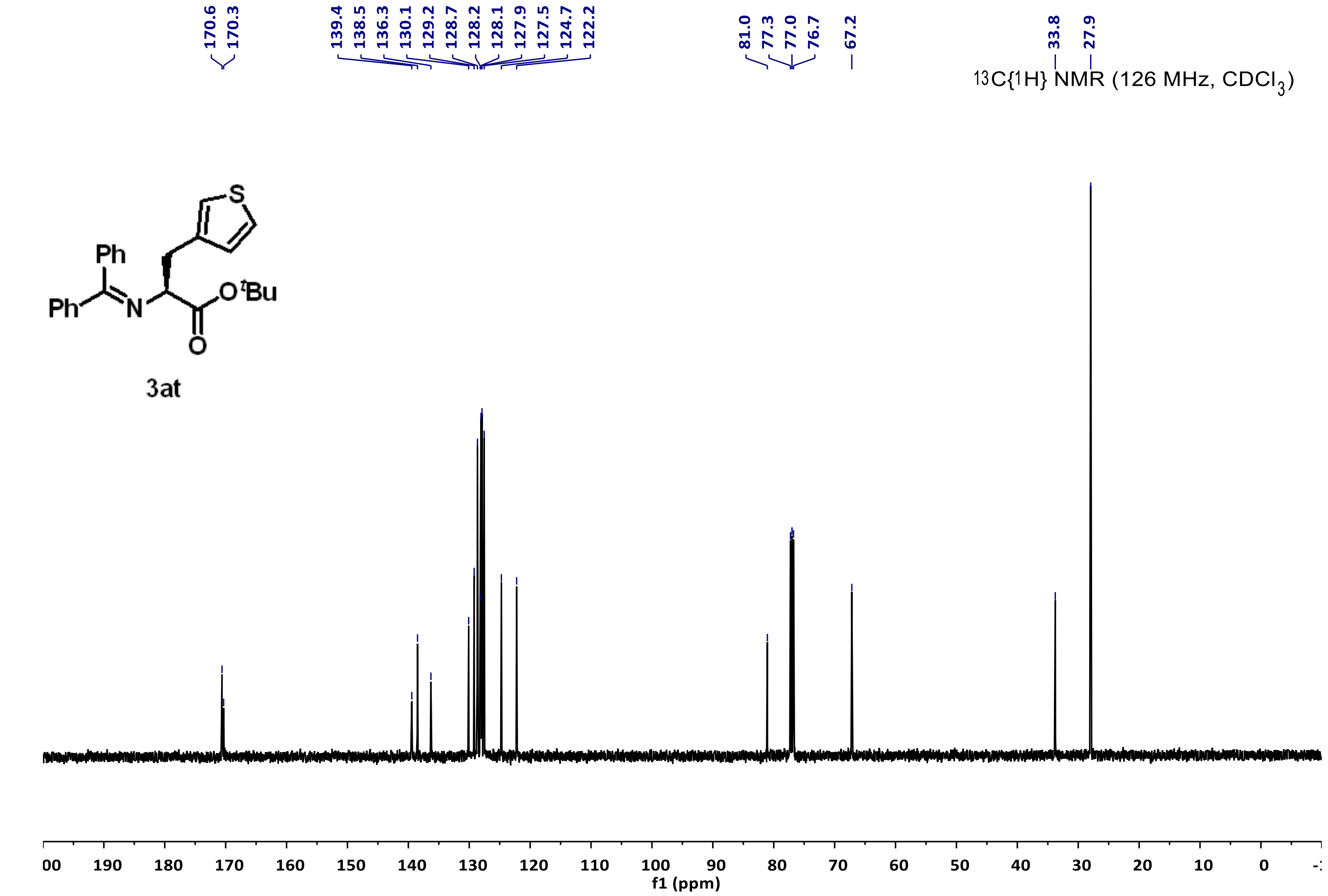

Supplementary Fig. 24. ^{13}C NMR spectrum of compound **3at**

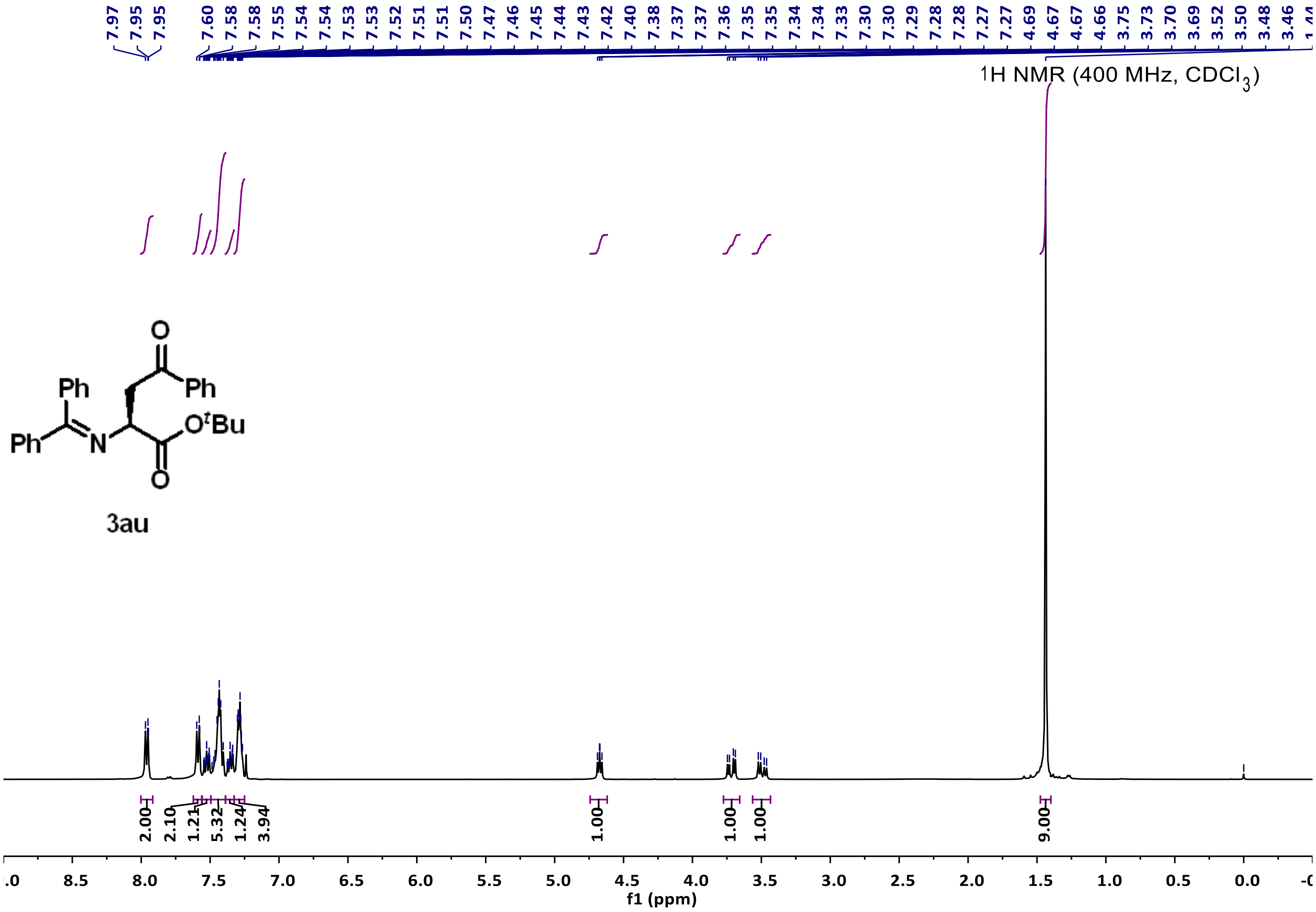

Supplementary Fig. 25. ^{1}H NMR spectrum of compound **3au**

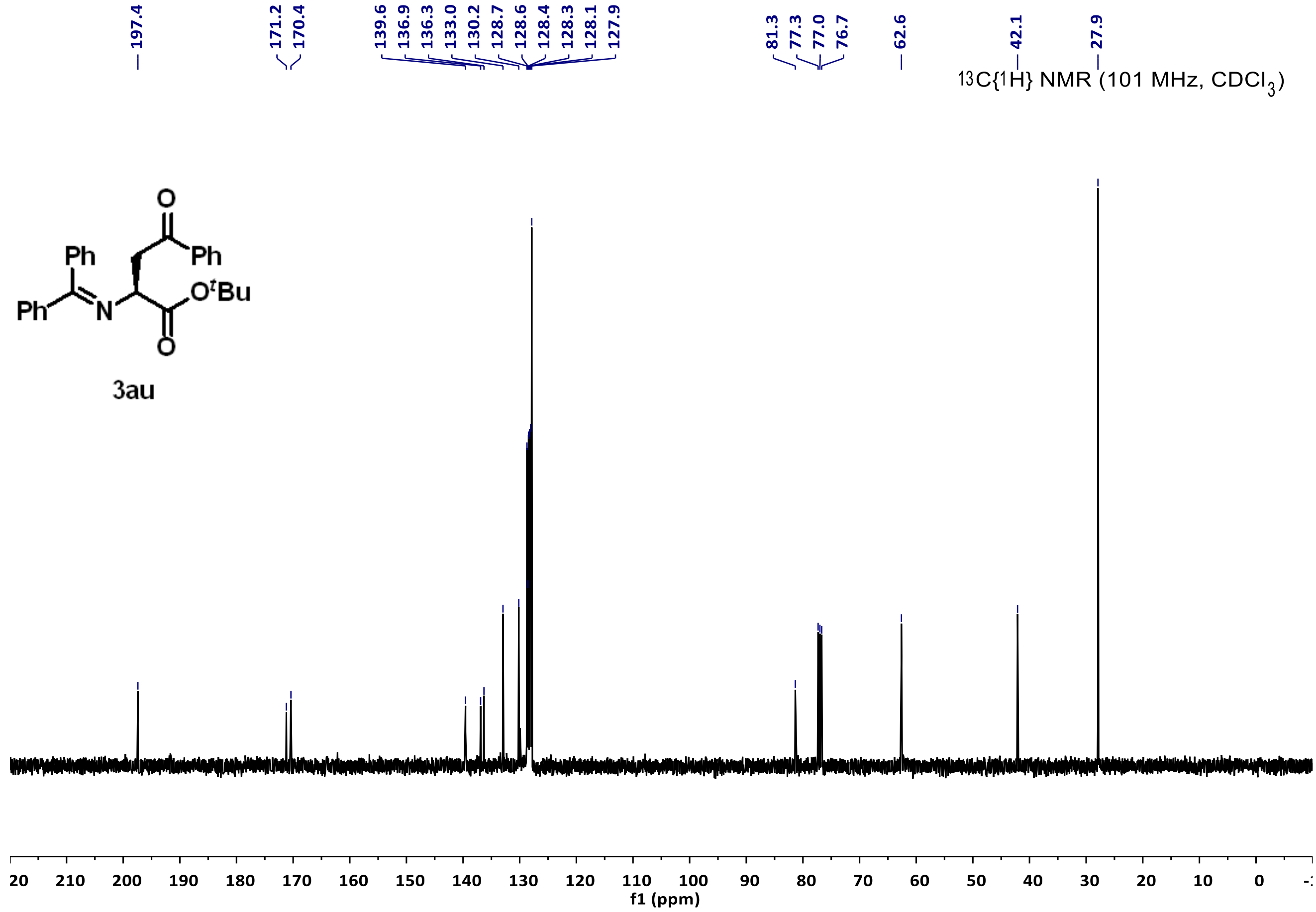

Supplementary Fig. 26. ^{13}C NMR spectrum of compound **3au**

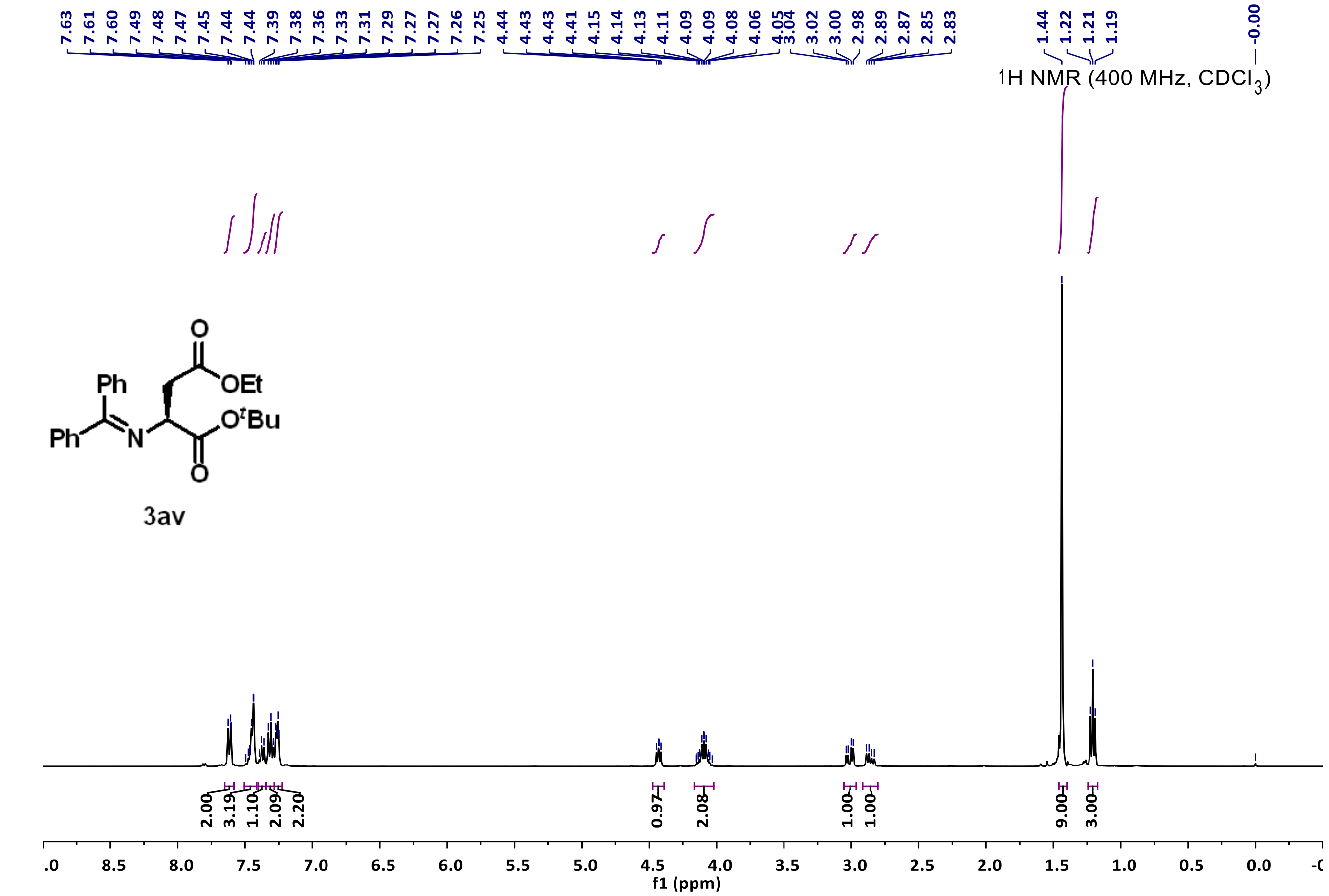

Supplementary Fig. 27. ^{1}H NMR spectrum of compound **3av**

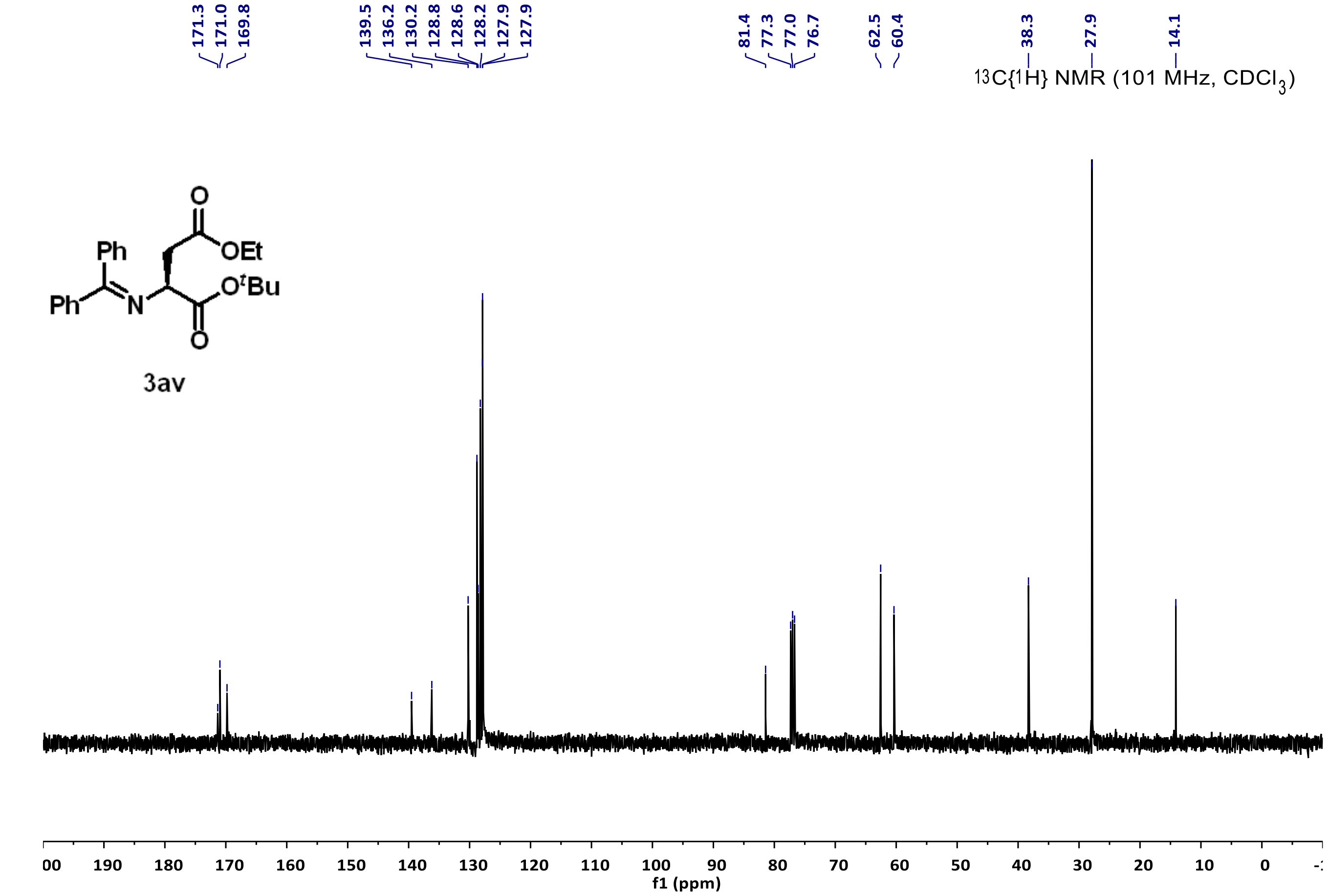

Supplementary Fig. 28. ^{13}C NMR spectrum of compound **3av**

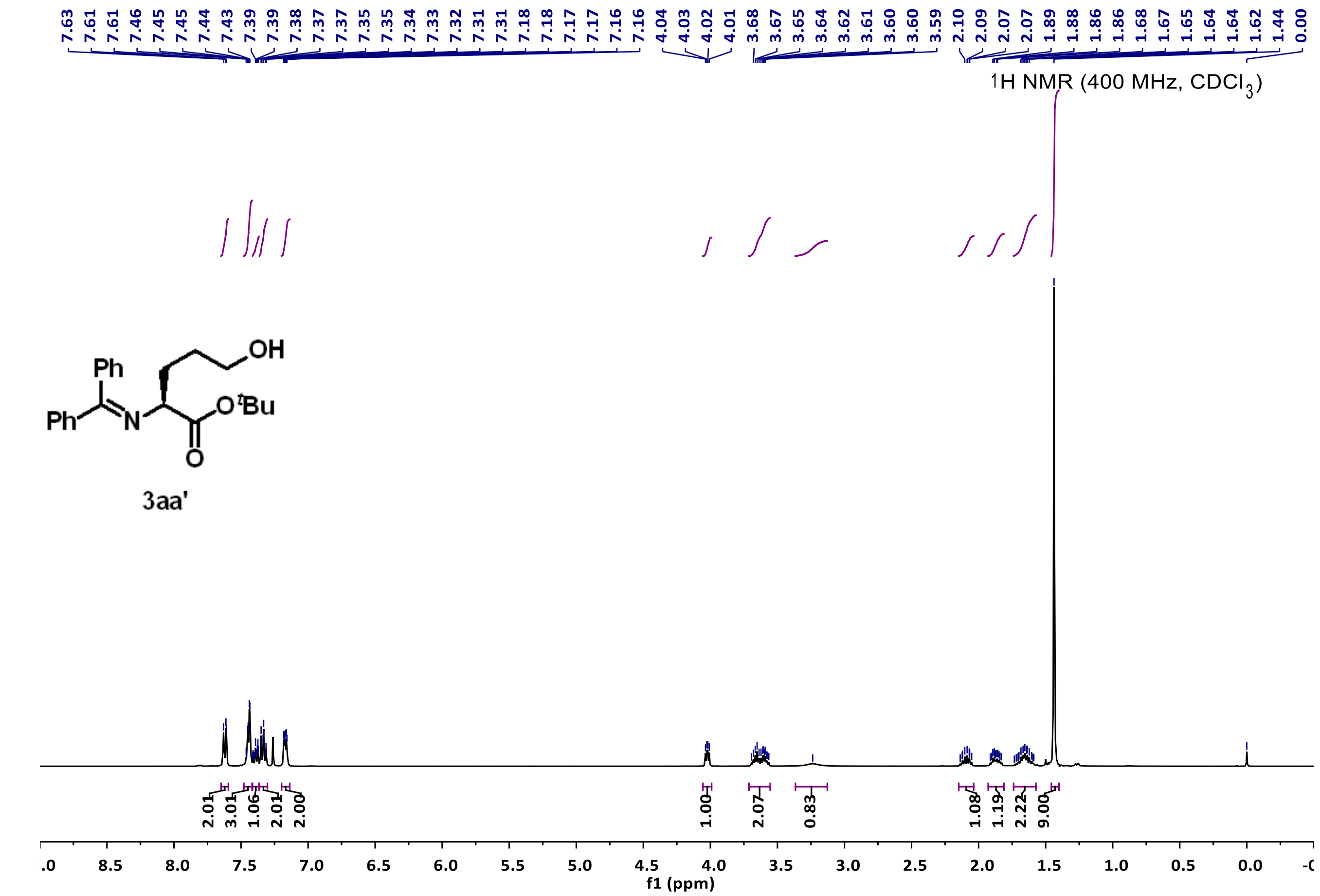

Supplementary Fig. 29. ^{1}H NMR spectrum of compound **3aa'**

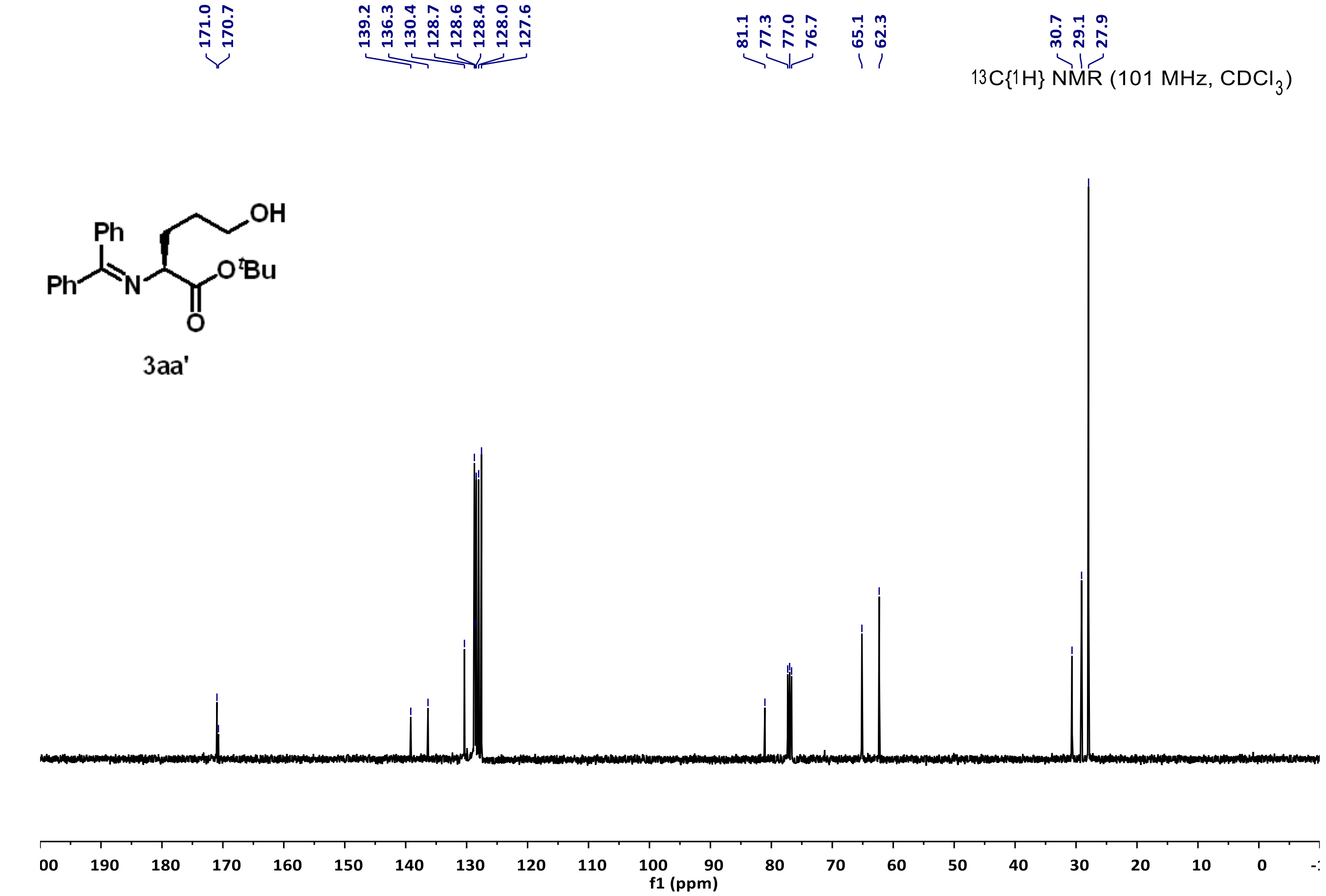

Supplementary Fig. 30. ^{13}C NMR spectrum of compound **3aa'**

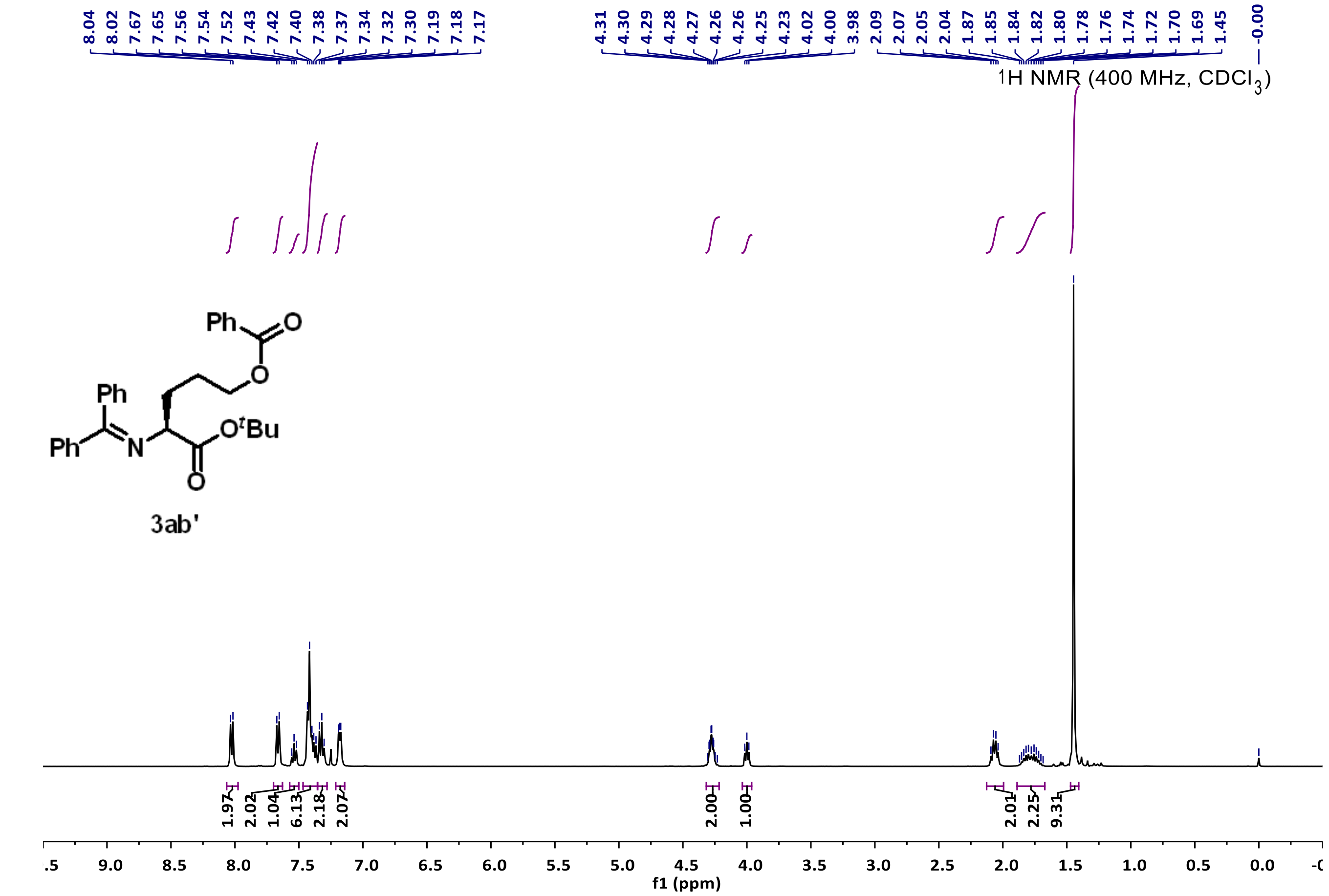

Supplementary Fig. 31. ^{1}H NMR spectrum of compound **3ab'**

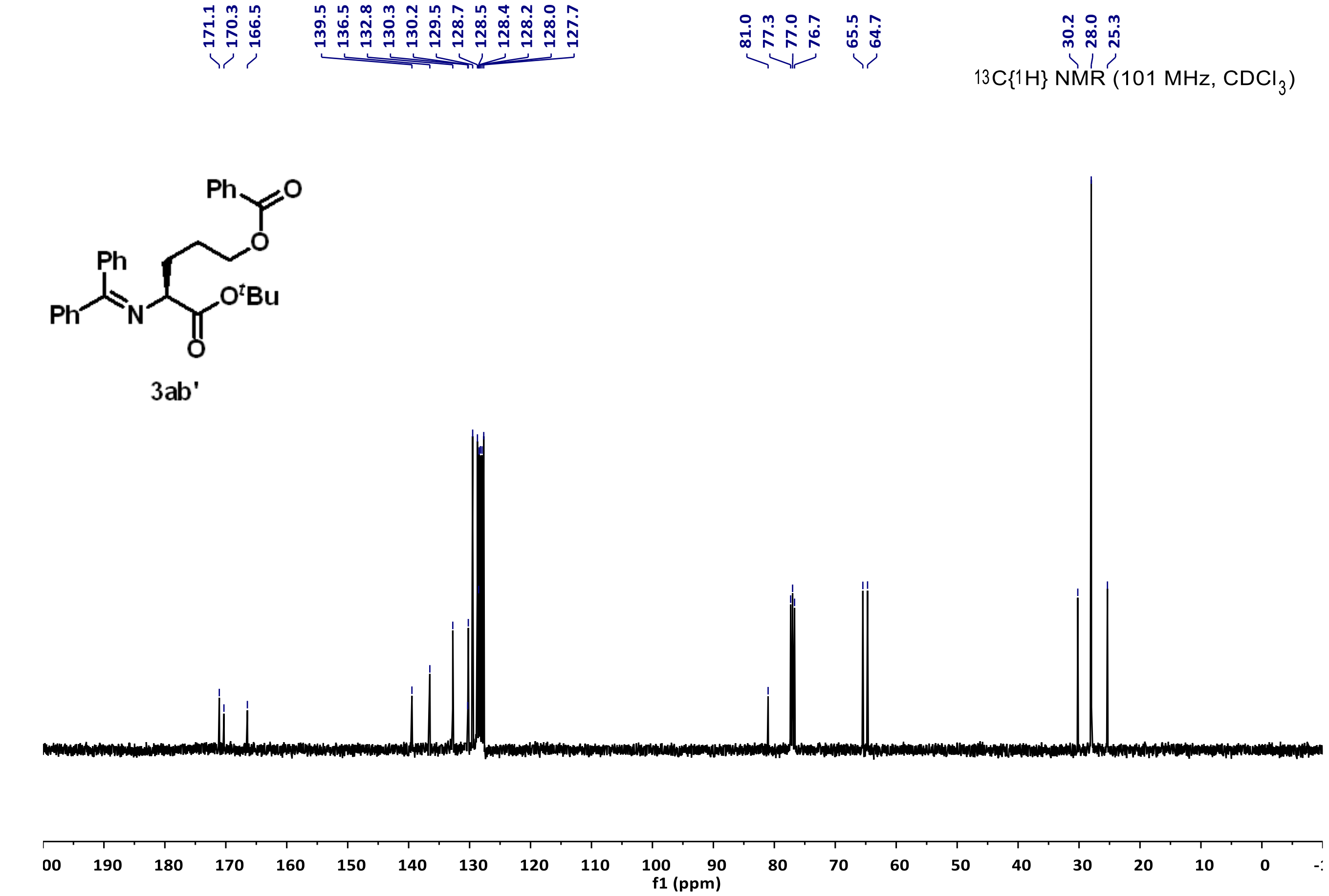

Supplementary Fig. 32. ^{13}C NMR spectrum of compound **3ab'**

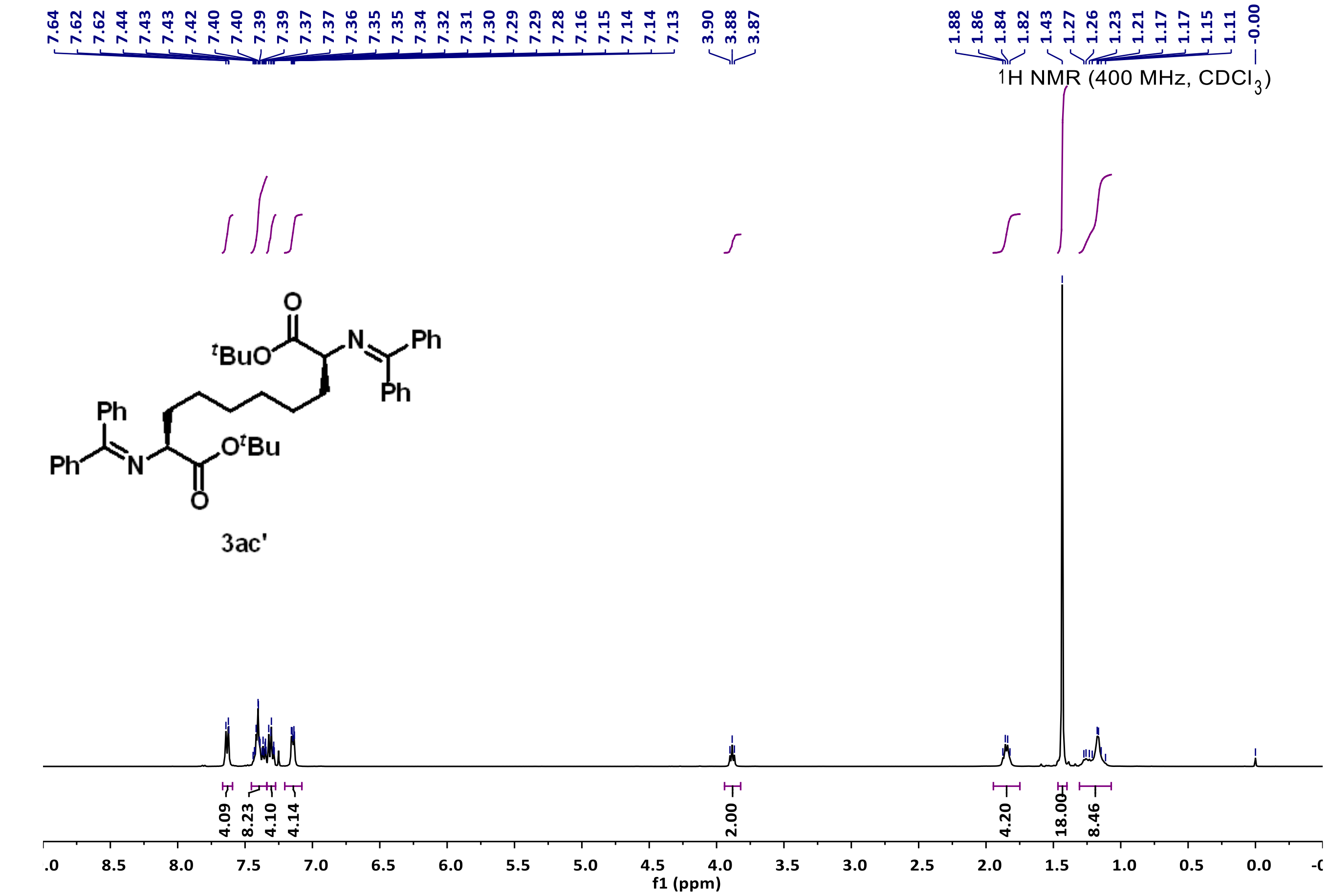

Supplementary Fig. 33. ^{1}H NMR spectrum of compound **3ac'**

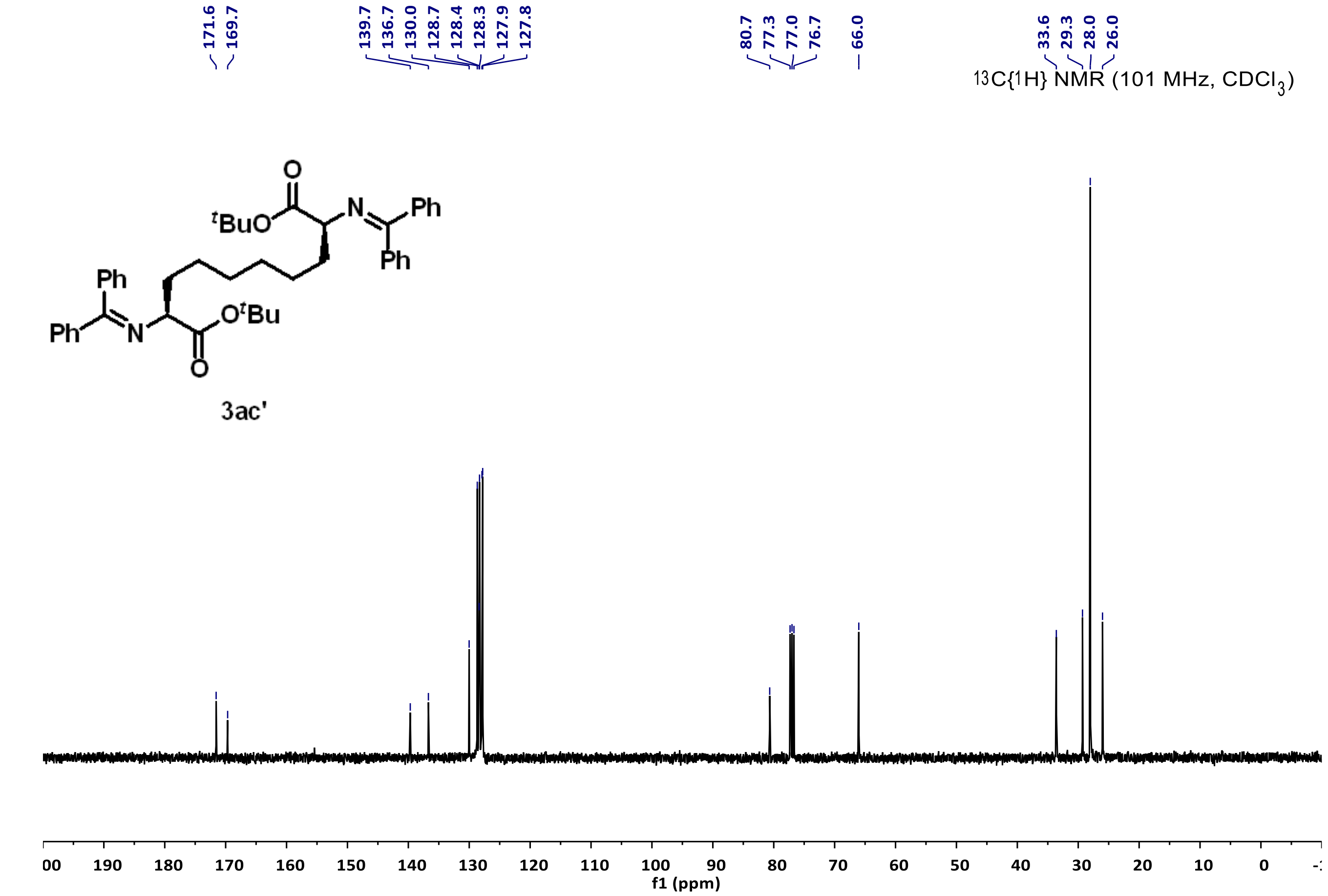

Supplementary Fig. 34. ^{13}C NMR spectrum of compound **3ac'**

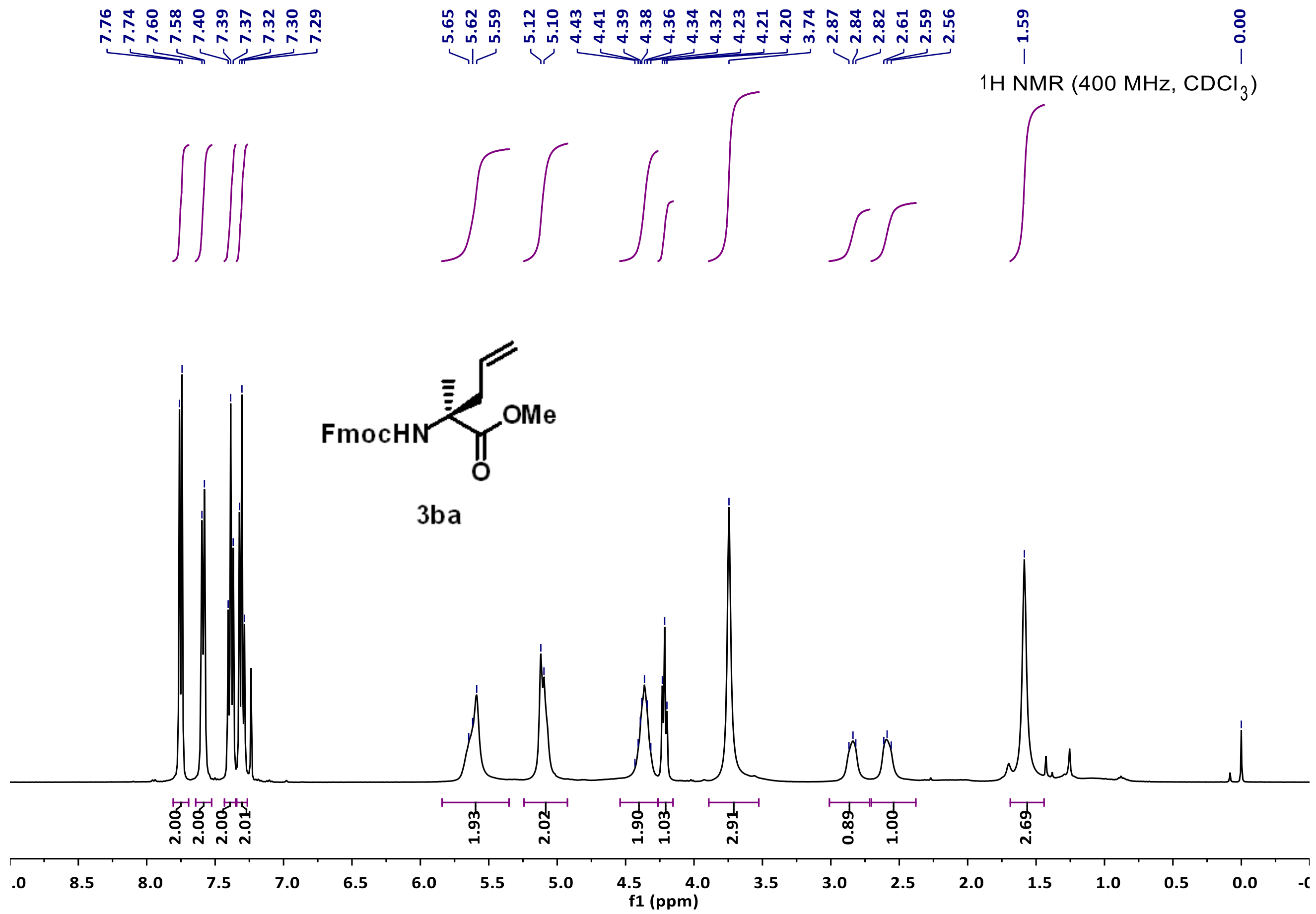

Supplementary Fig. 35. ^{1}H NMR spectrum of compound **3ba**

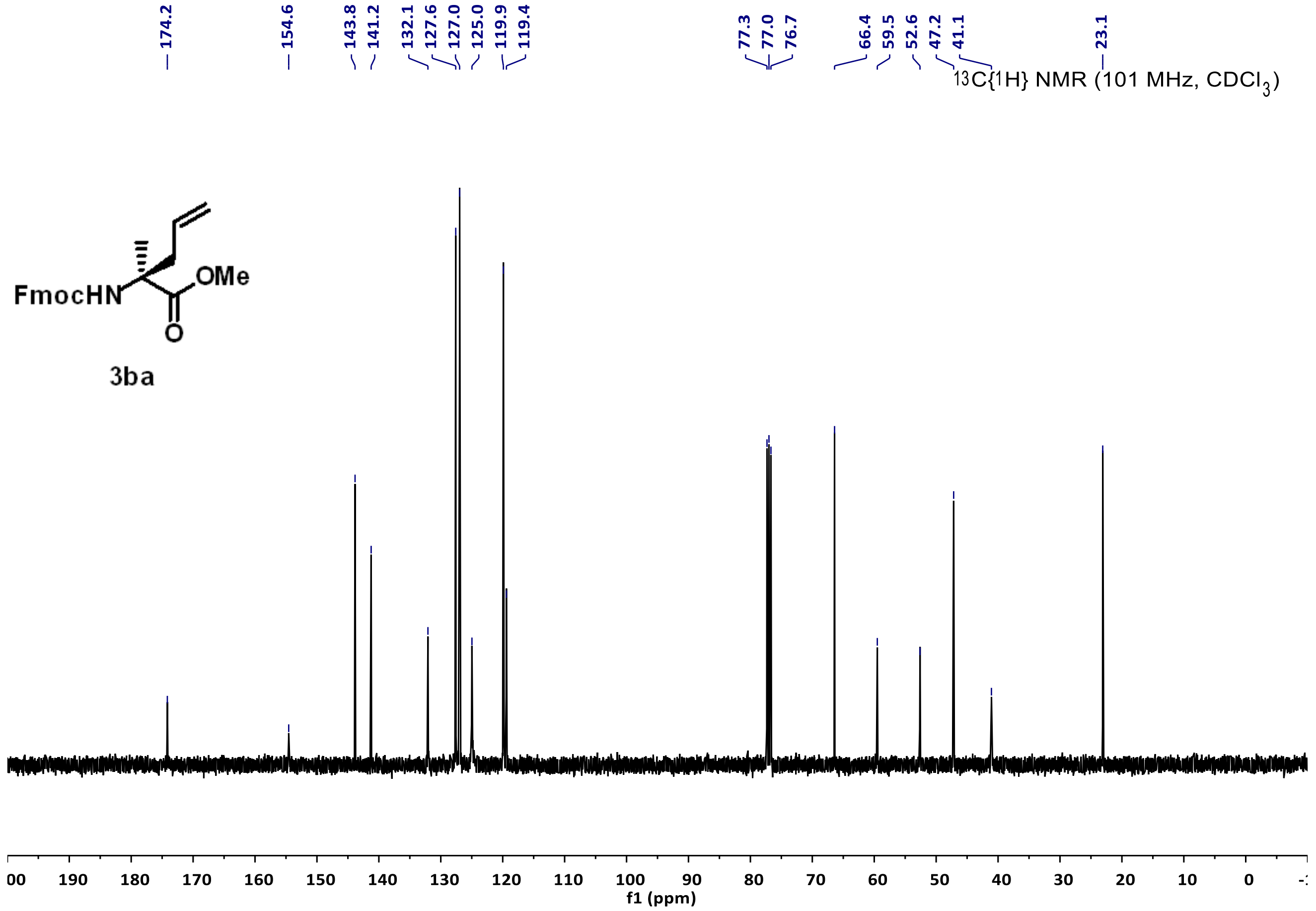

Supplementary Fig. 36. ^{13}C NMR spectrum of compound **3ba**

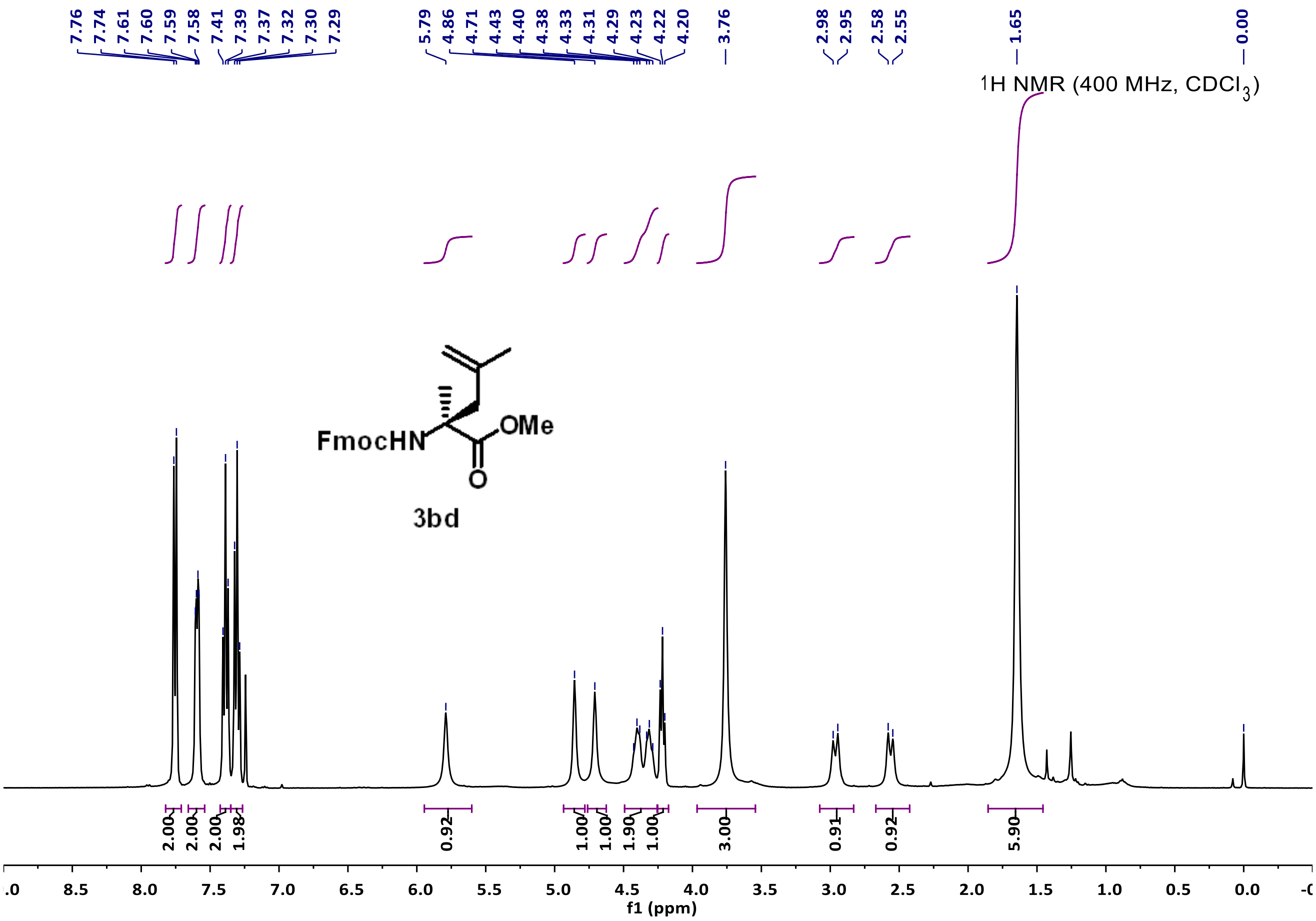

Supplementary Fig. 37. ^{1}H NMR spectrum of compound **3bd**

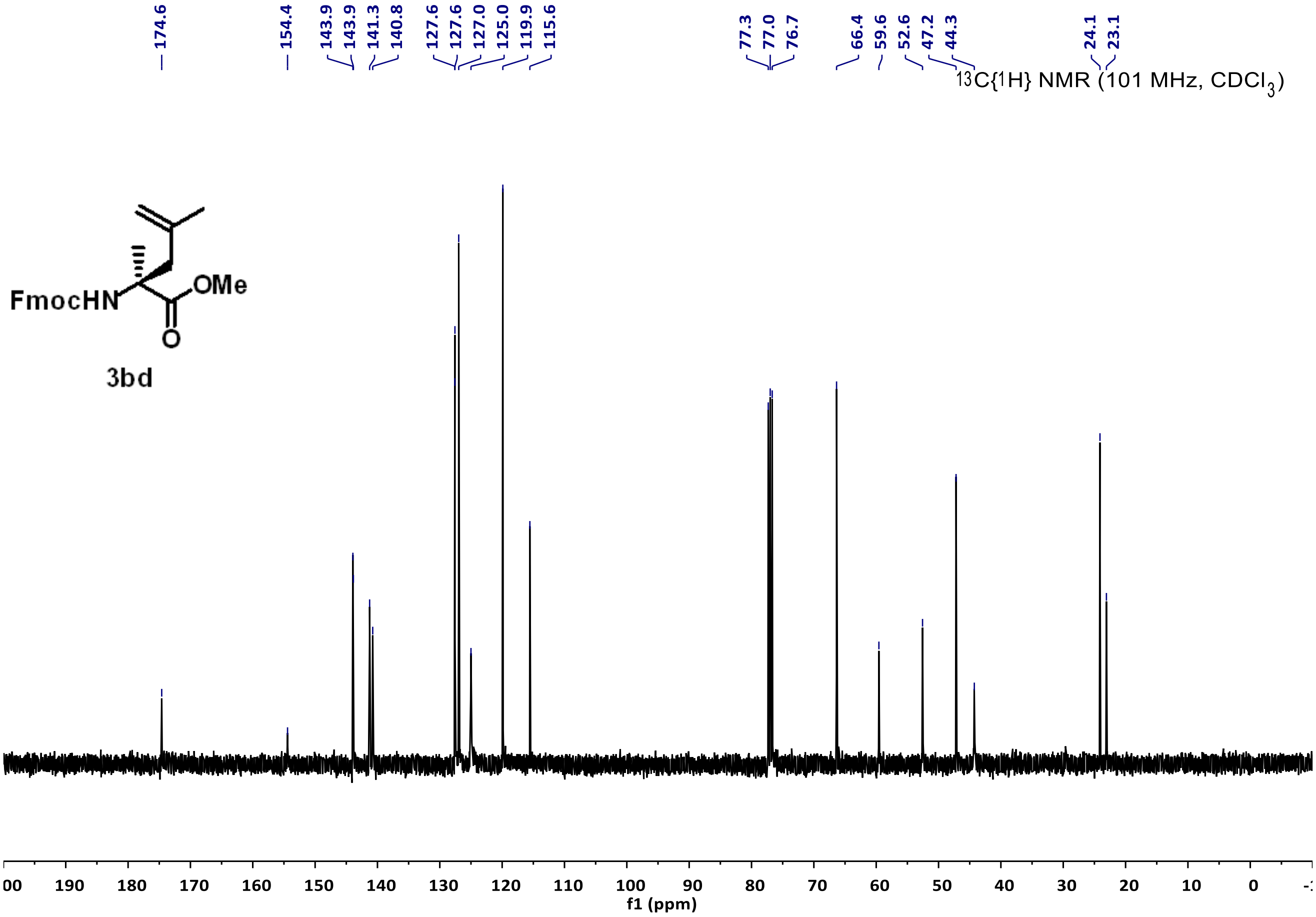

Supplementary Fig. 38. ^{13}C NMR spectrum of compound **3bd**

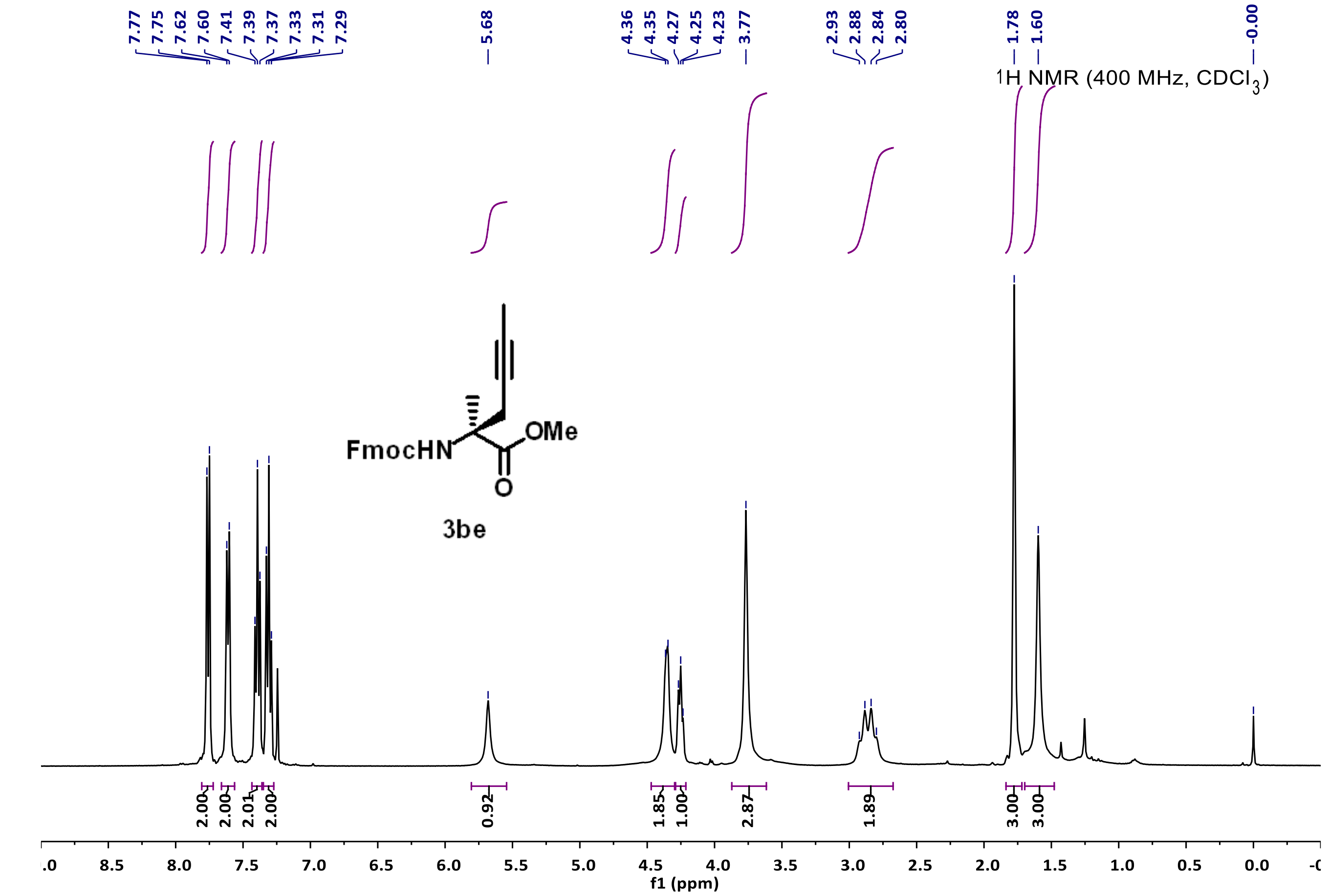

Supplementary Fig. 39. ^{1}H NMR spectrum of compound **3be**

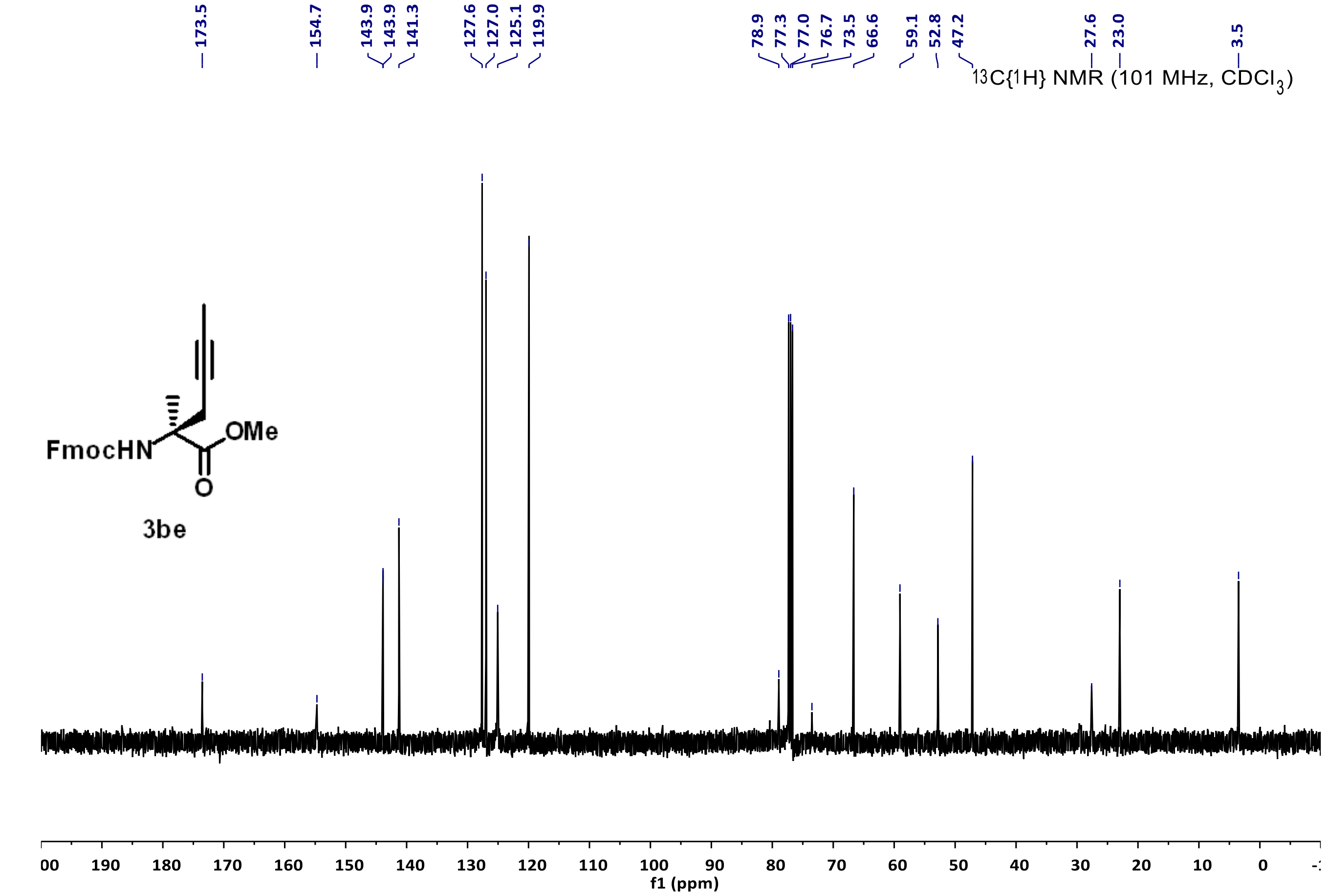

Supplementary Fig. 40. ^{13}C NMR spectrum of compound **3be**

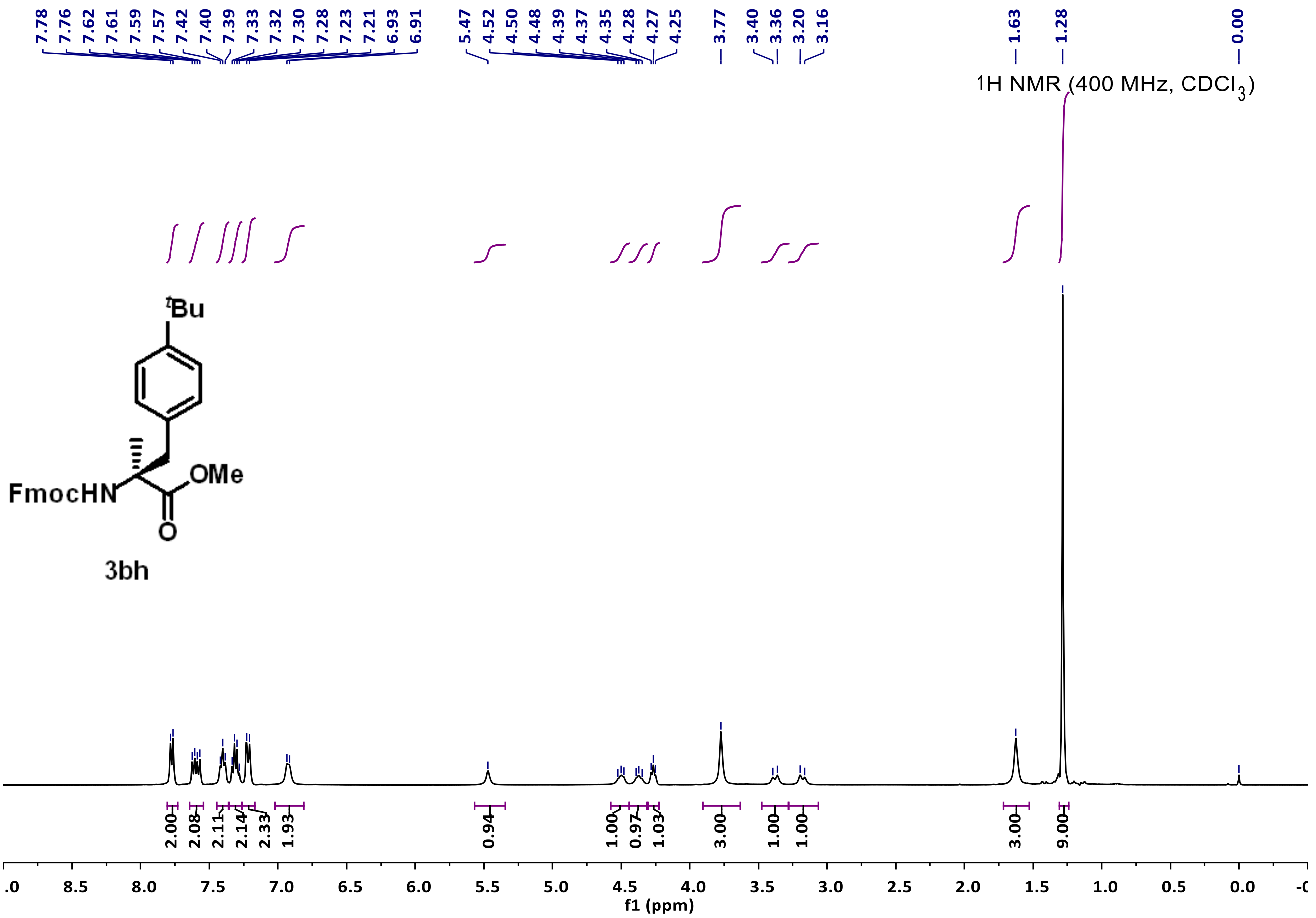

Supplementary Fig. 41. ^{1}H NMR spectrum of compound **3bh**

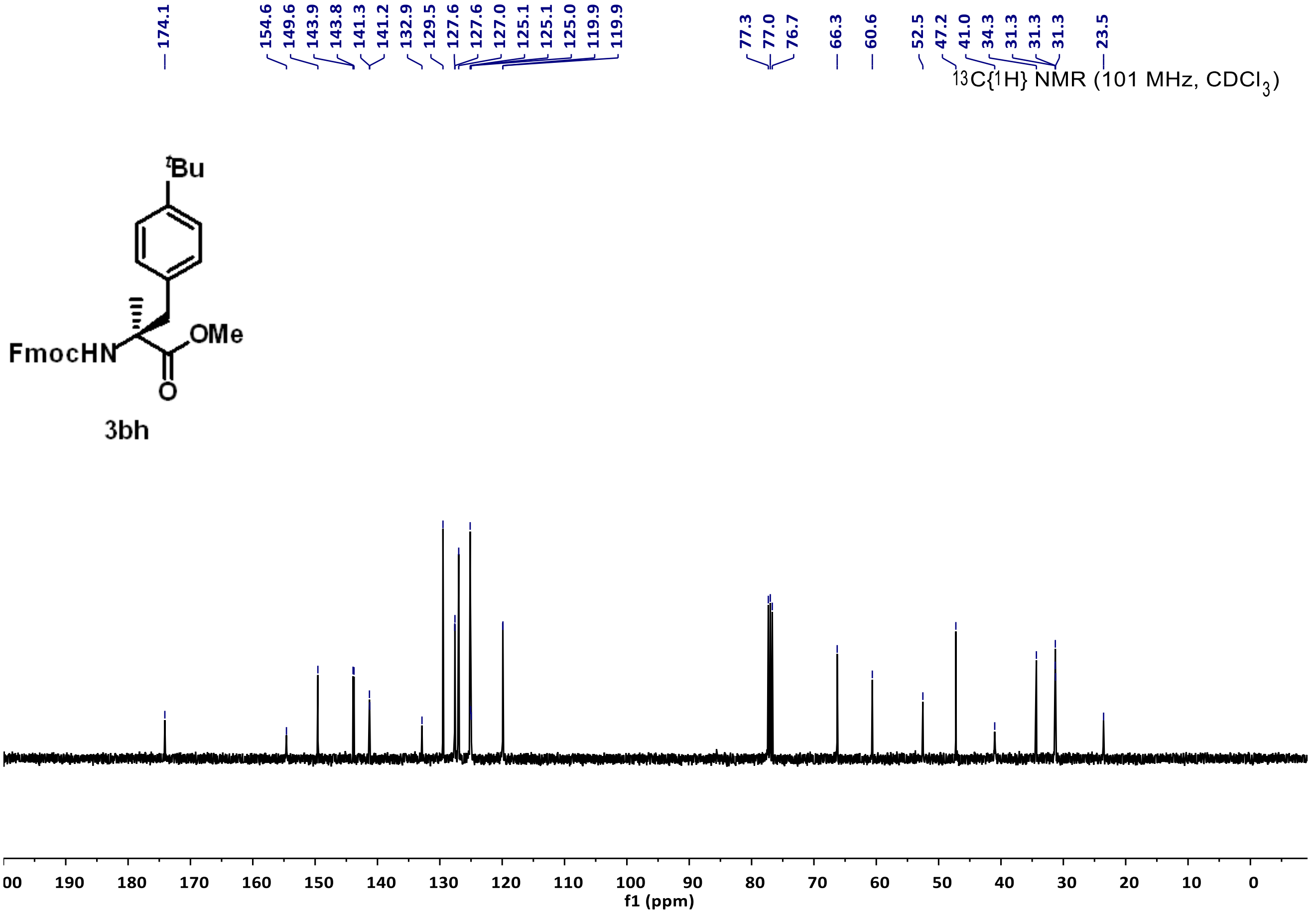

Supplementary Fig. 42. ^{13}C NMR spectrum of compound **3bh**

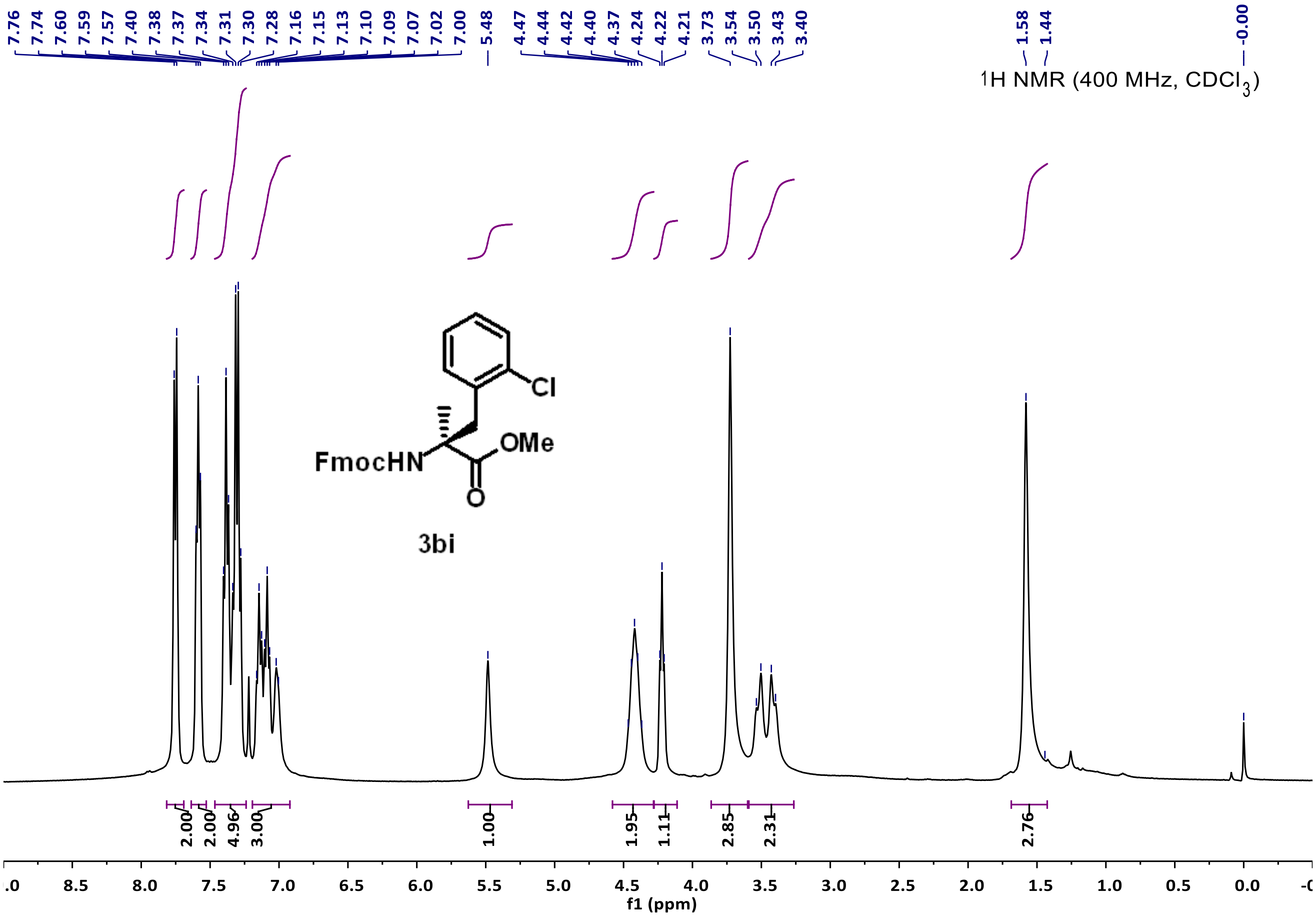

Supplementary Fig. 43. ^{1}H NMR spectrum of compound **3bi**

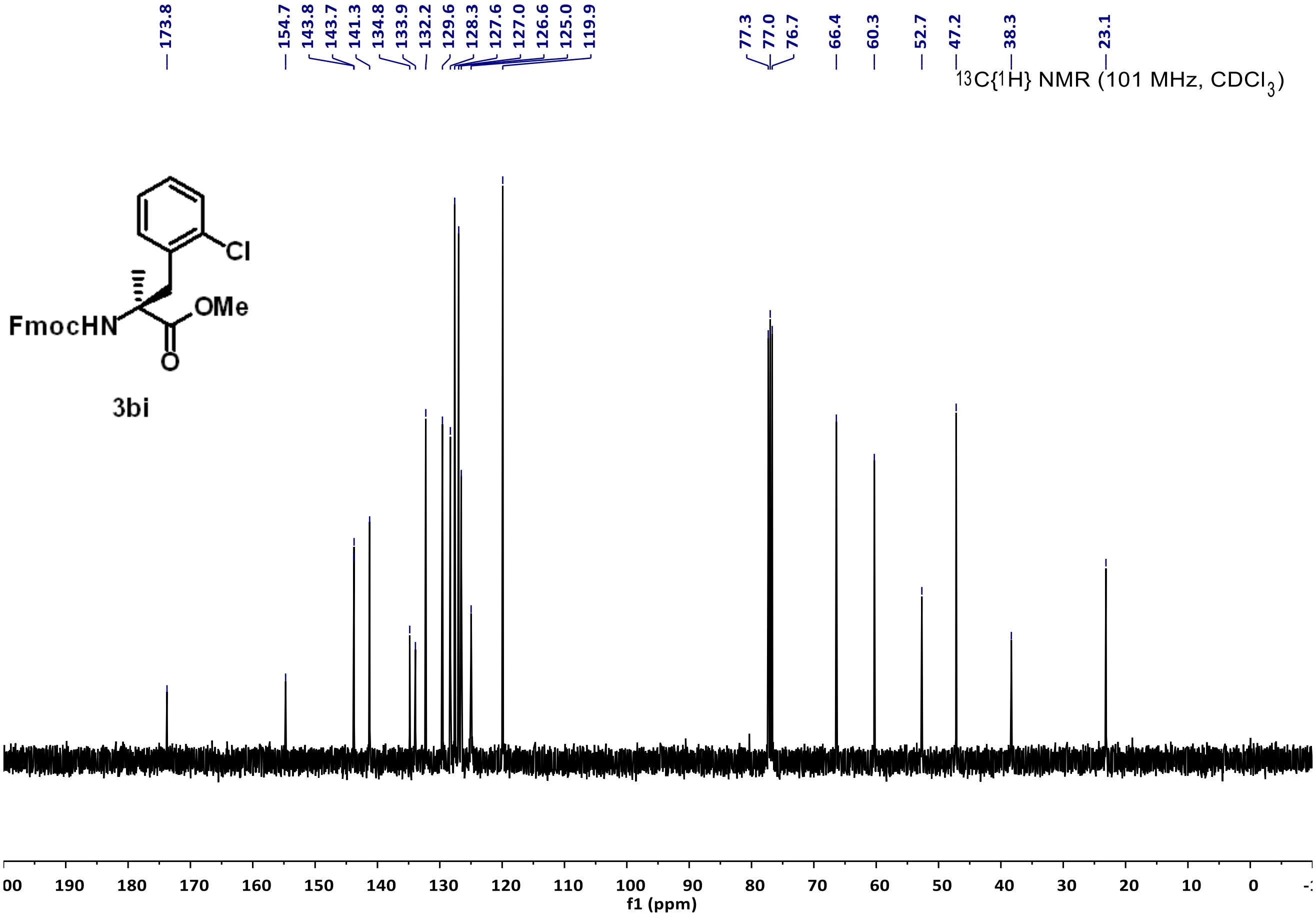

Supplementary Fig. 44. ^{13}C NMR spectrum of compound **3bi**

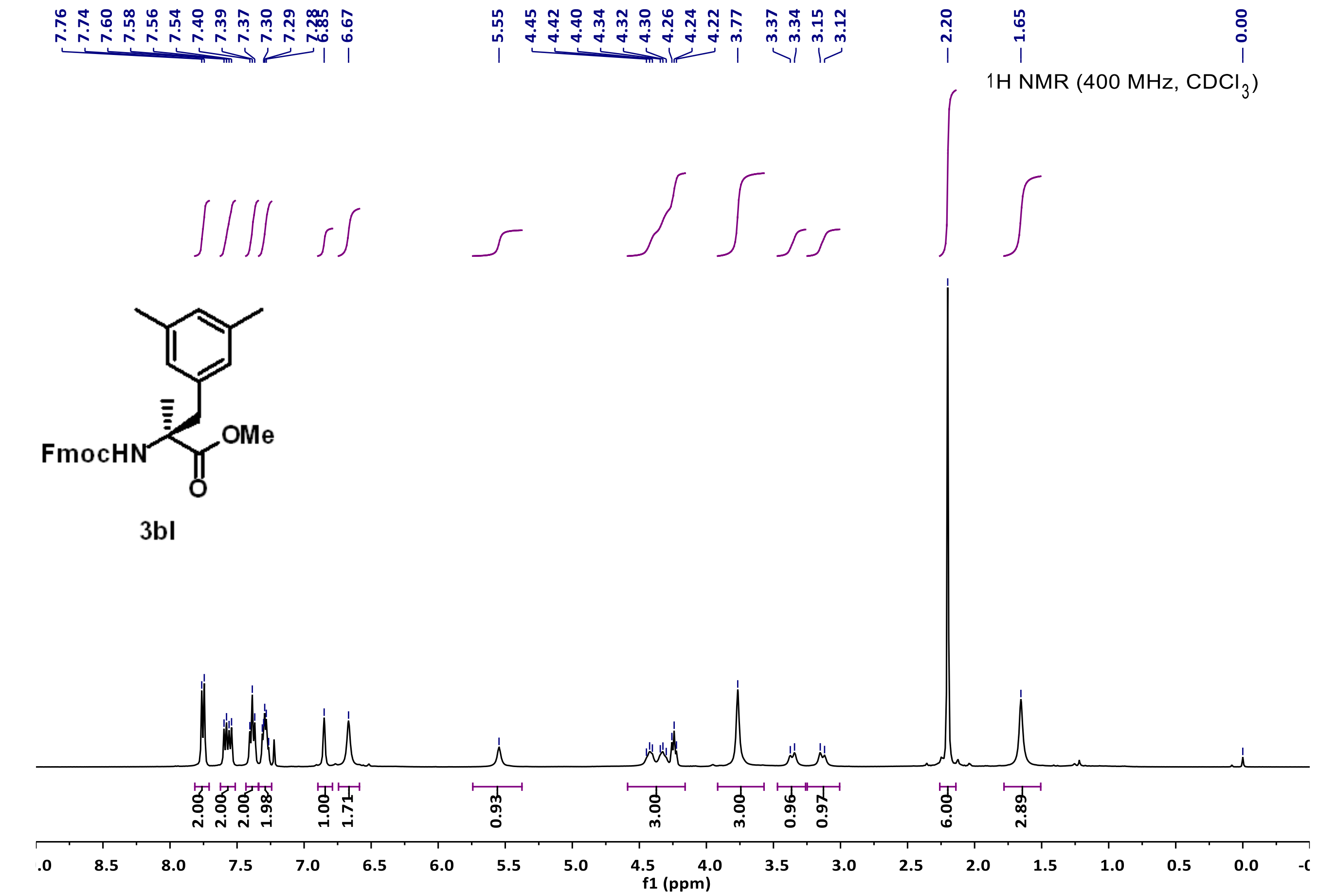

Supplementary Fig. 45. ^{1}H NMR spectrum of compound **3bl**

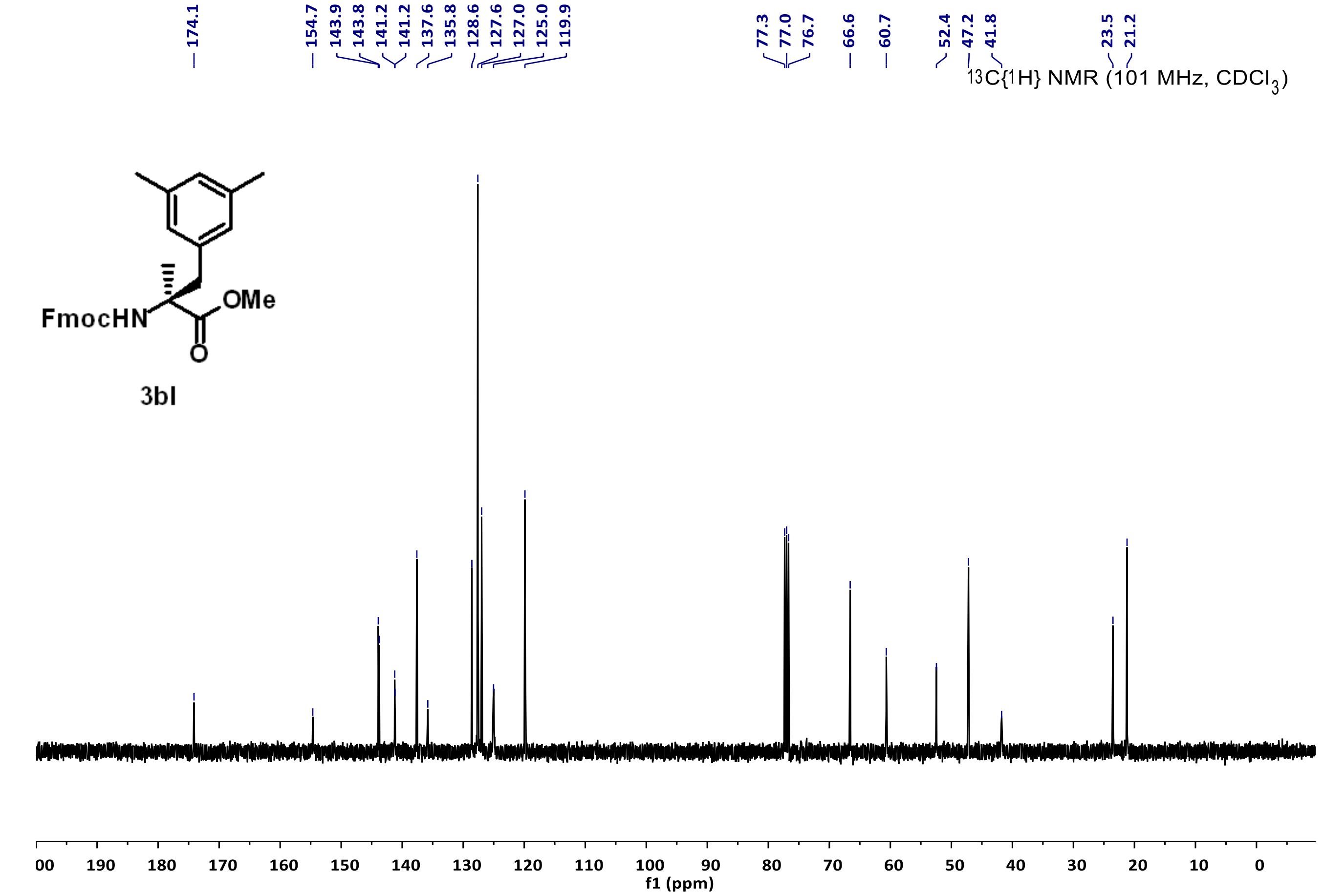

Supplementary Fig. 46. ^{13}C NMR spectrum of compound **3bl**

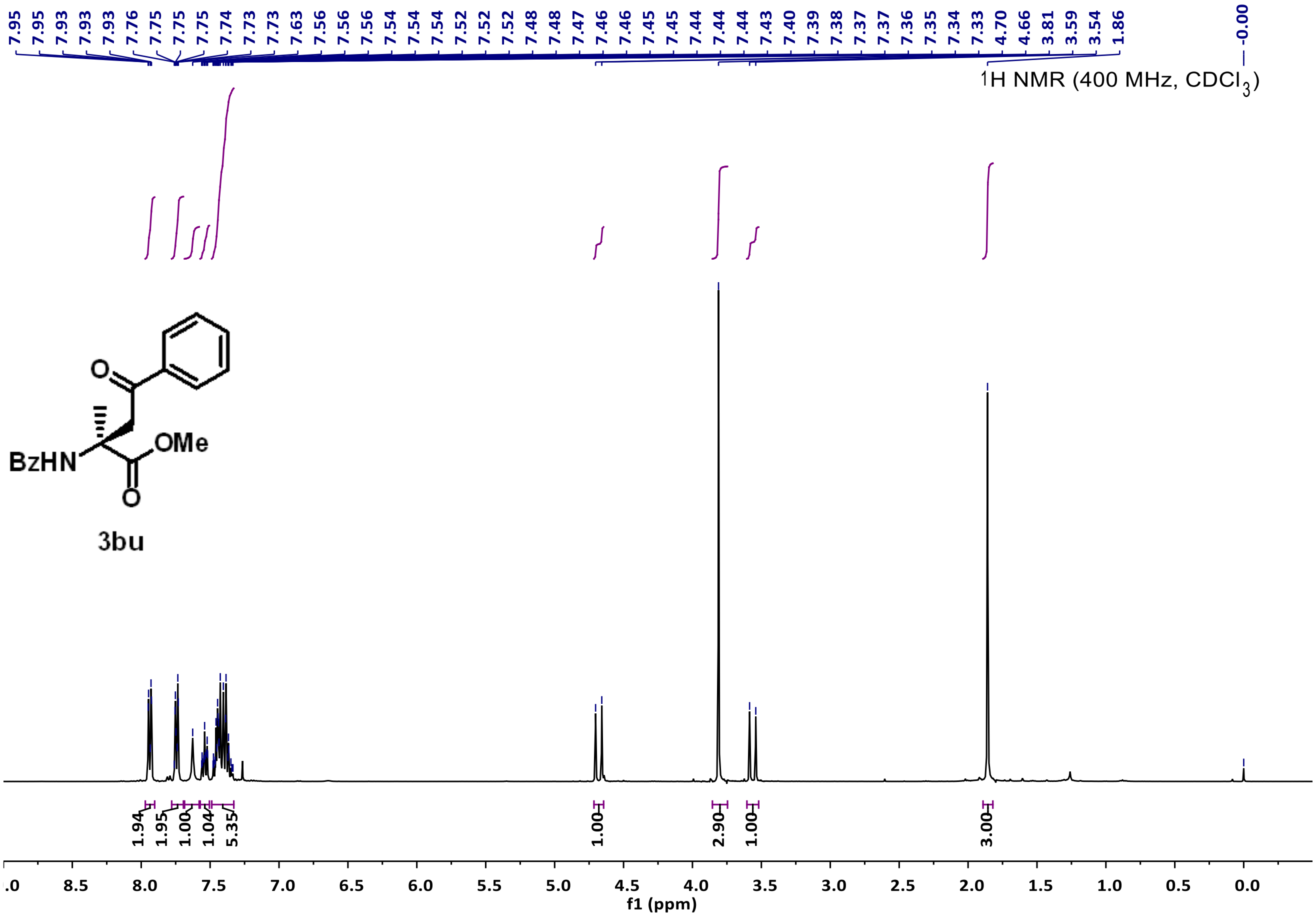

Supplementary Fig. 47. ^{1}H NMR spectrum of compound **3bu**

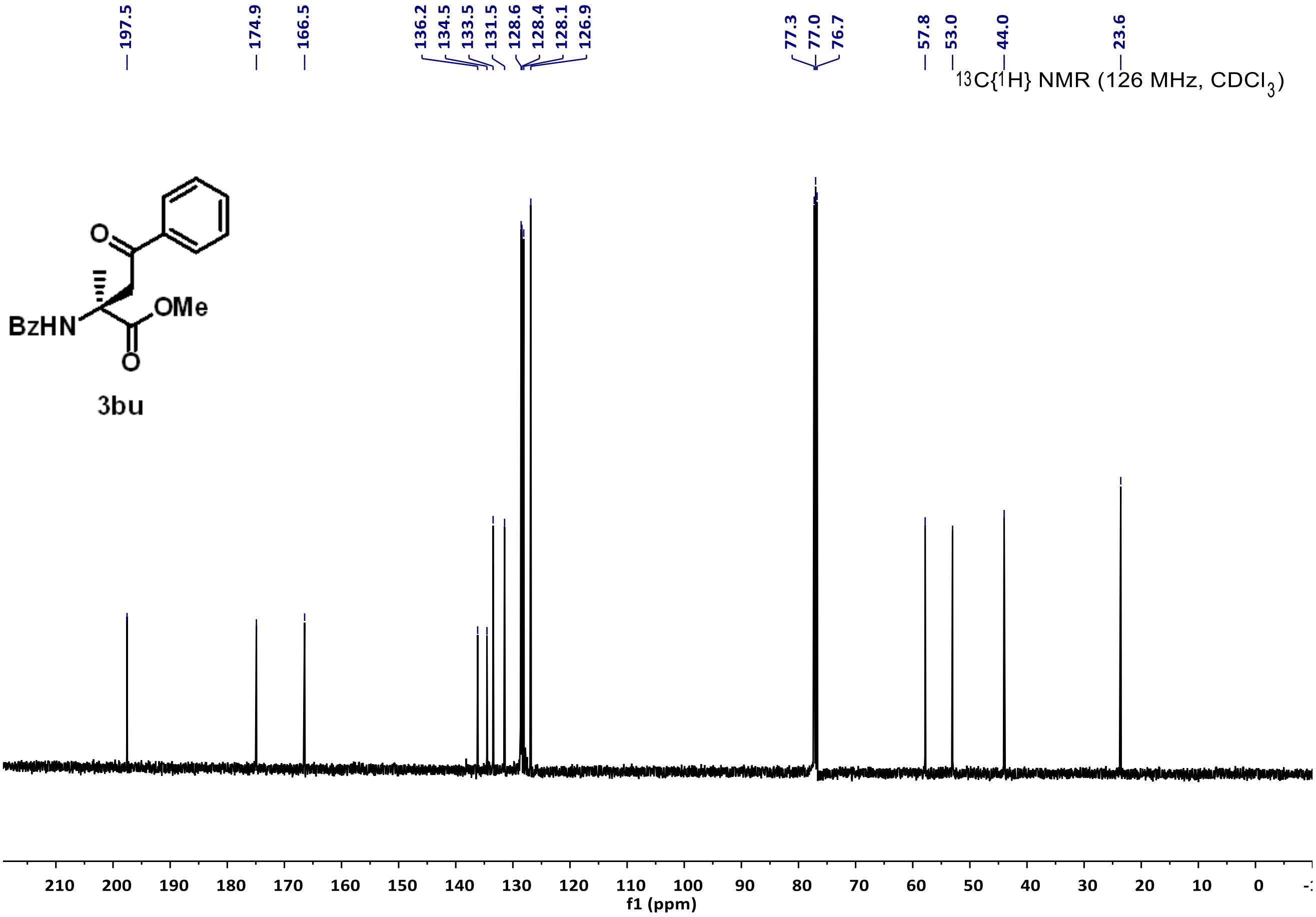

Supplementary Fig. 48. ^{13}C NMR spectrum of compound **3bu**

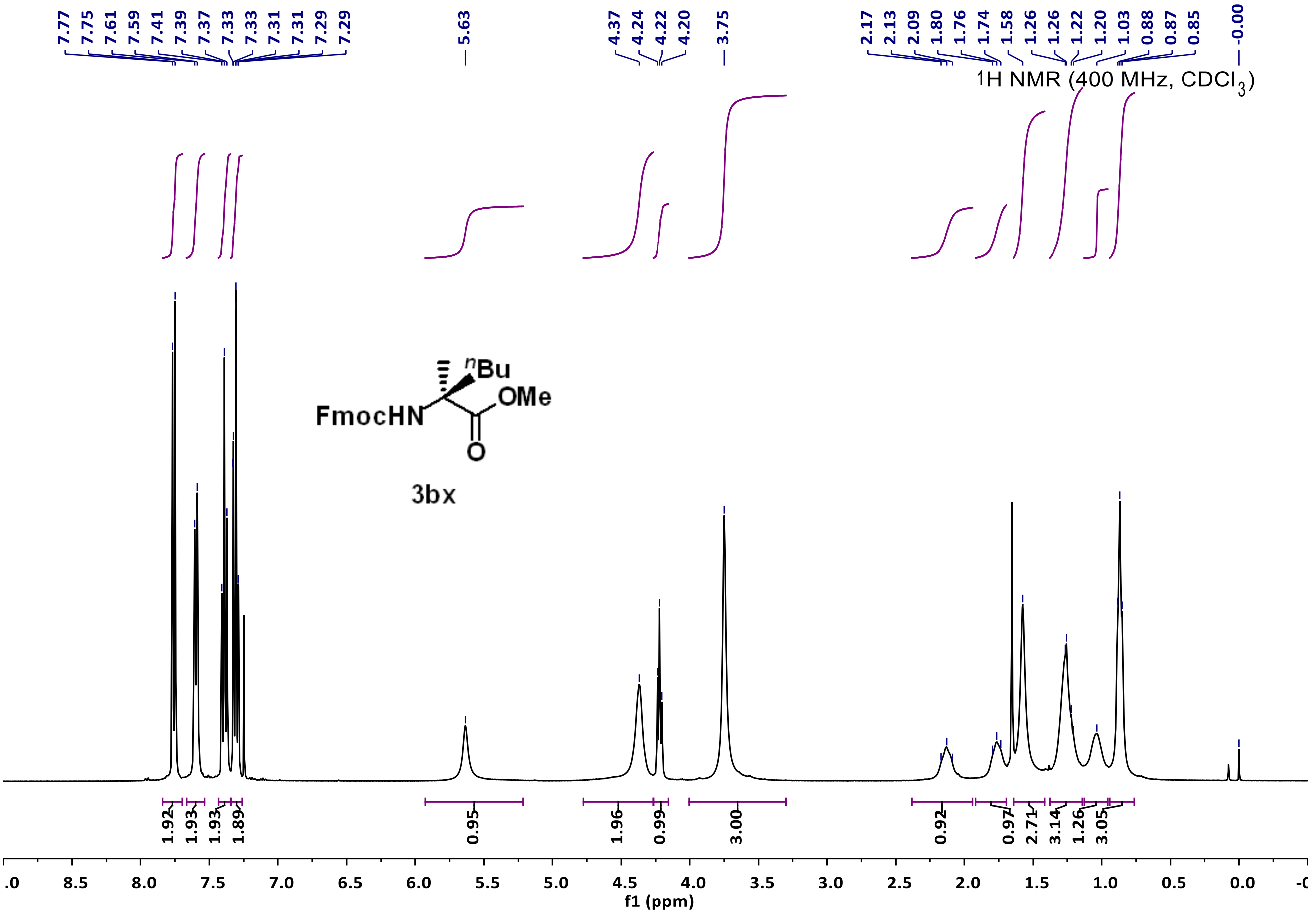

Supplementary Fig. 49. ^{1}H NMR spectrum of compound **3bx**

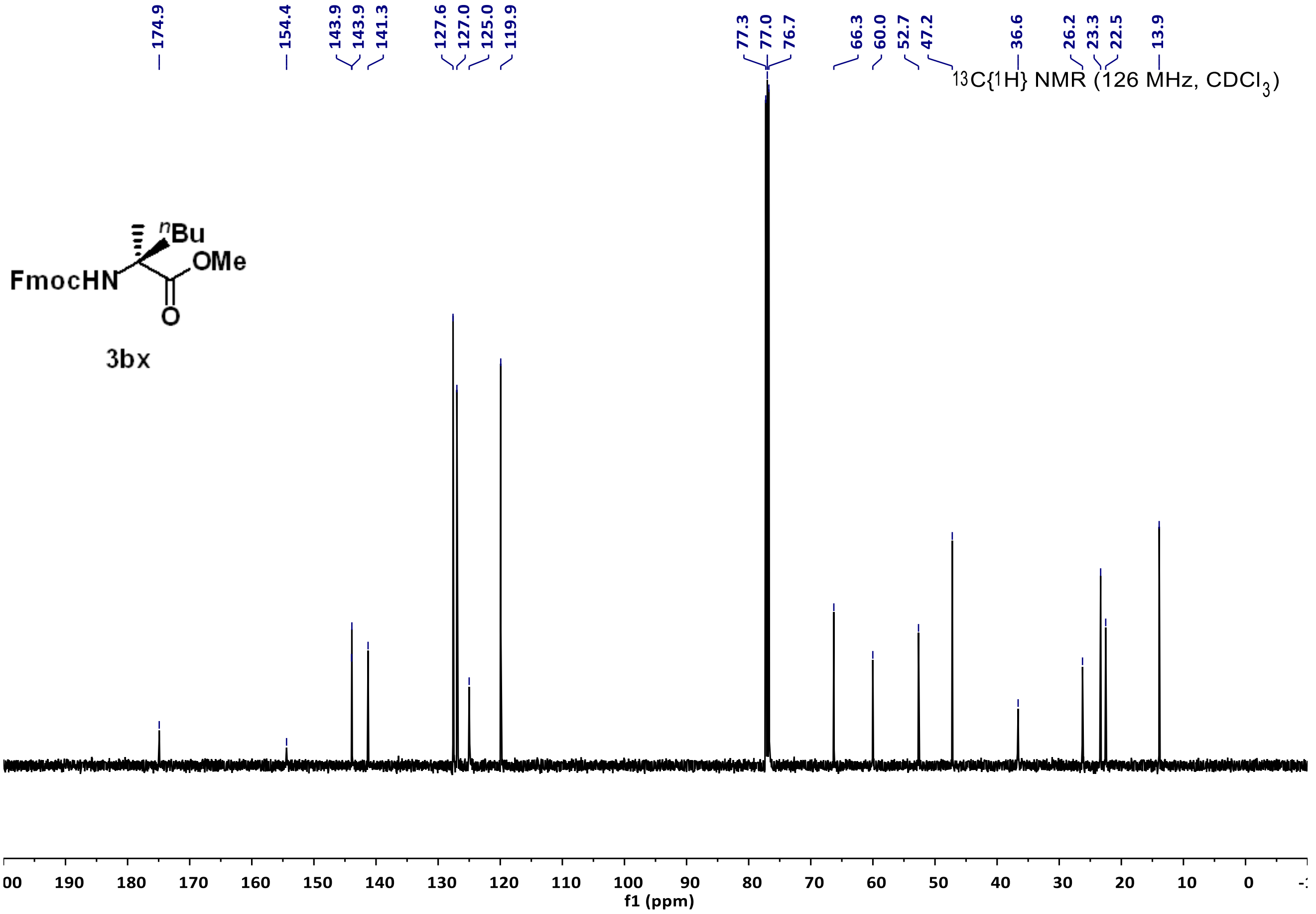

Supplementary Fig. 50. ^{13}C NMR spectrum of compound **3bx**

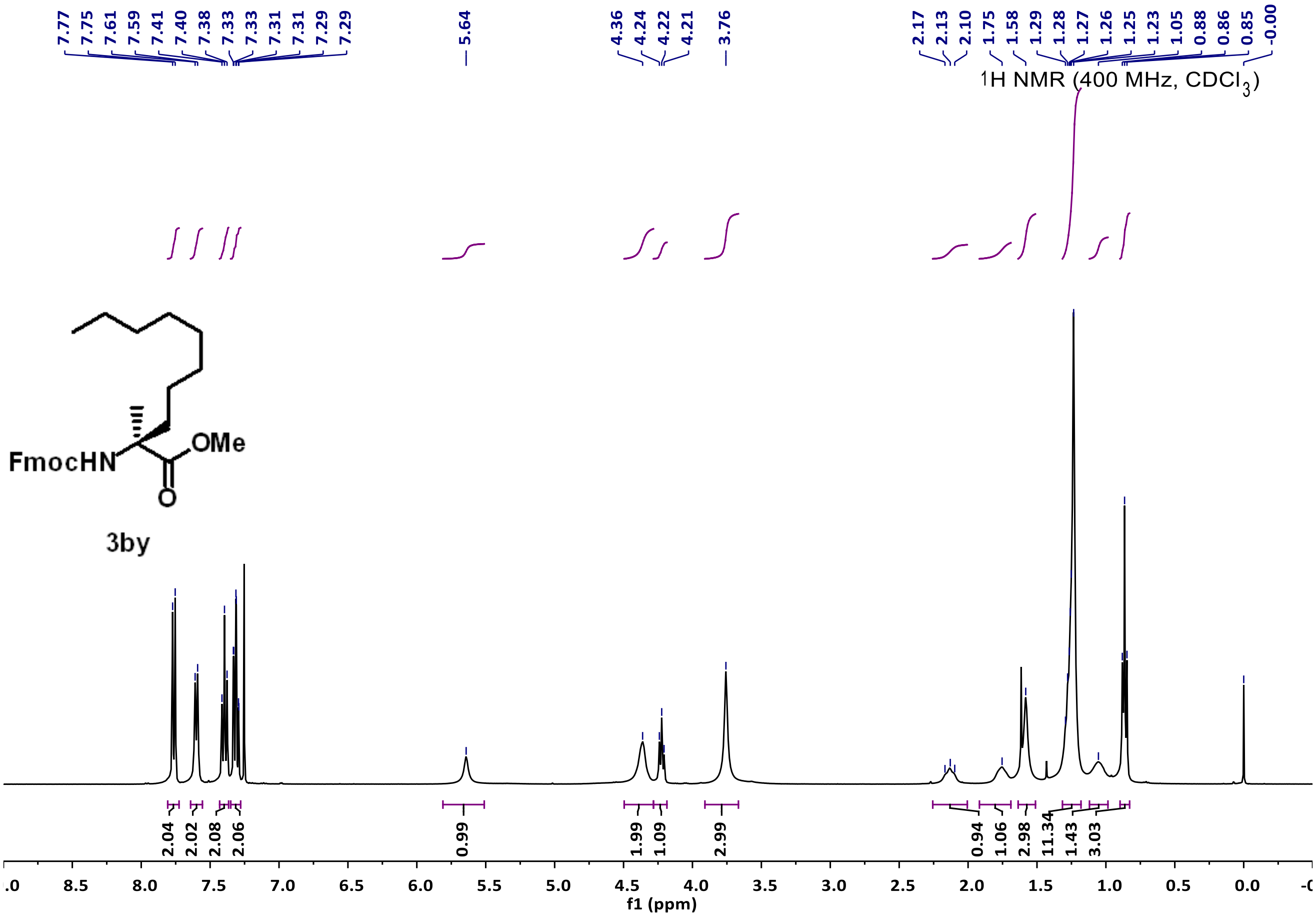

Supplementary Fig. 51. ^{1}H NMR spectrum of compound **3by**

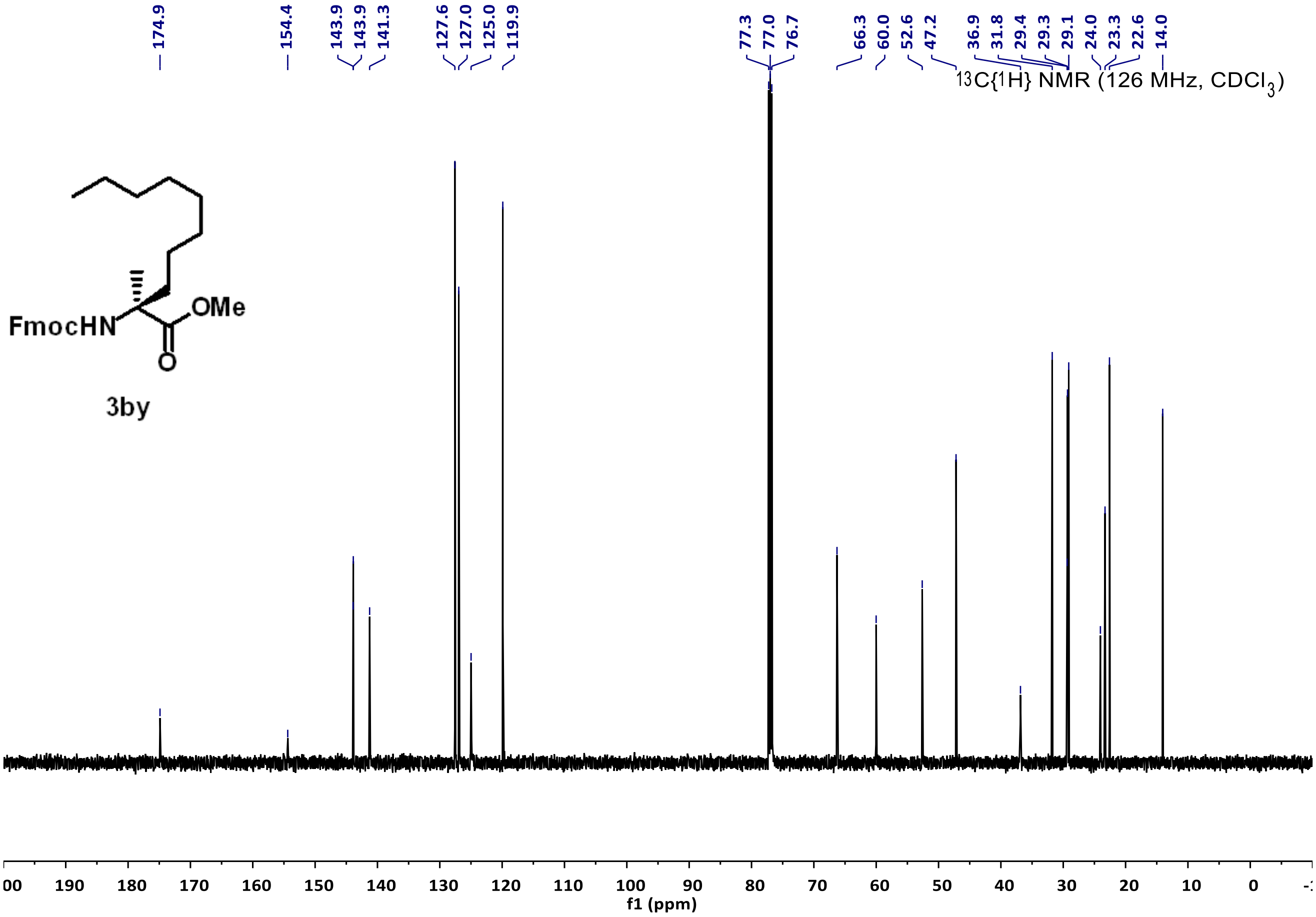

Supplementary Fig. 52. ^{13}C NMR spectrum of compound **3by**

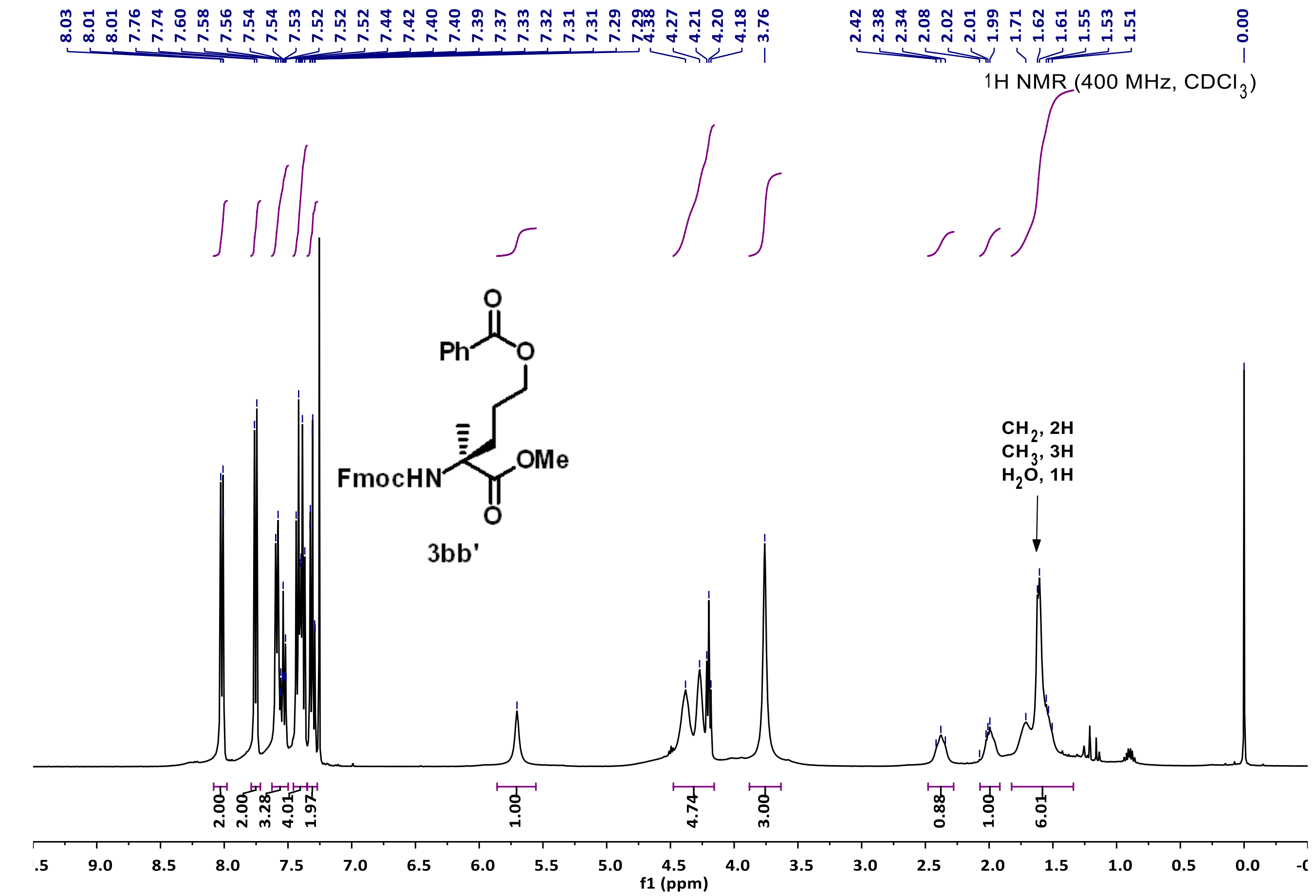

Supplementary Fig. 53. ^{1}H NMR spectrum of compound **3bb'**

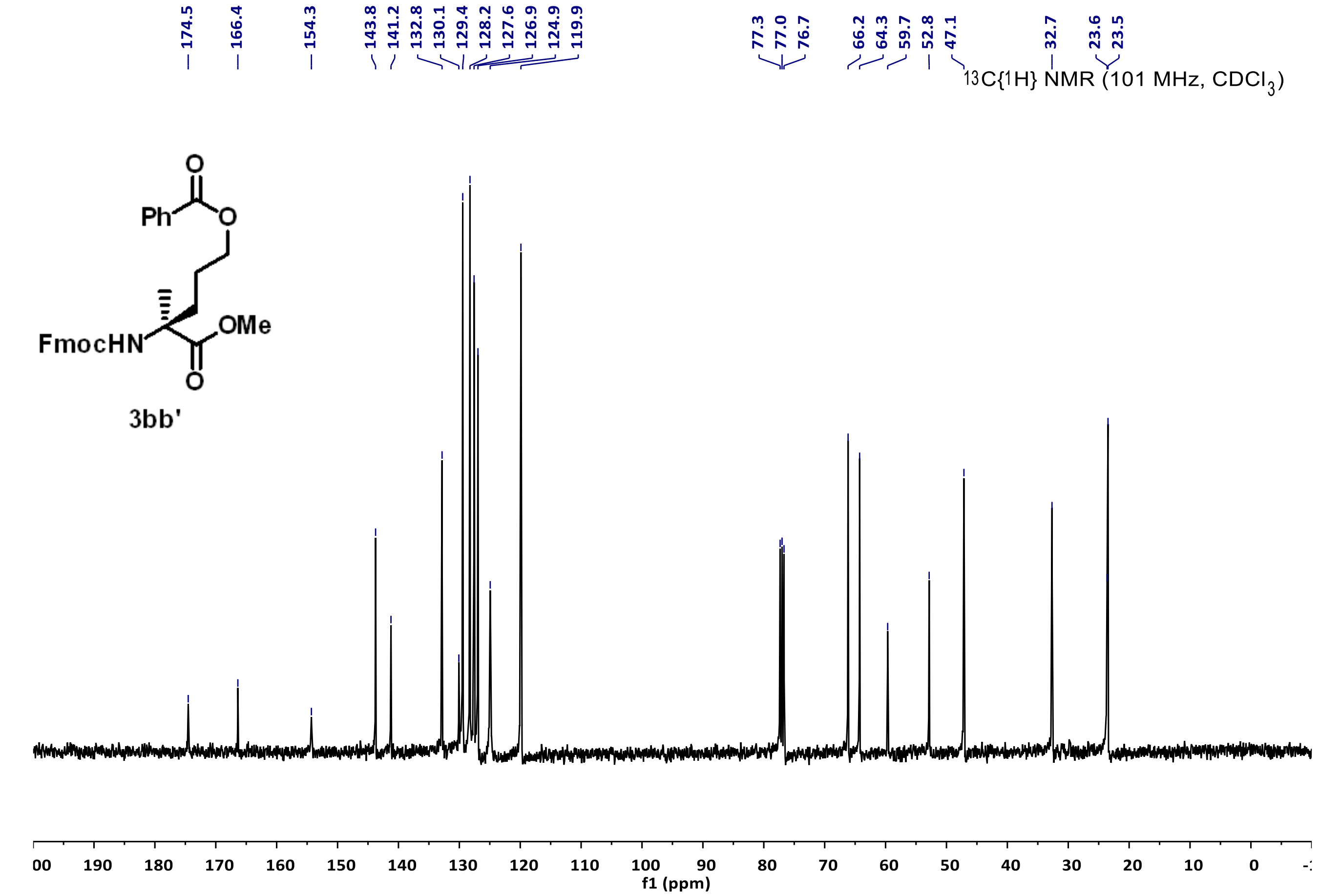

Supplementary Fig. 54. ^{13}C NMR spectrum of compound **3bb'**

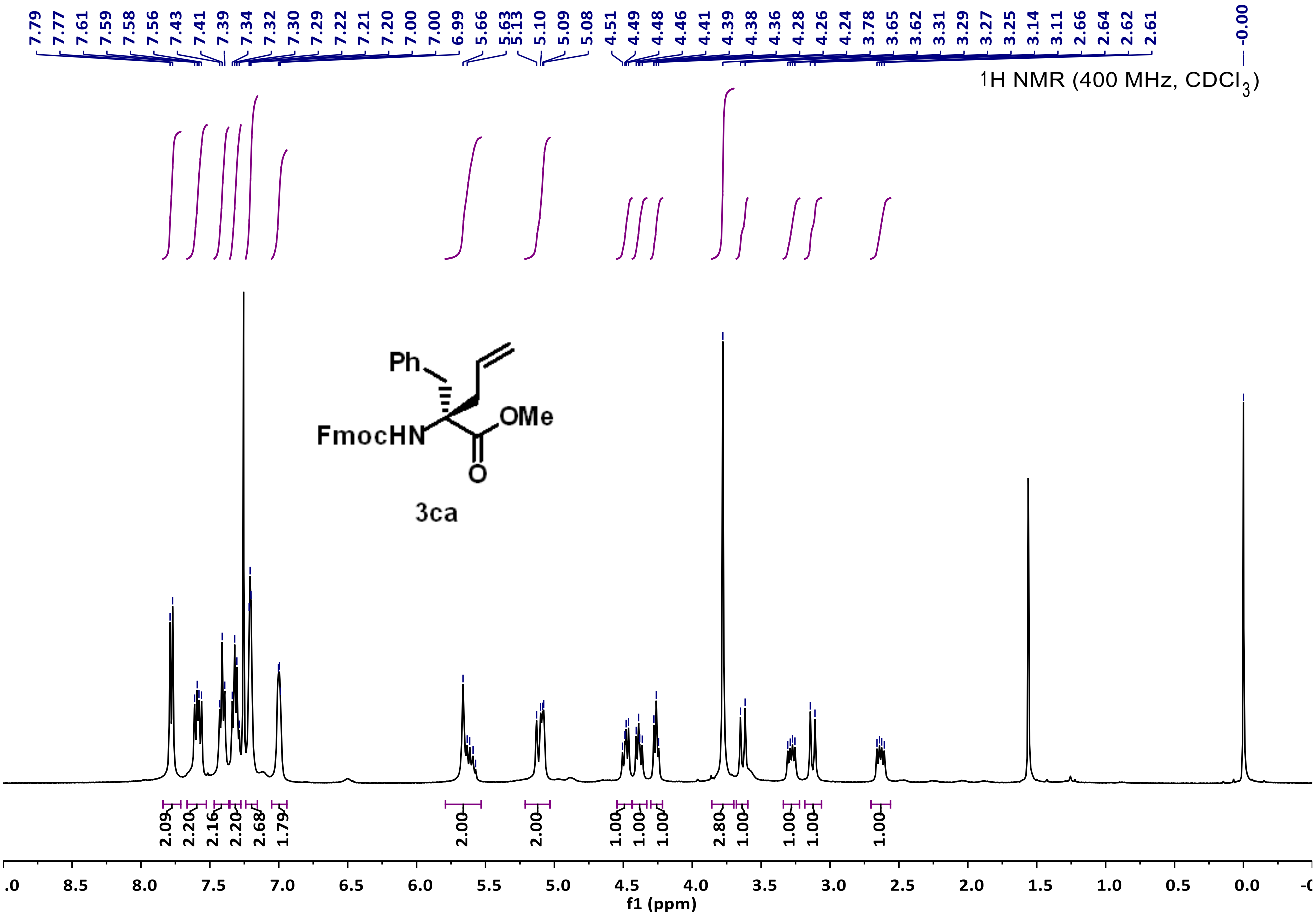

Supplementary Fig. 55. ^{1}H NMR spectrum of compound **3ca**

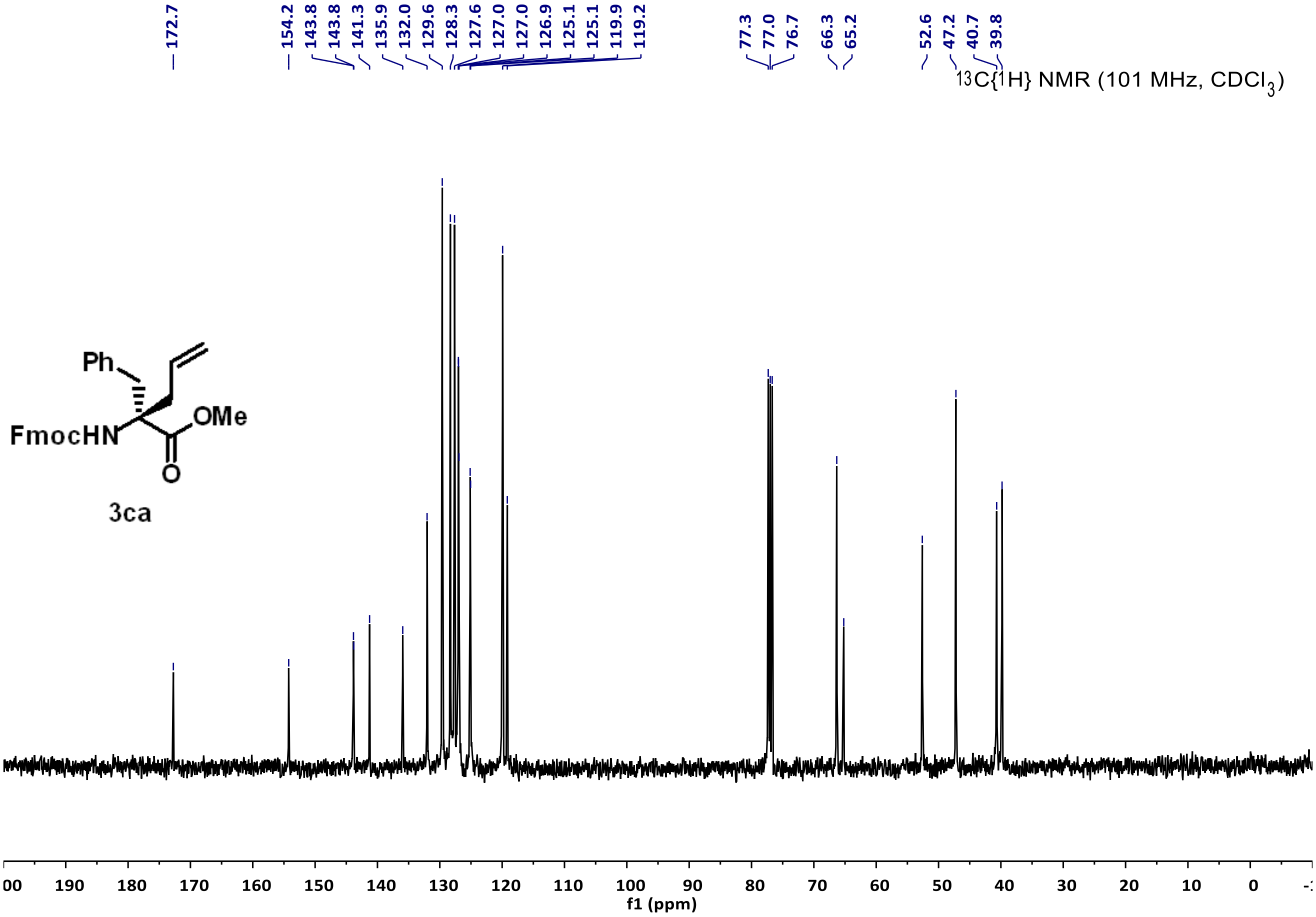

Supplementary Fig. 56. ^{13}C NMR spectrum of compound **3ca**

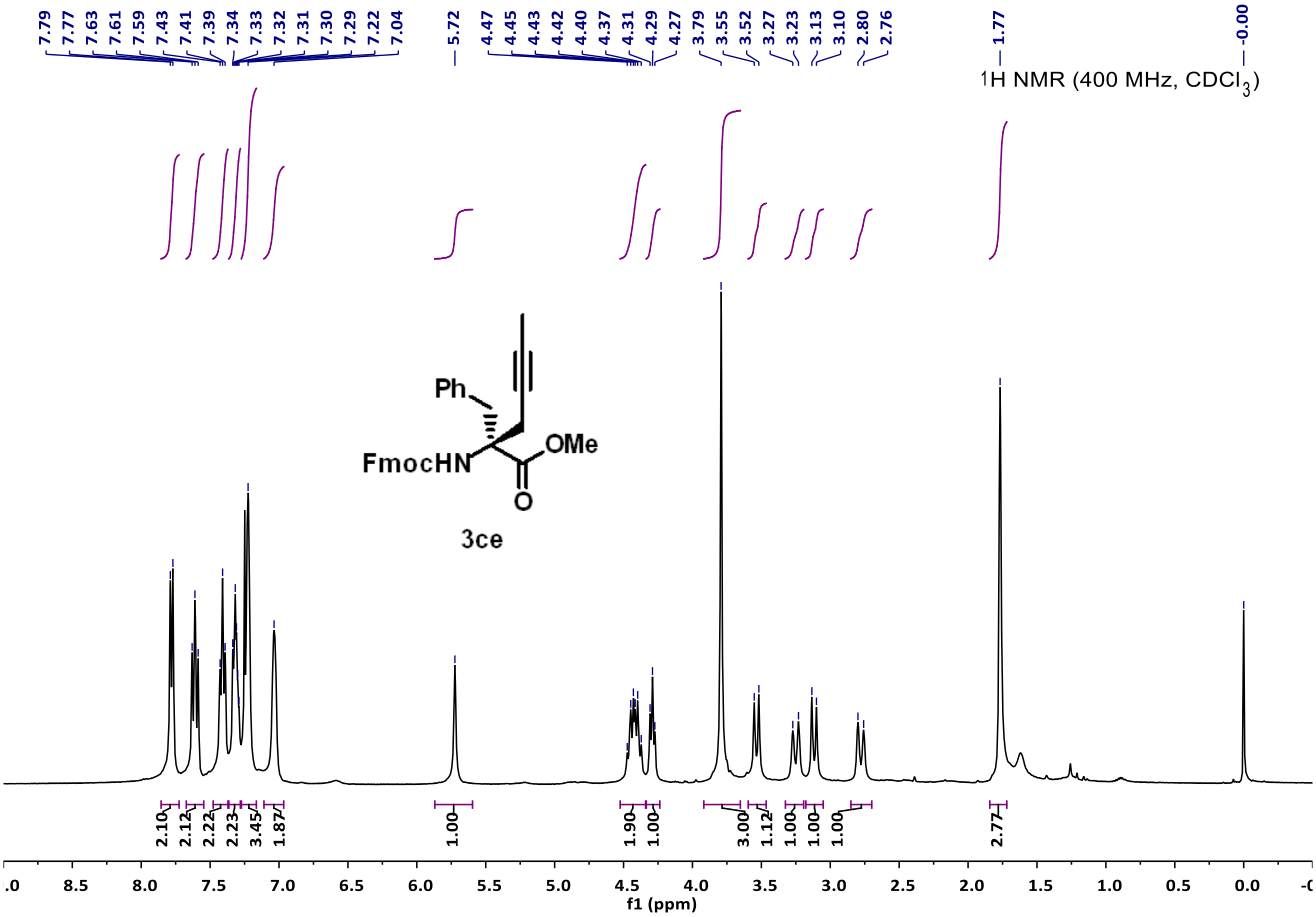

Supplementary Fig. 57. ^{1}H NMR spectrum of compound **3ce**

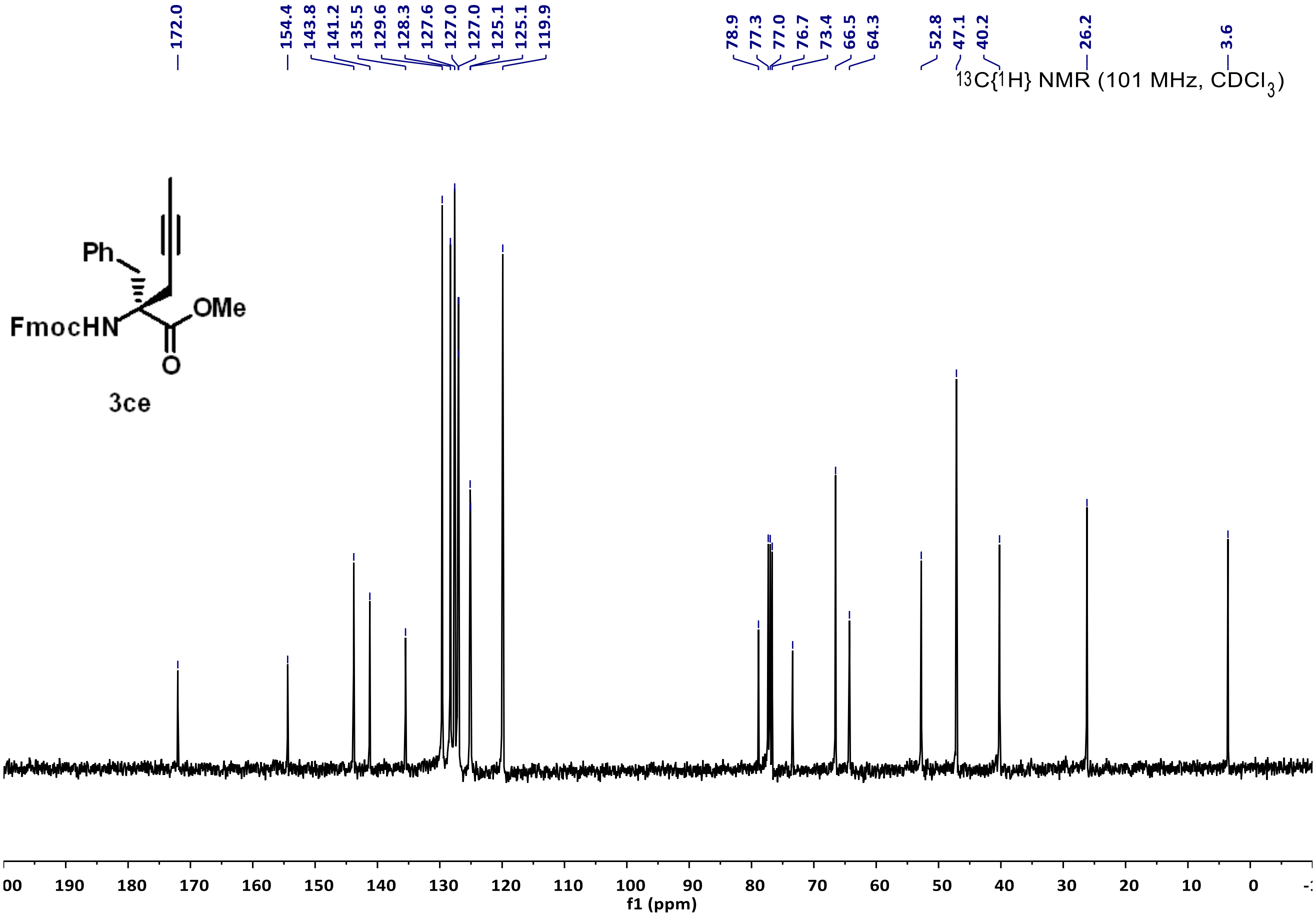

Supplementary Fig. 58. ^{13}C NMR spectrum of compound **3ce**

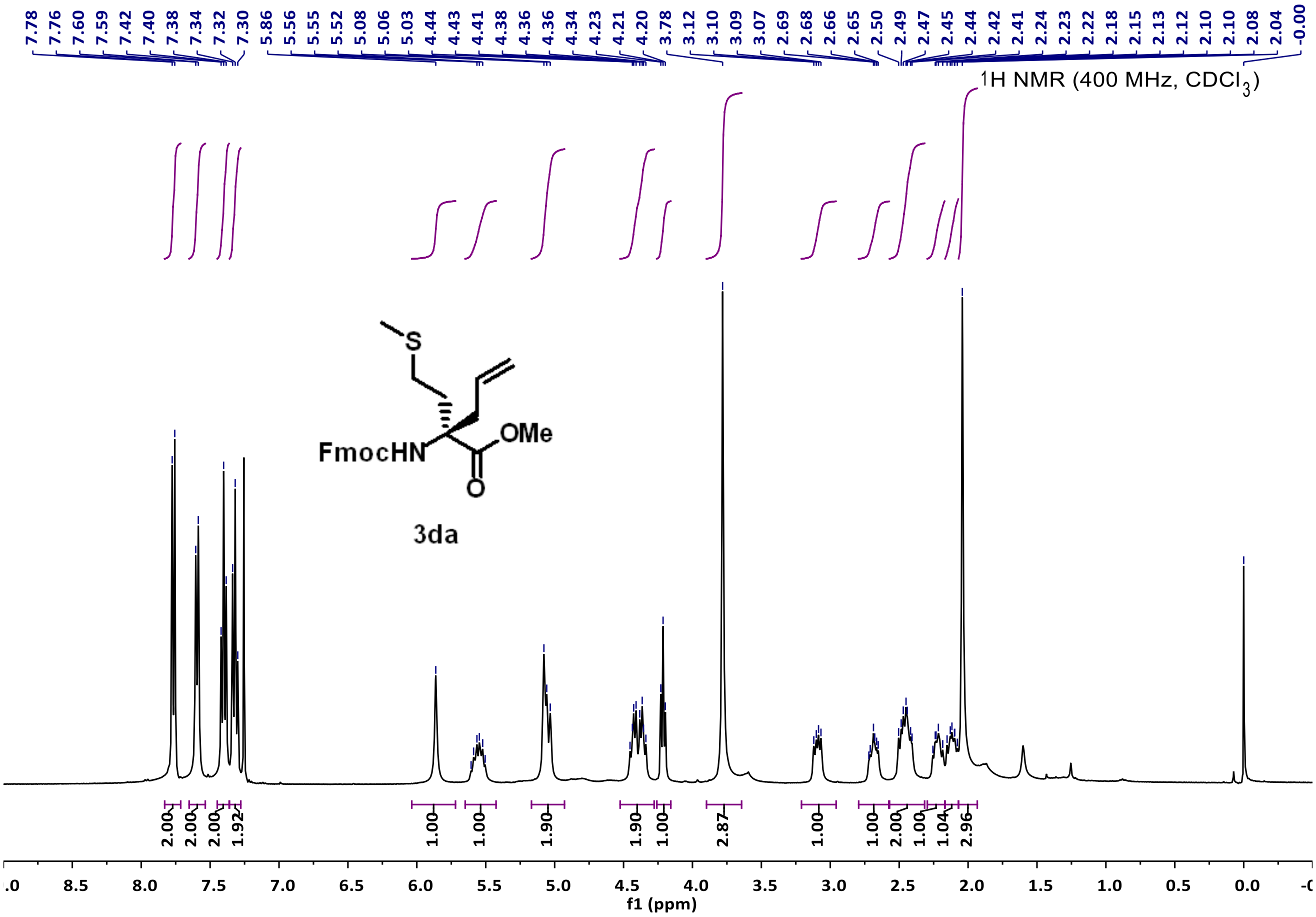

Supplementary Fig. 59. ^{1}H NMR spectrum of compound **3da**

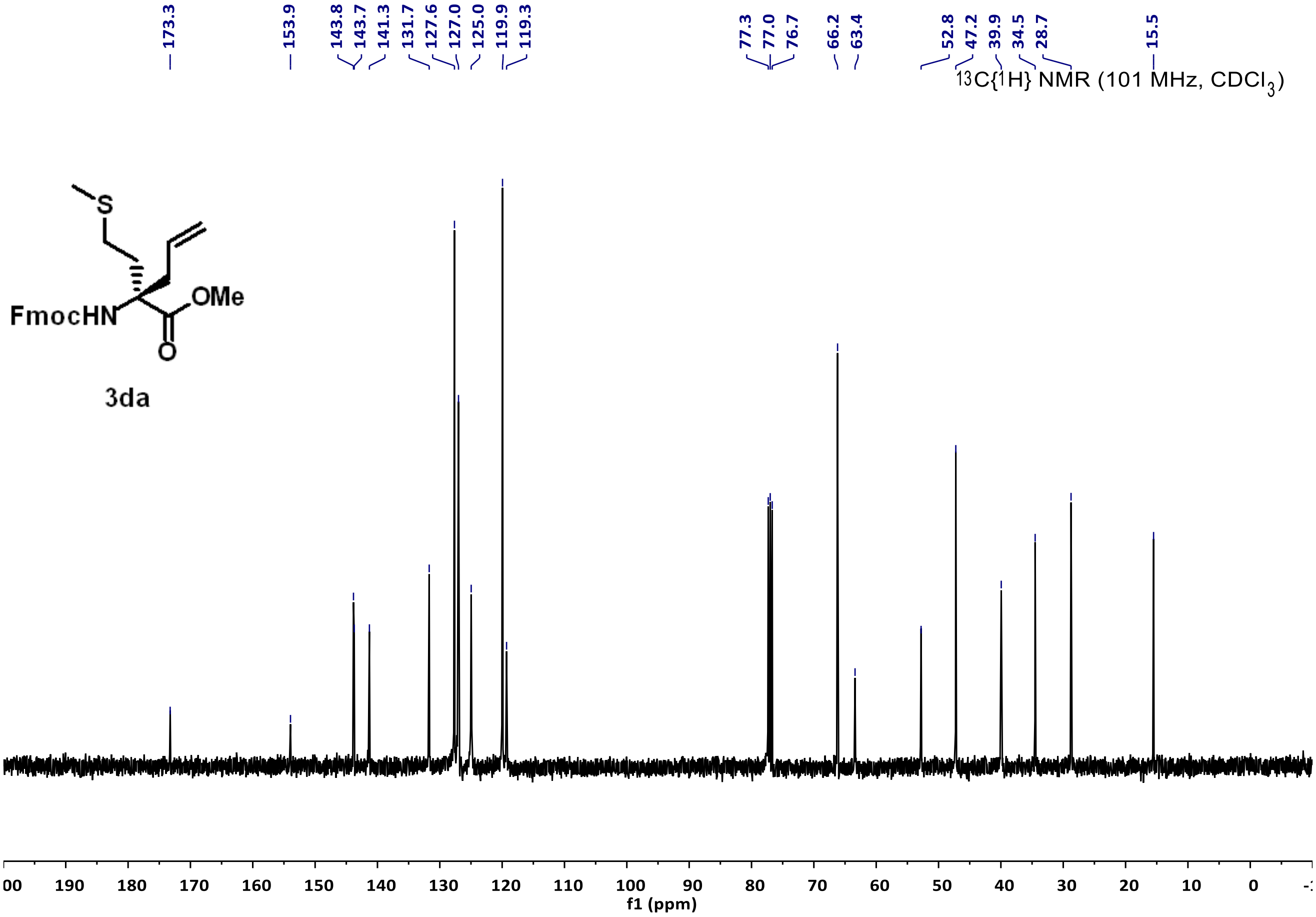

Supplementary Fig. 60. ^{13}C NMR spectrum of compound **3da**

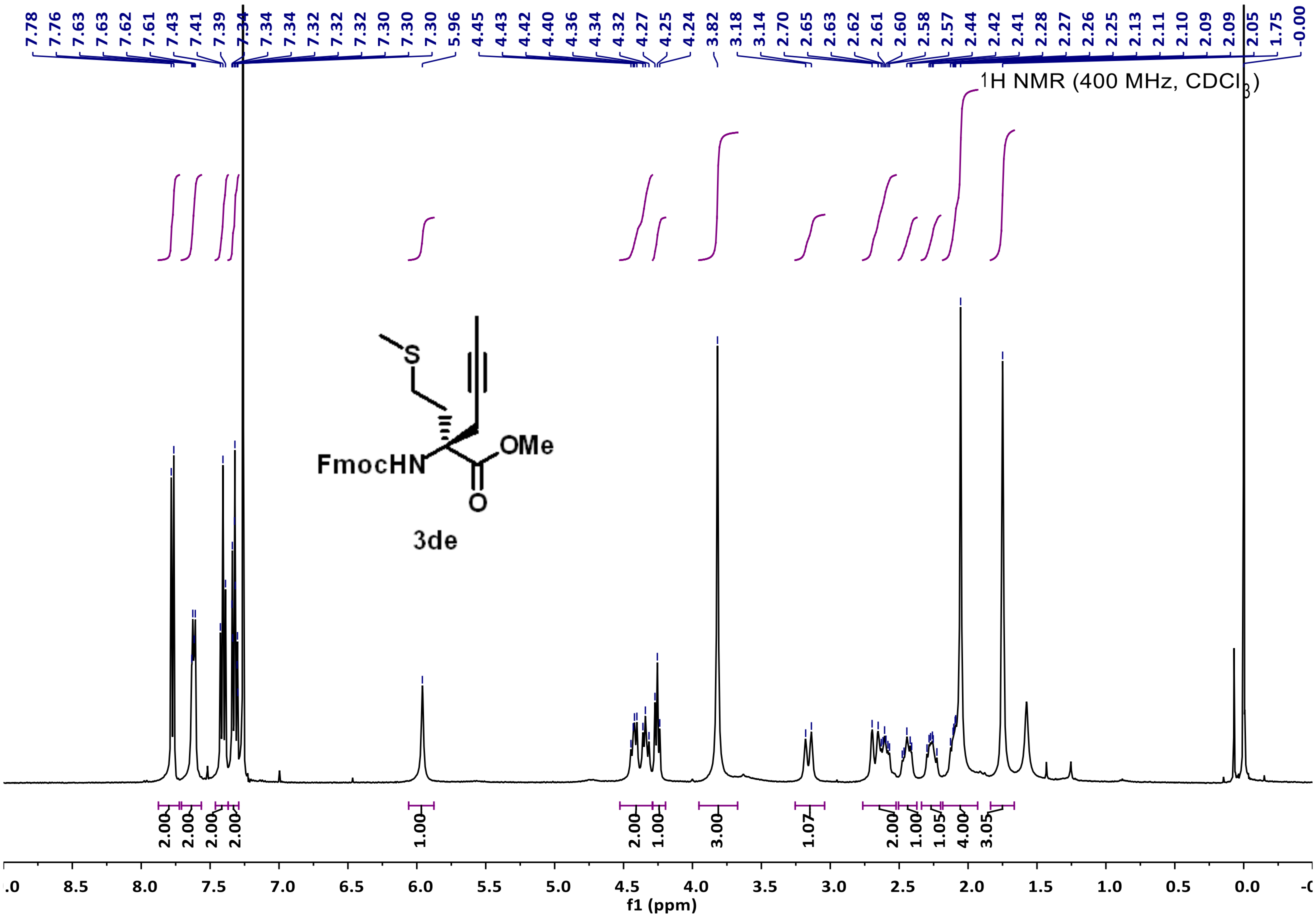

Supplementary Fig. 61. ^{1}H NMR spectrum of compound **3de**

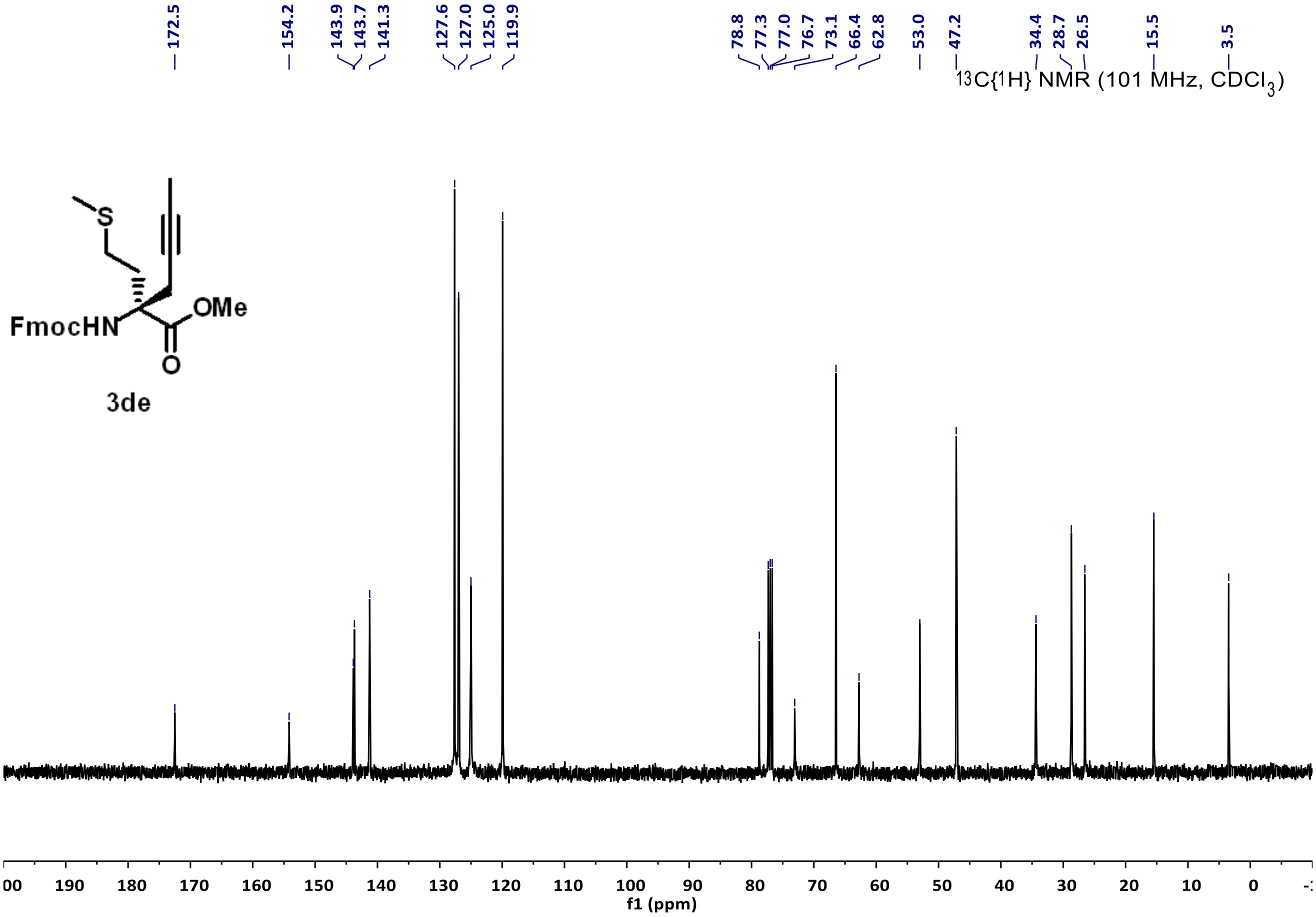

Supplementary Fig. 62. ^{13}C NMR spectrum of compound **3de**

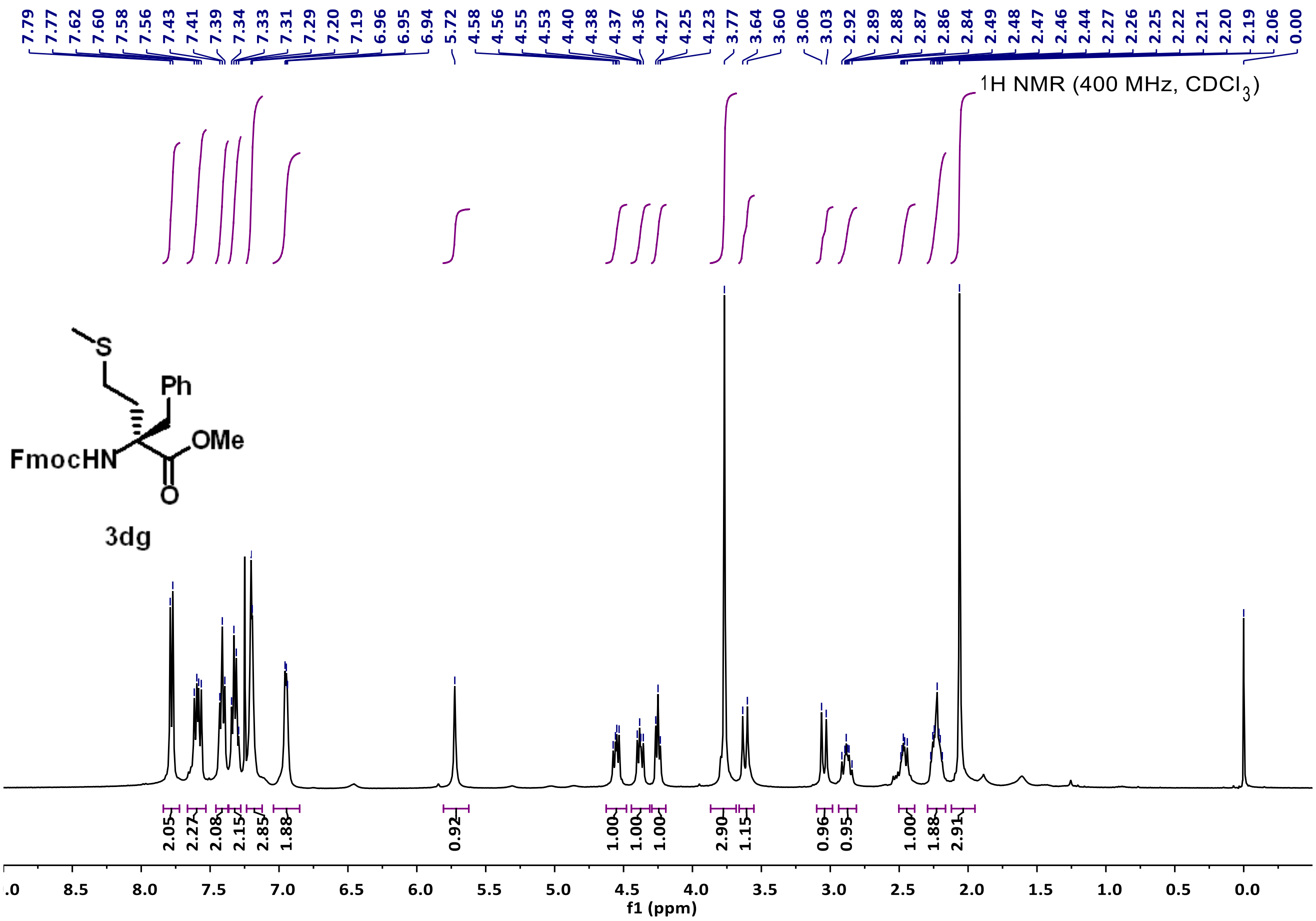

Supplementary Fig. 63. ^{1}H NMR spectrum of compound **3dg**

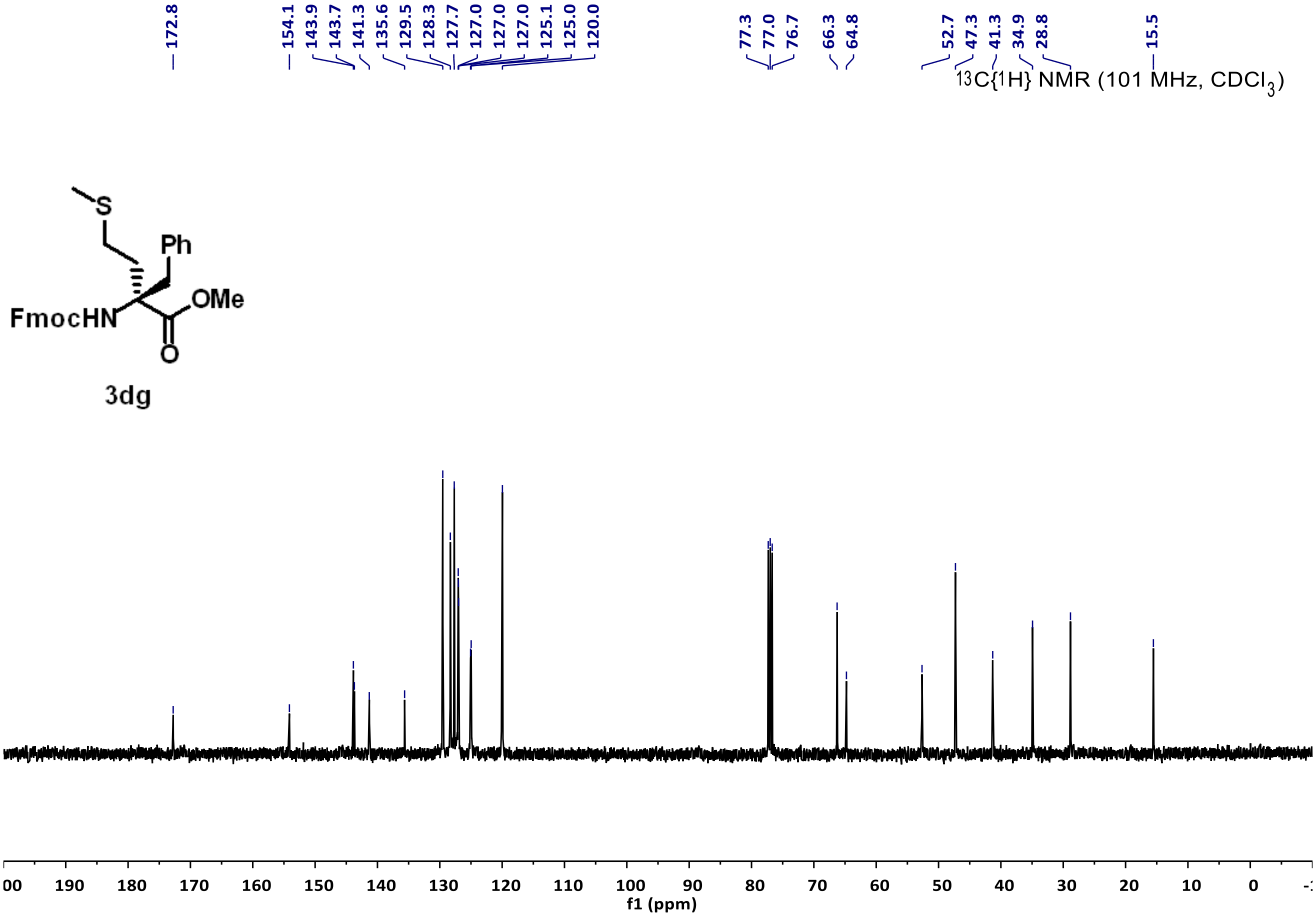

Supplementary Fig. 64. ^{13}C NMR spectrum of compound **3dg**

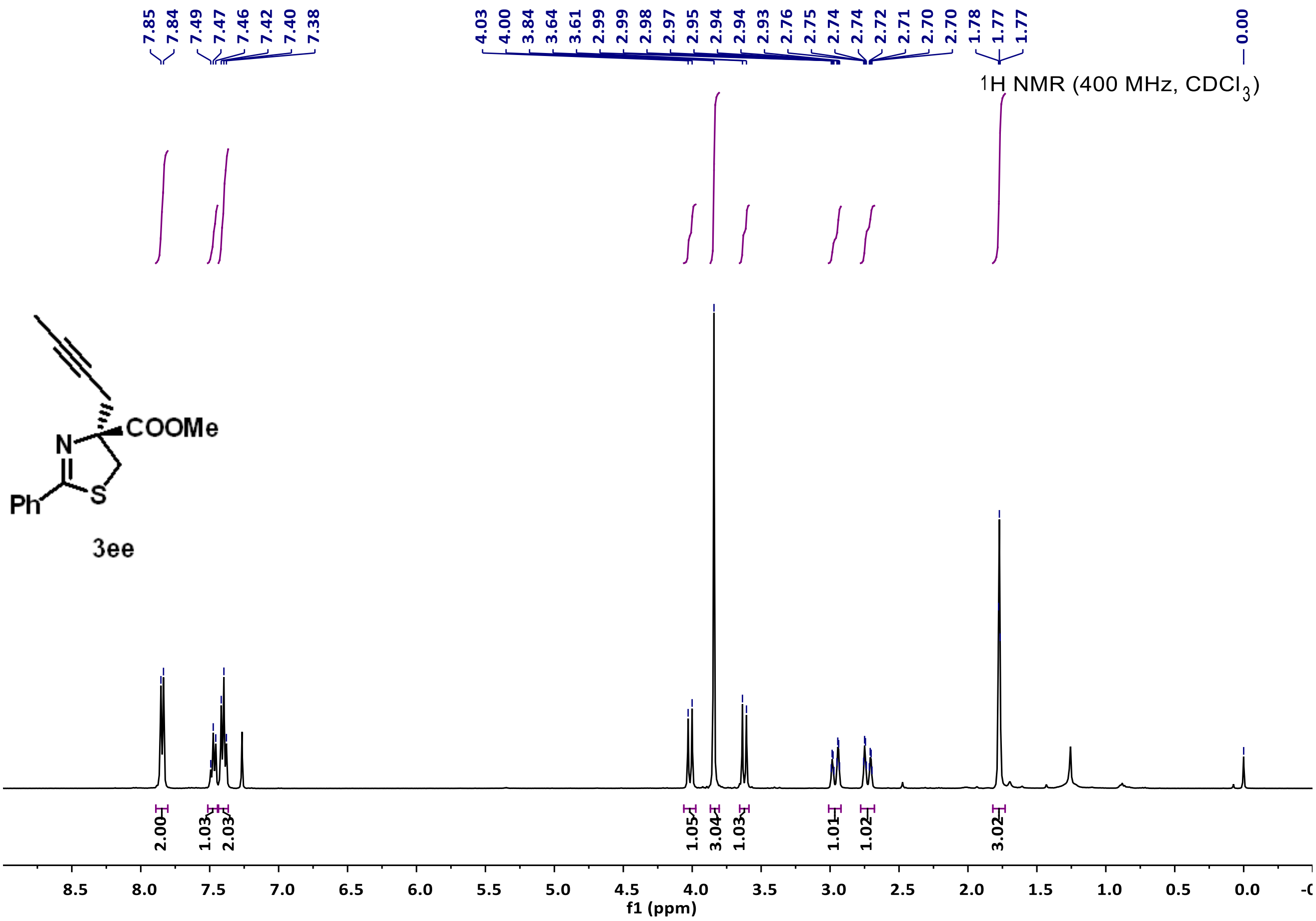

Supplementary Fig. 65. ^{1}H NMR spectrum of compound **3ee**

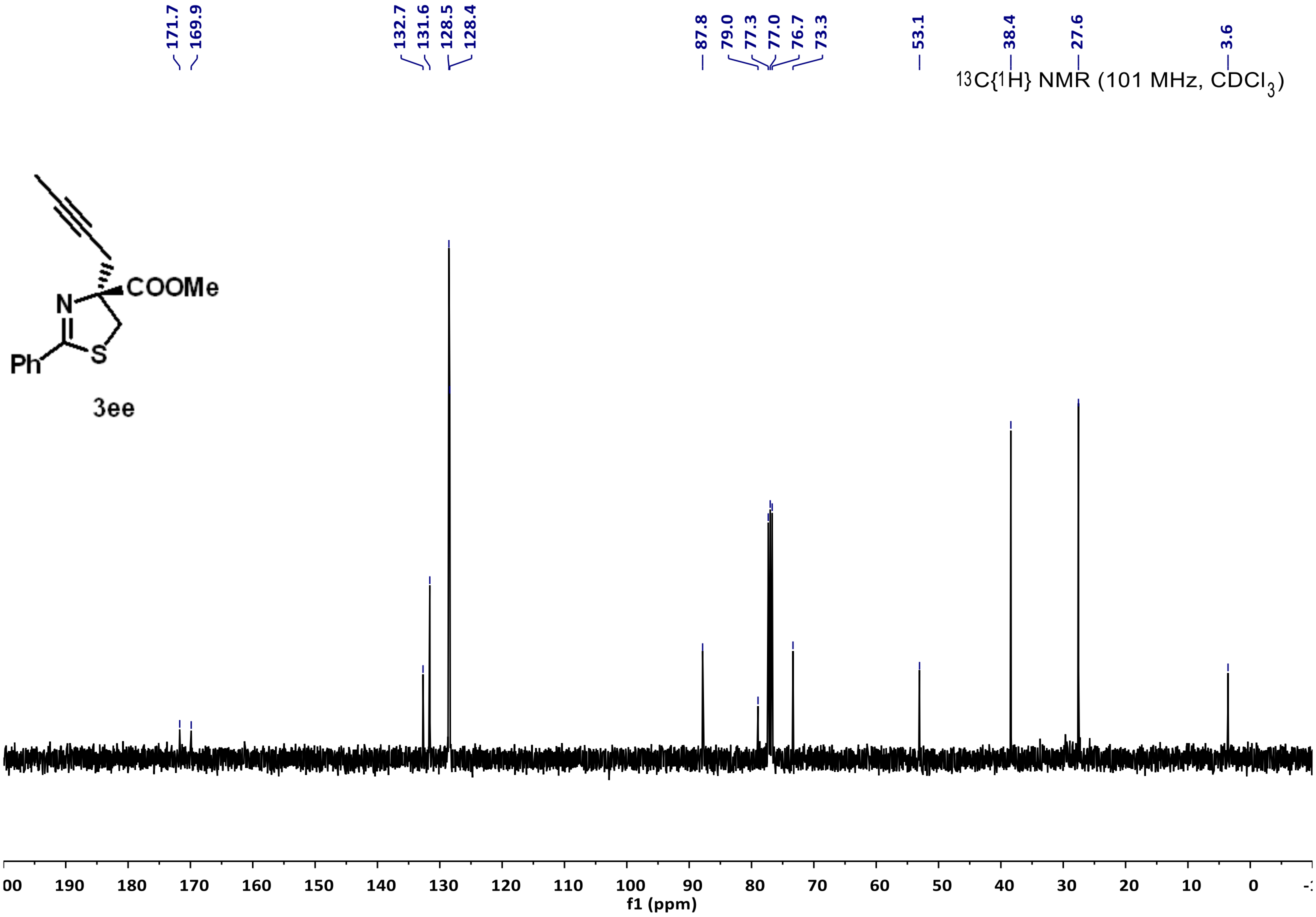

Supplementary Fig. 66. ^{13}C NMR spectrum of compound **3ee**

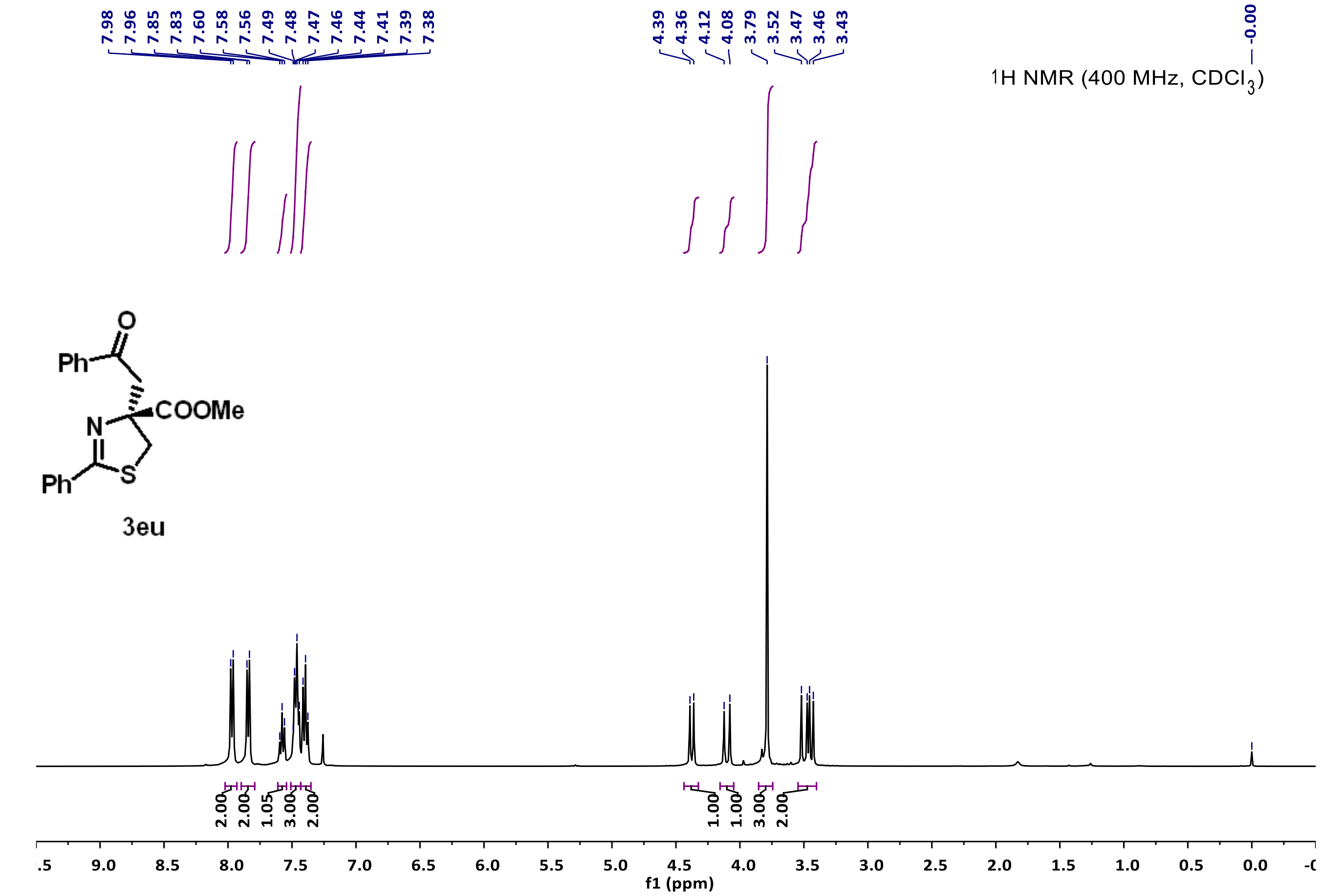

Supplementary Fig. 67. ^{1}H NMR spectrum of compound **3eu**

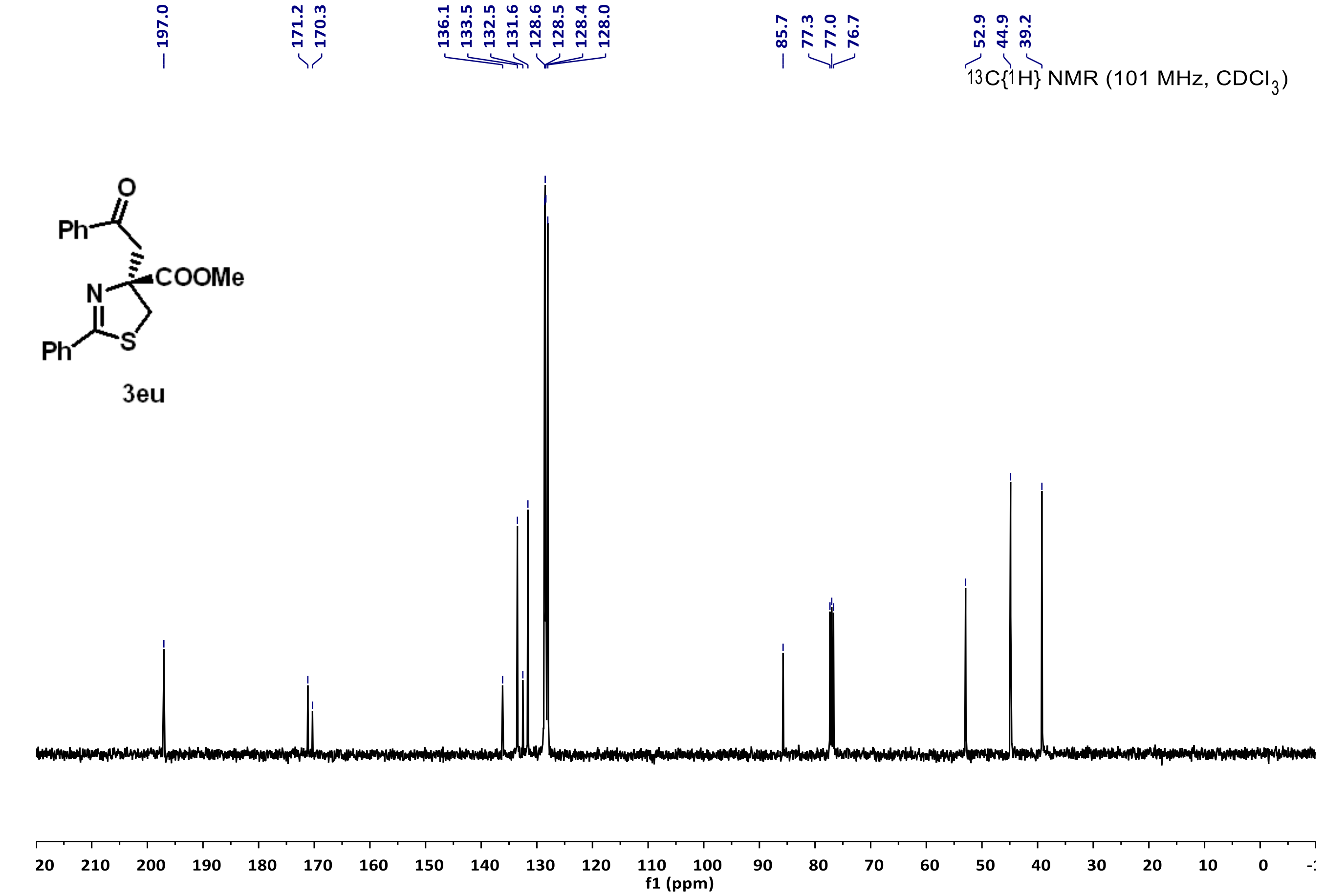

Supplementary Fig. 68. ^{13}C NMR spectrum of compound **3eu**

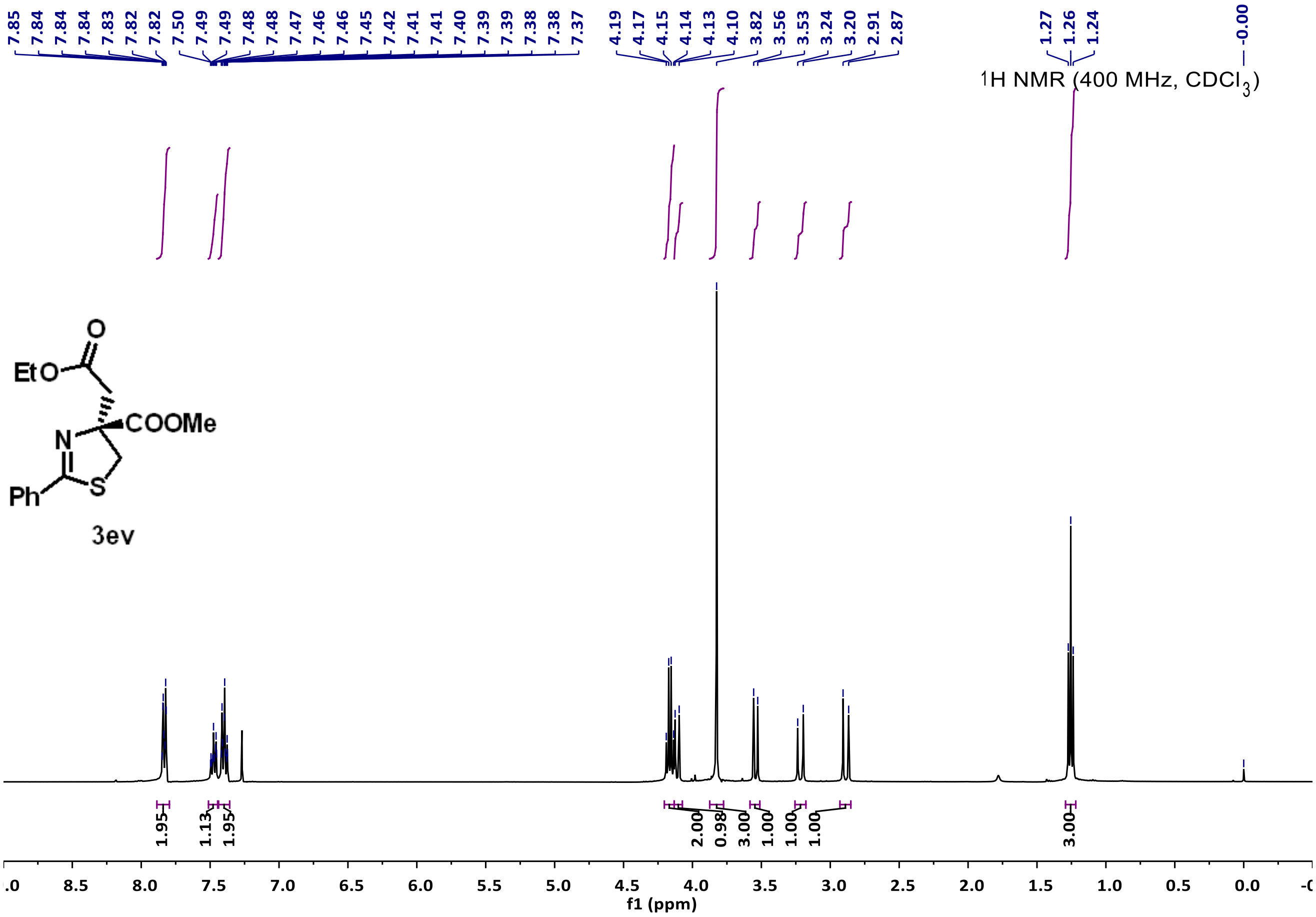

Supplementary Fig. 69. ^{1}H NMR spectrum of compound **3ev**

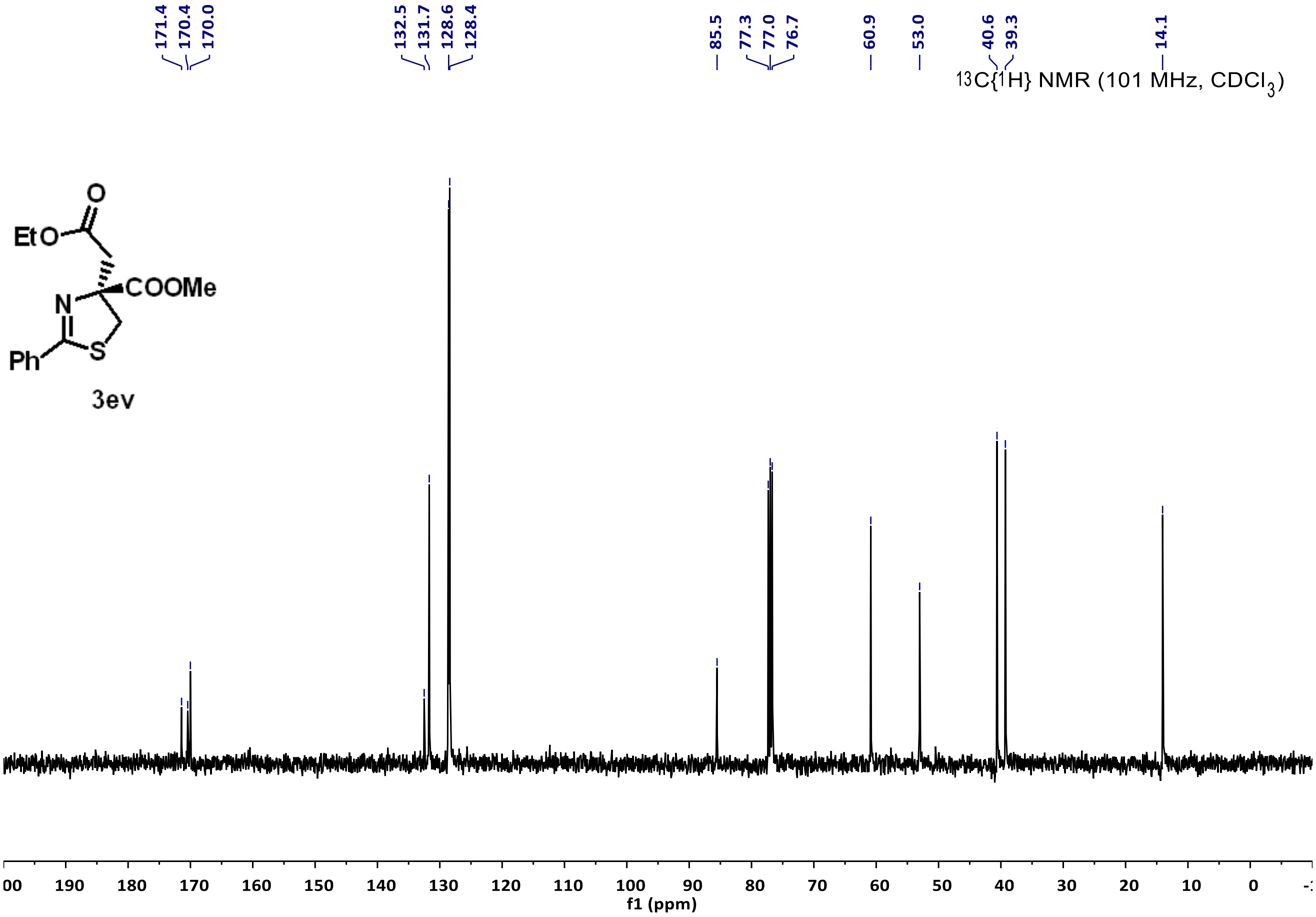

Supplementary Fig. 70. ^{13}C NMR spectrum of compound **3ev**

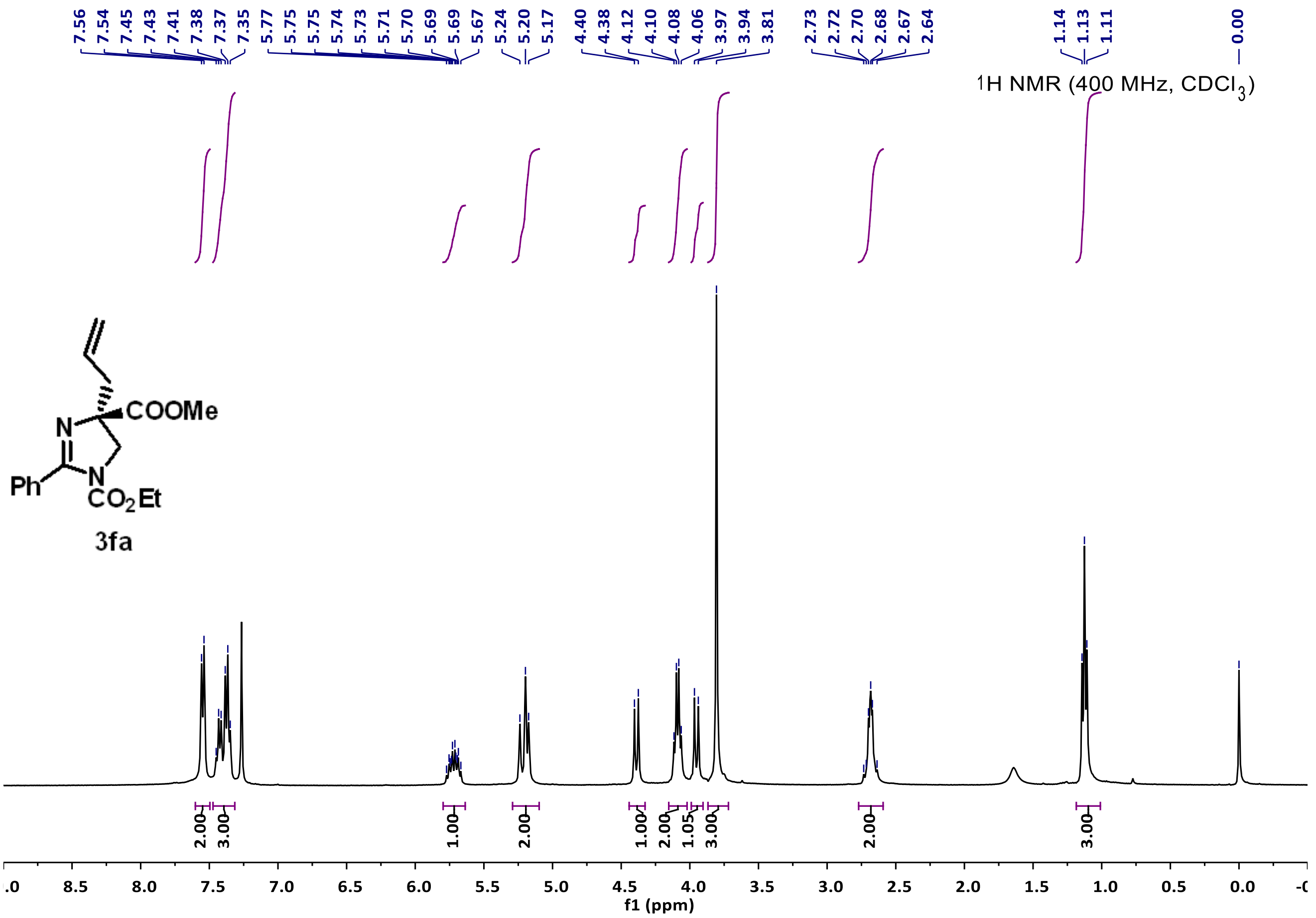

Supplementary Fig. 71. ^{1}H NMR spectrum of compound **3fa**

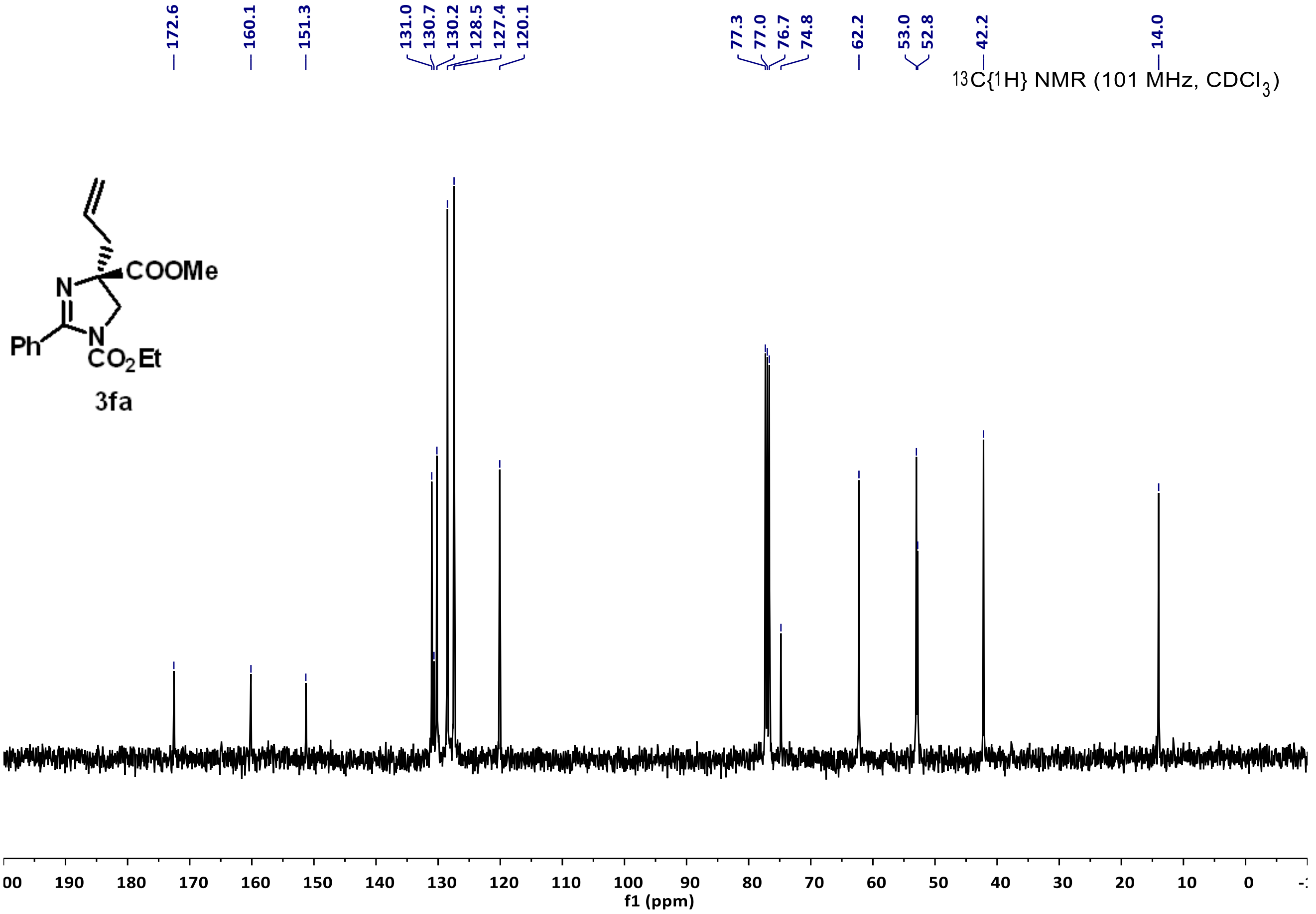

Supplementary Fig. 72. ^{13}C NMR spectrum of compound **3fa**

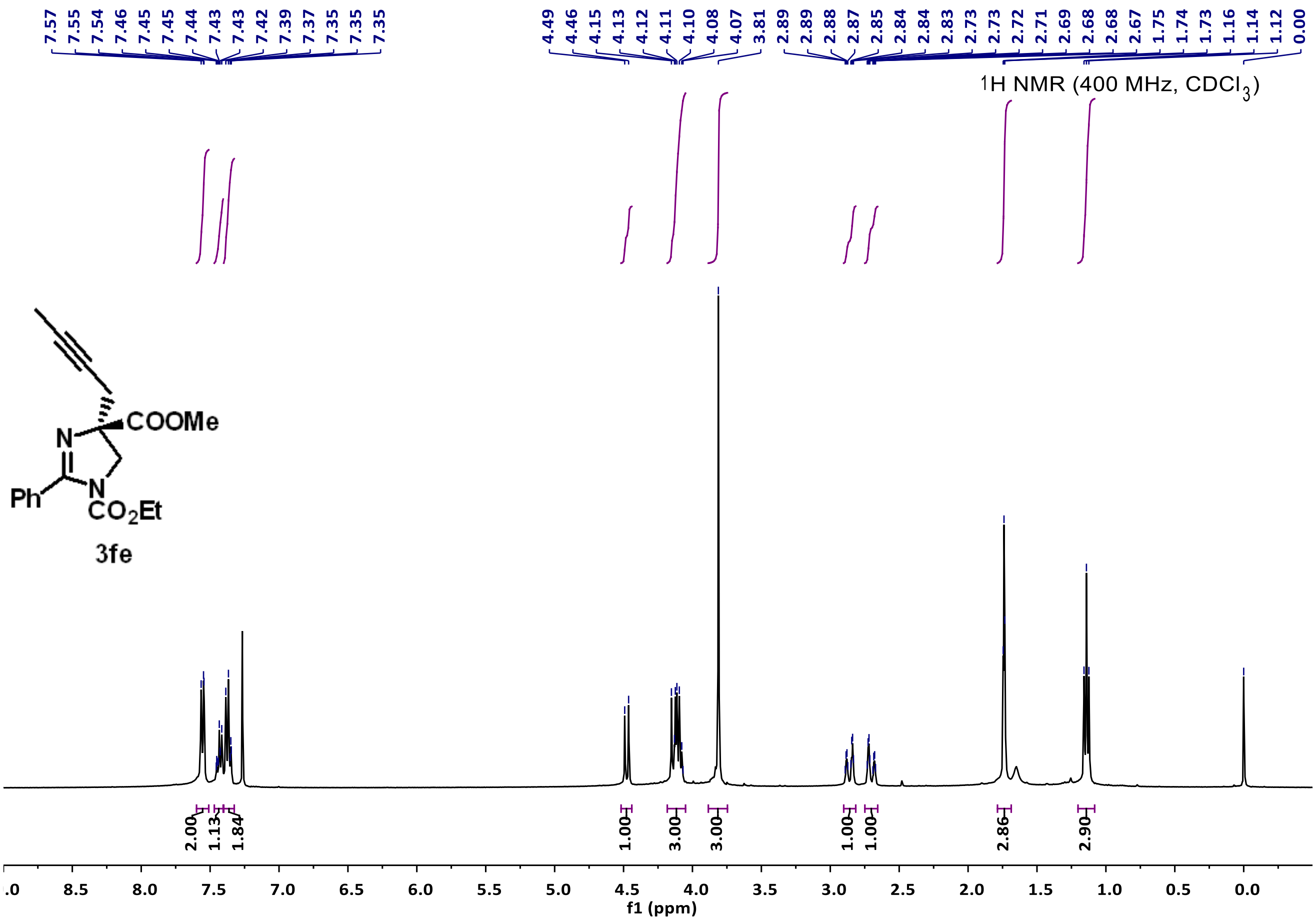

Supplementary Fig. 73. ^{1}H NMR spectrum of compound **3fe**

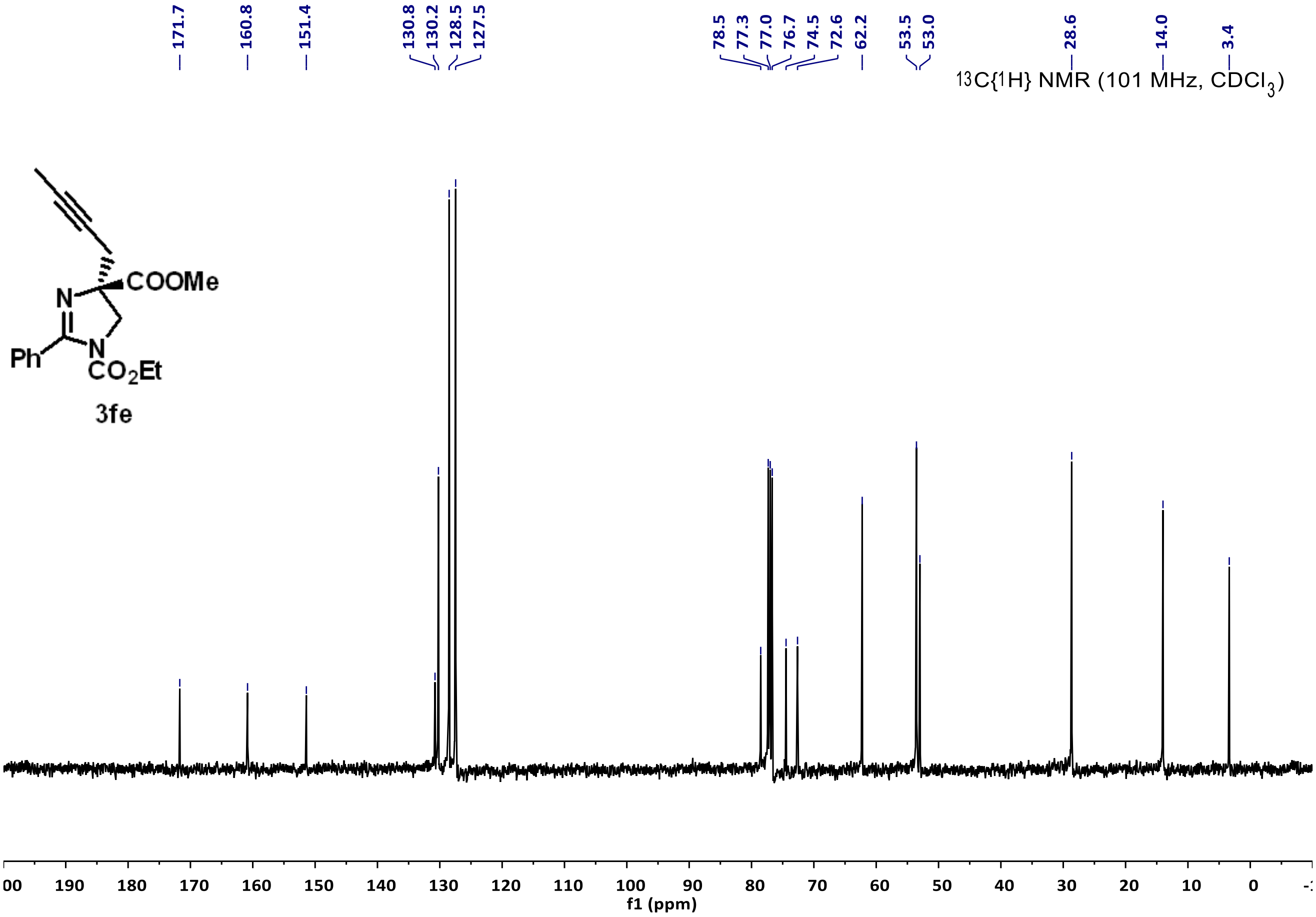

Supplementary Fig. 74. ^{13}C NMR spectrum of compound **3fe**

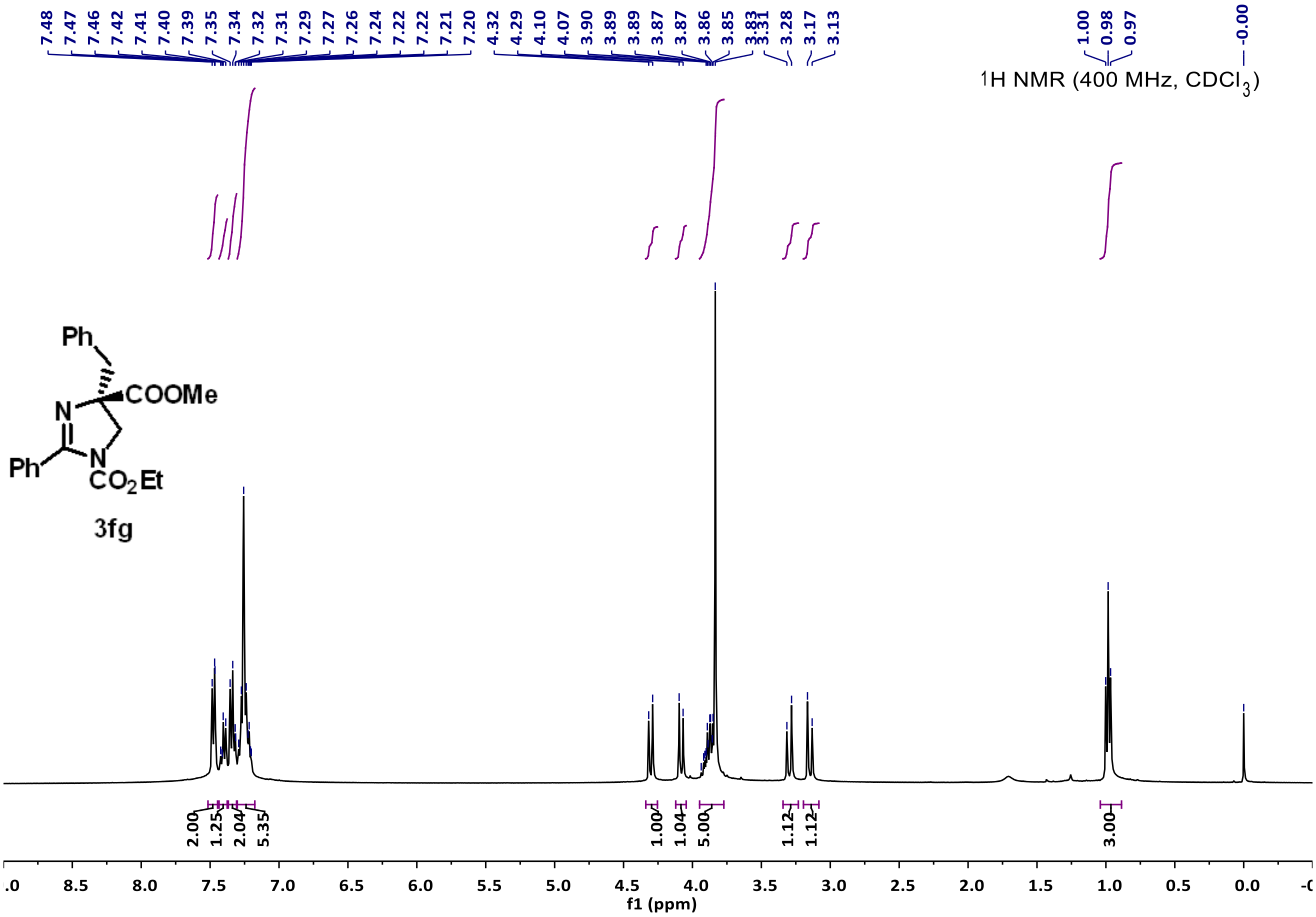

Supplementary Fig. 75. ^{1}H NMR spectrum of compound **3fg**

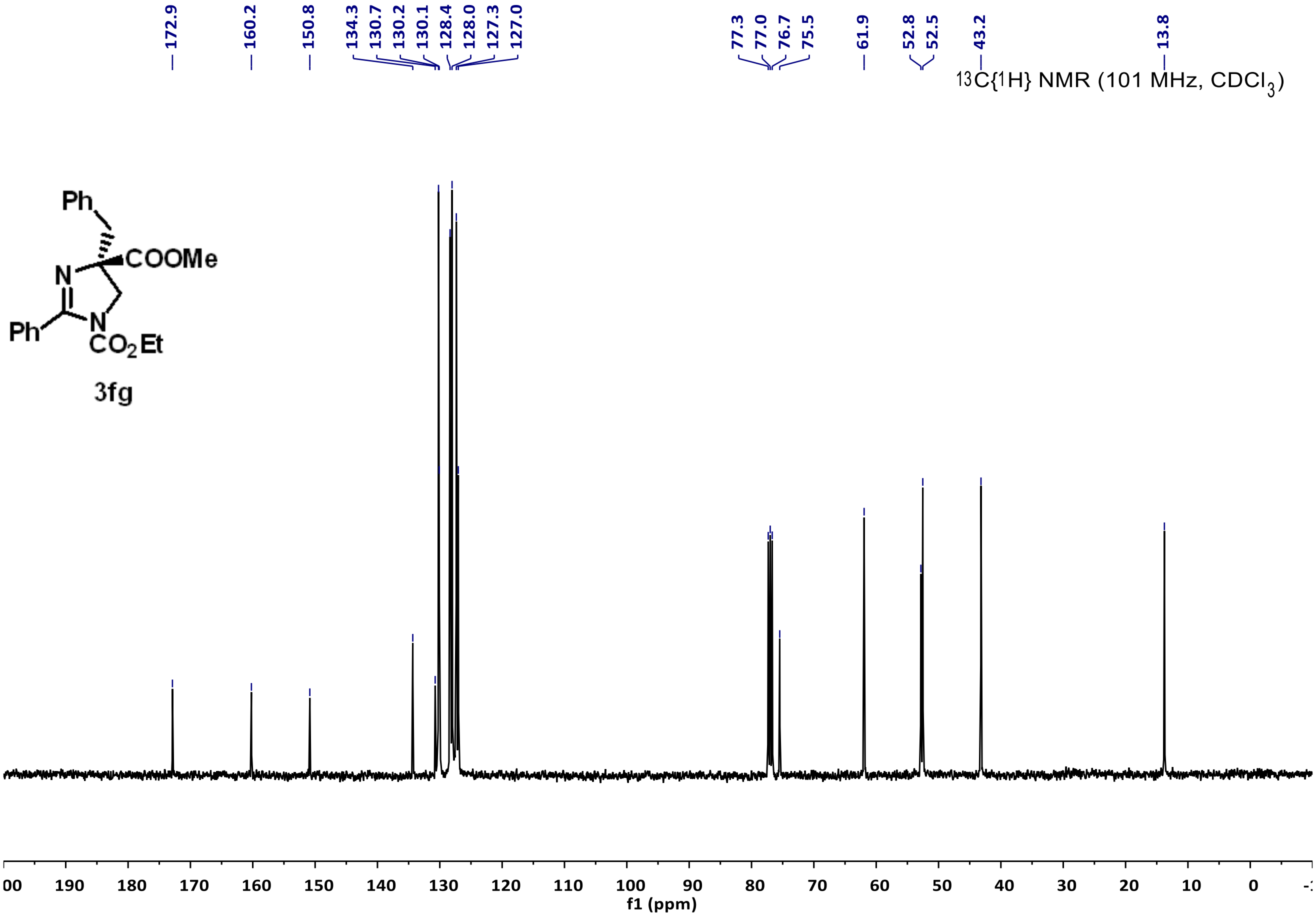

Supplementary Fig. 76. ^{13}C NMR spectrum of compound **3fg**

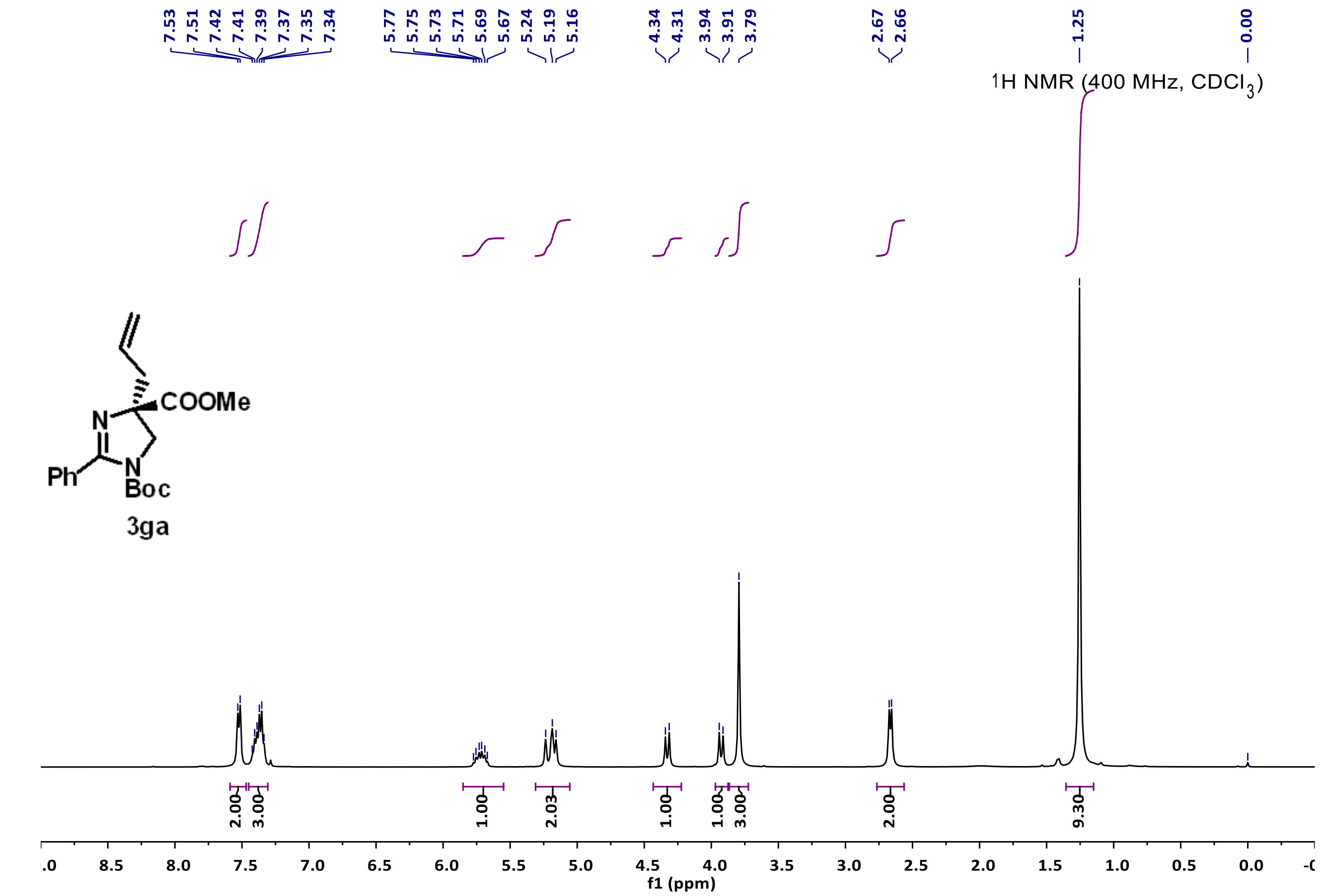

Supplementary Fig. 77. ^{1}H NMR spectrum of compound **3ga**

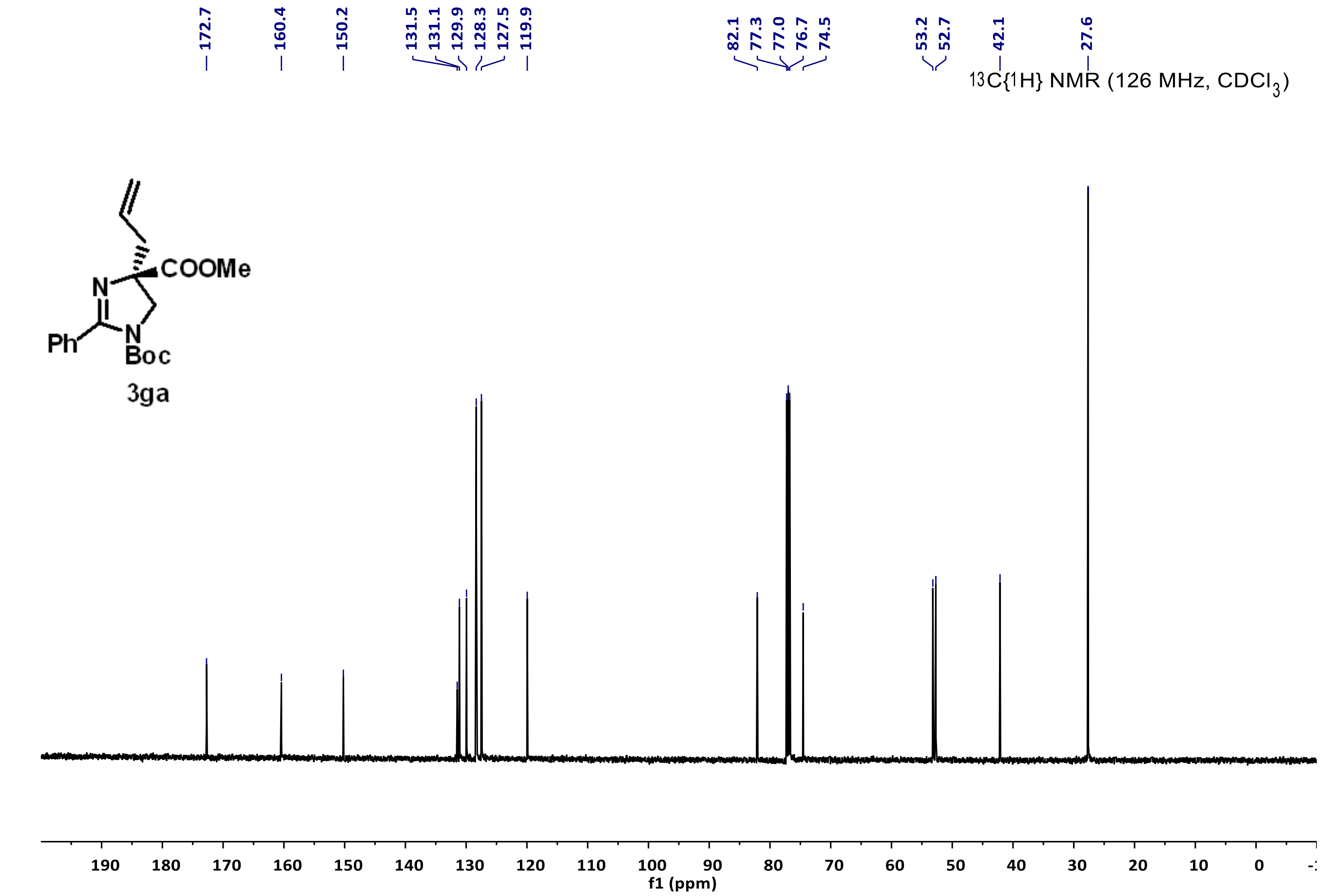

Supplementary Fig. 78. ^{13}C NMR spectrum of compound **3ga**

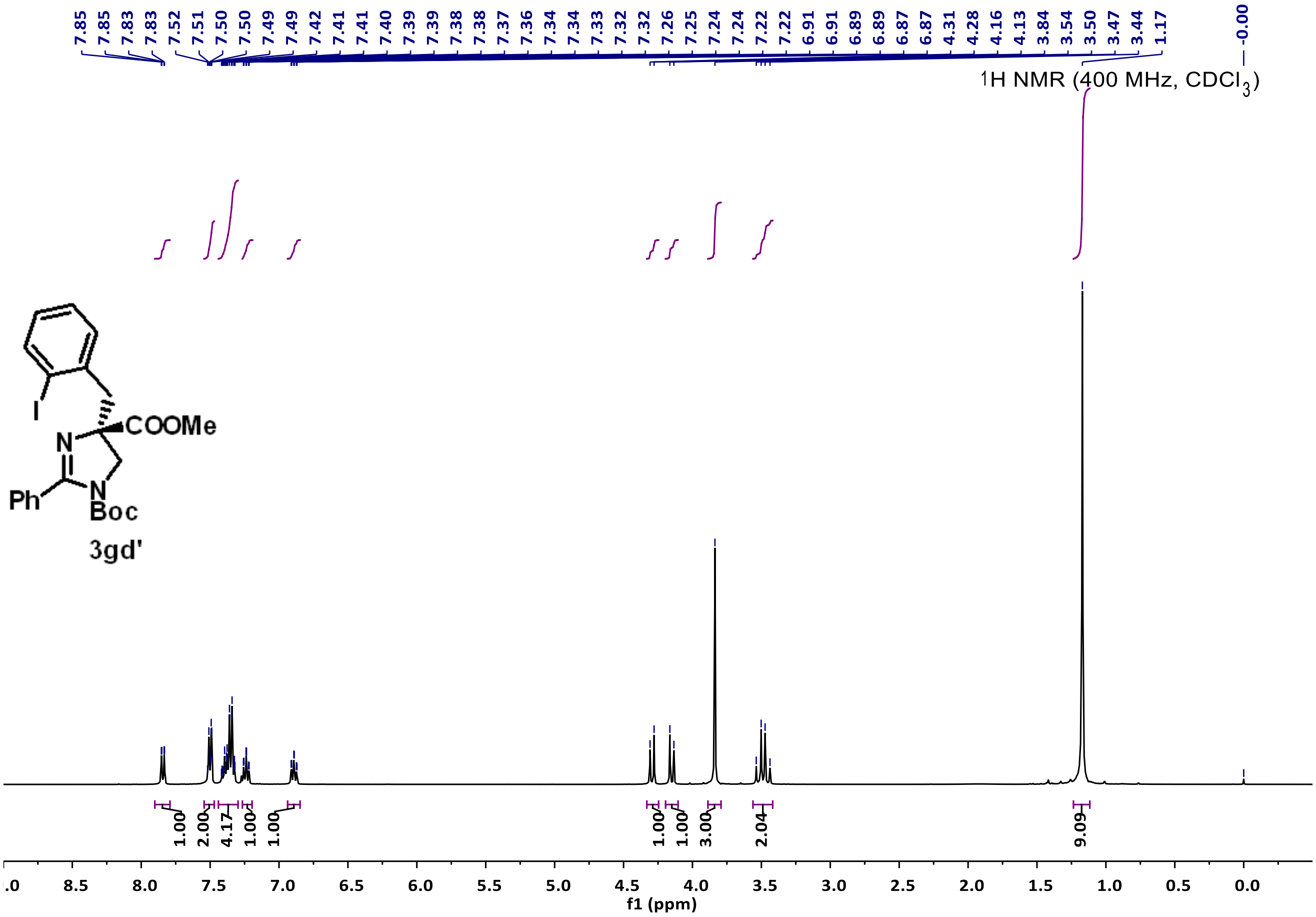

Supplementary Fig. 79. ^{1}H NMR spectrum of compound **3gd'**

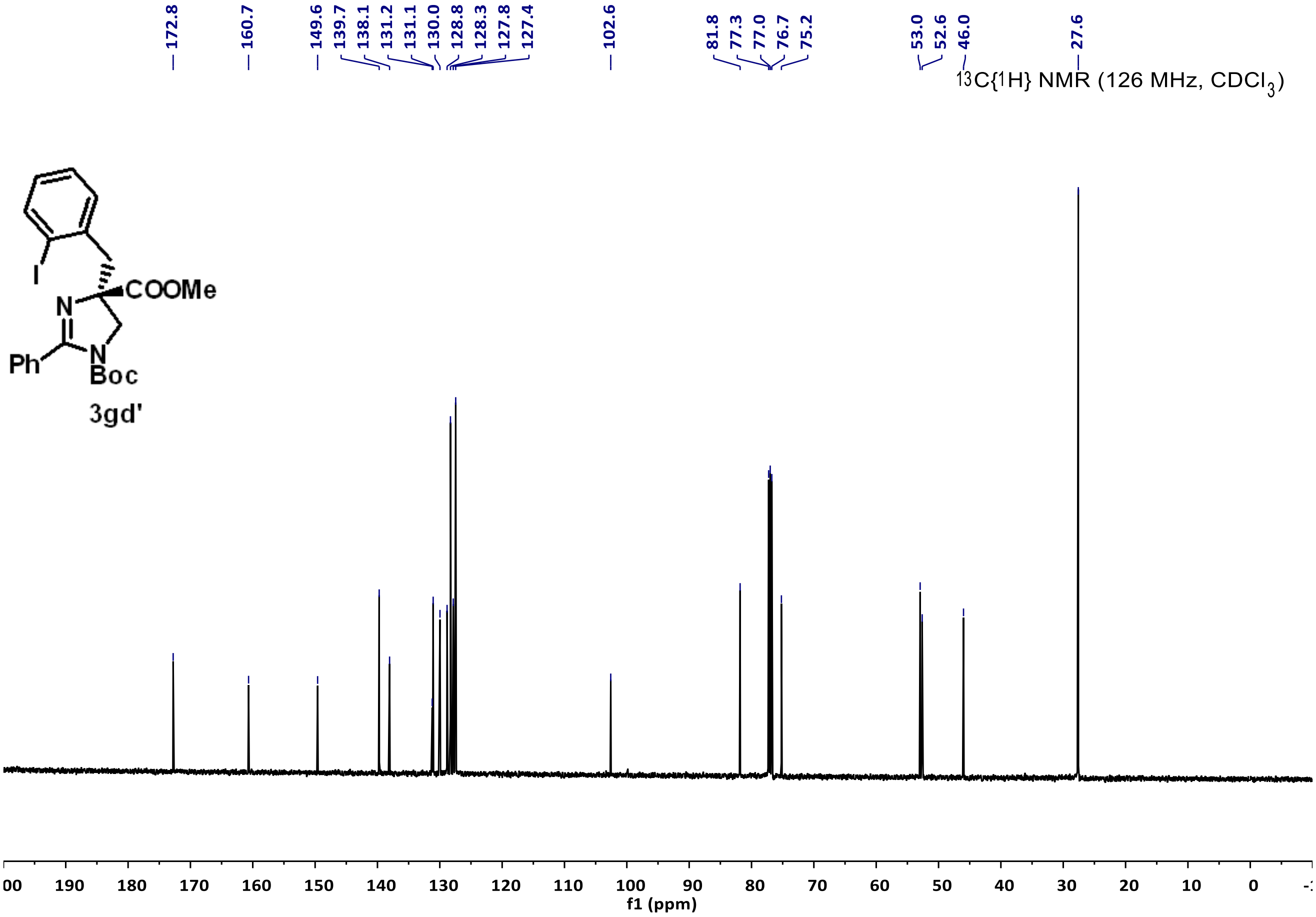

Supplementary Fig. 80. ^{13}C NMR spectrum of compound **3gd'**

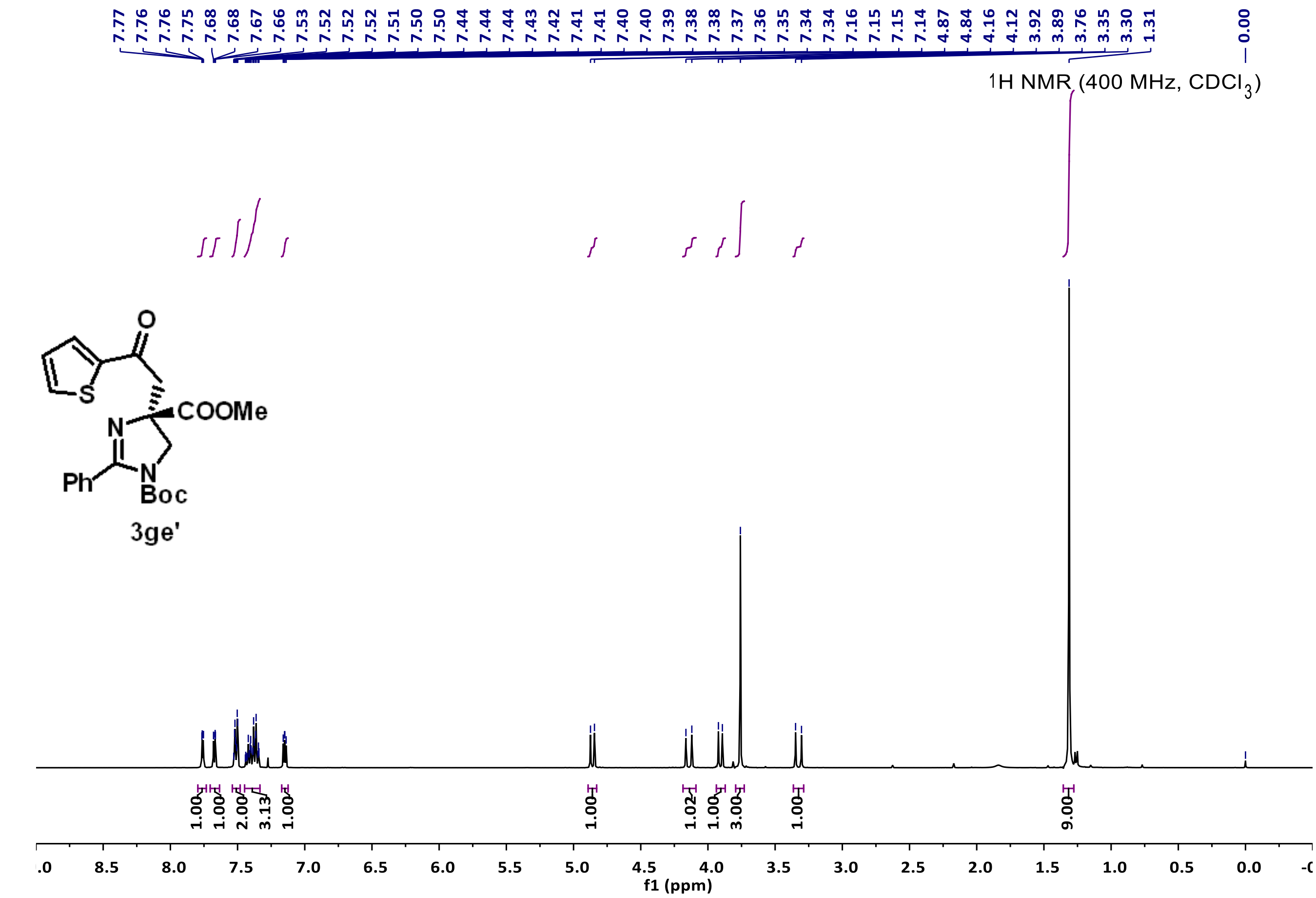

Supplementary Fig. 81. ^{1}H NMR spectrum of compound **3ge'**

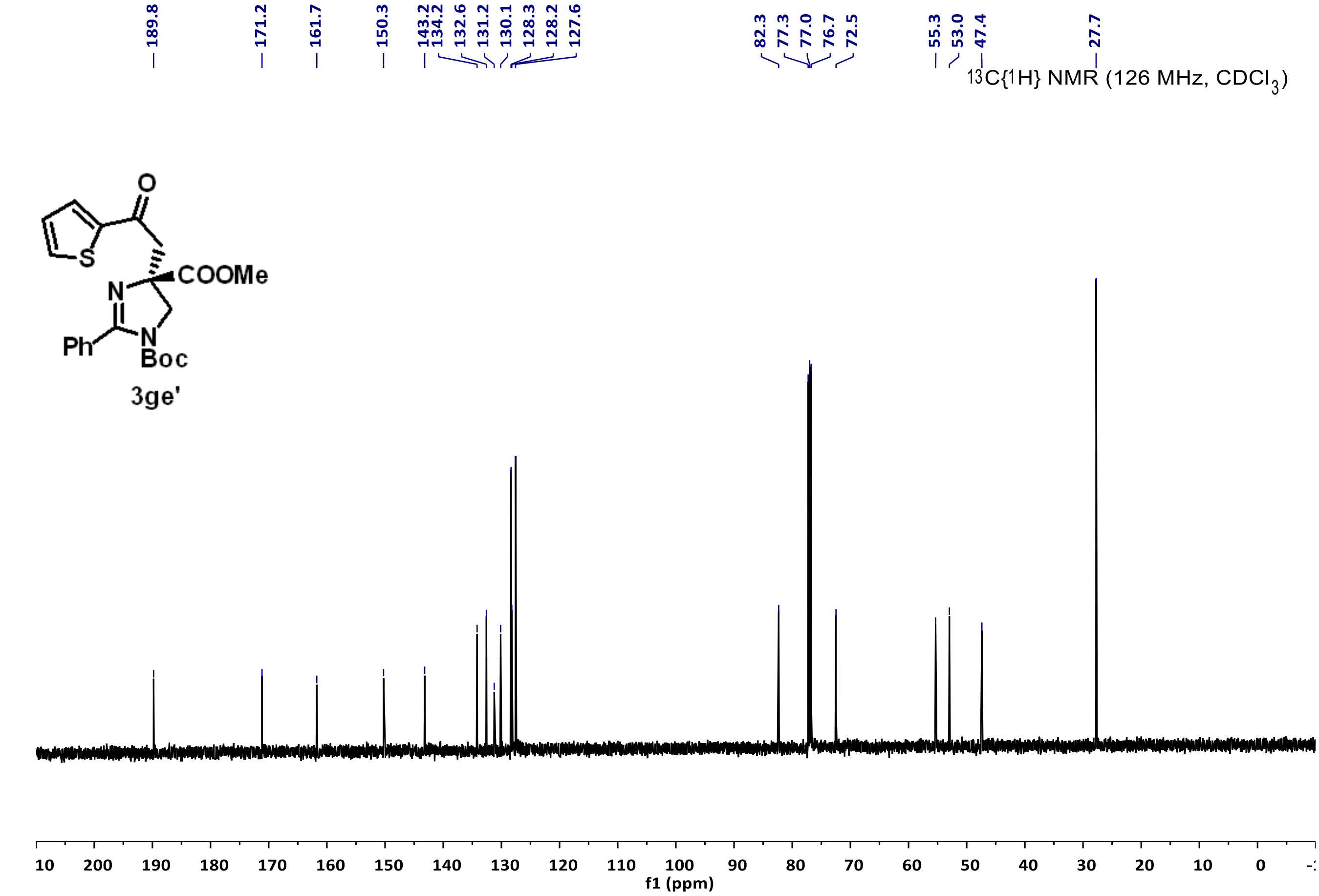

Supplementary Fig. 82. ^{13}C NMR spectrum of compound **3ge'**

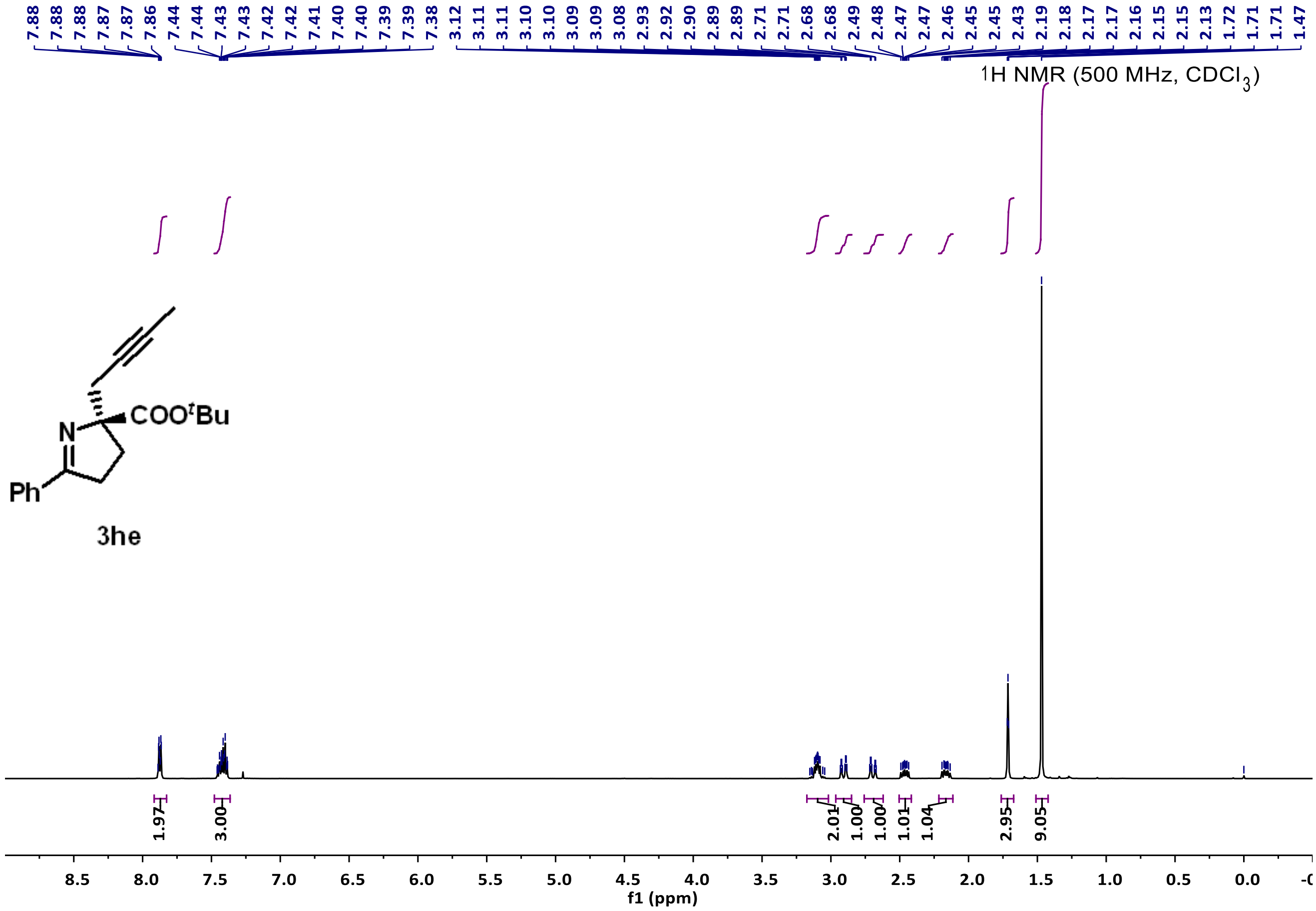

Supplementary Fig. 83. ^{1}H NMR spectrum of compound **3he**

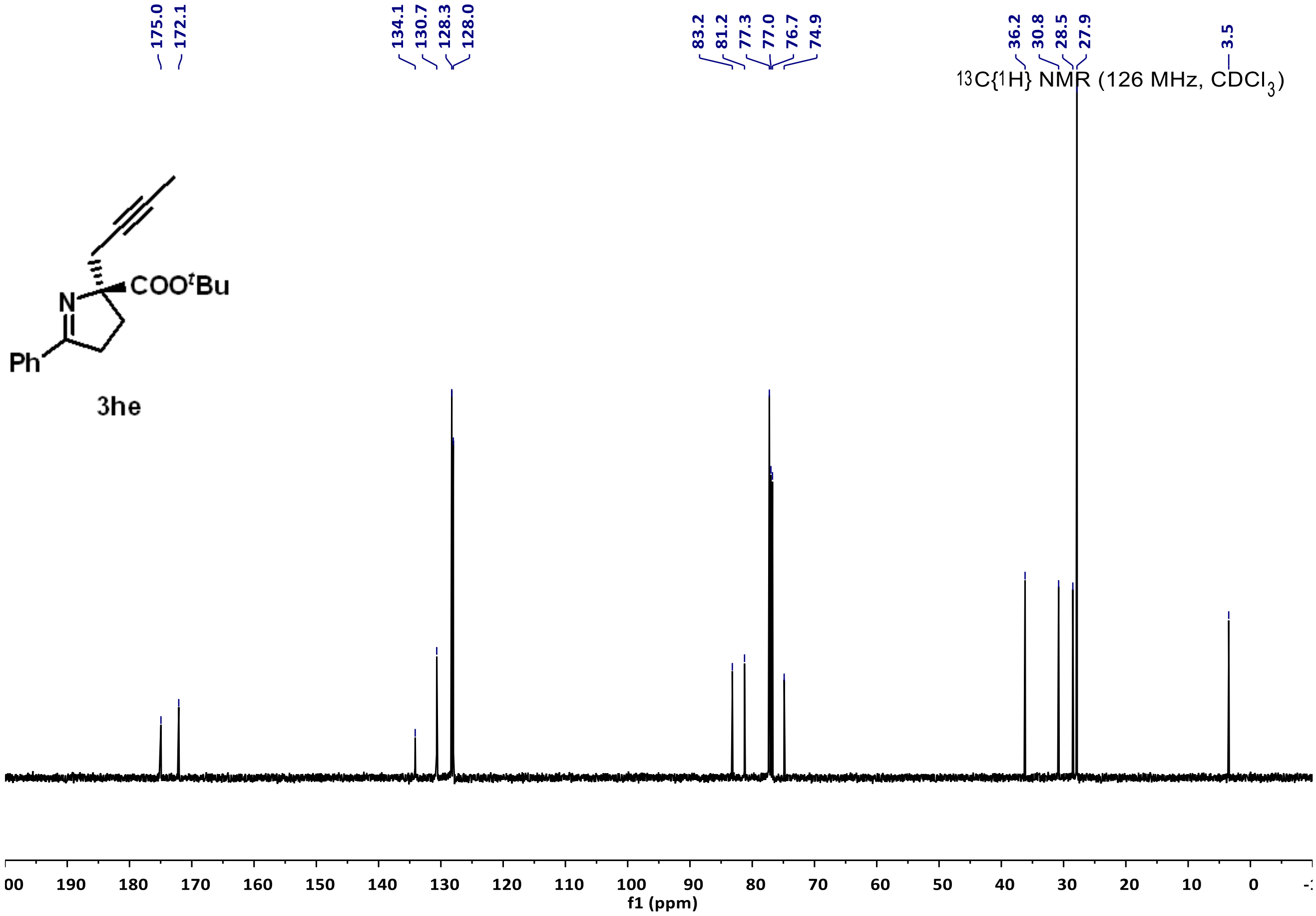

Supplementary Fig. 84. ^{13}C NMR spectrum of compound **3he**

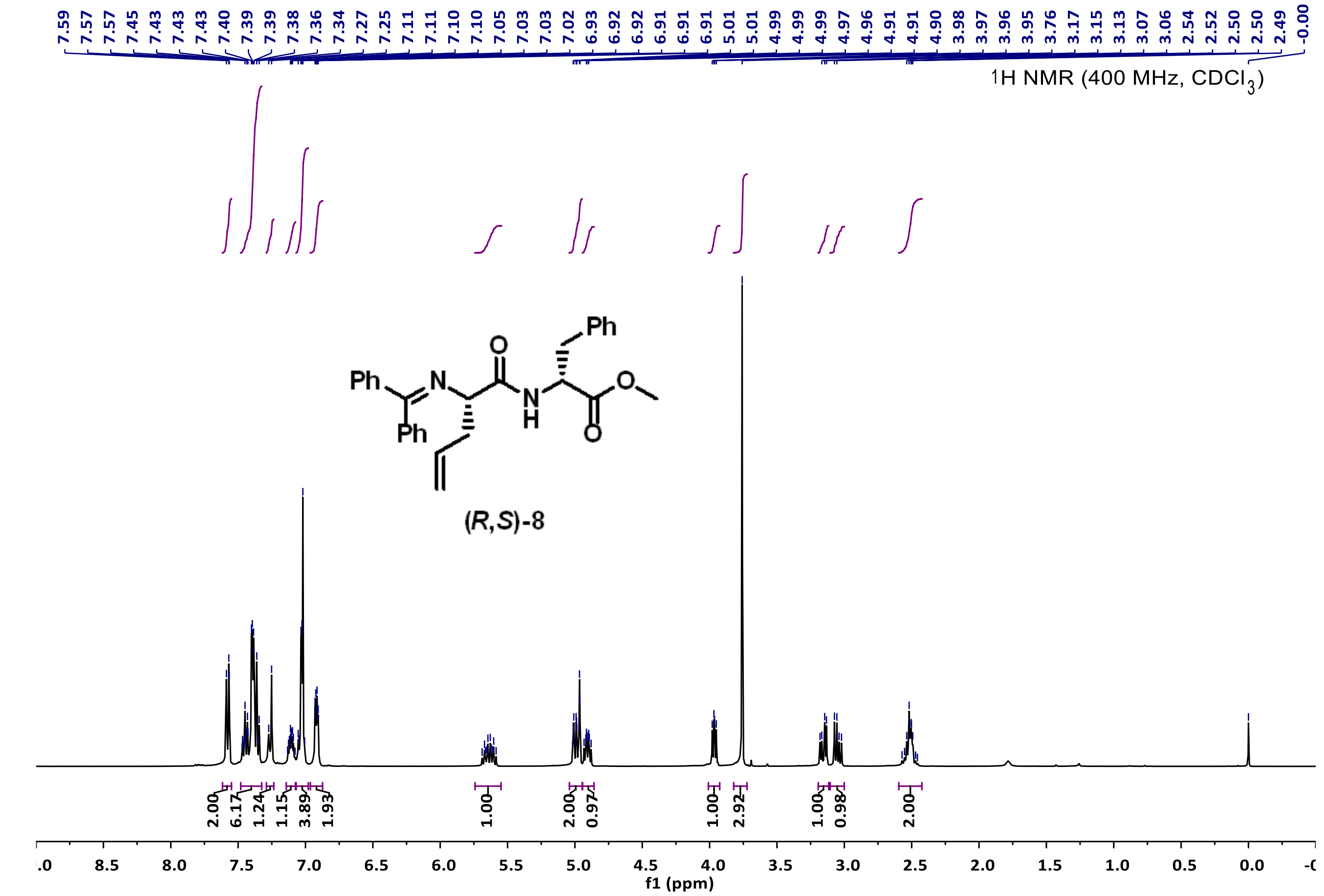

Supplementary Fig. 85. ^{1}H NMR spectrum of compound **(*R,S*)-8**

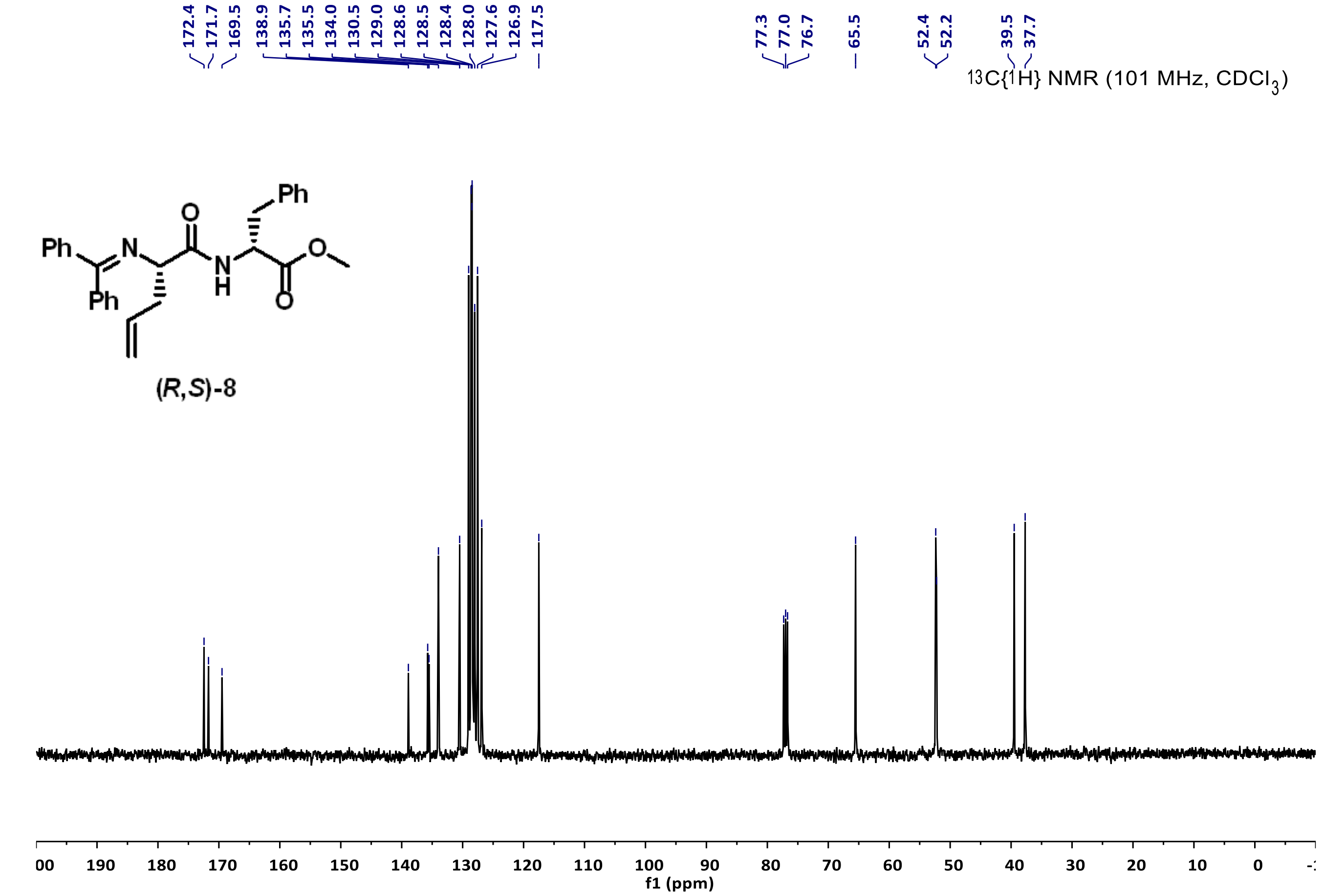

Supplementary Fig. 86. ^{13}C NMR spectrum of compound **(*R,S*)-8**

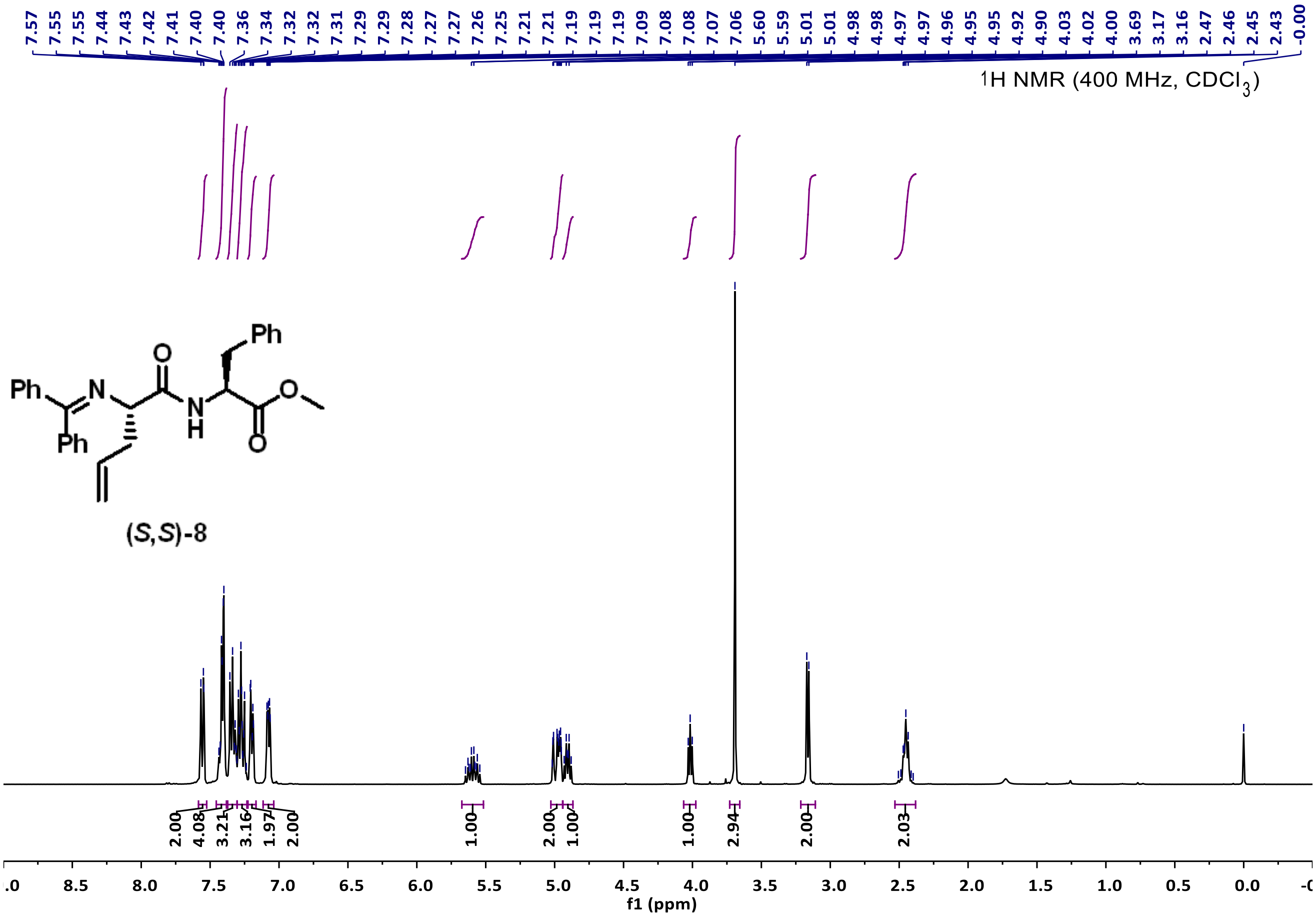

Supplementary Fig. 87. ^{1}H NMR spectrum of compound **(*S*,*S*)-8**

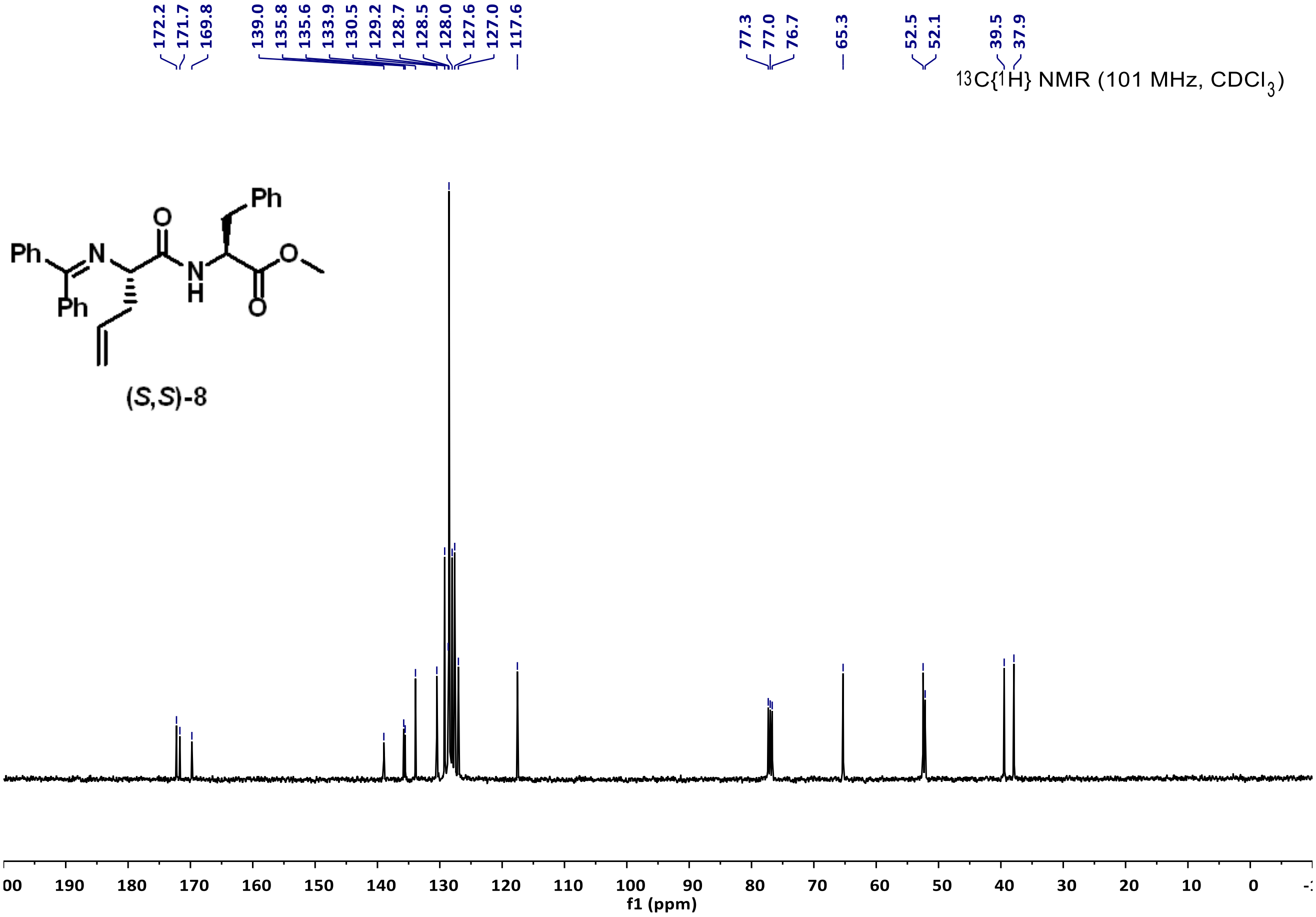

Supplementary Fig. 88. ^{13}C NMR spectrum of compound **(*S*,*S*)-8**

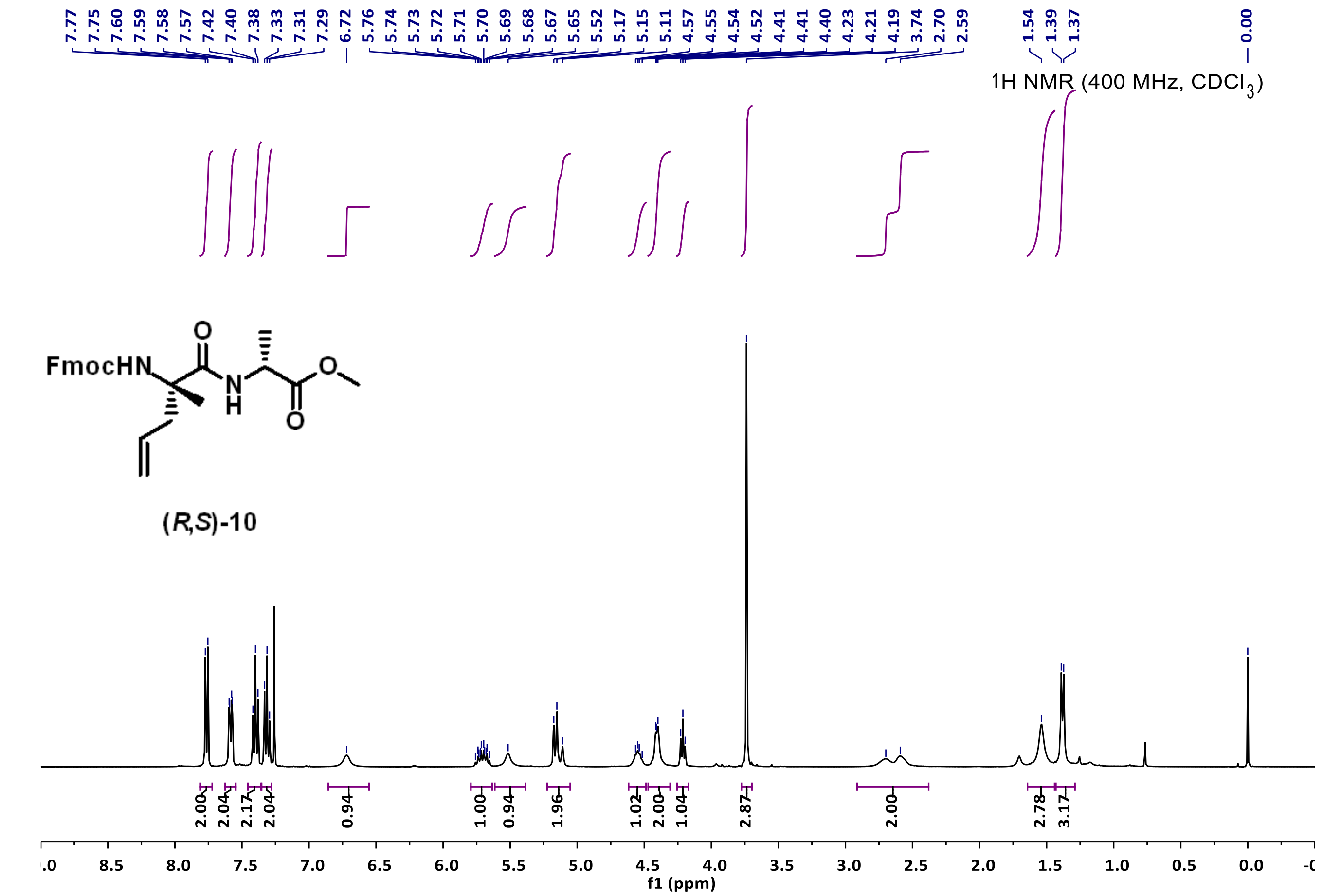

Supplementary Fig. 89. ^{1}H NMR spectrum of compound ***(R,S)*-10**

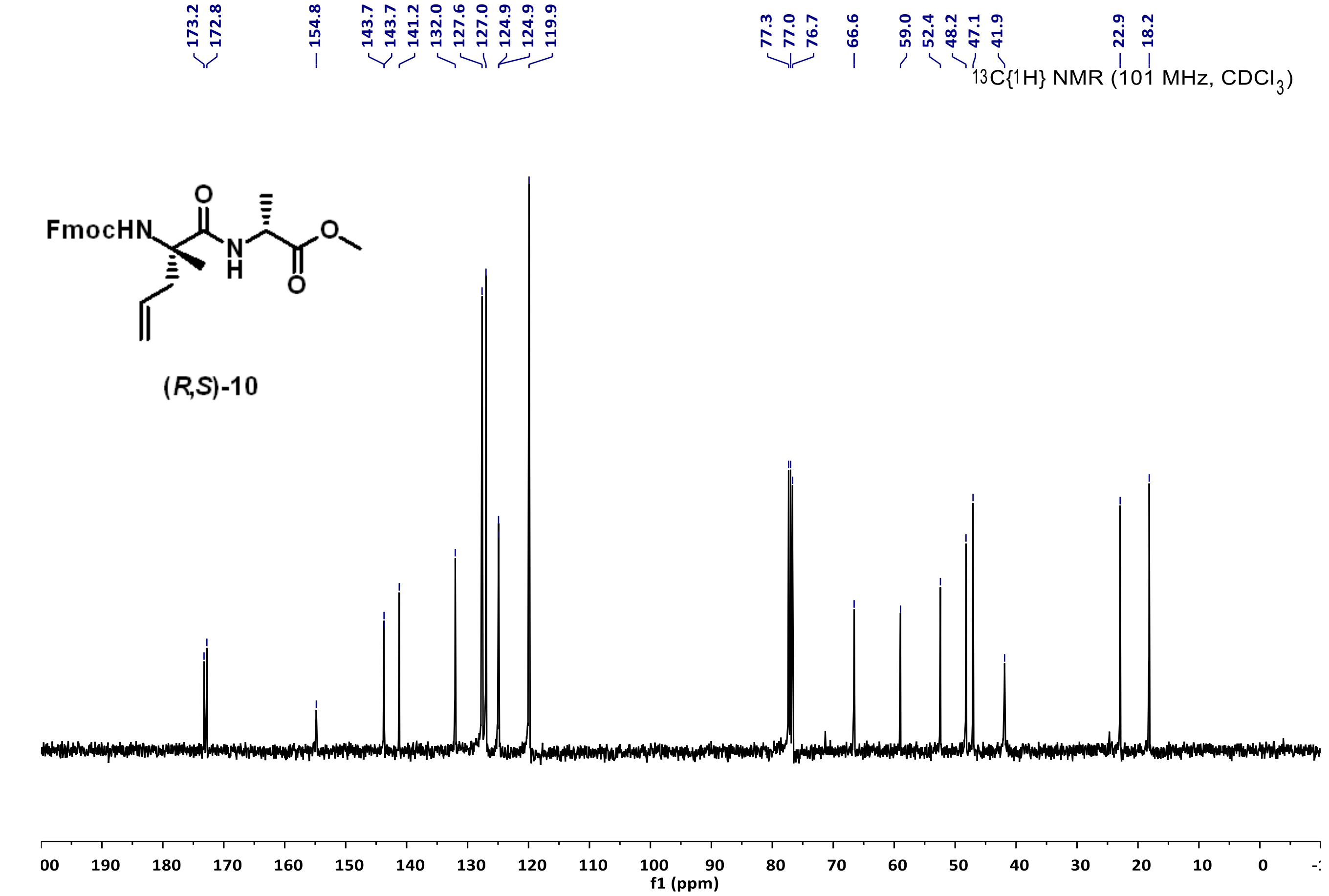

Supplementary Fig. 90. ^{13}C NMR spectrum of compound ***(R,S)*-10**

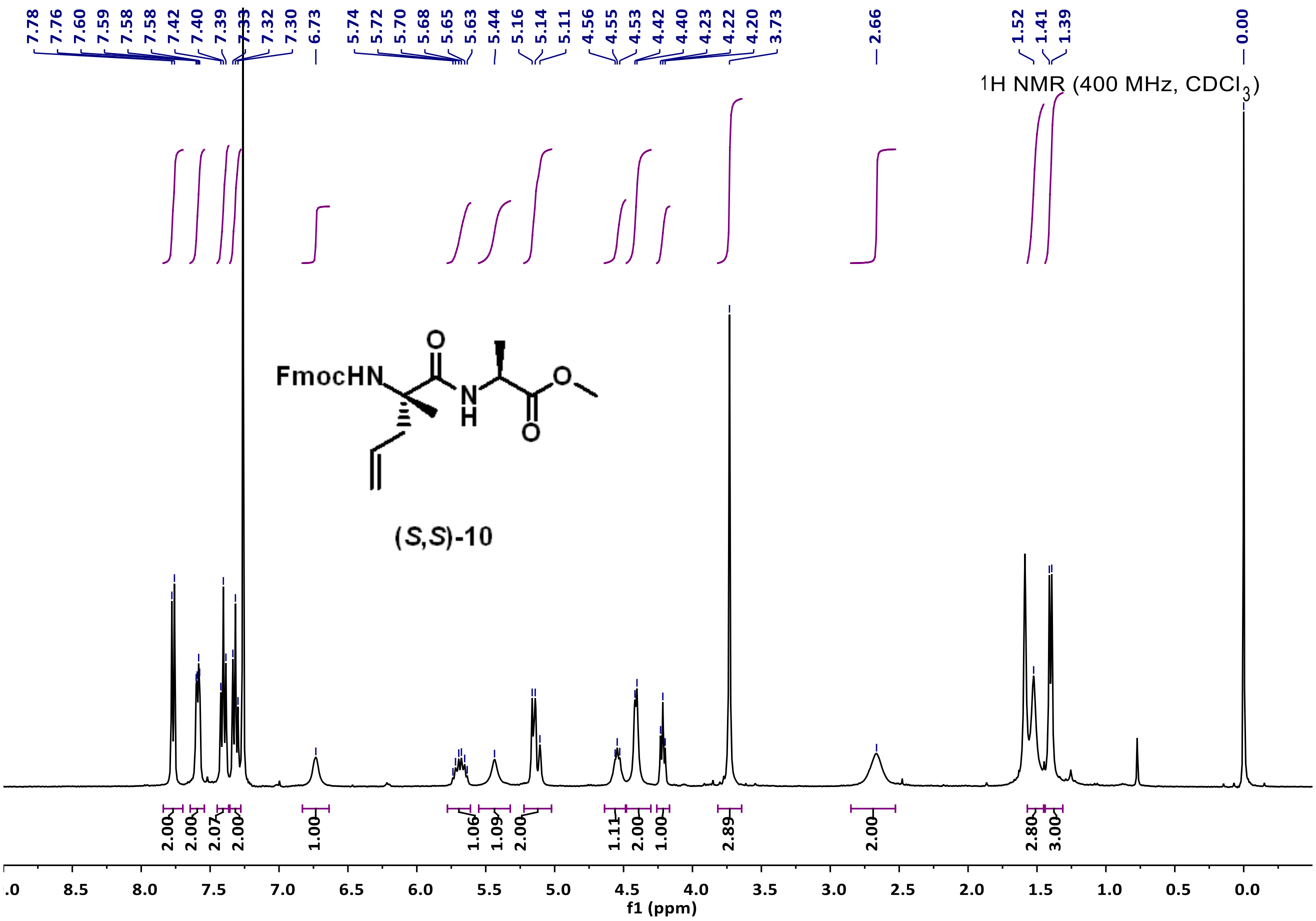

Supplementary Fig. 91. ^{1}H NMR spectrum of compound **(*S,S*)-10**

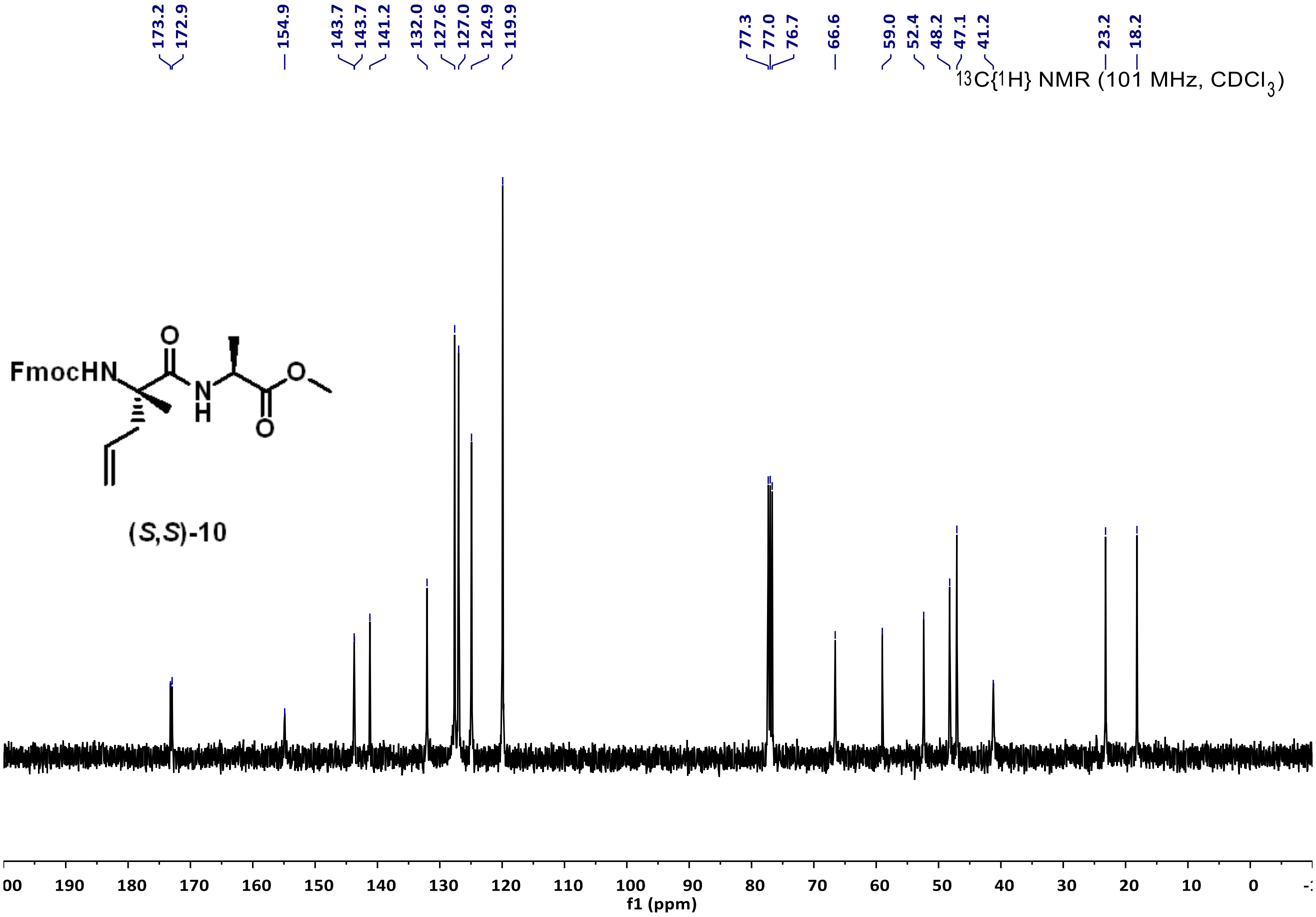

Supplementary Fig. 92. ^{13}C NMR spectrum of compound **(*S,S*)-10**

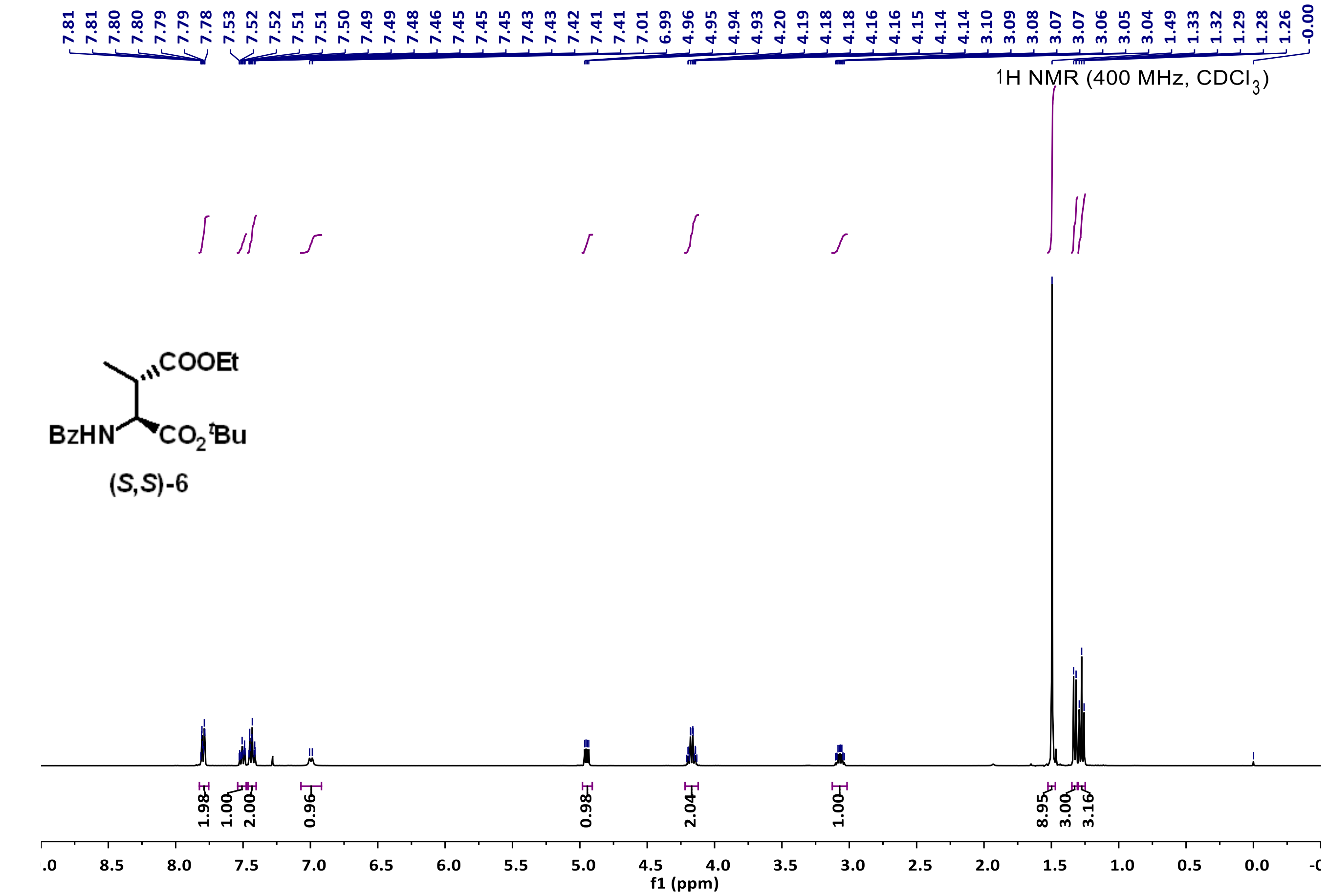

Supplementary Fig. 93. ^{1}H NMR spectrum of compound **(*S*,*S*)-6**

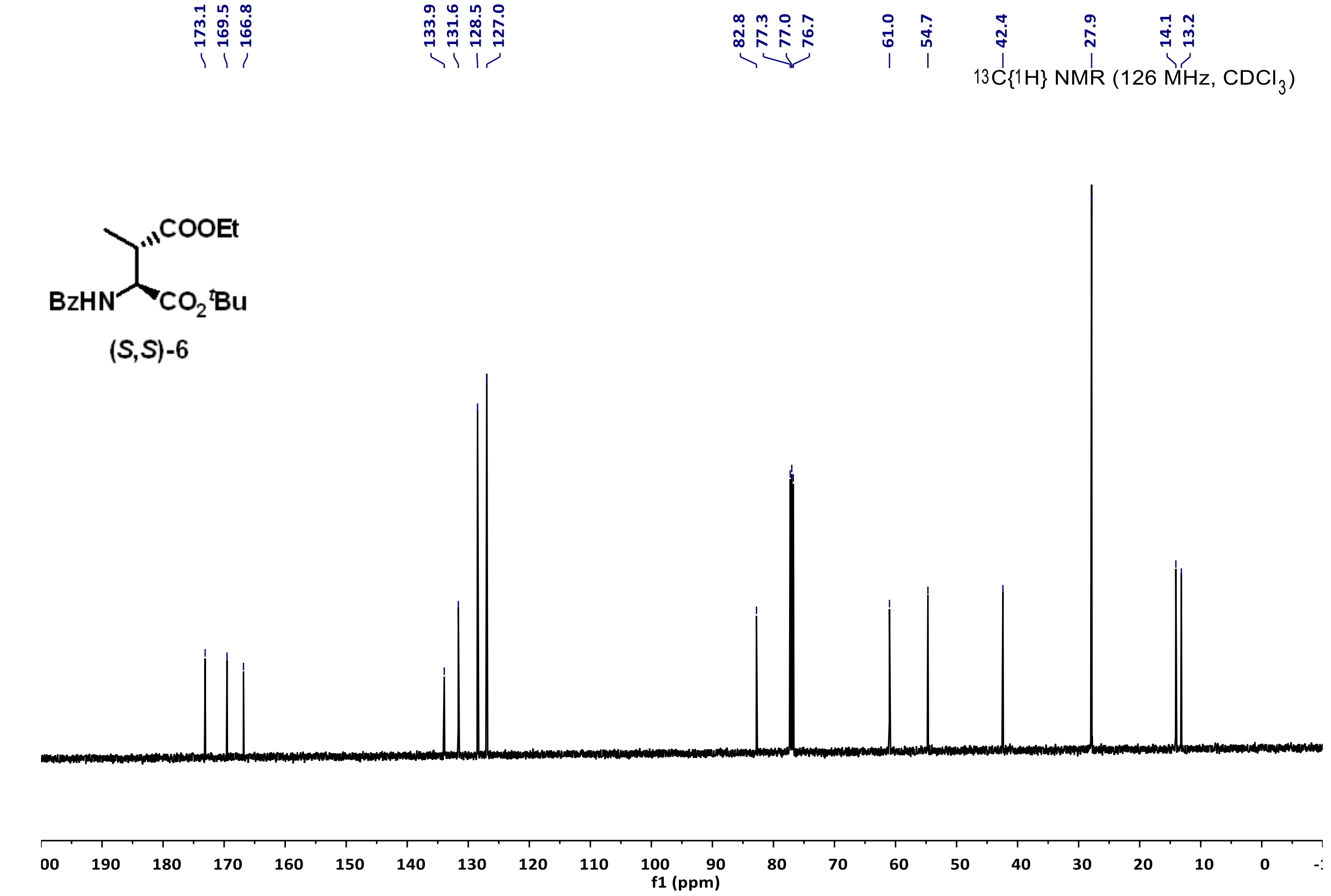

Supplementary Fig. 94. ^{13}C NMR spectrum of compound **(*S*,*S*)-6**

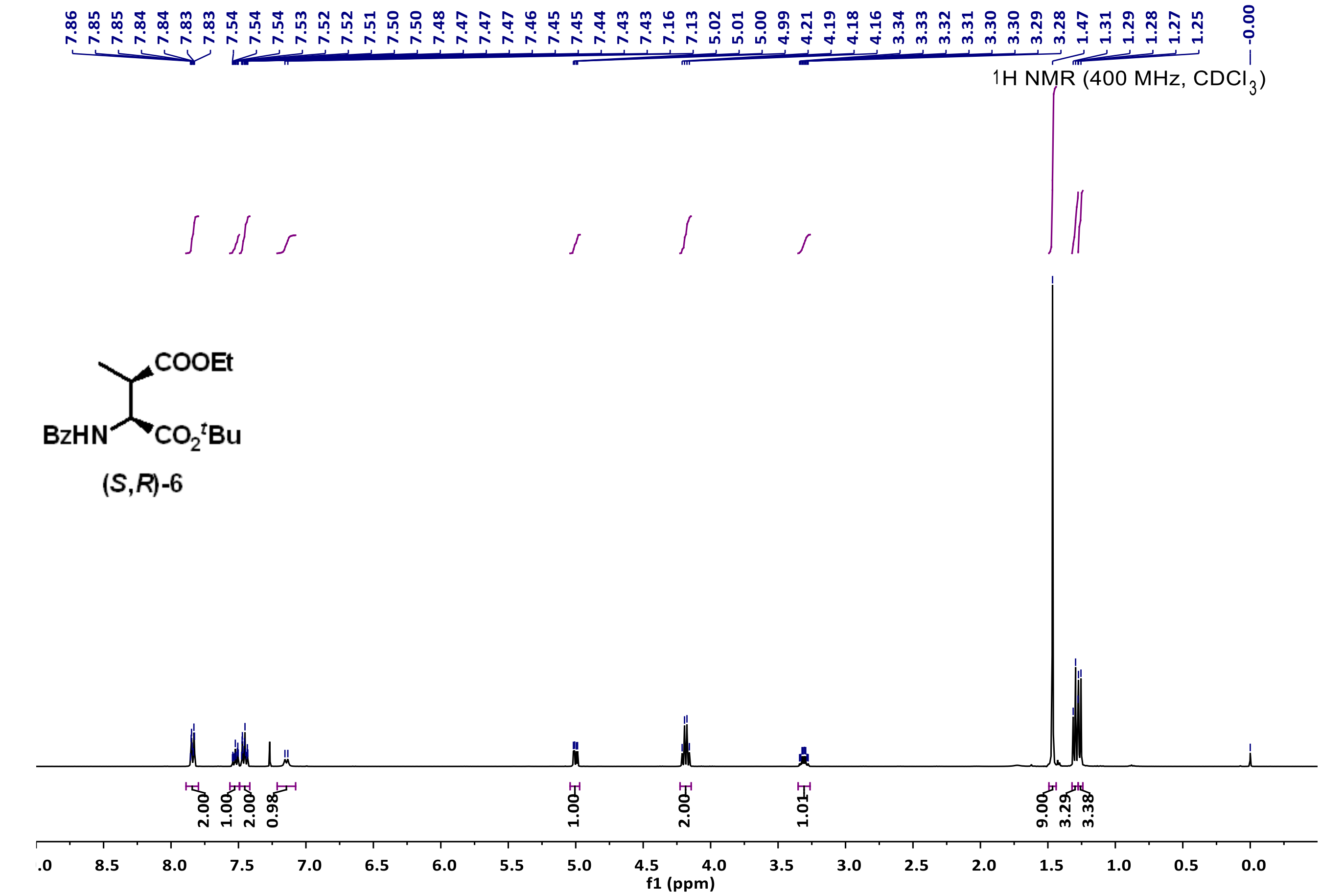

Supplementary Fig. 95. ^{1}H NMR spectrum of compound **(*S*,*R*)-6**

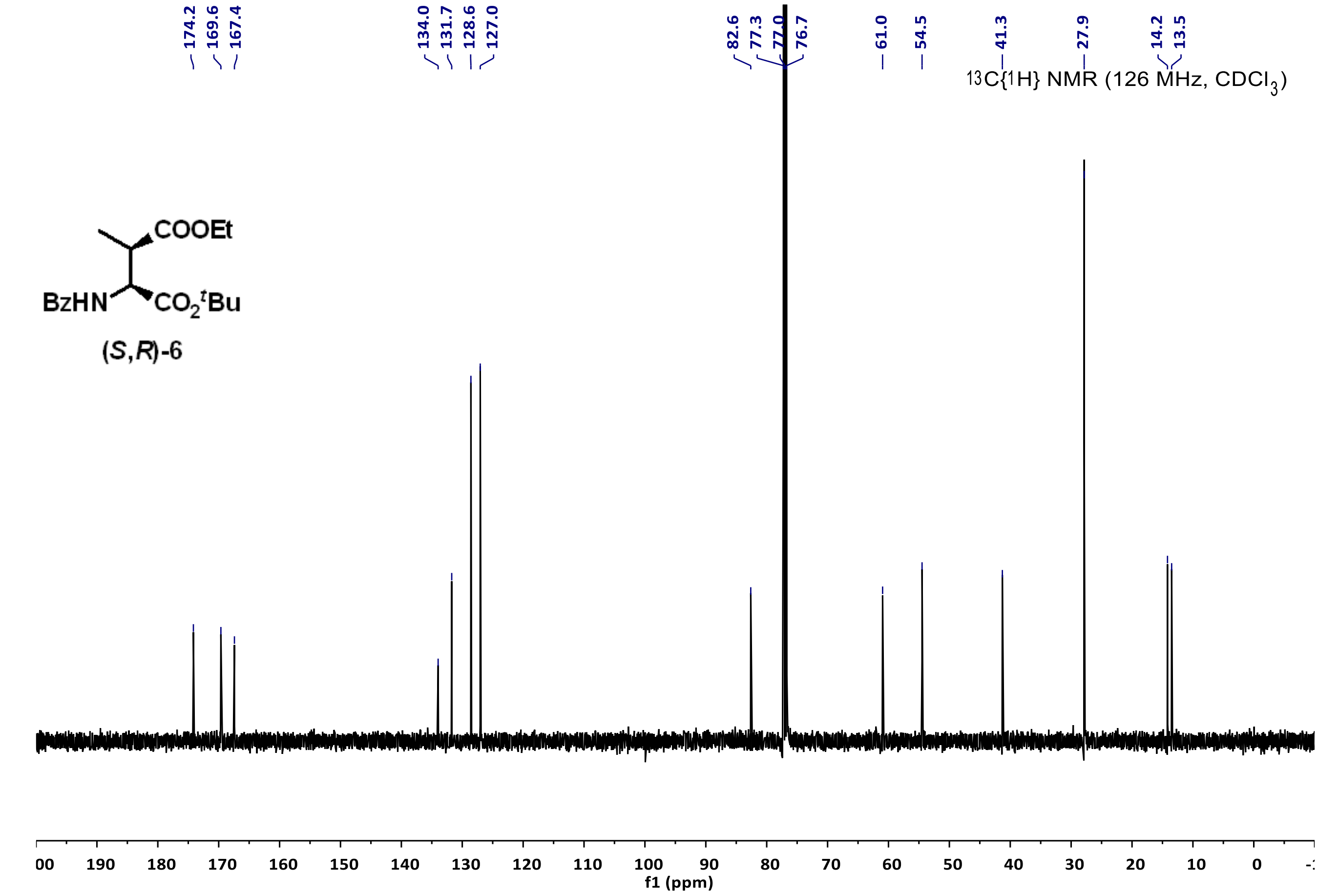

Supplementary Fig. 96. ^{13}C NMR spectrum of compound **(*S*,*R*)-6**
